# Early Mechanisms of Pathobiology Are Revealed by Transcriptional Temporal Dynamics in Hippocampal CA1 Neurons of Prion Infected Mice

**DOI:** 10.1371/journal.ppat.1003002

**Published:** 2012-11-08

**Authors:** Anna Majer, Sarah J. Medina, Yulian Niu, Bernard Abrenica, Kathy J. Manguiat, Kathy L. Frost, Clark S. Philipson, Debra L. Sorensen, Stephanie A. Booth

**Affiliations:** 1 Molecular PathoBiology, National Microbiology Laboratory, Public Health Agency of Canada, Winnipeg, Manitoba, Canada; 2 Department of Medical Microbiology and Infectious Diseases, University of Manitoba, Winnipeg, Manitoba, Canada; University of Alberta, Canada

## Abstract

Prion diseases typically have long pre-clinical incubation periods during which time the infectious prion particle and infectivity steadily propagate in the brain. Abnormal neuritic sprouting and synaptic deficits are apparent during pre-clinical disease, however, gross neuronal loss is not detected until the onset of the clinical phase. The molecular events that accompany early neuronal damage and ultimately conclude with neuronal death remain obscure. In this study, we used laser capture microdissection to isolate hippocampal CA1 neurons and determined their pre-clinical transcriptional response during infection. We found that gene expression within these neurons is dynamic and characterized by distinct phases of activity. We found that a major cluster of genes is altered during pre-clinical disease after which expression either returns to basal levels, or alternatively undergoes a direct reversal during clinical disease. Strikingly, we show that this cluster contains a signature highly reminiscent of synaptic N-methyl-D-aspartic acid (NMDA) receptor signaling and the activation of neuroprotective pathways. Additionally, genes involved in neuronal projection and dendrite development were also altered throughout the disease, culminating in a general decline of gene expression for synaptic proteins. Similarly, deregulated miRNAs such as miR-132-3p, miR-124a-3p, miR-16-5p, miR-26a-5p, miR-29a-3p and miR-140-5p follow concomitant patterns of expression. This is the first in depth genomic study describing the pre-clinical response of hippocampal neurons to early prion replication. Our findings suggest that prion replication results in the persistent stimulation of a programmed response that is mediated, at least in part, by synaptic NMDA receptor activity that initially promotes cell survival and neurite remodelling. However, this response is terminated prior to the onset of clinical symptoms in the infected hippocampus, seemingly pointing to a critical juncture in the disease. Manipulation of these early neuroprotective pathways may redress the balance between degeneration and survival, providing a potential inroad for treatment.

## Author Summary

Neurodegenerative diseases affect an ever-increasing proportion of the population; therefore, there is an urgent need to develop treatments. Prion disorders belong to this group of diseases and although rare and uniquely transmissible, share many features on a sub-cellular level. Central to disease is progressive synaptic impairment that invariably leads to the irreversible loss of neurons. Understanding this process is undoubtedly essential for rational drug discovery. In this study we looked at neurons very early in disease, when prions are barely detectable and there are no clinical symptoms observed. Specifically, we performed a comprehensive analysis of transcriptional changes within a particularly dense area of neurons, the CA1 hippocampus region, from prion-infected and control mice. In this way we were able to enrich our data for molecular changes unique to neurons and minimize those changes characteristic of support cells such as astrocytes and microglia. We detected the activation of a transcriptional program indicative of a protective mechanism within these neurons early in disease. This mechanism diminished as disease progressed and was lost altogether, concurrently with the onset of clinical symptoms. These findings demonstrate the ability of neurons to mount an initial neuroprotective response to prions that could be exploited for therapy development.

## Introduction

Prion diseases, or transmissible spongiform encephalopathies (TSEs), are fatal neurodegenerative diseases that affect both humans and animals. The conversion of the normal prion protein, PrP^C^ (cellular prion protein), to the infectious form, PrP^Sc^ (Scrapie prion protein), is responsible for the disease pathology which is characterized by deposits of protease-resistant prion protein (PrP^Res^), extensive vacuolation plus microgliosis and astrocytosis. Although this protein conversion typically occurs over a long pre-clinical incubation period, neuronal dysfunction has been observed at these non-symptomatic stages of disease. In particular, synaptic loss was identified in animal models of prion disease prior to the manifestation of clinical symptoms [1]–[4]. This pathological change must be driven by the activation of molecular responses in prion affected cells during these long pre-clinical sequelae. Currently, the specific cellular responses by neurons to the presence of PrP^Res^, whether directly or indirectly associated, remain largely unknown. Identifying these specific pathways during prion disease progression, especially at pre-clinical stages of disease, may potentially help guide research in the understanding of prion-induced pathobiology.

Assessing global gene expression patterns to identify perturbed transcriptional networks during disease provides insight into the molecular pathobiology of prion diseases. To this end, numerous gene expression studies have been performed for many prion and mouse genetic strains with the aim of identifying affected pathways during disease [5]–[13]. Many of these studies investigated the changes in gene expression profiles over time from whole brain tissue by looking at both pre-clinical and clinical stages of disease. Nevertheless, the progressive genetic changes that eventually lead to neuronal death, or the mechanism by which neurotoxicity occurs, still remain unresolved. Typically, high-throughput studies on whole brain tissue are very challenging to interpret because of the cellular and functional complexity of the brain. Brain tissue is made up of a myriad of neuronal cell types that work together in intricate cellular networks. Adding to this complexity is the multitude of supporting cells such as astrocytes, microglia and oligodendrocytes that outnumber neurons by at least 10∶1, even in the healthy brain. Therefore, whole brain mRNA profiles are representative of an abundance of cells masking temporal genetic changes that are restricted to similarly affected neurons.

To overcome this challenge, we used laser capture microdissection (LCM) technology to isolate neurons from brain tissue sections [14]–[17]. We chose to isolate neurons distinct to the *Cornu Ammonis* layer of the hippocampus (CA1) because neurons in this region are known to undergo damage and degeneration during the course of prion disease [2] and represent a relatively homogeneous population of cell bodies for RNA isolation. We used this strategy to isolate this small group of neurons, rather than individual cells as a compromise; testing too small a sample size would increase variability due to heterogeneity of individual neurons [17] and a low amount of starting material would limit sensitivity for detecting lower copy number transcripts. We chose to sample more than one serial section for our studies in order to increase the probability of capturing these disease-affected neurons, especially at early stages of disease when we presume that only some neurons in the region are similarly affected.

We performed a thorough high-throughput screen for messenger RNAs (mRNAs) and microRNAs (miRNAs), a class of small RNAs with important roles as post-transcriptional gene regulators [18], [19]. Despite their recent identification, a substantial research effort has already shown that miRNAs are pivotal in fundamental processes such as neuronal differentiation, development, plasticity and survival (for review, see [20]). Links between miRNA dysfunction and neurodegenerative diseases are also becoming increasingly apparent [21], [22]. This follows from the observation that the loss of miRNA expression in the brain by inactivation of miRNA processing leads to neurodegeneration in a number of animal models [23]–[26]. Augmentation of our mRNA profile with miRNA expression levels is an important step in gaining a more complete understanding of the molecular mechanisms that are affected during the progression of prion disease. Furthermore, given their ability to modulate pathways related to survival, the introduction or knock-down/out of a miRNA may be an avenue for the development of therapeutics.

In this current study, we identified numerous genes and miRNAs, many of which were novel to prion pathobiology, that have altered expression levels in the microdissected CA1 neurons from infected mice. Uniquely, we found that transcript expression followed a temporally distinct pattern revealing the disease to be a dynamic process even at pre-clinical time periods, a characteristic not captured in a whole tissue analysis. More specifically, we identified a bi-phasic molecular response where the presence of a neuronal protective mechanism was up-regulated during early prion disease and this protection was subsequently diminished at late stages of infection, in line with the clinical manifestation.

## Materials and Methods

### Ethics statement

All procedures involving live animals were approved by the Canadian Science Centre for Human and Animal Health - Animal Care Committee (CSCHAH-ACC) or the University of British Columbia Animal Care Committee according to the guidelines set by the Canadian Council on Animal Care. All protocols were designed to minimize animal discomfort. The approval identifications for this study were animal use document (AUD) #H-08-009 and AUD #H-11-020.

### Animal time course and sample collection

CD1 mice between 4 and 6 weeks of age were inoculated intraperitoneally with the Rocky Mountain Laboratory (RML) strain of scrapie using 200 µl of 1% brain homogenate in PBS from either clinically ill or normal control mice. Animals were sacrificed at intervals following inoculation and at the onset of clinical symptoms. Brains were collected at 6 time points [40, 70, 90, 110, 130 and terminal 153–161 days post infection (DPI)] and processed accordingly for either microdissection or pathological analysis. The terminal time point henceforth is designated as ‘end-point’, or EP, in the manuscript. Clinical signs used to delineate EP were kyphosis, dull ruffled coat, weight loss of 20% or more and ataxia. Samples designated for microdissection were covered in optimal cutting temperature (OCT) medium (Sakura Finetek), flash frozen using a dry ice/methanol mixture and stored at −80°C until processing, while samples planned for pathology were kept in formalin prior to tissue processing and embedding.

### Tissue processing and laser capture microdissection

Brain samples frozen in OCT were cryo-sectioned into 8 µm thick coronal sections containing the CA1 hippocampal regions, placed on polyethylene-napthalate (PEN) membrane slides (Life Technologies Inc.) and stored at −80°C for less than 4 weeks before processing. The staining and preparation of sections for the laser capture microdissection (LCM) procedure was done using the LCM staining kit (Ambion) following manufacturer's recommendations. The microdissection of the CA1 hippocampal regions were done using the Veritas LCM instrument (Arcturus) where the laser power was set between 50–100 mW and each capture was done with 2 or fewer laser pulses. Overall, about 700–1500 neuronal CA1 cells were captured per cap by combining multiple hippocampal serial sections from each animal.

### RNA extraction and quality assessment

Total RNA was isolated using the RNAqueous –Micro Kit (Life Technologies Inc.) following the manufacturer's instructions. RNA concentration and quality was assessed for each sample, in duplicate, via the 2100 Bioanalyzer RNA 6000 Pico Kit (Agilent Technologies Inc.) using the protocol provided by the manufacturer. Samples with less than a RIN value of 5.9 were not used for downstream applications (**Figure S1**). We used the same sample to assess global mRNA and miRNA expression profiles as described below.

### Whole-genome transcription profiling and analysis

To determine gene expression changes, RNA from 4–6 infected and 4–6 control mice were assayed on the Agilent whole mouse genome 4×44K arrays (Agilent Technologies Inc.). Sections of microdissected neurons that were taken from each individual mouse were pooled; this was considered to constitute a biological replicate for subsequent analyses. Due to the low abundance of total RNA isolated from each mouse, two rounds of amplification from 2 ng of total RNA was performed using the Amino Allyl MessageAmp II aRNA Amplification Kit (Life Technologies Inc.) following the manufacturer's protocol. Amplified RNA samples were labeled using either Alexa Fluor 555 (Life Technologies Inc.) or Alexa Fluor 647 (Life Technologies Inc.) as described in the Amino Allyl MessageAmp II aRNA Amplification Kit. Two-colour competitive hybridizations were performed by randomly mixing one control with one infected sample for each time point and hybridized against the same array. Dye swap experiments were executed to remove potential dye bias, resulting in a total of 8 arrays per time point. The hybridization, wash and scanning protocols were followed as described for the two-color microarray-based gene expression analysis (Agilent Technologies Inc.) according to the manufacturer's recommendations. The conversion of the raw image files to data files and subsequent quality control (QC) assessment was performed using the Feature Extraction Software versions 9.1 to 10.5.1.1 (Agilent Technologies Inc.) and only arrays that passed the QC were further considered in the analysis. This resulted in at least 4 array data sets to be compared for all time points (Gene Expression Omnibus # GSE34530).

For Gene Ontology (GO) designation, we filtered out data by selecting genes as ‘present’ based on microarray probe signal intensities above a conservative detection threshold level of 100 units. A high threshold level was chosen due to the variation inherent when using very small amounts of RNA as starting material. This variation particularly affected the detection of low abundance transcripts resulting in the prediction of inaccurate ratios for many genes. The value of 100 units was chosen arbitrarily to remove some of this variability within the data. Significance was assigned to genes exhibiting at least a 2.5-fold change in expression over mock-infected mice and having a false discovery rate (FDR) calculated to be lower than 1%. We performed an initial comparison of enriched gene ontology designations within these lists with respect to prion disease progression using the program ToppCluster [27]. Lists from each time point were submitted to ToppCluster and using a p-value cutoff of 0.05 and the Bonferroni correction method, we determined the most significantly altered “Gene Ontologies” as well as associated “Mouse Phenotypes”. Up-regulated and down-regulated genes are portrayed separately as we noticed that the gene ontology annotations were largely distinct. Networks containing these enriched terms were then constructed to allow visualization of the functional relationships that accompanied disease progression. The ‘Abstracted’ network option in ToppCluster was used for this purpose, resulting in a matrix file compatible with Cytoscape [28], [29]. The network graphics were generated using the Spring Embedded Layout function and significance was based on edge weights. Hierarchical cluster plots were produced using GeneMathsXT (www.applied-maths.com) employing the cosine correction and WPGMC (median linkage) measure.

### TLDA microRNA real-time assay and analysis

To assess the differential miRNA expression profiles throughout the time course study, TaqMan low density arrays (TLDA) for Rodent card A (Life Technologies Inc.) were used to profile 335 unique mouse miRNAs. Pre-amplification of these cDNA reactions was performed as per manufacturer's recommendations for low RNA input, which has previously been shown not to affect the miRNA expression profiles [30]–[33]. Briefly, 1 ng of total RNA from each sample was used for the reverse transcription (RT) reaction along with megaplex primer pools for Card A. The RT reactions were then mixed with the TaqMan Universal PCR Master Mix (2×), No AmpErase UNG (Life Technologies Inc.) and loaded onto each TLDA. Overall, 2 scrapie infected and 2 control mouse samples were separately run per TLDA card. All real-time PCR assays, including mRNA and miRNA validations, were run on the TaqMan 7900HT Thermocycler with Sequence Detection System (SDS) software version 2.3 and analyzed by automatically calculating the Ct values via the RQ Manager version 1.2 (Life Technologies Inc.).

TLDA analysis was preceded by an initial inspection of each amplification curve. We considered curves that did not exhibit smooth amplification characteristics as background noise and Ct values for such curves were removed from further analysis. The choice of most appropriate normalization controls and the delta Ct calculations were performed using the real-time StatMiner software version 4.2 (Intergromics). The normalization control for Card A was chosen using a three step procedure. Initially, we narrowed down our list of potential controls based on the smallest Ct range found across all detectors during our time course study of which both snoRNA-135 and snoRNA-202 had a Ct range of 1.8. By comparing U6 (a commonly used control), snoRNA-135 and snoRNA-202 using 3 available stability scoring methods from StatMiner (Normfinder, Genorm and minimum variance median), we found that snoRNA-135 was the most stable endogenous control in our experiment (**Figure S2A**). Lastly, we considered the abundance of each endogenous control in the system. The limited total RNA input that was used for the qRT-PCR assays further diluted the signal of many miRNAs we were detecting. Hence, we also decided to consider the kinetic differences between low abundance and high abundance miRNAs in the assay when choosing our endogenous control as we expected highly abundant small RNAs to behave differently in the assay than the less abundant miRNAs. Considering that the abundance of many of our assayed miRNAs were exhibiting medium to low expression levels, snoRNA-135 was again the best candidate to fit this criteria (**Figure S2B**). Therefore, the Ct values of snoRNA-135 for each card were used to normalize the signal from each probe (delta Ct). Fold changes were calculated by the 2^−(delta delta Ct)^ method [34] with standard error and Student's t-test statistics assessing significance. Due to the variability of the samples collected, true significance was represented by p-value≤0.1.

One-dimensional hierarchical clustering was performed on probes resulting in fold changes for at least 6 individual arrays (representing 3 different time points). This was done via GeneMathsXT using the Average Linkage (UPGMA) method and the Euclidean distance (with variance) measure.

### Quantitative RT-PCR and analysis for mRNA gene expression

We further assessed the expression profiles of 30 genes using quantitative real-time PCR (qRT-PCR). A total of 2 ng of total RNA from 4 infected and 4 control mouse samples for each time point were reverse transcribed separately using the High Capacity cDNA reverse transcription kit (Life Technologies Inc.). cDNA was purified using the ChargeSwitch PCR Clean-Up Kit (Life Technologies Inc.) as per manufacturer's recommendations. Concentration and rough quality was assessed using the Nanodrop ND-1000 Spectrophotometer (Thermo Scientific). Reverse transcribed DNA samples were pooled based on time point and treatment and a total of 50 ng of cDNA was used for each individual real-time reaction, performed in triplicate. For the real-time PCR reaction we used the TaqMan fast universal PCR master mix (2×), no AmpErase UNG (Life Technologies Inc.) following the fast run specifications recommended by the manufacturer. GAPDH was used as the endogenous control and fold changes were calculated by the 2^−(delta delta Ct)^ method. Standard error and Student's t-test statistics were calculated to determine significance for which a p-value≤0.05 was chosen as the cut-off.

### MiRNA validation using qRT-PCR

We analyzed the expression levels of 7 miRNAs (miR-16-5p, miR-26a-5p, miR-29a-3p, miR-140-5p, miR-132-3p, miR-146a-5p and miR-124a-3p) using a multiplex qRT-PCR approach. Overall, samples from 3–4 mice were pooled per treatment for each time point and 1 ng of the total RNA was used for the multiplex qRT-PCR reaction using the TaqMan Universal PCR Master Mix (2×), no AmpErase UNG (Life Technologies Inc.) as described by the manufacturer. To save on the limited amount of material available from each LCM preparation, we performed a multiplex qRT-PCR. More specifically, each cDNA primer was diluted ¼ to make the final primer stock and 4 µl of this stock was used in place of the 5× TaqMan miRNA RT primer. Real-time assays were run in triplicate, and Ct values for each probe were normalized to the snoRNA-135 control. The analysis was done as described for the mRNA qRT-PCR.

### Immunohistochemistry

We followed the same immunohistochemistry protocol to stain for IBA1, CREB and phospho-CREB (pCREB). For IBA1, we used rabbit anti-IBA1 antibody (Wako Pure Chemical Industries, Ltd.) at a 1∶1500 dilution in EnVision FLEX antibody diluent (Dako). Total CREB was detected using rabbit anti-CREB (48H2) antibody (Cell Signalling Technology) at 1∶2000 dilution in SignalStain Antibody Diluent (Cell Signaling Technology) while pCREB was detected using the rabbit anti-pCREB antibody (Millipore) at a 1∶800 dilution in EnVision FLEX antibody diluent.

Each brain sample was fixed in 10% neutral buffered formalin and paraffin-embedded from which 5 µm thick coronal serial sections containing the hippocampal region were produced. Sections were, baked overnight at 37°C, deparaffinized and hydrated. Endogenous enzyme activity was blocked by immersing the sections in 2.5% hydrogen peroxide (Fisher Scientific) with 5% ethanol for 10 minutes at 37°C. Subsequent antigen retrieval was done by placing the slides in 10 mM sodium citrate buffer (pH 6.0) and incubating at 121°C for 10 minutes followed by 30 minutes cool down at room temperature. A total of 100 µl of the specific primary antibody was added to the sections and incubated overnight at 4°C. Antibody signal was detected using the Klear Mouse HRP-Polymer DAB Detection System (Golden Bridge International GBI, Inc.) following the manufacturer's recommendation Sections were counterstained with hematoxylin for 20 seconds, dehydrated, cleared and mounted using Paramount (Fisher Scientific).

### Staining for PrP^Res^ deposits

Infectious PrP^Sc^ deposits were identified by using rabbit prion monoclonal antibody (EP1802Y) (Abcam) at a 1∶7000 dilution in EnVision FLEX antibody diluent. Briefly, we executed the same preliminary procedure as described for the immunohistochemistry section, including the antigen retrieval and blocking endogenous enzyme activity. At which point the staining intensity was enhanced by incubating section in 80% formic acid for 10 minutes at room temperature. Slides were rinsed in tap water for 10 minutes and washed twice for 2 minutes each in TBS/Tween (TBS/T). Slides were then treated with 4 M guanidine thiocyanate at 4°C for 2 hours, washed with TBS/T twice for 2 minutes each and treated with diluted rabbit anti-PrP at 4°C overnight. Stain detection was performed as described above using the Klear Mouse HRP-Polymer DAB Detection System. This was followed by counterstaining with hematoxylin, clearing and mounting as described above.

### Immunofluorescence

We used a double immunofluorescence staining procedure to detect astrocytes and nuclei in our sections. Briefly, we executed the same preliminary procedure as described for the immunohistochemistry sections, including the antigen retrieval step, at which point sections were blocked in 1∶20 normal goat serum (Cedarlane) that was diluted in EnVision FLEX antibody diluent for 1 hour at room temperature. The slides were incubated with rabbit anti-GFAP antibody (Abcam) at a dilution of 1∶500 in EnVision FLEX at 4°C overnight. Sections were rinsed twice with TBS/T and incubated for 1 hour at room temperature with Alexa Fluor 594 goat anti-rabbit IgG secondary antibody (Life Technologies Inc.) diluted to 1∶1000 in EnVision FLEX. Slides were then rinsed twice with distilled water for 2 minutes each and DAPI (Life Technologies Inc.) was used to stain nuclei at 1∶1000 dilution for 20 minutes at room temperature. Slides were then rinsed with distilled water, dehydrated, cleared and mounted with DPX containing coverslips (Sigma).

Flouro-Jade C (Millipore) staining was employed to detect degenerating neurons. Slides were processed as described above up to the antigen retrieval step at which point they were transferred to Flouro-Jade C solution for 30 minutes. Slides were rinsed twice in distilled water for 2 minutes per rinse followed by dehydration, clearing and mounting as described above.

### Immunohistochemical image acquisition and analysis

Immunohistochemistry slides were scanned using a MIRAX MIDI scanner (Zeiss) and regions of interest were exported and analyzed with ImageJ software [35]. Briefly, images were first colour deconvolved to separate haematoxylin and DAB stains. A haematoxylin-stained nuclei channel was used to delineate the CA1 hippocampal region. Isolated nuclear regions were obtained by empirically determining a thresholded pixel intensity to generate a mask. The masked area was measured and then used to segregate the CA1 hippocampal region in the DAB-stained probe channel. Positively-stained regions were thresholded and the masked area was measured. A scoring method was required for some DAB-stained probe channels. Positive signal intensity was binned into 3 ranges by increasing threshold amounts from background to a maximal intensity. Graphing and statistics were performed using GraphPad Prism version 5 (GraphPad Software).

### In situ hybridization

Each section was deparaffinized, rehydrated and wasted twice in DEPC-PBS for 2 minutes each. Slides were fixed with 4% paraformaldehyde (PFA) for 20 minutes at room temperature and washed twice in DEPC-PBS for 5 minutes each. Permeabilization was performed by treating the slides with 10 µg/ml proteinase K (Life Technologies, Inc.) for 10 minutes at 37°C and washed twice in DEPC-PBS for 2 minutes each. Slides were fixed again using 4% PFA for 15 minutes and washed with DEPC-PBS. Pre-hybridization of slides was performed by exposing the slides to the pre-hybridization buffer (BioChain Institute, Inc.) for 4 hours at 60°C. We added 50 nM of the linearized DIG-labeled miRNA-132 LNA probe (Exiqon) in ready to use hybridization solution (BioChain Institute, Inc.) to the slides and hybridized at 55°C overnight. Slides were washed in 2×SSC (Ambion) for 10 minutes at 55°C followed by a 1.5×SSC wash for 10 minutes at 55°C and twice in 0.2×SSC for 20 minutes at 37°C. Slides were then washed in TBS/T twice and incubated in 1× blocking solution (BioChain Institute, Inc) for 1 hour at room temperature. For visualization, we incubated the slides with 1∶500 AP-conjugates anti-digoxigenin antibody in block solution at 4°C overnight. The slides were then exposed to alkaline phosphatase buffer twice for 5 minutes each at room temperature. Slides were incubated with NBT and BCIP for 4 hours at room temperature, rinsed with distilled water and counterstained with Nuclear Fast Red (Vector Laboratories, Inc.). These slides were mounted using aquatic buffer.

### Bioinformatic miRNA target prediction

TargetScan version 5.2 (June 2011) was used to predict mouse specific miRNA targets. We chose all the conserved target gene lists for each of the 6 miRNAs along with miR-124a-3p target lists for further analysis. We compared all the predicted target genes for each miRNA using ToppFun module from the ToppGene Suite software (http://toppgene.cchmc.org/) [36]. ToppFun detects enrichment of genes for various features from which we chose to focus on Gene Ontology (GO) molecular function, biological processes and cellular component features. For each analysis, we used the Bonferroni correction and a p-value of 0.05 as a cut-off.

## Results

### The cellular architecture comprising the LCM sampled CA1 region is changing during prion disease progression

Mice were intraperitoneally (IP) inoculated with either the RML strain of mouse-adapted scrapie, or mock-infected, and animals were sacrificed at six time points spanning very early to terminal disease [40, 70, 90, 110, 130 and terminal 153–161 days post infection (DPI)]. Densely packed pyramidal cells from CA1 hippocampal tissue were isolated from 8 µm sections using the LCM. Total RNA was extracted and the relative global transciptome expression levels between infected and control mice was determined at each time point (**Figure S3**). Agilent whole mouse genome microarrays comprising 44,000 probes were used to detect mRNA expression levels. In total, we found 2,580 genes to be differentially expressed by greater than 2.5 fold during at least one time point with false discovery rates (FDR) of <1% (**Table S1**).

Prior to performing an in depth analysis of time-dependent transcriptomic changes, we assessed the relative abundances of neurons, astrocytes and microglia in the sampled CA1 region from both infected and uninfected mice. This served as a baseline for the relative proportions of these cells in each sample, and to qualitatively measure the extent of infiltration and activation of astrocytes and microglia within this region over time. We initially inspected the expression levels of numerous cell type-specific marker genes which were further correlated with quantitative immunohistochemistry. The expression of the house-keeping gene GAPDH, like the majority of genes assayed on the array, remained relatively stable throughout the time-course for both control and infected mice (**Figure S4**); therefore, GAPDH was used as a control for internal variability.

**Figure 1:**
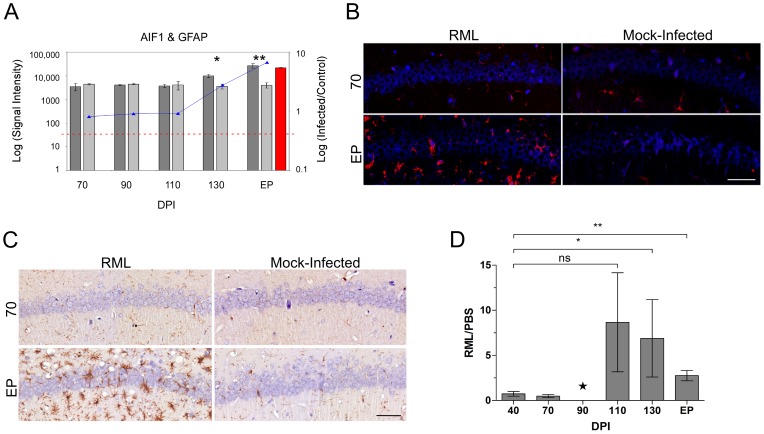
The distribution of resident immune cells in the CA1 hippocampal region of both control and prion infected mice. (**A**) The expression levels of microglia (AIF1) and astrocyte (GFAP) cell markers determined by microarrays in both control and infected samples. The bars represent log signal intensity values where dark gray bars indicate infected samples while light gray bars represent control levels of AIF1. Statistical significance was calculated by the Student's t-test where * reflects a p-value≤0.05 and ** for p-value≤0.01. The AIF1 log fold change is plotted as a line graph. The red bar reflects GFAP signal in prion infected sample as we did not detect any signal in control samples. The horizontal dotted red line represents the signal intensity threshold set at 100. (**B**) Immunoflourescence images for both control and infected mice at 70 and EP times post infection showing the presence of GFAP (red) within the CA1 nuclear cell layer (blue). Scale bar reflects a 50 µm region. (**C**) Representative immunohistochemistry images of microglial CA1 regions of control and prion infected samples. Images represent 70 and end point (EP) DPIs showing microglial cells stained brown contrasted against the blue nuclear stain. Scale bar reflects a 50 µm region. (**D**) The quantified presence of microglia in control and prion infected samples during the course of disease. The star for the DPI 90 time point indicates insufficient replicates for accurate statistical analysis. Statistical significance was calculated by the Student's t-test; ns means no significance; * for a p-value of ≤0.05 and ** for a p-value of ≤0.0005.

Astrocyte abundance in the LCM generated samples was assessed using glial fibrillary acidic protein (GFAP) expression levels. We found that the GFAP spot intensities were below the cut-off level for detection in all of the CA1 samples dissected from control mice. In turn, a detectable level of GFAP was only evident in RNA samples from RML-infected mice sacrificed at terminal stages of disease, or end point (EP) (**Figure 1A**). Immunohistochemical staining of GFAP reciprocated these findings; stained cell bodies of astrocytes were normally present in extremely low numbers in the CA1 microdissected region. Significant infiltration of astrocytes to this area was apparent during clinical disease (**Figure 1B**). We next determined the presence of microglial marker genes within the CA1 hippocampal region. The expression of the microglial marker genes allograft inflammatory factor (AIF1), otherwise known as IBA1, and also F4/80 a marker of murine macrophage populations (data not shown), were detected in both control and infected mice at all time points. Nevertheless, a statistically significant increase in the abundance of these transcripts in infected animals was only evident at 130 days post infection and EP (**Figure 1A**). Immunohistochemistry using an antibody directed against IBA1 mirrored this data (**Figure 1C and D**). Microglial staining was evident in all samples and was associated with cells interlaced amongst the densely packed CA1 neurons. Staining was more intense beginning at 110 DPI and the total numbers of cell bodies stained brown was approximately 3-fold higher in infected mice versus control by 130 DPI and EP, thus, mimicking the microarray expression profile.

**Figure 2:**
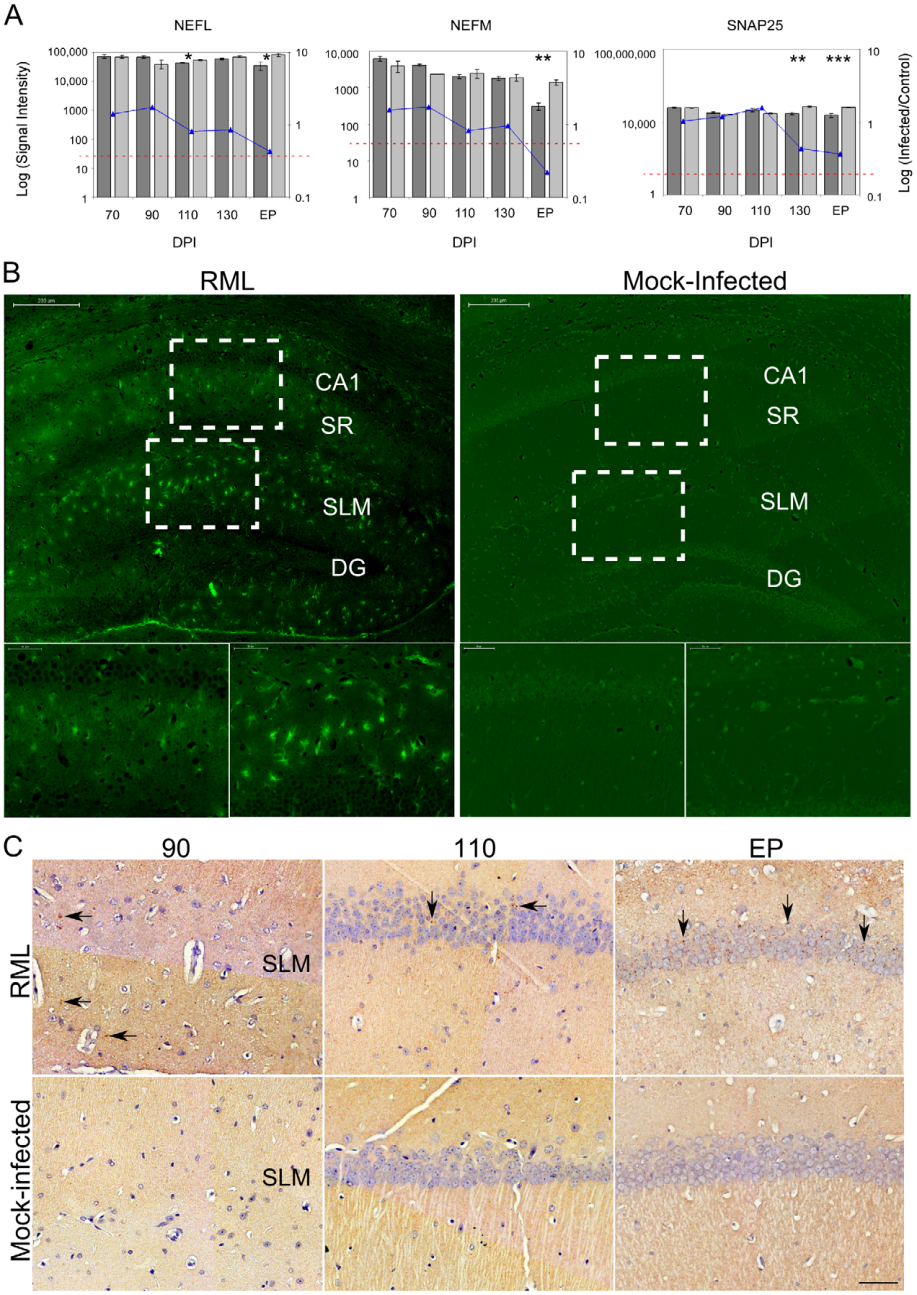
The overall neuronal architecture of the CA1 hippocampal region and the localization of PrP^Res^ deposits during prion disease. (**A**) The gene expression levels of 3 neuronal markers (NEFL, NEM and SNAP25) in control and prion infected samples. The bars represent log signal intensity values where dark gray bars indicate infected samples while light gray bars represent control levels for each specified gene. Significance was calculated by the Student's t-test where * represents a p-value≤0.05; ** reflects a p-value≤0.01 and *** stands for a p-value≤0.001. The log fold change is plotted as a line graph for each gene. The horizontal dotted red line represents the signal intensity threshold set at 100. (**B**) Neuronal degeneration was identified by staining hippocampal regions with FlouroJadeC at late (EP DPI) stages of prion disease. The large images represent the hippocampal region (scale bar represents 200 µm) while the outlined rectangles represent the regions that are further magnified (scale bar is 50 µm). CA1, cornu ammonis 1; DG, dentate gyrase; SR, stratum radiatum; SLM, stratum lacunosum-moleculare. (**C**) The localization of PrP^Res^ deposits in the hippocampal region at early (90 and 110 DPI) and late stages of disease (EP). Brown staining represents PrP presence while blue shows nuclear staining. Arrows indicate some of the PrP^Res^ deposits that were identified throughout disease progression.

Gene markers for neuronal cells such as the neurofilament medium and light polypeptides (NEFM and NEFL) plus synaptosomal-associated protein 25 (SNAP25) were confirmed as highly enriched in the sampled tissue, as was expected given the stringent dissections (**Figure 2A**). We observed a reduction in the signal intensities of these marker genes only at clinical stages of disease in comparison to age-matched, mock-infected controls. Statistically significant reductions of these genes were evident at 130 DPI and EP, however, the decline in expression was relatively small compared to the total signal suggesting that a substantial proportion of neuronal cell bodies were present at EP. We noticed that the neuronal genes showing the most significant decreases in expression, and that were clearly detectable by 130 DPI, coded for proteins that are associated with synaptosomal fractions, such as SNAP25. We further visualized degenerating neurons using Flouro-Jade C, a stain that associates with all neurons undergoing degeneration, regardless of specific insult or mechanism of cell death [37]. Flouro-Jade C staining revealed relatively few degenerating neurons in the sampled CA1 pyramidal layer, even at the end stage of disease (**Figure 2B**), which agrees with the microarray findings. This mirrors previous studies showing that cell death in this region occurs in the final stages of disease [2], [38]. However, Flouro-Jade C staining did reveal that interneurons in adjacent layers of the hippocampus, located within the *stratum radiatum* (SR) or *stratum lacunosum-moleculare* (SLM), exhibited higher proportions of degenerating neurons much earlier in the disease process; by 110 DPI with scarce staining even detectable at 90 DPI (data not shown). These results would suggest that some neurons are more susceptible to damage associated with prion replication and that CA1 pyramidal neurons appear to be particularly robust. However, since the CA1 neurons are intricately associated with adjacent interneurons through synaptic connections, the observed neuronal degeneration in these interneurons would most likely impact the responses of the neurons residing within the CA1 hippocampal region.

The accumulation of PrP^Res^ in the CA1 hippocampal region over time was also confirmed by immunohistochemical staining. Occasional PrP^Res^ deposits associated with CA1 neuronal projections were first detectable at 90 DPI in the *stratum lacunosum-moleculare* with more consistent buildup evident from 110 DPI until EP (**Figure 2C**). Although minimal PrP^Res^ presence was directly associated with the cell bodies of CA1 pyramidal neurons during early stages of disease, the dendrites of these neurons pass through the SR and/or SLM regions. Replication here could well contribute to a prion replication-associated response that is reflected in the alteration of mRNA expression within the cell bodies of these neurons.

Overall, we found that the temporal expression changes of cell-specific gene markers from microarrays correlated well with the different cell types and neuronal degeneration observed by immunohistochemical and Flouro-Jade C staining. Given that extensive infiltration of astrocytes and amplification of microglia are only evident at late stages of infection, we believe that transcriptional alterations observed prior to and including 110 days post infection most likely reflect either neuronal events or very early transcriptional changes in microglia precipitating their activation and accumulation.

### Transcriptomic analysis reveals temporal alterations in mRNA expression congruent with prion disease progression

**Figure 3:**
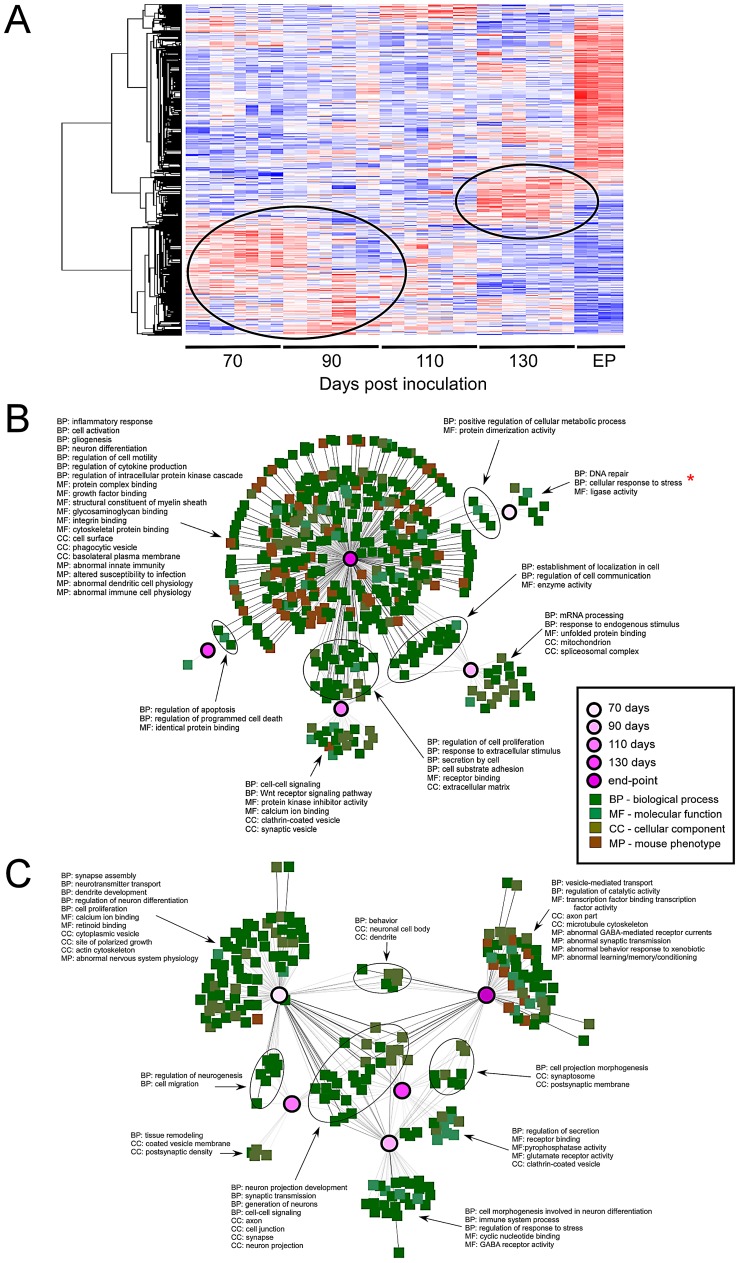
A dynamic temporal gene expression profile identified between prion infected and control samples. (**A**) Hierarchical clustering of 1026 genes that showed significant differential expression across time between the prion-infected and mock-infected control groups (ctrl; FDR<0.001 and at least two fold change). Red indicates increased and blue indicates decreased expression levels relative to the mean (white). The two circles highlight the “waves” of genes that are temporally deregulated during prion disease. (**B**) Gene ontology networks for up-regulated genes are grouped according to the indicated time points analyzed. Select ontologies are highlighted of which majority reflect immune-regulated genes at late stages of disease. The gene ontology involved in the stress response is highlighted by a red * that occurs at 70 DPI. (**C**) Gene ontology networks for down-regulated genes are grouped according to the indicated time points analyzed. Select ontologies are highlighted for which majority reflect neuronal-specific gene function.

The most striking finding in our analysis of the microarray data was that alterations within the LCM sampled region followed a strictly temporal pattern (**Figure 3A**). Differential expressions at 70, 90 and 110 DPI (pre-clinical) are quite dissimilar to those changes that are induced at 130 DPI or EP. The timing of the major sequential changes in CA1 neurons, beginning by 70 DPI, correlates well with detectable prion replication in the hippocampus and suggests that the transcriptome is temporally altered as a direct molecular response to the progressive replication of prions. However, we cannot rule out earlier changes as we did not perform exhaustive analysis prior to this time. Interestingly, between 110 and 130 DPIs, the transcriptional response in the CA1 pyramidal region transitions in two ways; firstly, the initial wave of differential expression either returns to basal levels or follows an inverted pattern, where for example, previously up-regulated genes become down-regulated. Secondly, smaller waves of novel gene expression changes become apparent by 130 DPI, with very strong differential expression detectable at EP.

Our first step in the analysis was to compare those gene-ontology functional designations that were enriched with respect to days post inoculation in order to identify some of the annotated molecular events that accompany prion disease-progression. Briefly, lists of genes that exhibited at least a 2.5-fold change in expression over mock-infected mice with an FDR lower than 1% were compared between each time-point. An initial finding showed the significant delineation between up-regulated and down-regulated genes within ontologically classified genes across the data set. We found that we were able to improve the discrimination of significant ontology's amongst these genes by analyzing these two groups separately. Networks generated using the program ToppCluster are displayed in **Figure 3B and C** where some of the major annotations in terms of gene functional groups are highlighted (detailed annotation of the networks is provided in (**Figure S5**).

Up-regulated genes fell into a small number of statistically significant ontological groupings at the early time-points; whilst an overwhelming abundance of these enriched gene groups were found only at the clinical end-point of infection. Although a representative group of ontologies are highlighted in **Figure 3B**, approximately 85% of the ontological and phenotypic gene categories at clinical end-point reflect the development and function of immune-related cells. The appearance of these genes correlate well with the infiltration of activated GFAP expressing astrocytes plus increased numbers and activation of microglia, and contains the majority of genes previously reported to be differentially expressed in prion disease [5], [7], [13]. A hierarchical cluster plot summarizes the expression pattern of a group of immune-related genes that have been previously identified to be over-expressed in prion disease as well as other neurodegenerative conditions (**Figure S6**). Interestingly, although the majority of these genes are only strongly expressed during clinical disease, a minority of genes with immunological functions begin a sequential over-expression at 110 DPI such as ICAM1, VCAM1, FYB or S100A4, while some genes show a down-regulated trend such as IRAK1 or CDC42. The adhesion molecules ICAM1 and VCAM1 are typically expressed on endothelial cells and cells of the immune system, including microglia and astrocytes [39]. In this case, microdissection clearly allows us to track the temporal expression of these adhesion molecules well before the detection of immune activation markers such as cytokines and chemokines. These genes are likely expressed in the resting microglia that we found to be closely associated with neurons in this region. They appear to be up-regulated within the CA1 pyramidal region prior to the infiltration of activated immune cells and may be involved in either the activation of phagocytosis and prion-aggregate removal, or in the recruitment of and/or induction of inflammatory pathways in microglia or astrocytes [40], [41]. These particular genes may be useful genetic indicators of very early cellular response(s) to damage.

**Figure 4:**
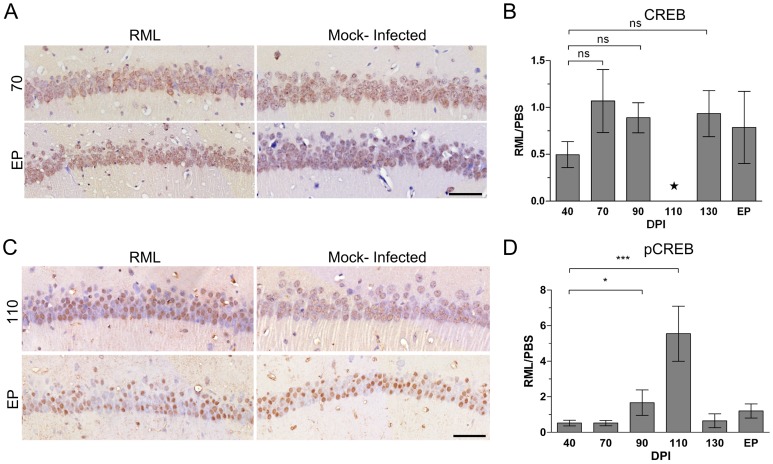
Immunohistochemical detection of CREB in CA1 hippocampal neurons reveals an up-regulation of the phosphorylated form of the protein during early prion disease. (**A**) Immunohistochemical representation of total CREB levels at 70 and EP post infection in either RML or mock-infected animals. Brown staining represents CREB levels against the blue stained nuclei. Scale bar represents 50 µm. (**B**) Quantitative assessment of CREB levels by comparing RML infected and control CA1 hippocampal samples throughout prion disease progression. The star represents insufficient replicates for statistical purposes. (**C**) Representative immunohistochemistry images of pCREB at pre-clinical (110 DPI) and clinical (EP DPI) prion disease. Brown staining represents pCREB between RML infected and control samples. Scale bar represents a 50 µm region. (**D**) Quantitative assessment of all sections for pCREB were taken at each time point with Student's t-test statistic; * for a p-value of ≤0.05 and *** for a p-value of ≤0.0001.

Up-regulated genes enriched at pre-clinical stages fell into much fewer significant groups of functionally related transcripts. However, the significant groups we identified were found to include genes expressed in response to various stressors as early as 70 days post-infection (**Figure 3B**). A significant number of these transcripts code for genes known to be regulated by the transcription factor cyclicAMP responsive element binding protein 1 (CREB1). In fact, the IPA analysis tool identified CREB as the top upstream regulator of these genes with a p-value of 10^−15^ (data not shown). These included a number of highly inducible transcription factors such as FOS, EGR2, EGR4 and NR4A1. No significant change in CREB mRNA was identified by microarrays, which was confirmed by qRT-PCR (data not shown) and immunohistochemical staining of total CREB in the CA1 region (**Figure 4A and B**). However, it is the phosphorylation of CREB at Ser133 that leads to the recruitment of transcriptional coactivators required for the expression of a large number of genes (for review, see [42]). Staining with an antibody to detect CREB phosphorylated at Ser133 showed that pCREB significantly increased in CA1 sections from infected mice only during pre-clinial disease, as compared to similar sections from uninfected mice (**Figure 4C and D**). In addition, a number of kinases such as TRIB1 and CAMK2D plus extracellular matrix glycoproteins such as LAMC2 were also strongly up-regulated. We chose 3 of these, EGR2, CAMK2D and TRIB1, to validate by qRT-PCR. All three of these genes were similarly up-regulated in LCM samples at pre-clinical disease taken from a second group of infected mice inoculated in an independent experiment (**Table 1**).

**Table 1:** The list of genes we further investigated using qRT-PCR.

Gene ID	Pre-Clinical	Clinical	Implicated Function	References
CAMK1	++ (++)	(++)	Ca^2+^/calmodulin-dependent protein kinase that is a positive transducer of growth cone mobility and axon outgrowth	[145]
DAB2	++ (+)	(++)	negative regulator of neurite outgrowth	[123]
TRIB1	++ (+++)		negative regulator of retinoic acid receptors and is involved in modulating signaling of MAP kinase cascades	[96], [147], [148]
MCL1	++(+)		anti-apoptotic protein that is responsive to excitotoxic insults in the central nervous system	[149]
DOCK1	+++ (−)	(+++)	promotes spine morphogenesis in hippocampal neurons	[119]
ProSAPiP1	+		postsynaptic scaffolding protein the helps regulate synaptic development and plasticity	[150], [151]
SLC6A13	++ (+++)	++ (+++)	GABA transport protein (GAT-2) which is primarily localized to extrasynaptic areas and may regulate extrasynaptic GABA levels	[152]
EGR2	++ (+++)	++	immediate early gene induced by BDNF and is important for terminal dendrite differentiation	[153]
RASGRF2	+++ (++)	−− (−−)	A calcium sensor that is member of calcium/calmodulin-regulated guanine-nucleotide exchange factors that activates Ras GTPases	[104]
WASF2	++ (+)	(++)	regulates dendite spine formation	[154]
HOMER1	+ (++)	(−)	dendritic scaffold protein that regulates group 1 metabotrophic glutamate receptor (mGluR) function and has roles in growth cone turning	[105]
GRIN2B	−− (−−)	(−−−)	NMDAR subunit that promotes neuronal cell death and survival	[92], [93], [155]
MYO5A	+ (+)	− (−−−)	modulator of synaptic plasticity by helping release neuropeptides via exocytosis recycling of neurotransmitter receptors via endosomes	[120]
MYO6	++ (+)	+ (+)	plays a role in clathrin-mediated endocytosis of AMPARs and BDNF-mediated neurotransmission	[121], [156]
CAMK2D	+++ (++)	++	Ca^2+^/calmodulin-dependent protein kinase that may function in long term potentiation and neurotransmitter release	[157]
PPP1R15	+++ (++)	(−−)	dephosphorylates eIF2α-P	[155]
RNASEN	(−)	−	core nuclease that initiates microRNA processing in the nucleus	[158]
HDAC9	− (−−)	(−−)	histone deacetylase 9 enzyme inhibits dendrite growth	[115]
RCAN1	− (−)		regulator of calcineurin 1 mediates apoptosis via caspase-3 activation	[159]
MEF2D	−		transcription factor that positively regulates hippocampal synaptic function and promotes neuronal survival	[160], [161]
PIP5K3	− (−)	+	lipid kinase that regulates endosomal and lysosomal degradation of calcium channels, protecting neurons from excitotoxicity	[162]
BAD	− (+)	+ (+)	pro-apoptotic Bcl-2 family member	[163]
CREB1		+	transcription factor involved in neuronal pro-survival mechanisms	[164]–[166]
PPP3CA	−	+	calcineurin protein mediates neurotoxicity in Alzheimer's disease and decreases synaptic plasticity	[167], [168]
RAC1	− (++)	+	belongs to the Rho family of GTPases and is required for proliferation and survival of progenitor cells in subventricular zone	[169], [170]
CAMK4	(−−)	+/− (−−)	Ca^2+^/calmodulin-dependent protein kinase that stimulates activation of CREB and pomotes neuronal survival	[171], [172]
ADCY7		+++ (+++)	adenylate cyclase 7 which catalizes the formation of cyclic AMP; upstream signaling molecule for CREB activation	[173], [174]
CACNA1C	(+)	(++)	calcium channel that functions in NMDAR-independent synaptic plasticity and memory	[175]
CAP1	(−)	(−)	regulates neurite outgrowth by rearranging F-actin	[176]
GPM6B	(−)	(−−)	regulates seratonin uptake by modulating the trafficking of seratonin transporters	[177]

Student's t-test statistic was employed such that a deregulation in fold change between infected and control samples showed at least a p-value≤0.1. The+or−represents a 1–2 fold change; 2.1–3.0 fold change is represented by++or−−while a ≥3.1 is annotated as +++. Blank spaces represent a lack of significant signal between infected and control samples. Relative microarray fold changes are highlighted in parenthesis. Pre-clinical time period spans up to and including 110 DPI while clinical represents 130 DPI and EP.

Networks generated using enriched terms amongst down-regulated genes exhibited considerable functional consistency between pre-clinical and clinical time-points (**Figure 3C**). Notably, the ontology annotations strongly implicate alterations in the expression of genes that are involved in neuronal projection and dendrite development from early time points onwards. This is consistent with the reduction in dendrites reported as the earliest detectable pathological change in neurons during prion disease [1], [43]. Similarly, perturbations in synaptic transmission and synapse formation are implicated throughout disease progression, from mid-way through the incubation period and beyond [2], [44], [45], coinciding with our 70 DPI time-point. The 70 and 90 days post-infection times also reveal alterations in calcium ion binding and receptor activities while later time-points (130 and EP) show additional down-regulation amongst multiple ontologies relating to vesicle formation and transport, some structural proteins such as those related to cytoskeletal physiology as well as transcripts related to behavioral changes. The reduction in expression of genes specifically involved in synapse formation and/or maintenance, such as synaptophysin (SYP), synaptotagmin (SYT1), alpha-synuclein (SNCA) and SNAP25, plus general dendrite physiology, is particularly striking at 130 DPI and EP.

Interestingly, groups of genes that have been previously related to obvious phenotypic changes in mice (shown in **Figure 3B**) were almost solely contained within the end-point sampling, correlating closely with the onset of clinical symptoms beginning around 130 DPI. These phenotypes include abnormal synaptic transmission leading to behavior, learning and memory deficits plus abnormal innate immunity; all of which are clinical symptoms of neurodegenerative conditions including prion disease.

### Transcriptional alterations during early prion disease suggest the induction of a neuroprotective program that may be initiated by synaptic NMDA receptors

**Figure 5:**
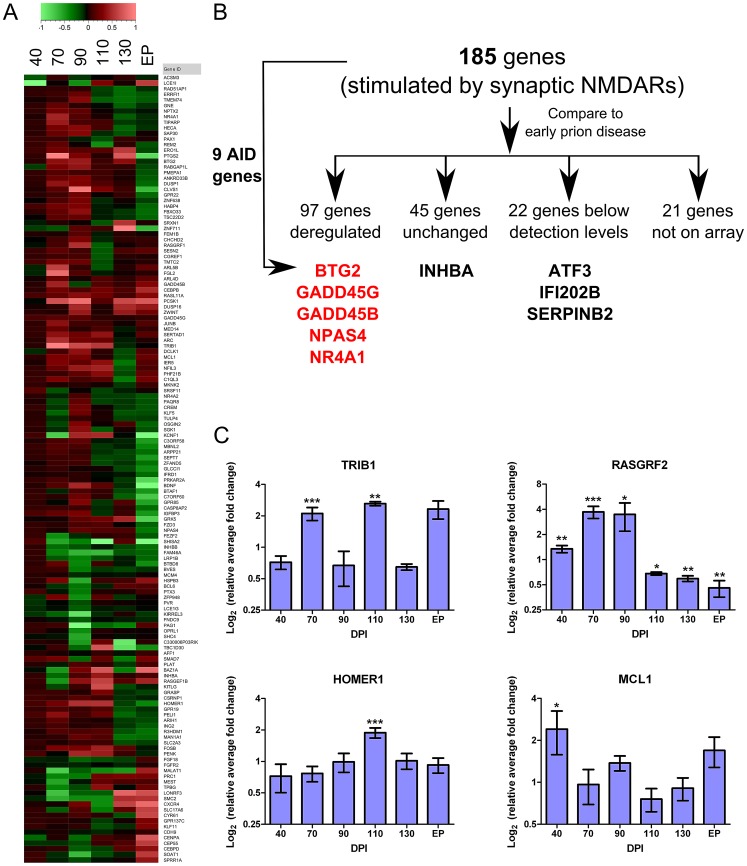
Genes deregulated during early prion disease suggest the stimulation of a neuroprotective mechanism. (**A**) A hierarchical plot of the 141 neuronal activity-regulated genes identified by Zhang and colleagues [47] that we also found deregulated during early prion disease in CA1 hippocampal regions. Legend describes the log fold change. (**B**) The detailed distribution of the genes we found deregulated in prion infection as compared to the list of 185 neuronal activity-regulated genes. From these initial 185 gene list, 9 genes were a core set of neuroprotective genes termed Activity-regulated Inhibitor of Death (AID) genes that are further highlighted in either red or black. Red represents an up-regulation while black represents either unchanged or not detected expression in prion infected samples. (**C**) Real-time PCR validation of 4 genes from the 185 gene list showing log2 ratio of relative average fold change over days post infection (DPI). Significance was calculated by the Student's t-test statistic where * was a p-value of ≤0.05; ** was a p-value of ≤0.01 and *** was a p-value of ≤0.001.

Detecting and exploring the earliest transcriptional changes that occur during pre-clinical disease (70–110 DPI) was the particular focus of this work. As previously described, a cluster of approximately 400 genes was significantly dysregulated in the pyramidal layer of the CA1 at these early time points. We performed a literature search to identify published genomic data generated from hippocampal neurons that may be associated with survival/death mechanisms. This meta-analysis proved fruitful; in particular, a comparison with data sets generated by Zhang *et al* to identify gene expression changes in hippocampal primary neurons in response to NMDA stimulation [46], [47] revealed striking similarities to our dataset. The aim of these two studies was to determine transcriptional programs triggered by the stimulation of either synaptic NMDA receptors (NMDARs) alone, or by overstimulation at extrasynaptic locations. Mounting evidence suggests that the subcellular location of these NMDARs in neurons is critical to the response that follows (for review, see [48]). Synaptic NMDAR activity appears to transduce a signal promoting a neuroprotective genetic cascade, whereas Ca^2+^ influx through extra-synaptic localized NMDARs appears to directly oppose these effects and promote cell death. In total, 185 neuronal genes were reported to be altered in expression following Ca^2+^ influx through synaptic NMDARs [46]. Validatory studies revealed that a number of these changes were able to promote neuroprotection in primary neurons. Comparison of this list with our cluster of genes altered between 70 and 110 DPI (FDR<5%) revealed 97 genes in common. Of the rest, 22 genes were below the level of detection in our study, 45 were unchanged early in disease although a number of these were altered during clinical disease, and 21 were not represented on our array (**Figure 5A and B**).

A core set of 9 genes were validated by Zhang *et al* [47] as being critical to the neuroprotective response and these were termed Activity-regulated Inhibitor of Death genes, or AIDs. Significantly, we identified 5 out of the 9 AID genes to be up-regulated (BTG2, GADD45G, GADD45B, NPAS4, NR4A1), INHBA did not appear to change in expression and the remaining 3 genes (ATF3, IFI202B and SERPINB2) were below the level of detection in our study (**Figure 5B**). We also used qRT-PCR to validate the pre-clinical over-expression of 4 of these genes previously unrecognized as playing a role in prion pathobiology (**Table 1** and **Figure 5C**); RASGRF2, TRIB1, MCL1 and HOMER1 were all confirmed to be up-regulated during the same time period post-infection in CA1 pyramidal neurons. We believe that the similar alteration of 60% of detectable genes from the Zhang study including many of the critical AID genes is highly suggestive of the induction of a similar neuroprotective program in the CA1 during early prion disease.

### Temporal changes in miRNA expression levels mirror transcriptome identified gene ontologies during the course of prion disease

**Figure 6:**
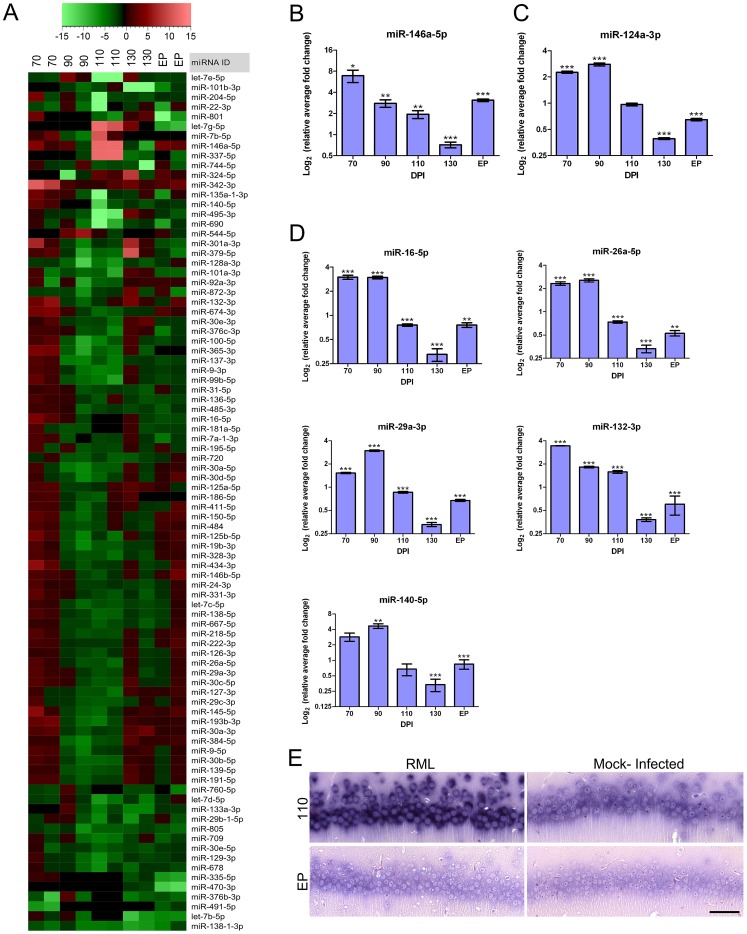
Global miRNA expression profile identified throughout prion infection further highlights a temporal dynamic in disease. (**A**) A hierarchical cluster plot showing fold changes of prion-infected as compared to control samples throughout the disease process. Legend indicates the fold change. (**B**) The validated miRNA-146a-5p expression profile during prion disease in the CA1 hippocampal region where * indicates a p-value≤0.1; ** reflects p-value≤0.05; *** represents p-value≤0.01 (n≥3). (**C**) Real-time PCR validation assay of miR-124a-3p throughout prion infection where * indicates a p-value≤0.1; ** reflects p-value≤0.05; *** represents p-value≤0.01 (n≥3). (**D**) Validation of 5 additional miRNAs using real-time PCR: miR-16-5p, miR-26a-5p, miR-29a-3p, miR-132-3p and miR-140-5p. Log_2_ of the relative average fold change is graphed against days post inoculation (DPI). Significance was determined using Student's t-test statistic where * represents a p-value≤0.1; ** represents a p-value≤0.05; *** represents a p-value of ≤0.01 (n≥3). (**E**) *In situ* hybridization of miR-132-3p in RML and control CA1 hippocampal samples at pre-clinical (110 DPI) and clinical (EP) prion disease. Dark blue/black staining shows the presence of miR-132-3p. Scale bar represents a 50 µm length.

We have previously used whole mouse brain tissue to identify miRNA expression alterations in prion infection [49]. We therefore extended this study to identify changes in miRNA expression levels in the same CA1 hippocampal RNA preparations that we used for mRNA profiling. Differential miRNA expression was determined using a multiplex qRT-PCR platform, TaqMan Low Density Arrays (TLDA). Over the course of the disease, we found 88 miRNAs that had altered expression levels for at least one time-point based on a p-value of <0.1 (**Table S2**). Analysis of the expression profiles of miRNAs from these samples revealed similar patterns of sequential expression to those exhibited by mRNAs (**Figure 6A**). From the 88 miRNAs found to be differentially expressed throughout the experimental study, 17 miRNAs were only deregulated early in the course of disease prior to 130 DPI, 57 miRNAs were up-regulated at 130 DPI and/or EP of disease while 8 miRNAs were detected only in infected samples at these time points (**Table S3**).

Although little is known about the functional roles of many miRNAs and therefore, classical bioinformatics analysis based on ontology is not very definitive; we were able to use prior knowledge to predict the roles of some of the temporally altered clusters of miRNAs. Previously, we showed that gene expression restricted to glial cells is either only detectable, or prodigiously induced, primarily in the LCM generated samples collected at EP. As this was also borne out by immunohistological staining, we hypothesized that glial-associated miRNA expression would follow a similar pattern. Specifically, we compared those 57 miRNAs deregulated or induced in the samples taken at clinical end-point with published data describing miRNA up-regulation in immune stimulated primary astrocytes [50]. Of the 57 miRNAs deregulated at these time points we found that 44, (∼77%), correspond to miRNAs that are induced in activated astrocytes (**Table S4**). By association, it is likely that the majority of these 44 miRNAs are involved in innate immune related functions within activated astrocytes and possibly activated microglia that infiltrate our dissection area during clinical disease.

We further observed that 8 of these miRNAs were either only detectable or induced during clinical disease based on TLDA results. Specifically, miR-19a-3p, miR-344-3p, miR-34b-3p and miR-497-5p were all detected only in samples from RML infected mice (**Figure S7A**). This pattern emulates the expression of astrocyte-specific genes such as GFAP. In turn miR-150-5p, miR-26b-5p and miR-410-3p were detectable in all the microdissected samples, although only substantially up-regulated at EP. By analogy with our mRNA data, we postulate that these miRNAs are either expressed at a basal level in microglia prior to their activation or alternatively, in resident neurons and are induced only by severe stress (**Figure S7B**). Although we cannot rule out the possibility of induction in damaged neurons, comparison of the relative level of expression of each of these miRNAs to a list of those expressed in mouse hippocampal neurons [51] reveals that the majority have very low abundance in neuronal cells (**Table S5**). Therefore, based on their pattern of expression and low abundance in neurons we conclude that these miRNAs are most likely induced in activated microglia.

In contrast to the other 8 miRNAs in this group we found miR-146a-5p to be up-regulated early in disease as well as at clinical end-point (**Figure 6B**). MiR-146a-5p was of interest as it has been reported to be up-regulated in brain tissue in a number of neurodegenerative conditions including prion animal models [50] as well as in human cases of Creutzfeldt-Jakob disease (CJD) and Gerstmann-Sträussler-Scheinker (GSS) [52]. Its function as an immune response regulator is well documented [53], and we have determined its induction in activated microglia [54]. The up-regulation of miR-146a-5p during the 70–110 DPI time-period, however, is suggestive to originate from CA1 pyramidal neurons although a specific role of this miRNA in neuronal cells has not been reported.

We next determined the abundance of miR-124a-3p, a miRNA that is well-known to be highly enriched in neurons [55], [56], to see if its expression level mirrored those of the neuronal-specific marker genes tested previously. We did indeed detect high levels of miR-124a-3p in both infected and uninfected mice and saw a significant reduction in miR-124a-3p abundance in the CA1 dissected samples from prion infected mice at the clinical stage of disease (**Figure 6C**). Interestingly, we noted that this miRNA was also up-regulated in both the 70 and 90 DPI samples collected from infected mice suggesting a potential disease-related function during this period.

Following confirmation that miRNA and mRNA expression profiles are to some extent analogous, our next goal was to validate the expression of the earliest miRNAs found to be deregulated; i.e. during the 70–110 DPI time-period. We had initially identified 17 miRNAs to be deregulated at this stage in disease from the TLDA assays (**Table S6**). We further confirmed the expression levels of 7 miRNAs using qRT-PCR, miR-16-5p, miR-26a-5p, miR-29a-3p, miR-132-3p, miR-140-5p, miR-124a-3p and miR-146a-5p, all of which were up-regulated in infected samples prior to 130 DPI (**Figure 6B, C and D**). The overall pattern of expression of the majority of this group was a significant up-regulation throughout pre-clinical disease followed by a drastic decrease in expression for many at 130 DPI and EP. This pattern of expression is analogous to many of the mRNAs that formed our early deregulated cluster. We also used *in situ* hybridization to confirm the expression level of miR-132-3p because the expression level of this miRNA is known to be induced by pCREB. We found that miR-132-3p appears to be significantly more abundant at pre-clinical time points in the CA1 granule neurons in infected versus uninfected cells (**Figure 6E**), in conjunction with pre-clinical pCREB expression levels.

To confirm that these miRNAs are likely enriched in neurons, we compared their relative expression to a list of miRNAs expressed at basal levels in the cell bodies of rat hippocampal neurons that was generated by Kye and colleagues [57]. As many miRNAs are highly conserved between mammals (**Table S7**) this type of interspecies comparison is applicable. Apart from miR-140-5p and miR-146a-5p, all of these early miRNAs are abundantly expressed in rat neurons (**Table 2**). Next generation sequencing of small RNAs isolated from mouse hippocampus also confirms the endogenous expression of all 7 of these miRNAs in this brain region [52]. In fact, miR-29a-3p, miR-26a-5p and miR-132-3p are particularly highly expressed in the hippocampus, being even more abundant than miR-124a-3p, such that 2.63, 1.54 and 0.43% of total miRNA reads were composed of these miRNAs, respectively (**Table 2**). Furthermore, miR-146a-5p, miR-16-5p and miR-140-5p, although lower in abundance than miR-124a-3p, were also present in mouse hippocampus in this study. Although some of these miRNAs are expressed at very low levels in neurons, miR-140-5p has been similarly detected by miRNA microarray of rat hippocampus [58] while miR-146a-5p presence has been linked to the CA1 region of the rat hippocampus in one study using *in situ* hybridization [59].

**Table 2:** The endogenous expression levels in rat hippocampus of the 6 validated miRNAs that were found to be over-expressed early in prion disease.

	Rat Hippocampus*	Mouse Hippocampus**		
miRNA ID	Ct in neuronal cell body	Total Counts (NGS data)	% of Total miRNA counts	% of miR-124a-3p counts
miR-29a-3p	20.45	388133	2.63	974.67
miR-26a-5p	14.64	227704	1.54	571.80
miR-132-3p	15.08	62864	0.43	157.86
miR-146a-5p	-	17814	0.12	44.73
miR-16-5p	17.01	12775	0.09	32.08
miR-140-5p	-	7898	0.05	19.83
miR-124a-3p	-	39822	0.27	100.00

MiR-124a-3p expression levels are shown for reference purposes. NGS refers to next generation sequencing.

* data from [57].

** data from [51].

- reflects lack of data.

### Bioinformatic prediction of miRNA function in neuronal-specific pathways

Although not enough is known about miRNA function in neurons to perform an ontological analysis as is done for mRNAs, we performed a preliminary analysis of the bioinformatically predicted targets of the 7 miRNAs confirmed to be dysregulated during early disease using TargetScan [60]–[62]. We found a total of 396 target mRNAs for at least one of the 7 miRNAs we validated that were also down-regulated between 70 and 110 DPI. To identify the biological processes that these genes are associated with, we ran an enrichment analysis based on GO terms on these genes. In total, 80 GO biological process annotations were identified (**Table S8**) of which many were associated with neuronal function. Of these, 14 processes are strictly related to neuronal functions (**Table 3**) where synaptic organization is the most represented pathway containing approximately 11% of the miRNA target genes that are both down-regulated and targeted by at least 1 of the 7 candidate miRNAs. This preliminary data suggests that further investigation into the functions of these miRNAs may help determine regulatory pathways that are affected during early pathogenesis of prion disease.

**Table 3:** The list of the 14 processes that consist of the miRNA target genes which are strictly related to neuronal function for which the p-value is less than or equal to 0.05.

Biological Process	P-value	Term in Query	Term in Genome	% represented
neurogenesis	2.31E-11	63	1015	6.21
generation of neurons	3.20E-09	57	959	5.94
neuron development	3.32E-09	48	715	6.71
neuron differentiation	2.45E-08	53	894	5.93
neuron projection development	3.48E-07	41	625	6.56
central nervous system development	4.41E-06	37	572	6.47
neuron projection morphogenesis	6.55E-05	33	523	6.31
axonogenesis	1.16E-04	31	482	6.43
regulation of nervous system development	1.57E-03	24	353	6.80
brain development	5.34E-03	25	405	6.17
tube development	8.64E-03	23	362	6.35
regulation of neurogenesis	1.12E-02	21	315	6.67
**synapse organization**	**3.79E-02**	**11**	**104**	**10.58**
forebrain development	4.36E-02	17	240	7.08

The highest biological process that is represented by these miRNA gene targets is synapse organization (bolded).

## Discussion

**Figure 7:**
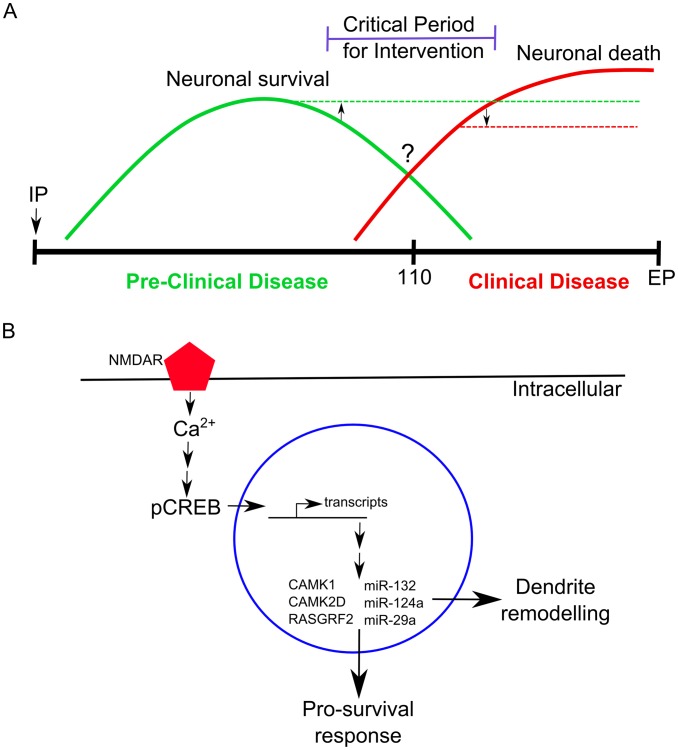
A schematic representation of the molecular changes we observed in neurons during prion disease. (**A**) A time-line showing the relative abundance of molecular signatures during pre-clinical and clinical prion disease in CA1 hippocampal neurons. Neuronal survival genes (solid green line) are most abundantly expressed during pre-clinical disease. At approximately 110 days post infection an unknown switch occurs (?) where neurons begin to elicit a death response (solid red line) which increases in abundance during clinical progression. A critical period exists for potential intervention strategies where targeting either the neuronal survival (upwards arrow and broken green line) or neuronal death mechanisms (downwards arrow and dashed red line) may help prolong disease onset. IP refers to the intraperitaneal inoculation route at 0 days post infection. (**B**) An example of molecular mechanisms that we observed to be affected during early prion disease. Synaptic NMDARs are stimulated, allowing for the influx of Ca^2+^ to enter the neuron which activates numerous signaling pathways one of which leads to the phosphorylation of CREB. The phosphorylated CREB activates transcription of many genes, including other transcription factors, within the nuclease resulting in the direct and indirect up-regulation of genes such as CAMK1, CAMK2D, RASGRF2, DOCK1 and microRNAs such as miR-132-3p, miR-124a-3p, miR-29a-3p. These effector molecules help neurons evoke a pro-survival response and stimulate dendrite remodeling mechanisms.

We have demonstrated that hippocampal CA1 cells undergo a clear temporal transcriptional response to the challenge of infection and propagation of prions. During pre-clinical disease a persistent stimulation of a programmed response was identified that appears to be mediated, at least in part, by synaptic NMDAR signaling. The coordinated program of the induced gene expression suggests the promotion of cell survival and neurite remodeling pathways. This response terminates prior to the onset of clinical symptoms in the infected hippocampus, seemingly pointing to a critical juncture in the disease (**Figure 7**). The changes described here center on the pre-clinical stages of disease at which time many of the gene alterations we identified are novel to prion disease.

### Cellular architecture of the CA1 hippocampal region during disease

Factors intrinsic to neurons are likely crucial for the initiation of degeneration in prion disease as prion replication in brain cells other than neurons does not appear to trigger neuronal death, and consequently, the clinical stage of disease [63]–[65]. Genome wide analysis of gene expression is a powerful tool for understanding the underlying pathogenic mechanisms of diseases. Several genome wide studies have been performed to date but the information they provide is very broad and encompasses all cell-types specific pathologies, with the most marked expression changes relating to gliosis. Discrimination between alterations in affected neurons and other cell types would increase the specificity of subsequent analysis and enable the determination of temporal changes specific to one cell type.

We chose to study CA1 hippocampal neurons, cells that are known to undergo physiological and morphological changes in mouse models of scrapie beginning at pre-clinical stages of infection. Our analysis firstly demonstrated that we were able to isolate relatively homogenous populations of neurons by LCM from mice sacrificed at various time-points throughout the incubation period of RML scrapie. The expression levels of neuronal marker genes, based on microarray signal intensity, was extremely high in our samples versus microglial and astrocyte markers, which were below the threshold set for detection by our arrays. These data correlated very well with immunohistochemical staining that revealed the presence of a few IBA1 stained microglial cell bodies and even less astrocytes amongst the densely packed neuronal layer. Astrocyte cell bodies stained for the marker GFAP were more abundant in areas through which dendrites of CA1 neurons pass. Although glial cell proliferation and activation is apparent within the diseased brain from the earliest times at which PrP^Res^ is detectable, their cell bodies do not appear to infiltrate the densely packed CA1 neuronal layer until late in disease. Accordingly, microglial and astrocyte genetic markers and the cytokines/chemokines they produce upon activation are not detectable by microarray in our LCM samples prior to EP. Therefore, we are confident that those alterations detected at pre-clinical stages of disease are highly enriched for CA1 neuron expressed genes.

Furthermore, FluoroJade C staining and analysis of neuronal marker genes was not indicative of widespread degeneration and loss of neurons within the LCM region. In fact, the neuronal cell bodies within this area appear relatively robust; other neurons in the hippocampal formation appeared more vulnerable to degeneration. Neurons in other brain regions, especially the thalamus area, also showed significant degeneration that was detectable using FluoroJade C at pre-clinical stages of disease. It will be of interest to compare the transcriptional response of these more vulnerable neurons to those of the CA1 layer. These neurons may well expose the pathways that lead to neuronal cell death from those that modulate the synaptic and morphological changes reported in the hippocampus.

### Comparison of the genes differentially expressed in neuronal CA1 hippocampal cells with global brain profiles

Although it is beyond the scope of this paper to compare and contrast all of the gene expression changes we identify with previous studies, it is important to make some general comments for comparative purposes. Most importantly, direct comparison with our own previously published data confirms that LCM in this case significantly increased the sensitivity for detecting altered genes that are associated with neuronal function, such as the transmission of nerve impulses and neuron projection morphogenesis. Particularly striking was the approximate 4-fold increase in the number of genes detected that are involved in synapse formation and processes relating to behavioral changes in mice. These gene expression changes intricately mirror the alterations in synaptic properties, and the morphology of dendritic spines, that are the recognized neuropathalogic features of early disease [1], [2], [66], [67].

A brief comparison of our data with the comprehensive genomic analysis of prion infected whole brain tissue presented by Hwang and colleagues [5] serves to illustrate some of the ways in which our targeted LCM studies can complement information gleaned on a global scale. For example, Hwang *et al* reported the novel finding that the global stimulation of the androgen biosynthesis pathway is a general feature of prion disease, implying that altered levels of neurosteroids may play a role in pathogenesis. These biosynthetic pathways were not detected in our study presumptively because cells other than those in the CA1 are carrying out these syntheses. However, multiple neurosteroid-induced signaling pathways such as the aldesterone and corticotropin-releasing hormone signaling pathways were found to be significantly deregulated in our data, interestingly at the earlier time-points (data not shown). These changes likely reflect neuronal physiology or perhaps pre-activation microglial alterations, thus, further supporting a link between perturbed androgen metabolism and glial activation or neuronal-glial signaling. Other metabolic pathways of interest identified by Hwang *et al*. [5] were those for arachidonate and prostaglandin synthesis. We found these pathways to be significantly deregulated at early time-points as well, suggesting their dysregulation in CA1 resident cells. No doubt, numerous other parallels could be drawn by detailed comparisons of individual genes.

We did look amongst our genes for clear evidence of pathways that may be involved directly in the cell death of neurons. One that may be important in prion induced neurodegeneration is activation of the endosomal and lysosomal pathway, or autophagy, within neurons [68]. However, we did not see any gene deregulation indicative of an increase in endosomal and lysosomal compartments or the autophagic system in our LCM neuronally enriched samples. The lysosomal activation marker LAMP2 is up-regulated only at EP, and we saw no change in the mRNA levels of the autophagy marker genes BECN1 (beclin 1), ATG5 and MAP1LC3B (LC3-II). This contrasts with some studies suggesting that either the activation of these systems within neurons, or their aberrant dysfunction, is an early and pervasive mechanism instigating the degenerative process [69]–[72]. This suggests that any changes we see in endosomal/lysosomal related genes over basal levels are limited to phagocytic cells clearing debris, or else, only triggered within terminal neurons long after the synaptic alterations are apparent. However, in assessing our data we also need to consider that this study relates to a highly specific group of neurons within the hippocampal CA1 region. As previously mentioned, FluoroJade C staining revealed that these neurons seem particularly robust in comparison to other neurons affected during prion disease. Therefore, it is possible that autophagy may play a larger role in these more vulnerable neurons. In addition, autophagy is a fundamental process that is carried out in all neurons and perhaps the process itself is important in prion pathobiology, but the up-or down-regulation of these pathways at the gene level does not occur. Further studies on other neuronal subsets that display differing vulnerabilities to prions will be of great interest in delineating the responses of different neurons and perhaps identify novel or cell-type specific pro-death programs. These are very important considerations for the development of appropriate therapies.

### Pre-clinical transcriptional alterations

Gene expression within the CA1 neurons is dynamic and appears to have at least two distinct phases. In this manuscript we describe the earliest detectable transcriptional alterations induced in infected mice that occur prior to any major loss of cells. At this stage, beginning between 40 and 70 DPI and ending prior to 130 DPI, we see a major cluster of altered genes and miRNAs that either return to basal levels, or alternatively undergo a direct reversal in expression profile during clinical disease. Notably, ontological analysis at each time point revealed that many of those neuronal specific genes were altered at pre-clinical stages of infection (**Figure S8**). Individual genes include alterations in receptors and ion channels that may lead to changes in the synaptic properties of the CA1 cells. Also altered are numerous cytoskeletal processes involved in dendrite morphology and synapse assembly. Pre-clinical changes also involve a regulatory response to stimulus and stress, including mitochondrial and spliceosomal changes. The dysregulated expression of genes involved in vesicular transport and function are evident by day 110 post-infection and it is not until day 130 that we see some expression of genes involved in the positive regulation of programmed cell death. Although we identified a number of genes involved in cell death and apoptosis, the list was quite small and does not point to a specific predetermined pathway.

Although at least 150 genes were up-regulated pre-clinically, these did not fall into many functional groups according to annotated ontologies. We looked at these in more detail and noticed that many were activity-regulated genes that are induced by various neuronal stimuli. Many, such as FOS and EGR1, are known to share binding sites for the trans-acting transcription factor CREB. Although CREB was not up-regulated at the mRNA level in the early stages of disease we were able to confirm by immunohistochemistry that phosphorylated CREB (the active form) is increased in CA1 pyramidal neurons during pre-clinical prion disease, correlating with the upregulation of potentially CREB regulated genes. Synapse dysfunction, compromising nuclear calcium signaling, is known to play a key role in multiple neurodegenerative diseases that are associated with the loss of synapses [73]–[74], of which prion disease is one example [75]–[77]. In this model, compromised synaptic signaling leads to the induction of calcium regulated genomic programs that are postulated to result in the degeneration and eventual death of neurons.

In recent years, a number of studies have shown that NMDA receptor signaling can play pivotal roles in the development of these pathologies as well as in other conditions such as ischemia, epilepsy and schizophrenia [78]–[80]. Additionally, NMDAR signaling was observed to be altered in PrP^C^ knockout mice in a number of reports showing that there is an impaired synaptic inhibition taking place via weakened GABA_A_ receptor-mediated fast inhibition [81] and an increased excitability of dentate gyrus hippocampal neurons from slice cultures [82]. Recent data further supports this observation by showing that PrP^C^ knockout enhanced NMDAR activity causing heightened excitability states of neurons and enhanced glutamate excitotoxicity, both *in vitro* and *in vivo*, suggesting that normal PrP^C^ mediates a neuroprotective role [83]. Therefore, we were very interested to discover that, in a meta-analysis of our data with other published sets of genomic data from hippocampal neurons, our gene list was highly reminiscent of that created by stimulation of synaptic NMDARs in primary hippocampal neurons [47].

NMDA receptors have distinct subunit compositions and subcellular locations that allow them to partner with different signaling pathways, creating functional diversity [84]. The NMDAR is composed of a subunit coded by three regulatory subunits whose composition can vary. The NR2 subunits reportedly differ between synaptic and extrasynaptic receptors. NR2B–containing receptors are more commonly located extrasynaptically and appear to promote cell death by inhibiting ERK1/2, inactivating CREB and stimulating excitotoxicity [85]–[87]. In turn, NR2A subunits appear to be involved in promoting cell survival [88]. It has been postulated that the ratio of NR2A and NR2B subunits also contributes to a balance between the protective and pro-cell death pathways [89], [90]. We found that GRIN2B, which is the gene coding for the NR2B subunit, was significantly down-regulated, according to microarray analysis, during early prion infection (70–90 DPI) with levels rising at later stages of disease. This finding was further validated by qRT-PCR. Interestingly, it has been previously reported that with decreased levels of NR2B subunit, the NR2A subunit increases in abundance at the synapse [91]. Therefore, our data is suggestive of an alteration in the ratio of these subunits towards a NR2A enriched composition in early stages of disease, substantiating the NMDAR neuroprotective response. Although some studies suggest that both NR2A and NR2B can activate cell death and survival [92], [93], NR2B receptor location preferentially at the extrasynaptic sites [94], [95] may reflect the pro-death phenotype. Additionally, we validated the expression of the transcription factor TRIB1 (tribbles), also found in the Zhang study [47]. The function of this gene is not well described, however, members of this family appear to be involved in the regulation of a number of fundamental signaling pathways and have been implicated in numerous human diseases [96]. This may well be an important regulatory molecule to investigate further in regards to neurodegeneration.

It has often been suggested that the lack of a proper neuroprotective response to the stress caused by prion build-up is at the heart of the neurodegenerative process. However, our analysis of temporally induced changes suggests that synaptic signaling leading to the induction of an anti-apoptotic genetic program is evident early in the course of disease. Perhaps, as disease progresses, these neuroprotective programs are gradually either shut-off or actively antagonized by a pro-death system. Excessive glutamate mediated NMDAR signaling (excitotoxicity) that initiates neurodegenerative processes has been linked to the development of a number of chronic disorders including Alzheimer's disease (AD) and perhaps prion disease [97]. It was therefore possible that the shift in gene expression patterns that occurs between 110 and 130 DPI represents a turning-point after which the over-stimulation of NMDARs switches from protection towards the induction of excitotoxicity related cell death. We looked at our data set further to investigate this possibility but we found little evidence of the induction of gene expression indicative of excitotoxicity. Of note, Zhang and colleagues identified a second group of genes whose expression was stimulated by an excessive amount of glutamate at extra-synaptic NMDARs and were involved in cell death [46] [47]. The expression of these genes was detected in the CA1 samples in our analysis, however, we did not see any deregulation of these genes, including the reported ‘key’ genes DAPK1 and CLCA1 (data not shown).

Although we did not detect a clear pattern of NMDAR stimulated pro-death genes, this does not necessarily rule out extrasynaptic pro-death signaling as it is possible that either additional genes were below our threshold of detection in the LCM generated samples, or that the pathway may be induced post-transcriptionally. Indeed, the total number of pro-death genes identified in the Zhang study was much fewer, only 11 versus the 185 pro-survival genes. Although we did not find a clear pro-death genomic program we did identify some up-regulated genes that have been implicated in neuronal death in a number of acute and chronic neurodegenerative conditions. The gene FOXO1 appears to be activated in response to various stress stimuli, such as epileptic seizures and oxidative and endoplasmic reticulum stress, and its role appears to be to eliminate damaged neurons by apoptosis [98]. A FOXO target gene also found to be up-regulated was BBC3 (PUMA), a Bcl-2 homology 3 (BH3)-only member of the Bcl-2 family that is known to be involved in endoplasmic stress-induced apoptosis in neurons [99]. The role of endoplasmic reticulum (ER) stress is an avenue that should be further explored in prion diseased neurons as potential mechanisms leading to their ultimate death.

NMDA receptors are highly permeable to calcium ions and it has been postulated that disruption of Ca^2+^ homeostasis may be a major contributing factor in prion disease. Cellular PrP protein may even have a major direct role in Ca^2+^ homeostasis (for review, see [100]). This could occur via two means: PrP^C^ misfolding during disease interrupts Ca^2+^ homeostasis or PrP^Res^ oligomerization forms pores in cell membranes which are permeable to ions. The downstream effects are fluctuations in the amounts of Ca^2+^ found in neurons that results in the type of physiologic and genetic impacts we see in neurons during early stages of disease, such as alterations in neuronal excitation and neurite outgrowth. A number of groups have reported a reduced Ca^2+^ response in brains and/or cultured neurons infected by prions along with electrophysiologic and morphologic synaptic abnormalities that may be the result of perturbed Ca^2+^ signaling [45], [101]–[103]. We chose to validate some of the genes involved in these processes that were dysregulated in the microarray analysis at early stages of infection. We confirmed the early upregulation of CAMK2D, CAMK4 and CAMK1; Ca^2+^/calmodulin-dependent protein kinases that are activators of the transcription factor CREB. NMDA glutamate receptors are coupled to the activation of the Ras/Erk signaling cascade and to the maintenance of CREB transcription factor via RASGRF1 (p140 Ras-GRF1) and RASGRF2 (p130 Ras-GRF2), a family of calcium/calmodulin-regulated guanine-nucleotide exchange factors that activate the Ras GTPases [104]. Consistent with this, ischemia-induced CREB activation is reduced in the brains of adult Ras-GRF knockout mice while neuronal damage is enhanced [105]. We validated the expression of RASGRF2 which was significantly up-regulated 70 and 90 DPI providing further support for stimulation of this pathway during early disease.

We validated the early over-expression of HOMER1, another gene that is induced by synaptic activity and a novel finding in prion disease. HOMER1 is a gene belonging to a family of dendritic scaffold proteins that regulate group 1 metabotrophic glutamate receptor (mGluR) function and have roles in dendrite morphology [106]. It has also been shown to be up-regulated in neurons early in Alzheimer's disease [107]–[109]. The binding of HOMER1 to mGluR1 leads to the release of intracellular calcium and major cellular consequences such as neuronal excitability changes, enhancement of neurotransmitter release, the potentiation of the activity of NMDA or AMPA receptors and effects on dendrite spine density and morphology [110]–[113].

An additional role for Ca^2+^/calmodulin-dependent kinases is in chromatin remodeling by the inhibition of HDAC activity through phosphorylation [114]. Interestingly, one of the gene expression alterations we validated was the progressive downregulation of HDAC9 over the course of the disease beginning at 90 DPI. A seminal study has also linked the expression of HDAC9 with dendritic growth in neurons [115]. In this study, knockdown of HDAC9 appeared to promote dendritic growth and concomitantly, expression of the immediate early gene FOS was down-regulated independent of synaptic activity. This study suggested that chromatin remodeling and the nucleocytoplasmic translocation of HDAC9 was able to regulate activity-dependent gene expression and dendritic growth [115]. The progressive down-regulation of HDAC9 correlates well with the down-regulation of immediate early genes at later stages of disease. Deregulation of genes involved in dendrite, axon and cytoskeleton development (both down- and up-regulation) was particularly obvious throughout the course of prion disease. Expression of another related gene, DOCK1, was assessed using qRT-PCR which was found to be up-regulated at 70 and 90 while down regulated by 110 DPI and remained unchanged during clinical disease. Previous reports showed that DOCK1 is involved in cytoskeletal reorganization [116] by mediating axon outgrowth, attraction [117] and pruning [118]. Recently, additional evidence suggests that DOCK1 mediates regulation of spine morphogenesis in cultured hippocampal neurons [119], further highlighting an essential role in dendrite morphology early in disease that is reminiscent of the neuronal protective mechanisms.

Another set of genes identified as deregulated at early stages of disease were those involved in transport processes such as binding of unfolded proteins, the localization of proteins to clathrin-coated and synaptic vesicles as well as those involved in adhesion and the spliceosome. It was beyond the scope of this study to validate the genes involved in all of these processes. However, we did confirm the early increase in expression of myosin motor genes, MYO6, MYO5A and DAB2 that function in intracellular vesicle and organelle transport by clathrin-mediated endocytosis in non-neuronal cells. Interestingly, all of these proteins have been shown to be expressed in neurons, localized to synapses and enriched at the postsynaptic density [120]–[122]. MYO6 has many roles in hippocampal neurons such as the clathrin-mediated endocytosis of acid-type glutamate receptors, such as α-amino-3-hydroxy-5-methyl-4-isoxazolepropionic acid receptors (AMPARs), and the formation of synaptic structures and astrogliosis [123]. MYO6 also binds directly to DAB2 which has also been postulated to be a negative regulator of neurite outgrowth [124]. MYO5A has been shown to be pivotal for the extension of the endoplasmic reticulum into neuronal dendritic spines [125].

### Early miRNA expression changes

We were especially excited to confirm the deregulation of a number of miRNAs in CA1 neurons during early prion disease as this is novel to the field. In a number of ways we found that miRNA expression profiles mirrored gene expression patterns. In total, 17 miRNAs were identified as significantly dysregulated between 70–110 DPI and we successfully validated the expression levels of 6 of these miRNAs: miR-16-5p, miR-26a-5p, miR-29a-3p, miR-132-3p, miR-140-5p and miR-146a-5p. All of these miRNAs were highly up-regulated in infected samples as compared to controls at early stages of infection. All miRNAs, except miR-146a-5p, showed a similar pattern to many of the mRNAs that were up-regulated early in that they returned to basal levels by 130 DPI and were down-regulated in clinical disease. Therefore, we hypothesize that by association these miRNAs may well have related functions. Although published studies on the functions of many of these miRNAs are few, a number provide evidence for the function of miR-29a-3p, miR-124a-3p and miR-132-3p in neuronal synapse formation and plasticity. More specifically, the dendritic arborization and neurite outgrowth appears to be controlled, in part, by these miRNAs.

MiR-124a-3p is one of the most studied miRNAs in the nervous system and is important in the differentiation of progenitor cells into mature neurons [126], [127]. Recent evidence suggests that miR-124a-3p promotes embryonic [128], [129] and adult neurogenesis *in vivo* [130]. Although the exact mechanism remains unknown, multiple gene targets for miR-124a-3p have been identified. One recent target of miR-124a-3p is Ephrin-B1 [131] which functions to regulate cytoskeletal dynamics [132]. Interestingly, neurons with higher miR-124a-3p expression levels exhibited increased neurite outgrowth [132], similar to miR-124a-3p over-expression in isolated cortical progenitor cells [128]. Recent evidence has also shown that LHX2 down-regulation by miR-124a-3p is important in preventing the apoptosis of hippocampal and retinal neurons during development [55]. Perhaps, this indicates that miR-124a-3p could potentially have a general role in neuroprotection. Similarly, neuronal-specific functions have been extensively shown with miR-132-3p levels. One of the targets of miR-132-3p in hippocampal neurons is the Rho family GTPase-activating protein p250GAP [133]. The repression of this gene via miR-132-3p results in a greater dendritic complexity of hippocampal neurons [133]. More specifically, a greater number of stubby and mushroom shaped spines exhibiting longer protrusion widths have been observed [134]. In turn, miR-29a-3p was shown to target ARPC3, a component of the ARP2/3 actin nucleation complex that initiates branching in actin filaments [135], which regulates the morphology of dendritic spines [136]. The authors found that miR-29a-3p reduced the number of mushroom-shaped dendritic spines on hippocampal neurons while filopodial-like outgrowths became more frequent [136]. Interestingly, the authors suggested that miR-29a-3p may function as a homeostatic mechanism to counterbalance excessive positive cues to stimuli at the synapse [136].

The enrichment shown amongst the group of miRNAs deregulated pre-clinically in our prion model for those that play roles in dentrite and synapse formation fits very well with the ontology assignments of genes similarly deregulated. Synaptic disruption and dendritic abnormalites have long been recognized as the earliest pathologic consequence of prion replication in the brain and miRNAs up-regulated in early disease appear to be key regulatory molecules in these processes. Dendritic spines are quite dynamic throughout the lifespan of a CA1 neuron, however, over-stimulation effects by a ‘toxic’ agent may further enhance the change in dendrite and spine morphology; a function that may potentially involve miR-124a-3p, miR-132-3p and miR-29a-3p and possibly also the other miRNAs, such as miR-16-5p, miR-26a-5p, miR-140-5p and miR-146a-5p whose functions in neurons have yet to be characterized.

Recent publications have, however, revealed one interesting gene target of miR-16-5p. One of these is amyloid protein precursor (APP) and in a murine model of early-onset Alzheimer's disease the authors found a decrease in miR-16-5p levels while APP was increased [137]. Upon a short term infusion of miR-16-5p into the brains of diseased mice, the authors found a decrease in APP accumulation, further suggesting that miR-16-5p has an inhibitory effect on APP protein formation [137] and therefore, acts as a protective molecule in the disease. In prion disease, we saw an up-regulation of miR-16-5p during early disease and the expression of this miRNA decreased with disease progression. It would be an interesting area of study to determine whether miR-16-5p has a similar neuroprotective role in prion diseased mice.

Although little is known about specific functions of miR-26a-5p, miR-140-5p and miR-146a-5p within neurons, recent evidence points to their involvement within the central nervous system. More specifically, miR-26a-5p has been shown to regulate photoreceptor L-type voltage-gated calcium channel alpha1C in retinal cells [138]. MiR-140-5p targets EGR2, modulating myelination in dorsal root ganglion and Schwann cell co-cultures [139] while miR-146a-5p has been identified in the CA1 neurons of rat hippocampus using *in situ* hybridization [59]. Therefore, additional investigation into the neuronal function of these miRNAs may further enhance our understanding of prion-induced pathobiology.

### MiRNA expression during clinical disease

As miRNA expression in prion disease has not been extensively reported in the literature we provide a brief discussion of those miRNAs deregulated, or only detected at EP, in infected mice. By analogy with gene expression data we expect that the majority of these genes will correspond to glial expressed miRNAs involved in an innate immune response. Noteworthy is the miR-146a-5p, a well know immune regulatory miRNA that is induced in activated microaglia and was significantly up-regulated at the end-point of disease with a fold change of 3.09±0.13 (p-value≤0.01) (**Figure 5D**). Increased expression of miR-146a-5p in mouse scrapie, human prion disease (CJD and GSS patients) and Alzheimer's disease has been reported and it is suggested that this miRNA may be a general biomarker of neuroinflammation [49] [52]. Accordingly, this role may potentially be applied to the other similarly expressed miRNAs we described. Interestingly, we also found that miR-146a-5p is up-regulated early in disease, suggesting a pre-clinical function during disease in addition to a role in activated glia. Although miR-146a-5p is well studied in a number of different immune cells its expression in neurons has not yet been reported. We did confirm that we can detect miR-146a-5p expression in this region by qRT-PCR as well as in primary mouse hippocampal neuronal cultures (data not shown). In addition, *in situ* hybridization from our (data not shown) and Aronica *et al* [59] suggests that hippocampal CA1 neurons express this miRNA in significant levels in contrast to other neurons. Further work on miR-146a-5p will be required to determine if it has a neuronal specific function. In immune cells, miR-146a-5p targets TRAF5 and IRAK1 to suppress the inflammatory response to stimulation and we also showed in microglia that predicted targets may be involved in microglial morphology [54]. It will be interesting to determine whether miR-146a-5p has any similar targets in CA1 neurons and whether it plays a role in neuronal degeneration.

Of note, we were unable to detect miR-494-3p or miR-342-3p to be deregulated in our samples at clinical disease, both of which were detected in scrapie infected mice [49] and in BSE-infected macaques, a model for CJD [140]. However, we determined that miR-342-3p is expressed at low levels in the hippocampal CA1 neurons while a greater expression of this miRNA was observed in cerebellar granule neurons (data not shown). Therefore, we expect that this discrepancy is most likely attributed to these miRNAs being expressed in particular brain cell types other than hippocampal CA1 neurons.

### Conclusions

Taken together, our work provides insight into the previously unknown temporal nature of the transcriptional changes that accompany prion replication. Beyond the reduction of transcripts related to synaptic vesicle trafficking and dendrite morphology in neurons during pre-clinical infection, our data suggests that miRNAs may be involved as post-transcriptional regulators of mRNAs that are functionally associated to axonal and dendritic synaptic remodelling. Dendritic spines are specific sites of protein synthesis and it is generally thought that dendritic mRNAs are transported to that location as part of ribonucleoprotein complexes [141]. MicroRNAs are then able to modulate dendritic morphology by regulating expression of proteins involved in the actin cytoskeleton [142] [132], [143]–[145] mRNA transport [146] and neurotransmission. However, the role of post-transcriptional deregulation of genes functionally associated to axonal and dendritic synaptic remodeling in prion diseases is as yet unknown. Given the concomitant alterations in genes known to promote neuronal survival we postulate that remodeling of dendrite morphology during pre-clinical disease is a response to stress. Further investigatation of the roles of the prion-induced miRNA deregulation will provide an inroad for the study of these as yet unexplored pathomechanisms in disease. Ultimately this knowledge may contribute to the identification of therapeutic molecules targeted to specific neuronal processes that contribute to disease pathobiology. The identification of the multi-phasic nature of the neuronal response adds an extra layer of complexity to the design of therapeutics. We envision two approaches, either the maintenance of the initial genetic response to promote neuronal survival or blocking a later event that promotes neuronal cell death (**Figure 7A**), such as that described by Moreno *et al* [4]. The introduction or knock-down/out of miRNAs themselves is one potential avenue for novel drug treatment. Manipulation of miRNAs may affect a number of pathways simultaneously and may allow the modulation of a specific process, rather than a complete inhibition of a pathway for which a basal level is required by the cell. Continued investigation of transcriptional and post-transcriptional deregulation in prion neurodegeneration will undoubtedly identify the molecular basis for clinical disease and help to evaluate new drug treatments.

## Supporting Information

Figure S1 Representative Bioanalyzer readings for select RNA samples isolated using the LCM. Electropherogram readings showing marker, small RNA, 18S and 28S peaks along with a gel representation of each indicated sample. The RIN values and RNA concentrations are indicated. Samples that were below RIN of 5.9 (below the dashed line) were not used for further downstream applications and are only included for comparative purposes (ie. sample with RIN of 3.1).
(PNG)

Figure S2 The determination of suitable normalization controls from miRNA TLDA cards using StatMiner stability methods. (**A**) The Ct profiles show variability in Ct signal throughout the time course experiment for select miRNAs. Two small RNA species were manually chosen based on minimal Ct ranges observed throughout the time course experiment and compared to the snoRNA U6, a commonly used control. The graph represents 2 TLDA cards per treatment for each time point tested. (**B**) Readout graphs representing stability scores for each RNA species analyzed separately and in combination. The lower the stability score or M value, the more stable the small RNA throughout the system. Highlighted throughout these graphs is the most stable control RNA, snoRNA-135.
(PNG)

Figure S3 A flow diagram representing the experimental methods we employed to determine both mRNA and miRNA expression profiles. Briefly, mice were inoculated intraperitaneally (IP) with the RML strain of scrapie (arrow at 0 DPI) after which we collected samples at 40, 70, 90, 110, 130 and EP days post inoculation. The increasing red gradient represents accumulating PrP^Res^ deposits in the hippocampal region of RML infected animals. From each sample that was collected, serial sections were prepared, stained and the CA1 neuronal dense regions were captured using the LCM. Total RNA was extracted and only samples that passed our quality control cut-offs were used for downstream processing, such as mRNA and miRNA profiling.
(PNG)

Figure S4 GAPDH levels in CA1 hippocampal regions in both control and infected samples throughout prion infection. Gene expression and relative fold change (line graph) of GAPDH for both control (dark gray bars) and infected (light gray bars) samples. Both of these are represented on a log scale. The horizontal dotted red line represents the signal intensity threshold set at 100.
(PNG)

Figure S5 The detailed image of Figure 3B and C showing visible annotation of the gene functional groups for (**A**) up-regulated and (**B**) down-regulated genes. Each node is labeled with the respective time point and the node labeled “160” reflects EP.
(PNG)

Figure S6 A hierarchical cluster plot of differentially expressed immune-related genes from CA1 areas that were also found in prion infected whole brain tissue and other neurodegenerative conditions. Red bars represent up-regulated genes while green bars represent down-regulated genes in RML infected samples as compared to controls. Arrows indicate genes further alluded to in the text. “End” refers to the end point (EP) of disease.
(PNG)

Figure S7 The significantly expressed miRNAs in RML samples during clinical stages of prion infection. (**A**) The average normalized Ct values (delta Ct) for 4 miRNAs that were only detected in RML samples at clinical stages of prion disease. (**B**) A total of 4 miRNAs exhibiting a heightened expression level in prion-infected samples as compared to controls during clinical disease.
(PNG)

Figure S8 The temporal expression profile of genes belonging to different ontological groups. Red bars represent up-regulated genes while green bars represents down regulated genes for each group.
(PNG)

Table S1 List of genes that were differentially expressed during at least one time-point in prion infected versus mock-infected mice. The criteria to determine significance was an FDR<1% and a fold change of at least 2.5. Empty spaces reflect lack of signal for the indicated probe in control or infected sample.
(PDF)

Table S2 List of miRNAs that were differentially expressed during at least one time-point in prion infected versus mock infected mice (p-value≤0.1). The bolded miRNAs were found to be significantly deregulated during pre-clinical disease.
(PDF)

Table S3 List of miRNAs that were up-regulated or only expressed in the infected samples at clinical stages of prion disease. Empty spaces reflect lack of signal for the indicated probe in control or infected sample.
(XLS)

Table S4 List of 44 up-regulated miRNAs at DPI 130 and/or EP in prion disease and also over-expressed in LPS-stimulated astrocytes.
(XLS)

Table S5 The expression level of miRNAs identified at 130 and EP of prion disease via next generation sequencing (NGS) data from rat hippocampal neurons. The specific NGS data was collected by Zovoilis and colleagues [47].
(XLSX)

Table S6 The list of 17 significantly differentiated miRNAs detected during pre-clinical stages of prion disease (p-value≤0.1). The average fold change and standard error (SEM) are listed for each miRNA.
(XLSX)

Table S7 List of mature miRNA sequences for the 7 miRNAs that we successfully validated. MiRNA sequences in black represent mature mouse sequences while the ones in red represent rat sequences.
(DOC)

Table S8 The list of the 80 gene ontology biological processes indentified by the 396 mRNAs predicted to be targeted by at least 1 of the 7 miRNA candidates.
(XLSX)
