# Supplementary figures and images for: Early Mechanisms of Pathobiology Are Revealed by Transcriptional Temporal Dynamics in Hippocampal CA1 Neurons of Prion Infected Mice
Source: PLoS Pathog. 2012 Nov 8;8(11):e1003002. doi: 10.1371/journal.ppat.1003002 (PMC3493483; doi:10.1371/journal.ppat.1003002)

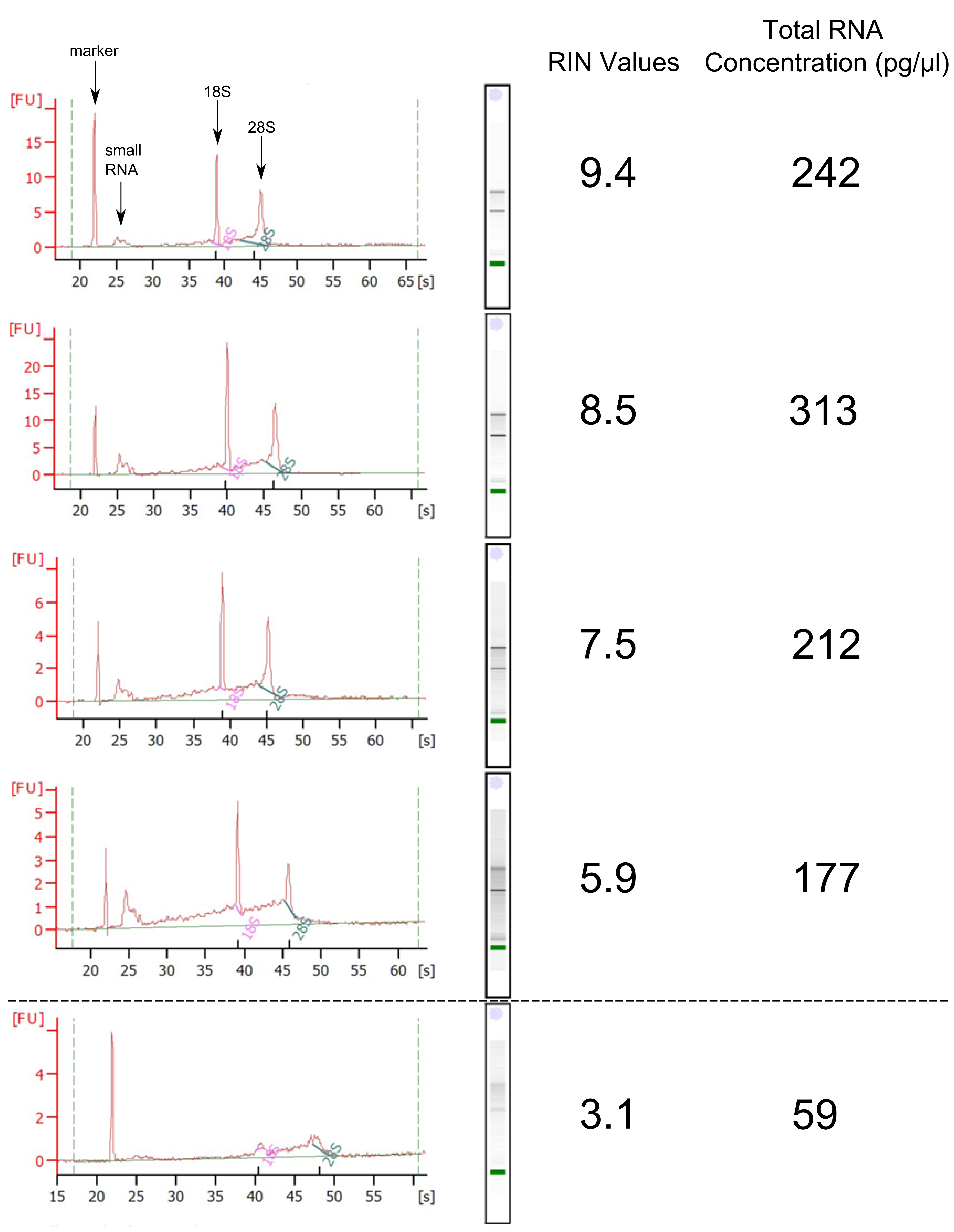

Supplement: Figure S1 — Representative Bioanalyzer readings for select RNA samples isolated using the LCM. Electropherogram readings showing marker, small RNA, 18S and 28S peaks along with a gel representation of each indicated sample. The RIN values and RNA concentrations are indicated. Samples that were below RIN of 5.9 (below the dashed line) were not used for further downstream applications and are only included for comparative purposes (ie. sample with RIN of 3.1). (PNG) [file ppat.1003002.s001.png]

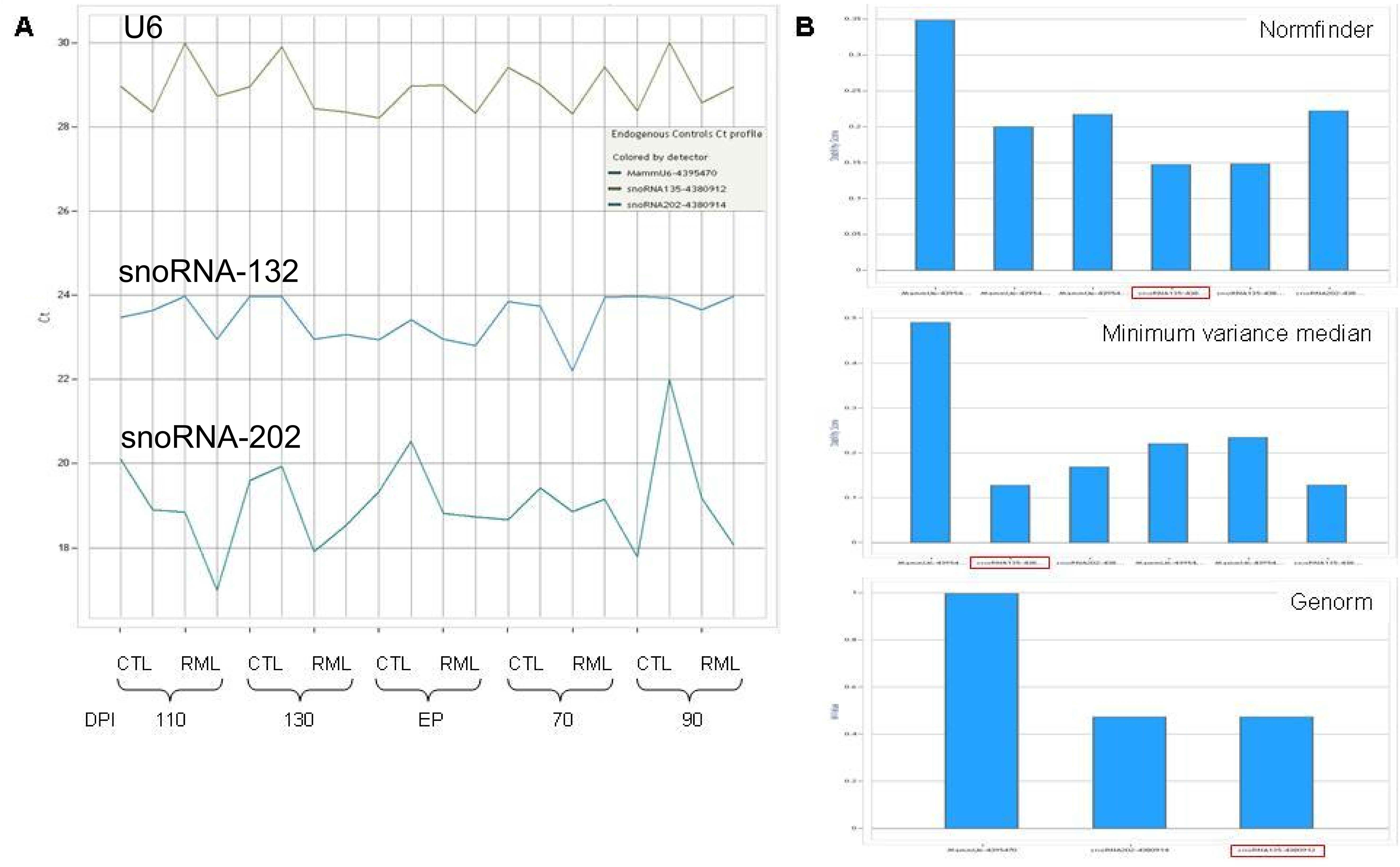

Supplement: Figure S2 — The determination of suitable normalization controls from miRNA TLDA cards using StatMiner stability methods. (A) The Ct profiles show variability in Ct signal throughout the time course experiment for select miRNAs. Two small RNA species were manually chosen based on minimal Ct ranges observed throughout the time course experiment and compared to the snoRNA U6, a commonly used control. The graph represents 2 TLDA cards per treatment for each time point tested. (B) Readout graphs representing stability scores for each RNA species analyzed separately and in combination. The lower the stability score or M value, the more stable the small RNA throughout the system. Highlighted throughout these graphs is the most stable control RNA, snoRNA-135. (PNG) [file ppat.1003002.s002.png]

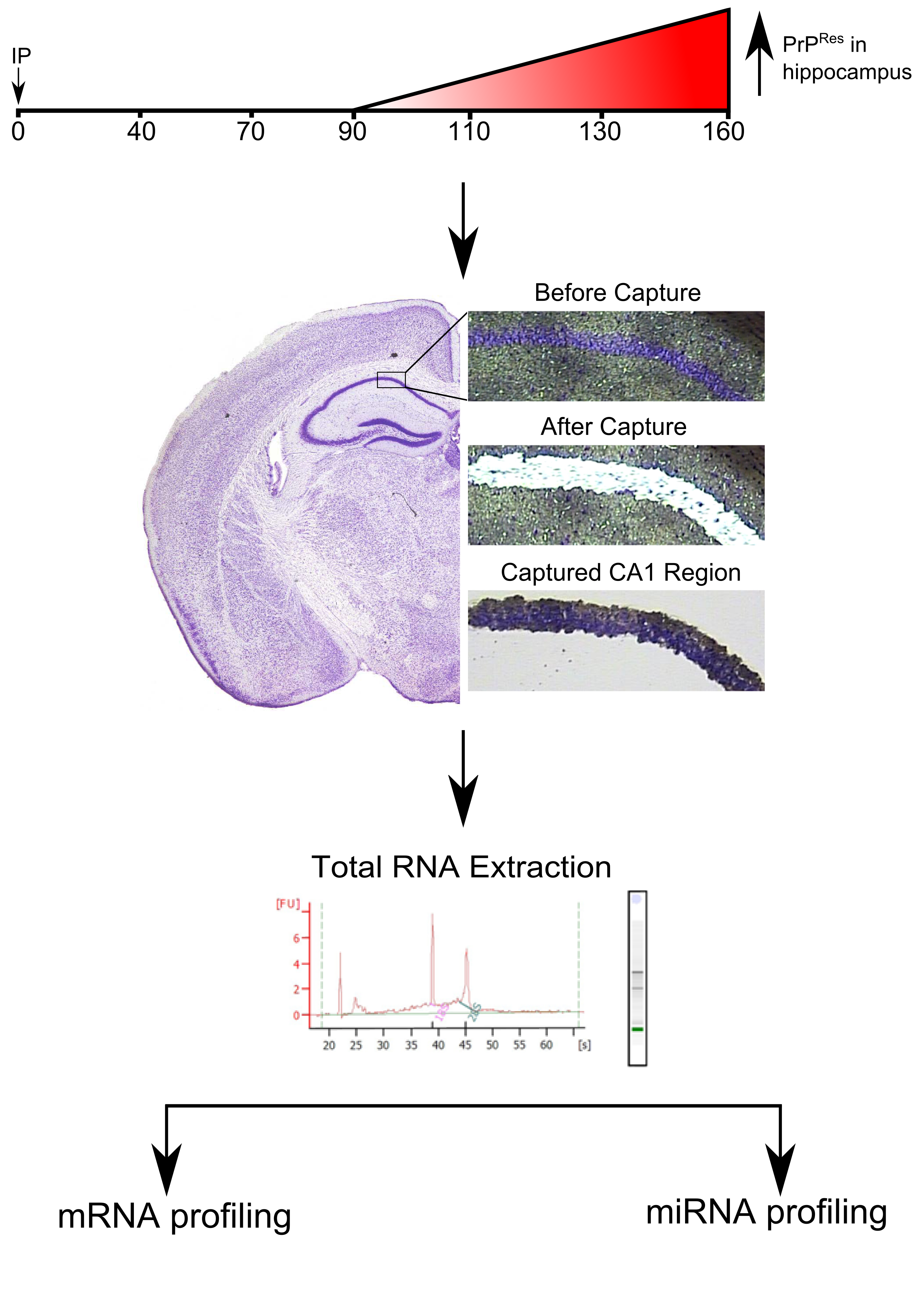

Supplement: Figure S3 — A flow diagram representing the experimental methods we employed to determine both mRNA and miRNA expression profiles. Briefly, mice were inoculated intraperitaneally (IP) with the RML strain of scrapie (arrow at 0 DPI) after which we collected samples at 40, 70, 90, 110, 130 and EP days post inoculation. The increasing red gradient represents accumulating PrPRes deposits in the hippocampal region of RML infected animals. From each sample that was collected, serial sections were prepared, stained and the CA1 neuronal dense regions were captured using the LCM. Total RNA was extracted and only samples that passed our quality control cut-offs were used for downstream processing, such as mRNA and miRNA profiling. (PNG) [file ppat.1003002.s003.png]

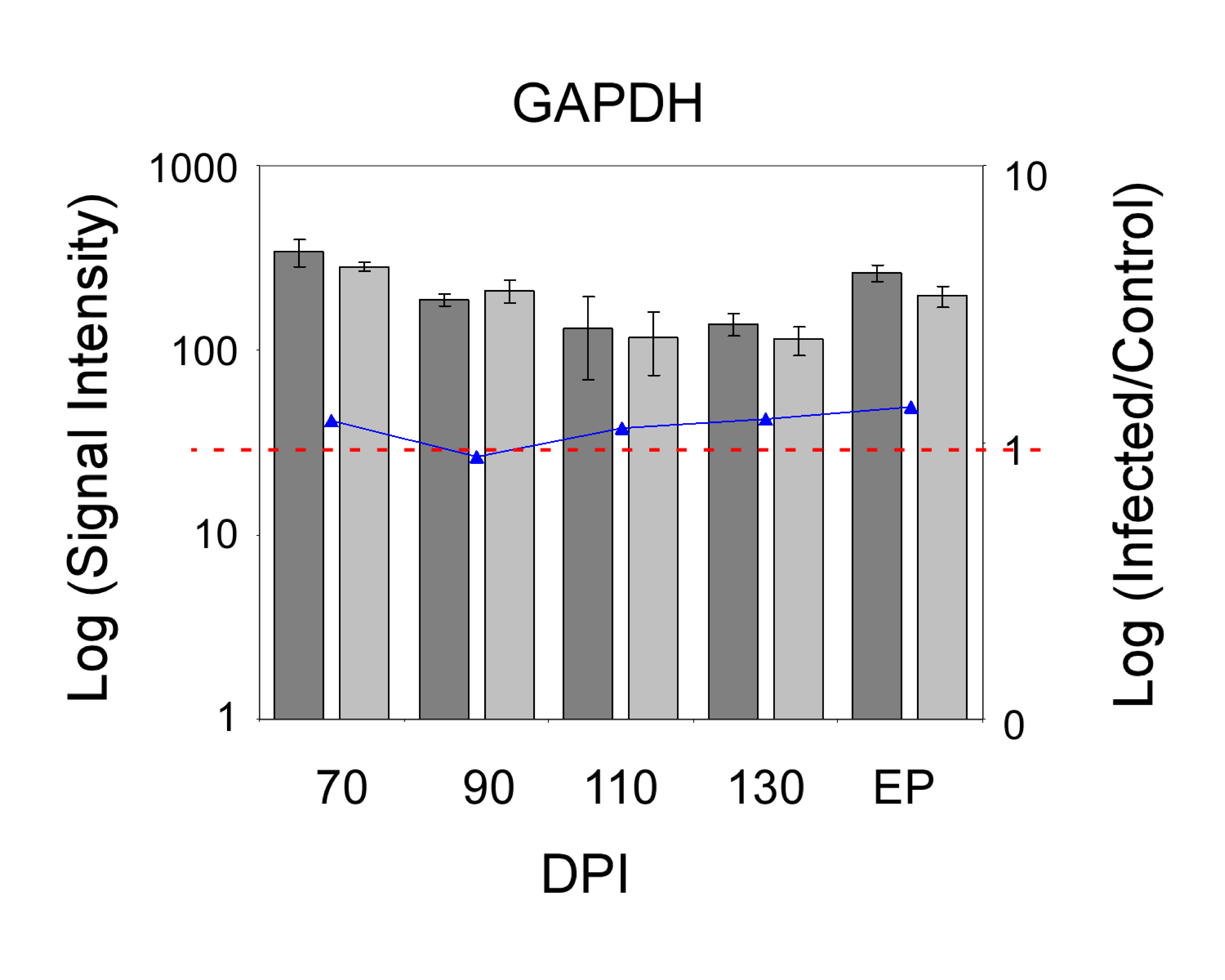

Supplement: Figure S4 — GAPDH levels in CA1 hippocampal regions in both control and infected samples throughout prion infection. Gene expression and relative fold change (line graph) of GAPDH for both control (dark gray bars) and infected (light gray bars) samples. Both of these are represented on a log scale. The horizontal dotted red line represents the signal intensity threshold set at 100. (PNG) [file ppat.1003002.s004.png]

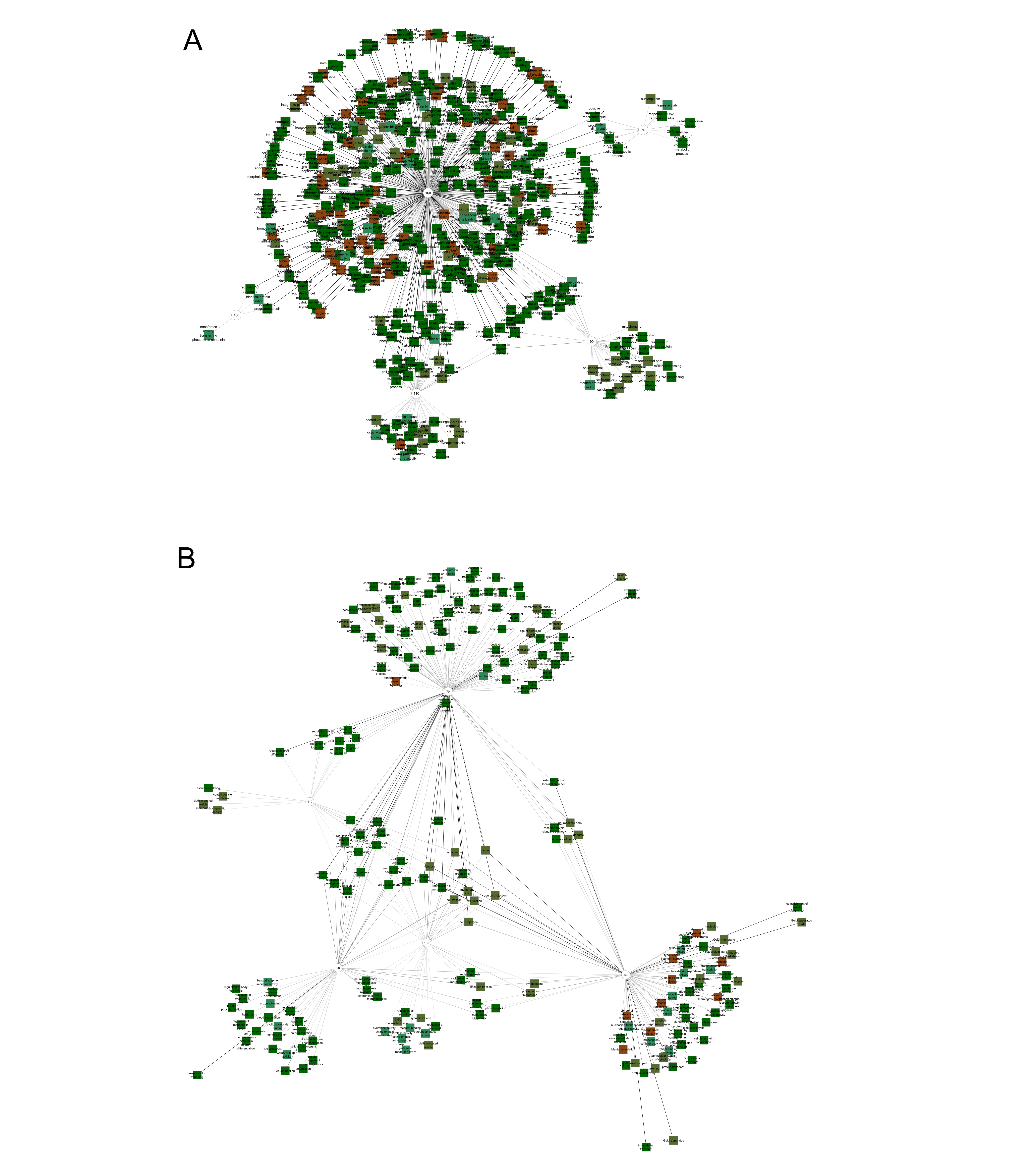

Supplement: Figure S5 — The detailed image of Figure 3B and C showing visible annotation of the gene functional groups for (A) up-regulated and (B) down-regulated genes. Each node is labeled with the respective time point and the node labeled “160” reflects EP. (PNG) [file ppat.1003002.s005.png]

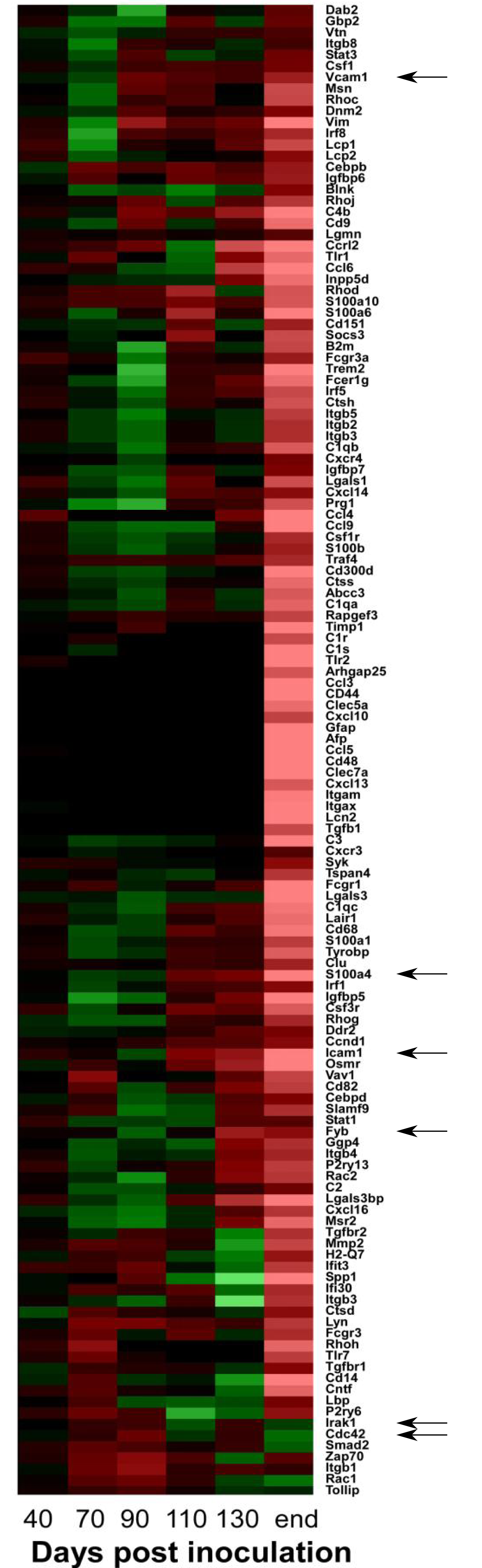

Supplement: Figure S6 — A hierarchical cluster plot of differentially expressed immune-related genes from CA1 areas that were also found in prion infected whole brain tissue and other neurodegenerative conditions. Red bars represent up-regulated genes while green bars represent down-regulated genes in RML infected samples as compared to controls. Arrows indicate genes further alluded to in the text. “End” refers to the end point (EP) of disease. (PNG) [file ppat.1003002.s006.png]

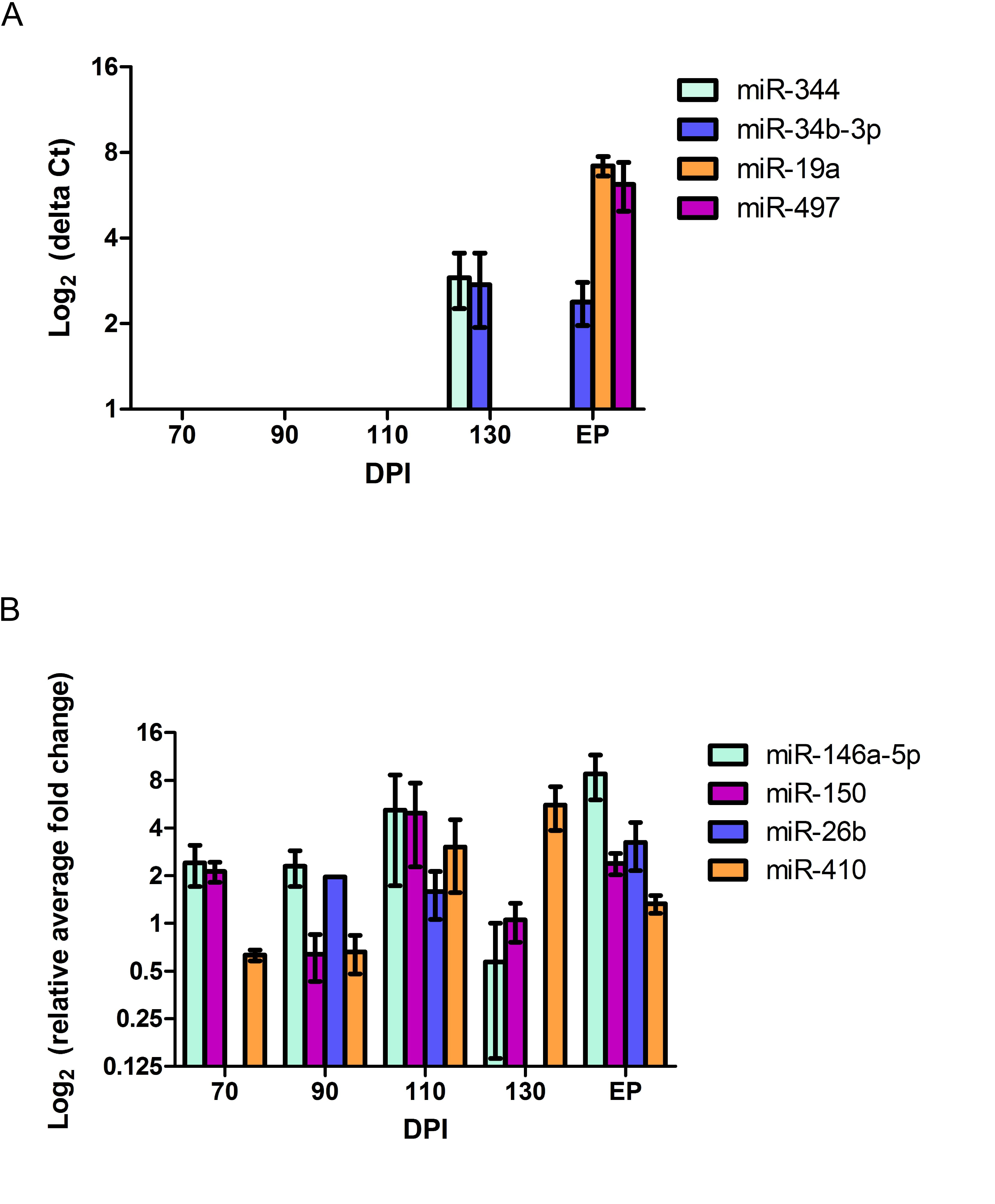

Supplement: Figure S7 — The significantly expressed miRNAs in RML samples during clinical stages of prion infection. (A) The average normalized Ct values (delta Ct) for 4 miRNAs that were only detected in RML samples at clinical stages of prion disease. (B) A total of 4 miRNAs exhibiting a heightened expression level in prion-infected samples as compared to controls during clinical disease. (PNG) [file ppat.1003002.s007.png]

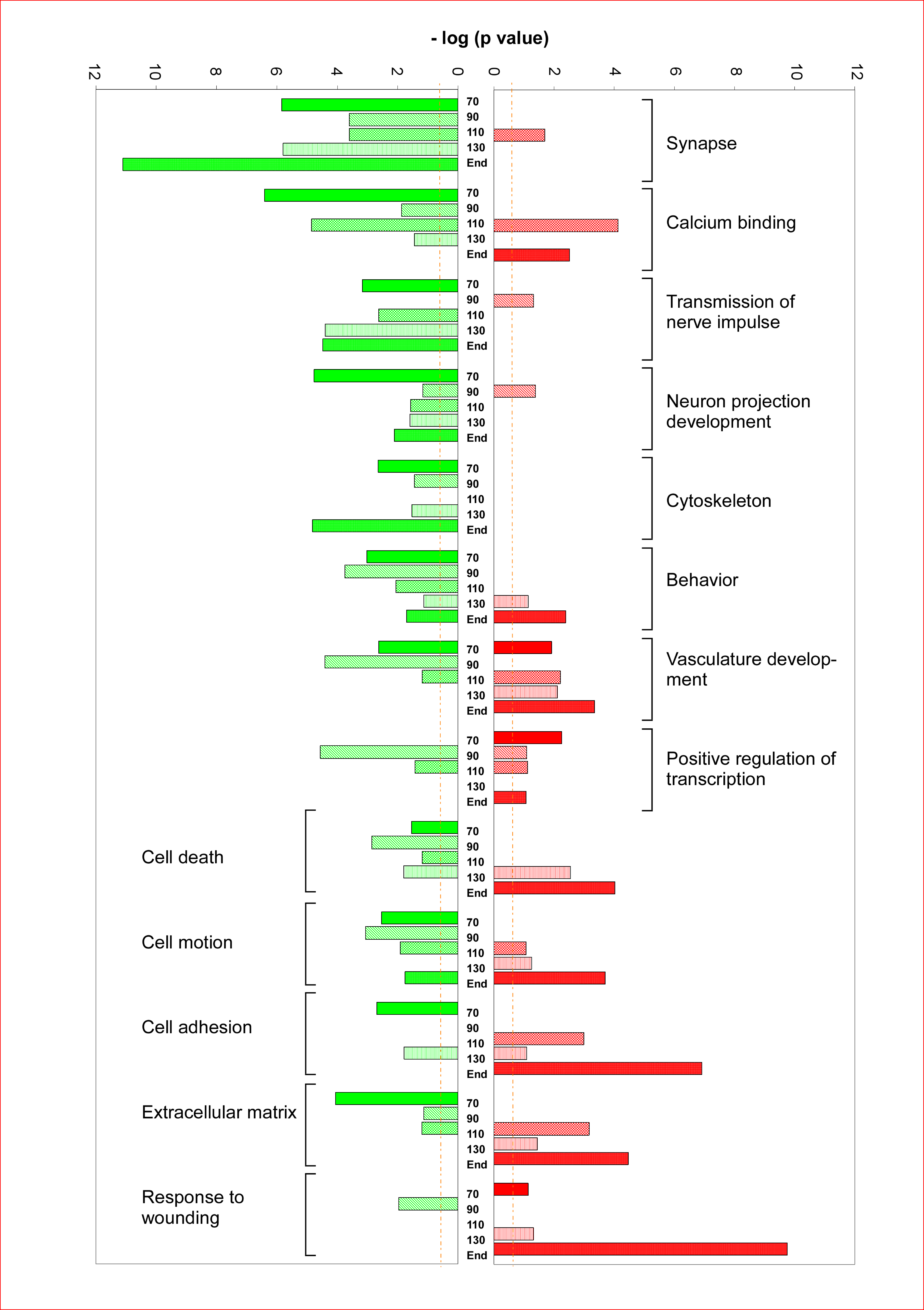

Supplement: Figure S8 — The temporal expression profile of genes belonging to different ontological groups. Red bars represent up-regulated genes while green bars represents down regulated genes for each group. (PNG) [file ppat.1003002.s008.png]
