# Supplementary material for: Early Mechanisms of Pathobiology Are Revealed by Transcriptional Temporal Dynamics in Hippocampal CA1 Neurons of Prion Infected Mice
Source: PLoS Pathog. 2012 Nov 8;8(11):e1003002. doi: 10.1371/journal.ppat.1003002 (PMC3493483; doi:10.1371/journal.ppat.1003002)
Supplement: Table S1 — List of genes that were differentially expressed during at least one time-point in prion infected versus mock-infected mice. The criteria to determine significance was an FDR<1% and a fold change of at least 2.5. Empty spaces reflect lack of signal for the indicated probe in control or infected sample. (PDF) [file ppat.1003002.s009.pdf]

	70 DPI		90 DPI		110 DPI		130 DPI		EP	
	Log Ratio	FDR	Log Ratio	FDR	Log Ratio	FDR	Log Ratio	FDR	Log Ratio	FDR
**5430435G22Rik**	-0.464	0.126	-0.074	12.522	-0.115	33.124	0.134	13.893	0.481	31.624
**A2LD1**	-0.141	3.157	-0.609	2.773	-0.180	12.575	0.138	41.825	0.451	0.213
**AADAT**	0.235	12.647	0.187	3.009	-0.130	6.932	-1.312	0.061	-0.150	24.398
**ABCA3**	0.124	49.540	-0.443	0.514	0.021	59.432	0.103	32.497	0.618	0.039
**ABCA5**	-0.378	0.511	0.262	0.595	-0.217	3.475	-0.643	11.241	0.246	24.398
**ABCB10**	-0.754	0.052	0.594	1.086	-0.372	4.053	-0.162	47.420	-0.489	8.002
**Abcb1b**	0.596	0.052	0.562	2.123	-0.405	2.659	-0.132	0.573	-0.265	6.001
**ABCC1**	-0.134	37.036	0.289	4.122	-0.157	22.899	-0.302	5.543	0.460	0.746
**ABCC3**	0.042	49.540	-0.204	3.009	0.062	49.127	-0.072	41.825	0.857	0.021
**ABCC5**	-0.172	15.278	0.806	0.602	0.060	49.127	0.244	3.866	0.642	0.021
**ABHD16B**	0.030	54.154	0.462	0.986	0.116	17.386	0.108	41.825	-0.249	0.307
**ABI1**	0.220	0.171	0.721	0.944	0.149	14.770	-0.100	19.971	-0.392	0.166
**ABI3**	0.174	37.036	-0.286	1.086	-0.103	36.892	0.145	32.497	0.606	0.021
**ABL2**	0.233	0.529	0.652	0.569	0.141	54.331	0.834	0.573	0.312	36.831
**ABLIM2**	-0.038	55.170	-0.284	5.829	0.267	2.056	0.221	8.987	0.512	0.066
**ABLIM3**	-0.092	29.668	-0.507	0.305	-0.142	20.025	0.198	3.866	0.197	2.886
**ACAD10**	0.200	0.780	0.352	6.703	0.015	58.928	0.910	0.061	0.118	43.175
**Acan**	-0.407	0.171	-0.242	2.859	-0.351	9.255	0.190	23.450	-0.146	33.879
**ACAT2**	-0.103	44.812	-0.478	5.829	0.204	8.121	-0.773	0.061	0.486	0.021
**ACBD3**	-1.233	0.052	0.071	7.537	-0.431	0.092	-0.890	0.061	-0.460	0.021
**ACCS**	0.193	5.928	-0.456	0.935	0.305	0.240	-0.110	49.358	0.339	24.398
**ACER2**	0.083	49.540	0.072	10.404	0.253	2.056	0.346	19.971	0.474	0.021
**ACOT9**	0.248	7.743	0.626	0.514	-0.189	2.311	0.049	50.493	-0.430	0.119
**ACP1**	-0.150	49.540	0.672	0.445	-0.301	26.120	0.533	0.292	-0.773	0.471
**ACP2**	-0.258	12.647	0.470	1.772	-0.162	33.124	-0.406	19.971	-0.664	0.021
**ACSL1**	-0.326	15.278	0.503	0.877	-0.348	1.556	-1.019	0.061	0.295	24.398
**ACTG2**	-0.259	0.170	0.390	0.595	0.565	4.733	-0.289	41.825	-0.274	24.398
**ACTL6A**	0.260	1.431	0.753	0.514	-0.036	56.823	-0.314	27.828	-0.135	31.624
**ACTR3B**	0.697	0.052	0.969	0.569	-0.159	2.659	-0.337	11.241	-0.411	6.001
**ACVR2A**	0.136	44.812	0.574	0.300	-0.537	2.311	0.487	16.936	-0.757	0.021
**ADA**	-0.167	1.431	-0.262	5.829	0.054	56.823	0.026	53.829	0.622	0.391
**ADAM17**	-0.237	5.928	0.279	7.537	-0.275	3.068	-0.913	1.210	-0.400	0.021
**ADAM19**	0.137	15.278	0.326	1.772	0.077	54.331	-0.774	1.617	-0.497	0.891
**ADAM8**	0.106	44.812	0.465	0.569	0.387	4.053	-0.444	32.497	0.153	31.624
**ADAMTS1**	-0.043	56.513	-0.173	11.783	-0.438	3.475	-0.721	0.906	0.118	44.203
**ADAMTS2**	-0.969	0.052	-0.911	0.986	0.138	41.103	0.509	1.210	0.781	0.045
**ADAMTS20**	0.298	0.666	0.418	0.300	0.173	26.120	0.164	13.893	0.121	43.008
**ADAMTS7**	-0.191	18.587	0.240	1.086	0.163	54.331	-0.191	32.497	1.174	0.021
**ADAMTS9**	-0.585	0.052	-0.902	2.859	0.134	29.328	-0.079	32.497	0.140	40.660
**ADARB2**	0.834	0.052	-0.520	2.859	0.558	0.092	-0.269	23.450	-0.341	0.391
**ADAT1**	0.125	44.812	-0.578	0.228	0.161	0.456	-0.556	0.109	0.307	24.398
**ADCY1**	-0.200	25.771	0.558	0.514	-0.069	56.823	-0.292	13.893	-0.427	0.993
**ADCY4**	-0.168	44.812	0.208	4.122	0.384	22.899	-0.193	32.497	0.939	0.021
**ADCY7**	-0.120	44.812	0.575	11.783	-0.334	10.694	0.814	0.506	1.133	0.084
**ADCYAP1**	0.103	44.812	-0.213	4.122	0.359	2.056	-0.545	0.341	-0.139	33.879
**ADI1**	-0.055	52.827	-0.602	0.107	0.103	20.025	-0.022	55.172	-0.251	0.166
**ADM**	0.282	0.052	-0.219	0.514			-0.480	0.250	-0.068	36.831
**ADNP2**	0.474	0.511	0.428	0.877	-0.181	3.475	0.098	11.241	-0.059	38.751
**ADORA2A**	-0.664	0.511	-0.272	3.009	0.134	54.331	0.199	19.971	0.130	14.822
**ADRA2A**	-0.152	3.157	-0.207	5.829	0.323	0.856	0.280	0.573	0.735	0.021
**ADRB1**	-0.140	9.997	0.678	0.514	0.102	54.331	0.247	0.121	-0.036	37.824
**ADRBK1**	0.222	0.529	0.484	1.029	0.715	1.819	0.195	23.450	0.583	0.021
**AFAP1L1**	-0.138	1.936	-0.478	0.784	0.371	3.068	-0.505	13.893	0.436	0.166
**AFF3**	0.189	21.797	0.527	0.514	-0.196	36.892	-0.173	27.828	-0.119	37.824
**AFF4**	-0.714	0.052	-0.576	2.773	-0.409	10.694	0.388	27.828	0.529	0.993
**AFG3L1**	-0.067	52.218	0.528	0.445	-0.208	9.255	0.378	1.332	0.375	11.001
**AFP**	-0.061	57.109	-0.771	1.086	-0.558	22.899	0.964	0.061	1.856	0.021
**AFTPH**	-0.136	49.540	0.253	6.703	-0.309	0.440	0.741	2.415	-0.714	0.021
**AGA**	0.454	0.666	0.509	0.986	-0.198	0.856	0.339	32.497	0.042	44.052
**AGBL5**	-0.418	0.398	-0.304	3.009	0.153	33.124	-0.064	41.825	0.628	0.039
**AGPAT1**	-0.096	49.540	-0.387	0.784	0.138	20.025	0.402	13.893	0.498	0.021
**AGPAT2**	-0.061	49.540	-0.103	9.629	-0.265	14.770	0.409	4.906	0.777	0.021
**AGPAT9**	0.170	1.431	0.543	0.107	0.203	2.311	1.009	0.061	-0.493	0.021
**AGPS**	-0.436	4.284	0.570	0.107	0.072	49.127	-0.452	13.893	-0.533	0.391
**AGR2**	-0.476	0.052	-0.290	0.986	-0.056	58.611	0.401	23.450	-0.202	14.822
**AGT**	-0.488	0.052	0.356	0.420	-0.713	0.092	0.530	0.292	0.821	0.062

**AGTR1**	0.457	29.668	-0.660	0.180	-0.288	29.328	-0.846	0.061	-0.663	0.021
**AGXT2L1**	-1.083	0.052	-0.269	0.595			1.063	0.947	1.153	0.213
**AHCTF1**	0.265	21.797	0.726	1.212	0.209	0.856	0.375	1.332	-0.272	31.624
**AHCYL2**	-0.505	0.666	0.282	2.300	-0.319	2.659	0.493	0.061	-0.714	0.021
**AHDC1**	-0.170	9.997	0.282	0.354	-0.213	29.328	0.164	32.497	0.780	0.021
**AHNAK**	-0.288	0.052	-0.300	0.784	0.657	0.092	-0.644	0.727	0.976	0.021
**AIF1**	-0.188	44.812	-0.089	0.264	-0.106	29.328	0.451	0.061	0.841	0.021
**AIFM2**	0.377	44.812	0.427	0.354			-0.215	0.947	0.220	24.398
**AIM1**	-0.508	0.052			-0.142	54.331	-0.171	52.656	0.517	11.001
**AK2**	-0.221	7.743	-0.301	2.123	0.213	49.127	-0.481	0.855	-0.853	0.066
**AKAP10**	0.321	0.126	0.594	0.595	0.387	2.659	0.146	23.450	-0.427	0.045
**AKAP8**	-0.036	55.559	0.321	3.009	-0.043	58.928	-0.570	0.506	0.229	24.398
**AKAP9**	0.074	44.812	0.222	2.773	0.133	20.025	-0.197	2.913	-0.571	0.021
**AKR1B10**	0.092	49.540	-0.384	2.123	-0.260	0.240	0.747	0.399	0.951	0.021
**AKT3**	0.114	37.036	0.266	5.829			0.619	0.061	-0.296	0.471
**ALAS2**	-0.192	4.284	-0.408	0.944	-0.164	22.899			0.625	0.119
**ALDH16A1**	-0.061	57.109	-0.650	0.514	0.749	0.092	-0.032	49.358	0.555	0.119
**ALDH1A1**	-0.146	55.870	-0.116	4.122	0.256	0.402	0.115	32.497	0.425	0.993
**ALDH1A2**	-0.637	0.052			-0.220	49.127			0.383	4.218
**ALDH1B1**	0.137	5.928	0.145	13.299			-0.184	13.893	-0.519	0.021
**ALDH1L2**	0.056	44.812	-0.380	0.859	0.118	33.124	-0.242	19.971	0.370	24.398
**ALDH3B1**	-0.174	37.036	-0.230	2.859	0.647	0.724	0.294	0.727	0.646	0.269
**ALDOC**	-0.040	49.540	-0.512	4.122	0.217	20.025	0.111	23.450	0.587	0.021
**ALOX12B**	0.072	54.154	-0.283	0.784	0.471	0.092	0.166	1.332	0.654	0.391
**ALPK3**	0.374	0.052	0.152	6.703	0.366	5.705	-0.505	27.828	-0.344	0.039
**ALS2**	0.274	0.171	-0.423	2.773	-0.164	14.770	-0.686	0.250	-0.336	0.119
**AMHR2**	0.604	0.126	0.299	5.829	0.519	3.068	-0.495	32.497	0.007	44.052
**AMIGO2**	-0.430	12.647	0.326	1.029	-1.051	0.724			-0.577	24.398
**AMPH**	-0.604	0.052			-0.372	0.092	0.056	47.420	0.093	44.052
**AMZ1**	-0.329	9.997	0.094	9.629	0.059	54.331	0.657	0.061	0.523	0.649
**ANAPC16**	0.019	56.130	0.230	5.829	0.031	58.074	-0.558	0.061	-0.446	0.471
**ANGPTL4**	0.091	44.812	-0.197	8.881	0.169	49.127	0.211	0.727	0.610	0.039
**ANK1**	0.441	0.171	0.253	4.122	-0.219	9.255	0.430	0.061	0.234	14.822
**ANK2**	-1.015	0.052	-0.467	6.703	-0.661	0.092	-1.733	0.061	-0.439	0.993
**ANKRD11**	-0.585	0.052	-0.348	2.773	-0.656	0.092	-0.374	11.241	0.431	0.649
**ANKRD26**	0.547	5.928	0.244	0.986	-0.640	0.092	-0.687	0.061	-1.114	0.021
**ANKRD44**	0.073	52.827	0.390	1.772	0.123	36.892			0.952	0.021
**ANTXR1**			0.561	0.986	0.098	33.124	-0.538	1.210	0.493	0.993
**ANTXR2**					-0.508	0.819	-0.075	41.825	-0.059	41.878
**ANXA2**	-0.081	29.668	0.282	8.881	0.290	1.309	-0.349	0.727	0.692	0.021
**ANXA3**	-0.332	12.647	0.404	1.772	0.395	0.440	0.181	41.825	1.000	0.021
**ANXA4**	-0.291	0.171	-0.240	4.122	0.116	36.892	-0.100	47.420	0.511	0.977
**ANXA5**	-0.234	5.928	0.331	1.212	0.154	17.386	-0.054	51.354	0.561	0.269
**ANXA6**	-0.537	0.052	0.444	2.123	-0.202	33.124	0.170	49.358	-0.430	0.993
**ANXA7**	0.151	15.278	0.455	5.829	-0.263	14.770	-0.243	0.177	-0.530	0.021
**ANXA8/ANXA8L1**	-0.193	0.666	-0.716	0.428	-0.061	49.127	0.058	51.354	0.199	24.398
**AOC2**	0.317	25.771	0.194	13.299	0.204	12.575			-0.676	0.021
**AP1G1**	-0.829	0.170	0.404	1.457	0.196	17.386	-0.163	1.332	-0.619	0.021
**AP3M1**	0.208	2.450	-0.402	2.123	-0.172	5.705	0.230	1.332	-0.533	0.066
**AP3M2**	-0.172	49.540	0.321	1.663	-0.242	36.892	-0.128	41.825	-0.579	0.021
**AP3S1**	0.141	44.812	0.653	0.420	-0.094	49.127	-0.048	51.354	-0.333	0.021
**APBA1**	-0.468	0.052	-0.602	0.514	0.449	0.092	0.173	49.358	0.179	24.398
**APBB2**	-0.562	0.052	-0.093	11.783	-0.438	1.819	-0.185	0.573	-0.527	0.213
**APH1B**	0.086	51.013	0.364	2.614	0.047	58.074	0.233	8.987	-0.401	0.993
**APLF**	0.301	0.666	0.346	1.534	0.122	49.127	0.663	0.061	0.319	11.001
**APLN**	-0.302	1.431	-0.332	3.205	0.040	49.127	-0.400	0.061	-1.109	0.021
**APLNR**	0.013	55.559	-0.242	2.859	0.393	0.092			-0.183	24.398
**APOBEC1**	-0.365	21.797	-0.326	6.703	-0.224	26.120	-0.057	53.272	0.839	0.066
**APOC1**			-0.242	4.122	0.078	49.127	-0.047	47.420	0.784	0.021
**APOC2**	0.088	44.812	-0.135	6.703	0.020	58.611			0.536	0.269
**APOC3**	-0.267	44.812	0.291	0.944	-0.250	2.056	0.976	0.130	0.866	0.021
**APOD**	-0.461	2.450	0.542	1.663	0.099	41.103			0.603	0.021
**APOE**			0.180	6.703	0.091	49.127	0.088	47.420	0.620	0.021
**APOL2**	0.134	12.647	-0.537	0.204	0.067	41.103	0.120	41.825	0.507	0.891
**APP**	-0.480	0.052	0.507	4.122	-0.402	3.475	0.369	19.971	-0.850	0.021
**AQP4**	-0.325	0.052	0.533	0.300	0.154	10.694	0.197	0.399	0.697	0.021
**ARAP2**	0.119	15.278	0.662	1.534			-0.130	19.971	-0.575	0.045
**ARAP3**	0.273	0.324	0.484	0.595	-0.024	60.505	0.184	8.987	0.435	0.119

**ARC**	0.583	0.052	0.311	0.569	0.271	1.098	-0.052	52.656	0.080	36.831
**ARF3**	0.154	37.036	0.714	0.264	0.015	59.432	-0.042	47.420	-0.381	0.045
**ARFGEF2**	0.259	1.431	0.577	1.029	-0.124	9.255	0.316	5.543	-0.317	0.021
**ARHGAP24**	-0.369	0.171	-0.899	2.614	-0.295	0.240	-0.274	27.828	-0.218	11.001
**ARHGAP26**	-0.281	44.812	0.410	0.107	0.176	22.899	-1.234	0.061	-0.504	24.398
**ARHGAP4**	-0.098	52.218	-0.568	0.772	0.283	20.025	-0.343	23.450	-0.067	38.751
**ARHGAP42**	-0.208	2.450	-0.522	0.107	-0.511	0.092	0.130	32.497	-0.646	0.021
**ARHGAP44**	0.069	44.812	0.067	8.253	-0.146	22.899	-0.143	8.987	-0.548	0.021
**ARHGAP5**	-0.097	44.812	0.727	0.514	0.041	59.793	0.770	0.250	-0.913	0.084
**ARHGAP6**	-0.555	0.052	0.200	2.614	0.266	8.121	-0.564	0.399	0.478	24.398
**ARHGAP9**	-0.081	52.827	-0.219	2.123	0.161	29.328	-0.031	53.272	0.521	0.021
**ARHGDIB**	0.132	49.540	0.129	2.614	0.021	59.793			0.728	0.084
**ARHGEF2**	0.502	0.930	0.367	7.537	0.212	1.556	0.306	5.543	0.178	37.824
**ARHGEF3**	0.576	7.743	0.687	0.420	0.041	56.823	0.091	41.825	-0.190	4.218
**ARHGEF6**	-0.620	0.052	0.645	1.289	-0.486	0.856	0.527	19.971	-0.460	0.746
**ARID4A**	0.355	4.284	0.556	0.944	-0.169	54.331	-0.388	0.399	-0.500	0.021
**ARL1**	0.068	49.540	0.707	0.354			-0.107	41.825	-0.334	0.039
**ARL4C**	0.061	44.812	0.159	2.773	0.060	54.331	0.403	0.292	0.439	0.891
**ARL4D**	0.384	0.529	0.435	0.514	0.152	8.121	0.082	32.497	-0.054	31.624
**ARL6**					-0.094	17.386	0.052	47.420	-0.507	0.021
**ARMC9**	0.140	21.797	0.405	0.669	0.249	33.124	0.372	0.341	0.216	33.879
**ARPC1B**	-0.080	44.812	-0.316	5.829	0.048	49.127	0.145	47.420	0.893	0.021
**ARPP19**	0.268	0.511	0.777	0.569	0.237	3.068	0.140	2.913	0.460	0.084
**ARPP21**	-0.257	44.812	-0.206	0.784	0.341	22.899	-0.148	5.543	-0.441	0.129
**ARRB2**	0.069	37.036	-0.380	0.784			0.303	2.415	0.400	0.021
**ASAH2**	-0.268	0.666	0.397	1.663	0.026	58.928	-0.419	0.353	-0.668	0.021
**ASAP2**	0.023	55.170	0.667	0.514	0.060	49.127	0.041	54.831	-0.150	1.977
**ASB11**	-0.216	0.930	0.278	1.772	-0.369	4.053	-0.076	52.124	-0.678	0.021
**ASPA**	-0.452	0.262	-0.642	2.859	-0.113	26.120	-0.125	52.124	-0.265	24.398
**ASPG**	-0.113	15.278	-0.231	1.534	0.342	0.092	0.292	11.241	1.214	0.021
**ASPH**	-0.335	0.529	0.557	2.859	-0.281	9.255	0.595	0.399	-0.408	0.119
**ASXL2**	0.139	44.812	0.415	3.205	-0.431	0.092	-0.994	1.332	-0.230	6.001
**ATAD2**	0.285	33.231	0.648	0.445	-0.126	55.771	0.128	11.241	0.251	0.746
**ATAD5**	-0.356	0.052	-0.197	0.514	-0.240	17.386	-0.098	41.825	-0.632	0.119
**ATAT1**	0.211	29.668	0.129	6.703	0.290	0.641	-0.447	13.893	0.466	0.021
**ATE1**	0.068	51.013	0.205	0.514	0.602	0.440	-0.451	4.906	-0.438	0.891
**ATF1**	0.210	33.231	0.677	0.514	0.112	49.127	-0.284	5.543	0.817	0.021
**ATF2**	-0.607	0.511	0.593	0.944	-0.403	8.121	0.446	0.061	-0.917	0.021
**ATF5**	0.021	54.701	0.386	1.772	-0.021	59.432	0.039	51.354	0.509	0.039
**ATG10**	-0.129	33.231	-0.293	5.829	-0.224	2.311	0.202	4.906	-0.516	0.021
**ATIC**	-0.234	51.013	-1.479	0.569	-0.524	0.240	-0.106	51.354	0.168	36.831
**ATL2**	0.204	2.450	0.420	0.457	0.402	3.068	0.297	4.906	0.080	39.468
**ATM**	0.390	0.511	-0.274	4.122	0.064	49.127	-0.178	27.828	-0.525	0.021
**ATMIN**	0.171	37.036	-0.358	2.300	0.088	26.120	0.131	41.825	0.706	0.021
**ATN1**	0.020	55.170	-0.390	5.829	0.129	49.127	0.183	41.825	0.589	0.021
**ATOX1**	0.036	49.540	-0.158	5.829	-0.203	1.819	-0.698	0.061	0.167	31.624
**ATP13A3**	-1.216	0.052	0.815	0.514	-0.096	54.331	0.072	52.656	0.425	24.398
**ATP1A1**	-0.076	49.540	0.129	11.783	0.285	4.053	0.143	47.420	0.529	0.649
**ATP2A1**	0.233	44.812	0.177	8.881	0.624	0.092	-0.785	0.061	0.710	0.993
**ATP2B1**	-0.141	9.997	0.692	1.289	0.170	12.575	-1.063	3.866	-0.730	0.045
**ATP2C1**	-0.464	0.171	0.226	4.122	-0.431	0.240	0.490	16.936	-0.623	0.021
**ATP2C2**			0.287	1.534	-0.258	41.103	0.773	0.292	-0.083	40.051
**ATP4A**	-0.053	56.874	0.085	12.522	0.662	4.733	-0.365	0.727	-0.400	0.746
**ATP5F1**	0.013	57.109	0.465	0.595	-0.139	3.068	0.078	47.420	0.383	33.879
**ATP6V0A2**	0.137	15.278	0.421	1.534	0.082	33.124	0.272	0.947	0.438	0.021
**ATP8A1**	-0.259	0.126	0.738	0.669	-0.582	0.517	-0.090	23.450	-0.540	0.993
**ATRNL1**	-0.521	0.052	0.543	0.267	-0.177	49.127	-0.337	1.210	0.395	0.993
**AUH**	0.455	0.052	-0.436	0.784	0.671	1.556	-0.841	0.947	0.548	0.021
**AUTS2**	0.090	37.036	-0.378	0.514	0.098	41.103	0.249	7.099	-0.391	0.649
**B2M**	-0.116	18.587	-0.436	3.205	0.120	49.127	-0.100	41.825	0.902	0.021
**B3GALNT1**	0.400	9.997	-0.412	0.569	0.041	55.771	-0.116	32.497	-0.154	24.398
**B3GALT2**	0.210	18.587	0.185	6.703	-0.195	1.819	0.486	0.913	0.256	33.879
**B3GAT1**	0.341	0.666	0.454	0.514	-0.074	55.771	-0.099	11.241	-0.155	24.398
**B3GNT2**	0.320	33.231	0.705	0.264	0.227	3.068	-0.042	50.493	-0.188	1.977
**BACE2**	0.795	0.324	0.065	8.881	-0.638	0.092	-0.869	0.399	0.621	0.021
**BACH1**	-0.353	0.780	-0.593	1.289	-0.405	5.705	0.234	32.497	-0.322	4.218
**BACH2**	-0.307	0.126			-0.148	14.770	-0.319	11.241	-0.563	0.021
**BAD**	-0.228	12.647	0.428	0.514	0.468	0.092	0.548	13.893	0.404	0.993

**BAG4**			0.688	0.877	0.091	29.328	0.140	47.420	-0.775	0.119
**BAI3**	0.209	9.997	0.686	0.514	0.207	5.705	-0.256	0.177	-0.620	0.045
**BATF**	-0.379	2.450	-0.191	2.300	-0.132	41.103	-0.705	1.878	0.696	0.021
**BATF3**			-0.133	0.784	0.202	36.892	0.216	32.497	0.443	0.601
**BAZ2A**	-0.115	49.540	-0.197	1.534	-0.103	58.406	-0.406	0.292	-0.064	33.879
**BBC3**	0.044	52.827	0.146	4.122	-0.229	12.575	0.170	32.497	0.583	0.021
**BBIP1**	0.057	44.812	0.385	5.829	-0.162	29.328	-0.059	47.420	-0.564	0.021
**BBS1**	0.663	0.052	0.589	0.602	-0.227	49.127	-1.179	0.061	-0.637	0.269
**BBS5**	0.033	54.701	0.646	0.595	-0.076	49.127	0.254	7.099	-0.358	0.993
**BCKDHB**	0.094	44.812	0.217	0.514					-0.411	0.021
**BCL11A**	-0.211	44.812	-0.379	3.009	-0.517	0.998	0.102	41.825	0.366	1.977
**BCL2**	0.164	44.812	0.531	0.929	0.223	6.932	0.342	11.241	0.397	0.213
**BCL2A1**	0.322	1.098	0.354	0.986	0.011	60.505			1.952	0.021
**BCL2L2**	-0.238	49.540	0.248	4.122	0.452	1.098	0.427	0.250	0.510	0.021
**BCL3**					-0.033	60.231	0.140	5.543	0.598	0.021
**BCLAF1**	-0.767	0.052	0.532	1.663	-0.399	10.694	-0.480	23.450	0.499	24.398
**BDNF**	0.181	0.780	0.866	0.669	0.225	2.056	-0.118	19.971	-0.942	0.021
**BDP1**	-0.198	0.666	-0.816	0.935	-0.102	54.331	-0.791	1.878	0.413	0.993
**BECN1**	0.185	12.647	0.537	1.086	0.032	58.074	0.100	47.420	-0.417	0.021
**BEND3**	-0.256	0.529	-0.425	3.205	0.059	54.331	0.431	0.130	0.088	39.468
**BEND6**	0.088	21.797	0.405	5.829	0.012	59.793	0.196	2.415	-1.175	0.021
**BFSP2**	-0.217	0.171	-0.286	8.253	0.343	3.068	0.175	19.971	0.926	0.021
**BHLHE22**	0.116	25.771	-0.138	4.122	0.345	1.309	0.387	4.906	0.603	0.021
**BHLHE22**	-0.402	0.930	1.110	1.029	-0.205	14.770	-0.139	41.825	-0.551	4.218
**BICD2**	-0.094	37.036	0.734	0.595	0.215	54.331	0.241	23.450	-0.371	0.119
**BIN1**	-0.508	0.052	0.499	2.859	-0.110	26.120	-0.486	32.497	-0.405	0.977
**BIRC5**	0.040	53.690	-0.134	8.881	0.296	0.659	-0.033	52.124	0.523	0.269
**BIRC6**	-0.246	0.666	-0.419	0.223	0.067	54.331			-0.025	39.468
**BLCAP**			-0.217	5.829	0.094	58.074	0.409	41.825	0.435	0.977
**BLNK**	-0.239	0.511	-0.161	6.703	-0.172	49.127	-0.196	23.450	0.489	0.891
**BLVRB**	0.212	5.928			0.093	33.124	0.055	49.358	0.560	0.119
**BLZF1**	0.087	51.013	-0.318	0.354	0.313	0.440	-0.310	0.947	-0.653	0.021
**BMF**	-0.075	33.231	-0.727	3.205	-0.046	56.823	-0.588	1.878	1.246	0.021
**BMP2K**	-0.507	0.052	-0.967	0.514	0.123	54.331	0.521	23.450	0.857	0.021
**BMP6**	-0.306	21.797	-0.122	0.784	0.464	0.092	-0.535	0.061	0.084	35.531
**BMP7**	-0.301	5.928			0.505	3.068	0.409	0.332	0.231	6.001
**BMPER**	0.130	44.812	-0.284	8.253	-0.388	41.103	0.370	47.420	-1.108	0.021
**BMPR2**	-0.661	0.052	0.377	1.663	-0.635	0.856	-0.275	0.109	-0.214	8.002
**Bnip3**	0.043	54.154	0.350	2.614	-0.136	22.899	-0.057	47.420	-0.592	0.021
**BPNT1**	0.087	49.540	0.403	0.595	0.130	49.127	0.074	49.358	0.212	24.398
**BRCA1**	0.450	0.324			0.194	41.103	-0.457	2.415	-0.142	33.879
**BRDT**	0.247	3.157	0.505	0.859	0.112	22.899	0.208	41.825	-0.088	33.879
**BTAF1**			0.619	4.122	0.089	20.025			-0.415	0.039
**BTBD3**	0.057	51.013	0.287	11.783	-0.205	26.120	-0.334	0.250	-1.074	0.021
**BTBD9**	0.192	33.231	0.217	1.663	0.105	17.386	0.433	1.878	-0.867	0.307
**BTG2**	-0.626	0.052	0.500	0.180	-0.355	26.120	0.559	13.893	0.206	36.831
**BTK**	-0.487	2.450	-0.499	0.595	-0.184	3.068	0.849	2.913	1.095	0.021
**BUB3**	-0.945	0.171	0.253	0.514	-0.409	49.127	-0.421	11.241	0.151	11.001
**BYSL**	0.264	2.450	-0.310	0.514	0.082	33.124	0.171	19.971	0.469	0.021
**BZW1**	0.289	4.284	0.574	0.784	-0.285	2.311	-0.211	2.913	-0.448	0.021
**C10orf54**	-0.191	44.812	0.145	3.009	-0.143	54.331	0.288	41.825	0.705	0.021
**C10orf81**	0.530	0.262	0.365	2.123	0.423	8.121	0.060	47.420	0.656	0.021
**C12orf35**	-0.204	0.666	0.370	0.784	-0.416	0.092	0.070	47.420	0.096	31.624
**C12orf5**	0.146	44.812	0.352	1.086	-0.302	2.311	0.493	0.061	-0.537	0.021
**C12orf51**	-0.595	0.262	-0.228	9.629	-0.606	0.092	0.245	1.210	0.598	0.045
**C16orf45**	-0.474	0.171	-0.123	4.122	-0.480	0.281	0.126	41.825	0.174	8.002
**C16orf62**	0.503	0.284	0.187	6.703	0.231	4.733	0.114	27.828	0.222	14.822
**C16orf74**	0.059	49.540			0.198	2.056	-0.088	47.420	0.424	0.193
**C16orf88**	0.691	0.052	0.276	1.029	0.067	57.395	0.597	0.341	0.211	14.822
**C17orf59**	0.264	0.262	0.211	8.253	0.270	17.386	0.531	19.971	-0.801	0.021
**C17orf70**	0.331	0.262	-0.134	2.859	0.049	54.331	-0.510	0.061	-0.617	0.021
**C19orf12**	0.030	54.701	0.131	0.514	0.088	41.103	0.085	41.825	0.482	0.084
**C1GALT1**	0.207	0.930	0.659	0.514	-0.063	49.127	-0.184	23.450	-0.424	0.119
**C1orf159**	0.058	54.154	0.480	1.772	0.077	41.103	0.196	41.825	-0.484	0.021
**C1orf168**	0.059	53.690	0.085	6.703	-0.354	33.124	-0.551	5.543	-0.722	0.649
**C1orf190**	0.314	0.126	0.420	1.663	0.061	54.331	0.511	0.061	0.457	0.119
**C1orf38**	0.059	49.540	-0.290	0.877	0.406	0.517	-0.136	13.893	1.118	0.021
**C1QA**	0.047	56.130			0.116	5.705	-0.074	27.828	1.066	0.021

**C1QB**			-0.340	2.859	0.059	49.127	0.086	32.497	0.870	0.021
**C1QC**	0.023	56.513			0.133	9.255	0.255	1.878	1.141	0.021
**C1QTNF5**	-0.298	0.529	-0.236	5.829	0.138	12.575	-0.180	1.617	0.462	0.129
**C20orf194**	0.575	0.780	0.499	2.614	0.389	8.121	-0.508	8.987	0.892	0.993
**C20orf94**	-0.050	52.827	0.248	2.123	0.110	49.127	-0.316	1.878	0.576	0.021
**C21orf91**	-0.296	52.218	-0.659	1.663	-0.223	9.255	0.907	0.061	-0.567	0.891
**C3**	-0.152	37.036	-0.134	5.829	-0.086	41.103	0.052	47.420	1.218	0.021
**C3orf67**	-0.436	0.398	-0.254	2.614	0.088	49.127	0.061	47.420	0.653	0.021
**C4B**	0.058	51.013	0.276	1.772	0.254	2.311	0.539	0.855	0.595	0.021
**C4orf19**	0.227	21.797	-0.402	0.986	-0.079	55.771	0.242	47.420	0.688	0.021
**C4orf33**	0.355	0.398	0.133	5.829	-0.329	3.475	0.436	0.177	0.400	0.045
**C4orf43**	0.278	25.771	0.527	0.300	0.041	54.331	0.152	19.971	-0.236	0.746
**C6orf222**	0.107	44.812	0.196	2.773	0.540	0.819	0.366	4.906	0.113	40.051
**C6orf35**	0.231	1.098	0.345	6.703	0.433	3.068	-0.319	32.497	-0.789	0.039
**C7orf36**	-0.545	0.171	0.797	1.212	-0.273	12.575	-1.584	0.061	-0.449	0.021
**C7orf57**	0.165	0.666	-0.127	4.122	-0.259	36.892	-0.551	0.121	0.214	33.879
**C7orf60**	0.069	49.540	0.755	0.514	0.073	36.892	-0.222	19.971	-0.502	0.391
**C8orf34**	-0.047	49.540	-0.201	0.784	-0.170	33.124	-0.447	0.061	-0.607	0.021
**C8orf4**	0.103	44.812	-0.378	3.205	-0.315	5.705	0.215	41.825	0.666	0.213
**C9orf71**	0.189	0.780	-0.231	3.205	0.200	17.386	0.164	47.420	0.716	0.021
**CA13**	0.567	0.170	0.870	0.107					-0.097	38.751
**CA5B**	-0.301	9.997			0.226	3.068	-0.367	4.906	0.503	0.391
**CAB39**	0.145	37.036	0.621	2.123	-0.126	36.892			-0.326	0.021
**CACNA1B**	0.084	49.540	0.575	0.986	0.158	3.068	0.321	0.130	-0.380	0.269
**CACNA1C**	0.165	5.928	0.332	0.986	-0.269	22.899	0.689	1.332	0.765	0.066
**CACNA1E**	-0.305	18.587	-0.315	5.829	0.121	14.770			-0.429	0.269
**CACNA2D1**	-0.545	0.398	0.403	5.829	-0.372	1.309	-0.826	0.109	-0.264	1.977
**CACNG2**	0.245	4.284	-0.478	0.514	0.359	3.475	-0.724	0.399	0.661	0.307
**CACNG5**	-0.709	0.052			-0.642	10.694			-0.476	24.398
**CADPS2**	-0.200	5.928	0.856	0.986	-0.115	33.124	0.383	11.241	0.421	0.471
**CALB2**	-0.368	0.171	-0.447	0.180	-0.508	0.281	0.471	0.121	0.395	0.391
**CALCRL**	-0.521	0.052	-0.226	4.122	-0.481	0.092	0.364	41.825	-0.263	24.398
**Cald1**	-0.168	21.797	-0.092	11.783	-0.344	3.068	-0.291	19.971	-0.382	0.746
**Calm1**	0.088	44.812	0.502	0.300	-0.136	33.124	-0.154	1.332	-0.493	0.084
**CAMK1**	0.087	12.647	0.494	0.829	0.278	0.724	-0.369	27.828	0.315	6.001
**CAMK2D**	-0.416	3.157	0.367	3.009	0.995	0.092	0.271	0.121	0.298	31.624
**CAMK4**	-0.201	44.812	0.270	0.445	-0.508	0.092	-0.591	0.353	-0.754	0.039
**CAMKMT**	-0.056	57.109	0.196	11.783	-0.284	3.068	-0.416	0.109	-0.163	37.824
**CAMTA1**	0.045	49.540	0.101	7.537	-0.069	58.074	-0.146	5.543	-0.436	0.891
**CANT1**	0.115	37.036	-0.514	0.223	0.160	6.932	0.145	47.420	0.414	24.398
**CAP1**	-0.289	0.170	-0.577	0.986	-0.248	22.899	-0.166	11.241	-0.492	0.021
**CAPG**	-0.077	57.367	-0.164	2.859	-0.108	33.124	0.608	0.061	0.860	0.021
**CAPN3**	0.317	21.797	0.440	0.663	0.035	58.074	-0.032	53.272	0.382	4.218
**CAPSL**	-0.313	0.511	0.326	0.174	0.130	58.406	0.269	47.420	-0.520	0.021
**CARD6**	-0.403	2.450			-0.167	54.331	0.606	0.061	-0.235	0.391
**CARKD**	-0.436	0.052	0.204	5.829	-0.290	4.733	-0.218	7.099	-0.286	0.649
**CARTPT**	-0.628	0.780	-0.148	4.122	0.191	12.575	-0.177	2.415	-0.328	24.398
**CASP12**	0.117	44.812	-0.148	4.122	0.113	49.127	-0.111	27.828	0.678	0.021
**CASP8**	0.115	51.013			-0.050	54.331	-0.293	19.971	0.433	0.901
**CAV1**	-0.182	4.284	-0.433	2.773	0.118	33.124	-0.152	23.450	0.473	0.307
**CAV2**	0.455	1.098	0.455	0.445	0.477	4.053	0.259	47.420	0.566	0.021
**CBLN1**	-0.352	0.398	-0.102	4.122	0.292	0.240	0.338	0.573	0.464	0.021
**CBX3**	-0.441	0.398	-0.582	2.123	-0.463	0.092	0.255	49.358	0.418	0.021
**CBX8**	0.166	12.647	-0.370	5.829	0.194	0.856	-0.322	47.420	0.688	0.045
**CCBE1**	-0.548	0.171	0.211	5.829	0.027	58.406	0.227	11.241	-1.500	0.021
**CCBL1**	0.107	37.036	-0.449	0.935	0.031	56.823	-0.044	49.358	0.124	37.824
**CCDC102A**			0.108	5.829	-0.403	0.856	-0.097	47.420	0.926	0.021
**CCDC109B**	0.037	54.154	0.451	0.122	0.107	36.892	0.071	41.825	0.516	31.624
**CCDC19**	-0.415	9.997	0.295	0.602	-0.139	9.255	0.371	2.913	-0.402	0.891
**CCDC25**	-0.543	0.052	0.695	1.457	-0.342	1.098	0.274	2.415	0.249	24.398
**CCDC64**	0.280	33.231	-0.098	3.205	0.051	54.331	-0.492	0.947	-0.246	1.193
**CCDC80**	-0.150	1.098	-0.103	10.404	0.324	0.659	-0.134	52.124	0.628	0.021
**CCDC85A**	-0.587	0.511	-0.875	1.086	-0.272	2.659	0.615	11.241	-0.369	0.993
**CCDC88A**	-0.484	0.052	-0.687	2.123	-0.382	6.932	-0.254	32.497	-0.555	0.021
**CCHCR1**	0.255	33.231			0.212	2.056	0.455	0.177	-0.192	31.624
**CCKBR**	-0.476	0.052	-0.128	12.522	0.365	0.659			-0.588	0.891
**CCL13**	-0.378	4.284	0.175	4.122	0.247	2.659	0.116	41.825	1.205	0.021
**CCL2**	-0.526	0.093	-0.357	2.773	-0.425	1.819	0.196	23.450	1.683	0.021

**CCL3**									1.616	0.021
**CCL4**	-0.277	37.036	-0.946	1.772	0.316	2.659	0.231	52.656	1.491	0.021
**CCL5**	-0.275	18.587	-0.125	5.829	-0.343	20.025	-0.081	47.420	0.595	0.021
**Ccl6**					-0.216	2.659	0.700	0.353	1.754	0.021
**Ccl9**	-0.167	37.036	-0.346	2.123	-0.318	0.440	0.120	41.825	1.317	0.021
**CCNB1IP1**	-0.262	52.218			0.266	6.932	0.203	47.420	0.605	0.021
**CCND1**	0.051	52.218	0.310	3.205	0.231	1.819	0.215	0.353	0.702	0.129
**CCND2**	0.412	0.052	0.451	1.457	-0.262	33.124			0.051	42.212
**CCNG1**	0.073	44.812	0.752	0.986	0.108	54.331	0.251	0.250	-0.307	2.886
**CCNI**	-0.425	0.052	0.258	0.514	-0.212	10.694	0.425	41.825	0.313	0.649
**CCNJL**	-0.154	29.668	0.336	3.009	-0.324	2.311	-0.242	8.987	-0.674	0.993
**CCNL1**	-0.538	0.052	0.648	2.614	-0.071	41.103	0.743	0.061	0.211	24.398
**CCNL2**	0.410	0.930	0.605	0.602	-0.087	41.103	-0.244	0.727	-0.364	0.119
**CCR5**	-0.076	44.812	0.405	0.445					-0.720	0.021
**CCR6**	0.151	1.936	-0.209	3.009			-0.468	0.061	0.106	31.624
**CCR9**	-0.056	44.812	-0.403	0.986	0.035	56.823			-0.189	24.398
**CCRL2**	-0.153	3.157	0.320	0.514	-0.249	3.068	1.121	0.061	1.139	0.021
**CD14**	0.162	21.797	-0.101	6.703			-0.493	0.061	1.171	0.021
**CD1D**	0.434	21.797	0.936	0.107	0.054	55.771	-0.229	51.354	0.543	0.977
**CD248**	0.067	52.218	-0.110	12.522	0.143	17.386	0.431	0.506	0.256	0.891
**CD300A**	0.243	1.098	-0.520	0.595	-0.369	1.556	0.847	0.947	1.242	0.021
**CD37**	-0.075	44.812	-0.588	0.514	0.210	17.386	0.105	49.358	0.742	0.021
**CD44**	-0.198	54.701	0.185	3.205	-0.428	0.092	-0.554	1.878	1.940	0.021
**CD48**	-0.106	57.293	0.073	13.299	0.420	0.856	-0.877	0.250	1.419	0.021
**CD52**	-0.088	49.540			0.189	49.127			1.200	0.021
**CD59**	0.595	0.171			0.401	0.856	-0.702	0.061	-0.954	0.021
**CD5L**	-0.086	44.812	-0.298	4.122	-0.064	54.331	-0.507	2.415	0.929	0.129
**CD6**	0.116	37.036	-0.223	3.205	0.488	0.440	-0.064	41.825	-0.155	24.398
**CD63**	-0.186	7.743	0.297	0.602	0.069	33.124	-0.079	49.358	0.686	0.021
**CD68**	-0.170	49.540	-0.101	7.537	0.312	2.056	0.134	8.987	1.205	0.021
**CD74**	-0.138	44.812	-0.361	0.569	0.177	2.311	0.025	55.172	0.879	0.021
**CD79B**			0.239	0.514					0.661	0.021
**CD82**	0.192	12.647			0.118	4.053	0.394	0.061	0.541	0.649
**CD86**			-0.125	8.881	0.406	0.641	0.252	19.971	1.012	0.021
**CD9**	-0.294	0.171	0.426	1.086	-0.120	49.127	0.425	11.241	1.074	0.021
**Cd99**	-0.174	1.098	-0.374	3.009	-0.132	54.331	0.213	11.241	0.435	0.021
**CDAN1**	-0.066	44.812	0.283	2.614	0.032	57.395	-0.084	19.971	0.778	0.021
**CDC20**	-0.310	2.450	-0.894	2.123	-0.090	49.127			0.607	0.993
**CDC40**	-0.918	0.052	0.471	0.595	-0.241	0.641	-0.920	0.061	-0.471	0.045
**CDC42**	0.078	44.812	0.643	0.514	-0.152	22.899	-0.174	19.971	-0.431	0.021
**CDC42BPA**	0.164	44.812	-0.293	2.773	0.170	12.575	-0.086	41.825	0.587	0.307
**CDC42EP1**	-0.151	37.036	-0.191	0.784	0.032	55.771	0.322	16.936	0.598	0.039
**CDC42EP3**	-0.735	0.093	0.361	4.122	-0.189	49.127	0.082	54.831	0.452	41.258
**CDC42EP4**	-0.267	37.036	-0.466	1.534	0.118	33.124	0.274	1.210	0.962	0.021
**CDC42EP5**	0.035	53.690			-0.271	5.705	0.362	2.415	0.438	0.891
**CDC6**	-0.087	56.130	0.506	0.484	0.560	5.705	0.554	2.415	-0.516	0.746
**CDCA4**	-0.041	52.218	0.224	9.629			-0.262	0.177	-0.354	0.993
**Cdca7**	0.107	44.812	0.237	6.703	-0.047	60.505	-0.542	0.061	-0.280	11.001
**CDH11**	-0.532	0.529	-1.000	1.029	-0.063	49.127	0.332	23.450	-0.435	0.021
**CDH20**	0.149	2.450	0.294	7.537	0.153	6.932	0.287	11.241	0.486	0.893
**CDH7**	-0.635	0.171	0.117	9.629	0.530	9.255	-0.399	7.099	-0.411	0.039
**CDK11A/CDK11B**	0.142	21.797	0.398	2.773	0.069	41.103	0.083	41.825	0.307	6.001
**CDK12**	-0.345	0.262	0.452	1.772	0.073	41.103	0.203	1.210	0.107	40.051
**CDK18**	-0.119	44.812	0.123	7.537	-0.094	58.074	0.317	0.061		
**CDK6**	-0.394	0.093	-0.173	5.829	-0.064	55.771	-0.144	27.828	-0.113	31.624
**CDKL5**	-0.305	0.529	-0.573	0.445	-0.547	0.092	0.124	47.420	0.071	42.369
**CDKN1C**	-0.616	0.529			0.140	54.331	-0.798	0.061	0.088	33.879
**CDKN2AIP**	0.266	0.511	-1.228	1.772	-0.447	5.705	-0.716	4.906	-0.397	0.993
**CDS2**	-0.159	44.812	0.545	1.289	0.106	54.331	0.461	0.061	-0.326	0.129
**CEBPA**			0.296	0.300	0.127	22.899	0.205	23.450	0.582	0.021
**CEBPB**	0.323	33.231	0.127	11.093	0.324	0.641	0.173	11.241	0.671	0.066
**CECR6**	0.528	0.052	0.384	0.514	0.365	0.402	0.221	19.971	-0.294	0.746
**CELF2**	-0.446	0.052	0.400	0.514	0.055	57.395	-0.340	0.855	-0.425	0.166
**CELSR3**	0.055	53.690	0.681	0.484	0.168	33.124	0.442	7.099	1.059	0.021
**CENPA**	0.101	49.540	-0.433	3.009	0.099	36.892	-0.089	49.358	0.748	0.021
**CENPE**	-0.089	49.540	-0.316	6.703	-0.845	0.092	0.378	47.420	-1.044	0.391
**CENPM**	-0.181	44.812	0.335	0.514	-0.053	54.331	0.807	0.061	-0.665	0.021
**CENTG2**	-0.132	15.278	0.261	1.772	-0.249	12.575	0.201	3.866	1.089	0.021

**CEP120**	0.137	33.231	-0.554	0.784	0.088	57.395	-0.179	8.987	-0.993	0.021
**CEP152**			-0.180	6.703	-0.098	54.331	-0.223	47.420	-0.788	0.021
**CEP27**	-0.559	0.780	0.366	0.569	-0.469	0.281	-0.401	2.415	-0.898	0.021
**CEP55**	-0.929	0.052	-0.154	4.122	0.832	0.092	0.183	27.828	-0.857	0.021
**CES5A**	-0.080	51.013	0.188	4.122	0.413	5.705			0.834	0.021
**CES7**	-0.324	7.743	-0.888	1.086	0.436	0.517	0.316	1.210	0.677	0.993
**CFI**	0.078	33.231	-0.221	5.829	0.081	49.127	0.199	1.878	0.893	0.021
**CFLAR**	-0.293	7.743	-0.582	1.289	0.289	0.641	0.359	13.893	0.534	0.269
**CHAT**	-0.115	3.157	-0.621	0.773	-0.041	56.823	0.036	52.656	0.143	31.624
**CHCHD5**	-0.144	44.812	-0.599	2.773	0.082	54.331	0.488	0.727	0.031	31.624
**CHD1L**	-0.117	49.540							1.277	0.021
**CHD5**	-0.405	0.171	0.461	0.514	0.204	4.733	0.235	27.828	0.124	31.624
**CHFR**	-0.554	0.052	0.628	0.595	0.040	55.771	-0.170	32.497	-0.112	31.624
**CHI3L1**	-0.061	56.513	-0.140	8.881	0.114	6.932	0.141	27.828	0.773	0.021
**CHIC2**	-0.101	33.231	0.549	0.180	0.045	58.074	0.034	52.656	-0.018	41.878
**CHID1**	0.258	7.743	0.508	0.428	0.093	41.103	-0.034	55.172	-0.026	40.051
**CHKA**	-0.233	0.262	0.612	2.773	-0.281	0.517	0.174	41.825	-0.440	0.746
**CHL1**	-0.344	29.668	-0.865	0.812	0.248	0.641	-0.153	13.893	0.228	24.398
**CHMP1B**	0.099	44.812	0.790	0.944	0.050	49.127			-0.436	0.021
**CHMP6**	0.406	0.052	-0.272	6.703	0.330	3.068			0.177	24.398
**Chn1**	0.226	0.529	0.520	4.122	-0.068	54.331	0.048	52.656	-0.347	0.269
**CHPT1**	-0.322	7.743	-0.316	2.859	-0.118	49.127	-0.584	0.061	0.190	14.822
**CHRDL1**	-0.487	9.997	0.402	1.772	0.902	1.309	0.278	3.866	1.076	0.119
**CHRM1**	0.169	3.157	0.285	5.829	0.034	58.074	0.446	0.727	-0.230	31.624
**CHRM3**	0.162	49.540	-0.800	1.534	-0.569	1.819	0.912	2.415	-0.270	6.001
**CHRNA1**	-0.635	0.126	0.502	0.264	0.162	54.331	-0.148	51.354	-0.202	24.398
**CHRNA7**	-0.653	0.647	-0.309	3.205	0.167	36.892	-0.185	32.497	0.046	43.965
**CHST3**	-0.488	0.324	-0.564	2.614	-0.100	36.892	0.073	51.354	0.255	0.977
**CHTF8**	0.171	18.587	-0.129	3.205	0.458	1.819	-0.299	23.450	-0.450	0.307
**CIDEA**	-0.300	7.743			-0.773	0.517	0.585	0.061	-0.179	37.824
**CISH**	-0.103	52.218	-0.143	4.122	0.025	59.793	0.093	51.354	0.449	0.993
**CIT**	-0.453	1.936	0.263	1.772	0.159	17.386	-0.365	0.506	0.273	33.879
**CITED4**	-0.093	37.036	-0.193	2.859	0.124	55.771	0.461	0.506	0.274	1.193
**CKLF**	0.184	29.668	-0.371	0.514	-0.194	8.121	0.213	41.825	0.425	0.471
**CLDN11**			0.163	0.784			-0.163	41.825	0.478	0.601
**CLDN22**	-0.360	15.278	0.200	5.829			-0.139	32.497	-0.556	0.993
**CLDN23**					-0.432	4.733			0.564	0.021
**CLDND1**	-0.276	21.797	0.408	0.514	0.288	14.770	-0.295	27.828	0.448	0.045
**CLEC10A**			0.539	0.174			0.383	47.420	0.532	0.891
**CLEC1B**									0.619	0.802
**CLEC4A**							0.287	41.825	0.560	0.021
**Clec4b1**	-0.049	54.701			0.054	54.331			0.721	0.045
**CLEC5A**	0.155	4.284	-0.165	13.299	-0.088	36.892	-0.405	7.099	0.962	0.021
**CLEC7A**	0.042	49.540	-0.151	5.829	-0.060	55.771			1.975	0.021
**CLIC5**	-0.176	5.928	-0.230	5.829	0.082	56.823	-0.760	0.810	0.599	0.993
**CLIC6**	-0.830	0.052	0.215	6.703	-0.306	4.053	-0.275	49.358	0.205	43.476
**CLIP1**	-0.149	44.812	-0.553	0.514	-0.257	1.819	0.047	47.420	-0.415	0.021
**CLN8**	0.375	1.936	0.455	0.107	0.601	0.092	-0.087	47.420	-0.670	8.002
**CLOCK**	0.364	0.171	0.545	1.772	-0.562	0.092	-0.267	1.878	-0.401	0.993
**CLTC**	0.171	29.668	0.655	0.784	-0.123	49.127	-0.443	3.866	-0.395	0.993
**CLU**	0.023	54.154			0.111	26.120	0.079	47.420	0.564	0.021
**CLVS1**	0.042	52.218	0.933	0.107	0.326	0.517	0.083	41.825	-0.575	0.021
**CLYBL**	0.341	0.052	0.531	2.300	0.126	33.124	-0.121	47.420	-0.518	0.021
**CMAS**	0.318	12.647	0.630	0.669	0.027	59.793	-0.106	16.936	-0.215	4.218
**CMBL**	-0.226	0.171	0.564	0.180	-0.142	20.025	0.059	41.825	-0.301	6.001
**CMKLR1**	-0.107	4.284	-0.417	0.784	-0.076	49.127	-0.048	55.172	-0.028	39.468
**Cml5**	0.165	44.812	0.474	0.784	0.150	33.124	-0.266	0.855	-0.422	0.993
**CMTM7**	0.274	29.668	0.383	1.029	0.079	54.331	0.086	11.241	0.683	0.021
**CMTM8**	-0.095	56.130	0.113	4.122	-0.528	0.092	0.495	0.506	-0.267	1.977
**CNGB1**	-0.733	0.324								
**CNIH3**	-0.107	49.540	0.801	0.669	-0.163	4.053	-0.137	49.358	0.454	0.021
**CNKSR3**	0.130	44.812	0.521	0.859	0.221	33.124	0.159	32.497	0.272	31.624
**CNN3**	0.074	44.812	-0.171	5.829	0.184	0.856	0.039	51.354	0.605	0.021
**CNOT10**	0.114	25.771	0.513	1.457	0.012	59.432	0.152	19.971	-0.738	0.021
**CNOT3**	-0.242	0.171	-0.504	0.944	0.217	4.053	0.290	47.420	0.737	0.891
**CNOT6**	0.041	56.513	-0.658	0.784	0.090	29.328	-0.104	13.893	-0.536	0.993
**CNTLN**	0.038	49.540	0.428	0.534	0.404	4.053	0.066	47.420	0.304	24.398
**CNTN1**	-0.038	52.827	0.140	4.122	-0.137	9.255	-0.167	23.450	-0.469	0.021

**CNTN4**	-0.124	3.157	0.389	5.829	-0.187	26.120	-0.545	1.332	-0.783	0.021
**COBL**	-0.497	0.052	-0.520	3.009	0.518	10.694	-0.453	3.866	0.048	43.476
**COL16A1**	0.070	52.218	0.533	0.484	0.072	49.127	0.647	0.130	0.951	0.021
**COL18A1**	-0.087	49.540	-0.170	3.205	0.467	0.556	0.545	5.543	0.273	11.001
**COL1A1**	-0.269	7.743	-0.320	6.703	-0.129	14.770	0.591	0.061	0.848	0.021
**COL25A1**	0.912	0.324	-0.501	0.514	-0.066	49.127	0.021	53.272	-0.026	40.051
**COL4A1**	0.064	49.540	0.372	1.086	0.034	54.331	-0.503	0.061	-0.379	0.045
**COL4A2**	0.267	4.284	0.294	4.122	0.082	49.127	-0.440	0.061	-0.660	0.021
**COL5A1**	-0.138	49.540	-0.221	4.122	0.171	55.771	0.424	27.828	0.923	0.021
**COL5A3**	0.082	52.827	-0.300	0.784	-0.256	2.056	0.939	0.061	0.975	0.021
**COL6A3**	-0.119	52.218	0.303	1.457	-0.501	0.092	0.223	7.099	0.081	33.879
**COPZ2**	0.094	44.812	0.445	4.122	0.477	0.641	0.446	1.617	0.603	0.021
**COQ4**	0.212	37.036	0.218	2.123	-0.141	29.328	-0.470	8.987	-0.598	0.021
**COX6A2**	-0.397	9.997	-0.352	4.122	0.334	0.092	0.119	41.825	0.573	0.993
**CP**	-0.270	29.668	-0.204	2.614	0.288	4.733	-0.350	0.121	0.338	4.218
**CPE**	0.239	1.431	0.674	0.445	-0.188	33.124	0.052	52.124	-0.476	0.084
**CPLX2**	-0.585	0.052	-0.247	8.253	0.347	26.120	0.283	19.971	-0.405	0.649
**CPLX3**	0.068	51.013	-0.519	0.859	0.604	3.475	-0.070	47.420	0.068	36.831
**CPNE2**	-0.224	0.780	-0.636	4.122	0.153	10.694	-0.146	41.825	0.517	0.021
**CPNE4**	-1.074	0.052	0.220	5.829	-1.033	0.092	0.863	1.617	-1.087	0.039
**CPNE5**	0.231	18.587	0.523	1.534	0.348	9.255	0.079	51.354	0.672	0.129
**CPNE8**	-0.043	54.701	0.729	0.420	0.063	41.103	0.206	16.936	0.403	0.993
**CPNE9**	-0.486	0.052	-0.228	6.703	0.465	3.068	-0.194	51.354	-0.572	14.822
**CPSF2**	-0.059	51.013	0.619	0.595	-0.360	12.575	0.703	0.506	0.249	0.649
**CPSF3**	0.302	0.930	0.451	0.986	0.195	17.386	0.071	47.420	-0.261	1.977
**CPSF6**	0.531	0.930	-0.296	2.859	0.012	59.793	0.371	23.450	-0.860	0.021
**CPT2**	0.063	49.540	-0.202	0.784	-0.276	0.240			0.476	0.891
**CRABP1**	0.632	0.171	0.339	0.354	0.413	4.733			0.322	11.001
**CREB3L2**	0.164	25.771	-0.261	0.595	0.476	0.092	-0.338	0.947	0.297	4.218
**CREB5**	0.302	7.743	0.445	0.514	-0.379	0.440	-0.320	41.825	0.871	0.993
**CREBL2**	-0.507	0.052	0.258	1.212	-0.178	49.127	0.301	0.130	0.664	0.213
**CREG1**	0.150	25.771	0.704	1.534	-0.116	26.120	0.089	52.124	-0.422	0.977
**CRHR2**	-0.188	33.231	-0.415	0.893	0.198	8.121	0.138	47.420	0.093	35.531
**CRIM1**	-0.763	0.093	0.281	5.829	-0.365	0.517	-0.390	23.450	-0.493	0.021
**CRIP1**	-0.074	49.540	0.445	0.595	0.251	0.440	-0.121	7.099	0.482	0.307
**CRIP3**			-0.444	0.986	0.221	0.856	0.060	47.420	-0.262	8.002
**CRISPLD2**	-0.146	9.997	-0.426	2.773	0.220	0.659	0.494	8.987	0.127	44.315
**CRLF1**	-0.300	21.797	-0.396	0.705	0.040	58.406	0.047	50.493	0.340	24.398
**CRLS1**	-0.125	44.812	0.691	1.289	-0.522	0.402	-0.098	13.893	0.371	31.624
**CROT**	-0.103	49.540	0.693	0.514	-0.103	22.899	0.279	1.617	-0.467	0.021
**CRTAM**	-0.079	49.540	-0.168	4.122	-0.205	20.025	-0.568	0.987	0.119	31.624
**CRYAA**	-0.672	0.052			-0.203	36.892	0.278	2.415	0.263	24.398
**CRYBA4**	-0.340	33.231			-0.207	54.331	0.309	27.828	0.909	0.021
**CRYM**	-0.092	52.218	0.116	8.253	-0.488	0.092	0.114	41.825	-0.423	0.039
**CSE1L**	0.088	37.036	0.494	3.205	-0.047	58.928	-0.220	0.292	-0.352	0.066
**CSF1R**	-0.071	57.367	-0.225	0.514	-0.095	26.120	0.225	2.415	0.630	0.021
**CSF2RA**			-0.437	2.773	0.100	41.103	-0.091	16.936	0.496	0.021
**CSF2RB**	-0.699	0.052	-0.234	4.122	-0.329	10.694	-0.375	27.828	0.681	0.021
**CSNK1G1**	-0.811	0.170	-0.636	2.773	-0.331	2.311	-0.701	1.878	-0.527	0.993
**CSPG4**	-0.243	3.157	-0.132	7.537	0.175	2.659	0.025	52.656	0.424	0.391
**CSPP1**	-0.058	55.170	0.093	10.404	-0.043	55.771	-0.380	0.061	-0.361	0.039
**CSRP2**	0.131	7.743	0.515	0.784	0.234	36.892	-0.598	0.506	0.146	14.822
**CST7**					-0.087	49.127			2.028	0.021
**CSTF1**	0.029	54.154	0.240	2.300	0.059	49.127	0.066	47.420	0.422	0.039
**CTBP2**	-0.324	0.170	-0.275	6.703	-0.218	36.892	0.053	47.420	-0.493	0.213
**CTDSP1**	-0.210	0.324	-0.113	7.537	0.028	57.395	0.177	2.415	0.447	0.269
**CTF1**	-0.259	9.997	0.141	7.537	0.086	41.103			0.709	0.021
**CTGF**	-0.434	1.936	0.389	0.484	0.180	49.127	-0.158	1.332	-0.102	33.879
**CTH**			0.687	0.300	0.043	58.074	-0.149	13.893	-1.097	0.021
**CTNNA2**	-0.302	0.052	0.437	0.595	-0.195	1.098	-1.064	0.061	-0.425	0.119
**CTNNB1**	-0.244	44.812	0.652	0.180	-0.114	33.124	-0.587	0.250	-0.948	0.021
**CTNNBIP1**	-0.066	37.036	-0.146	8.881	-0.050	56.823	0.078	47.420	0.470	0.649
**CTNND1**	0.226	1.936	-0.610	2.859	0.399	0.092	0.061	47.420	0.165	35.531
**CTR9**	0.080	44.812	-0.417	0.514	-0.088	26.120	0.079	27.828	-0.090	24.398
**CTSC**	0.142	44.812	0.464	2.300	-0.472	22.899	0.407	0.506	-0.118	36.831
**CTSD**	0.233	12.647	0.293	4.122	0.069	54.331			0.442	0.391
**CTSE**			-0.301	1.534			-0.035	50.493	0.463	0.398
**CTSH**	-0.019	57.293	-0.220	3.009	0.073	49.127	0.018	53.829	0.830	0.021

**CTSS**	-0.110	44.812	-0.163	4.122	-0.050	60.231			1.058	0.021
**CTSZ**	0.137	25.771	0.195	8.881			-0.046	51.354	0.641	0.021
**CTTNBP2**	0.289	21.797	0.464	0.986	-0.087	56.823	-0.288	16.936	-0.499	0.269
**CUL2**	0.077	52.218	0.513	2.859	0.031	58.611	0.122	41.825	0.829	0.021
**CUL4B**	0.068	52.218	0.510	0.986	-0.085	41.103	0.084	41.825	-0.287	1.193
**CUL9**	-0.111	29.668	0.390	2.773	0.054	54.331	-1.045	0.292	0.284	0.269
**CUTC**	-0.027	57.293	0.202	4.122	0.231	2.056	0.454	0.855	0.207	14.822
**CUX2**	0.091	29.668	0.206	2.773	0.265	12.575	0.206	1.878	0.565	0.649
**CX3CR1**	0.034	49.540	-0.390	0.514	0.338	33.124	-0.227	41.825	0.638	0.021
**CXCL10**									1.224	0.119
**CXCL13**					-0.092	54.331			1.167	0.021
**CXCL14**									1.009	0.021
**CXCL16**	-0.583	1.936	-0.343	0.514	-0.060	49.127	0.194	1.878	1.625	0.021
**CYB5R4**	0.100	33.231	0.524	1.534	0.298	17.386	0.537	11.241	-0.508	0.021
**CYBA**	-0.088	52.218			0.104	22.899	0.392	0.061	0.913	0.021
**CYFIP2**	-0.288	5.928	-0.449	0.595	-0.560	0.092	-0.144	47.420	-0.131	24.398
**CYLD**	0.418	0.171	0.289	1.457	-0.234	5.705	-0.108	32.497	-0.557	0.021
**CYP1B1**	-0.154	44.812	-0.232	11.783			-0.424	13.893	0.526	0.119
**CYP26B1**	-0.672	0.262	0.356	0.877	-0.612	1.309	-0.993	0.061	0.103	24.398
**Cyp2c44**	-0.153	49.540	-0.750	0.944	0.172	22.899			-0.306	4.218
**CYP2D6**	-0.587	0.052	0.307	0.784	0.042	55.771	-0.799	0.061	0.405	0.269
**CYP2G1P**	0.441	0.126	0.168	10.404	-0.152	3.475	0.400	4.906	0.544	0.021
**CYP2J2**	0.082	44.812	0.553	0.514	-0.065	49.127	0.333	2.415	-0.877	0.021
**CYP46A1**	-0.104	21.797	-0.571	0.614	0.009	60.505	0.021	55.172	0.115	31.624
**CYP4F12**	0.095	37.036	0.431	2.300	0.363	9.255	-0.323	0.109	0.465	0.993
**CYP4X1**	0.076	33.231	-0.444	1.029	-0.166	26.120	-0.041	49.358	-0.513	0.045
**CYP51A1**	0.223	0.171	0.652	1.212	0.324	3.475			-0.436	0.066
**CYR61**	0.042	52.218	0.133	1.534			-0.332	11.241	0.638	0.471
**CYTH4**	-0.088	33.231	0.102	12.522	0.059	49.127	0.632	0.292	0.935	0.021
**D2HGDH**	-0.346	1.098	0.339	0.784	-0.145	33.124	-0.524	0.061	0.352	31.624
**D830050J10RIK**	0.107	25.771	0.270	2.300	0.146	12.575	0.105	51.354	0.676	0.021
**DAAM1**	-0.312	1.431	0.440	1.029	-0.166	6.932	0.446	0.061	-0.338	0.021
**DAB1**	0.432	0.529	-0.526	5.829	0.282	1.309	-0.161	16.936	-0.231	0.166
**DAB2**	-0.092	44.812	0.420	0.122	0.024	56.823	-0.034	54.403	0.427	0.746
**DACH1**	0.172	12.647	-0.639	0.180	-0.295	6.932	0.227	0.727	0.042	43.008
**DACT2**	0.079	44.812	-0.390	4.122	0.272	6.932	0.412	1.617	0.553	0.066
**DAG1**	-0.089	51.013	0.558	0.784	0.217	10.694	-0.087	41.825	-0.427	0.021
**DAGLA**	0.232	1.431	-0.500	3.205	0.085	49.127	-0.300	8.987	0.619	0.166
**DAP**	0.077	53.690	0.250	3.205	0.506	0.092	0.160	41.825	0.242	11.001
**DAPP1**	0.476	0.398	-0.085	10.404	0.133	49.127	-0.608	2.415	0.101	44.052
**DARS**	0.382	12.647	0.721	1.457	-0.113	49.127	0.567	2.415	0.691	0.021
**DBI**	-0.027	55.559					0.127	16.936	0.542	0.066
**DCAF10**	0.165	18.587	0.508	0.514	0.103	17.386	0.374	0.121	-0.395	0.746
**DCAF12**	-0.315	37.036	0.632	0.204	0.070	49.127	0.179	0.855	0.396	0.119
**DCC**	0.022	54.701	0.278	0.514	-0.487	0.092	0.660	0.061	-0.393	0.391
**DCK**	0.204	2.450	0.225	4.122	0.093	54.331	-0.382	0.109	-0.525	0.119
**DCLK1**	-0.381	0.171	0.468	0.445	-0.579	0.092	-0.190	0.947	-0.541	0.021
**DCLRE1A**	0.211	21.797	-0.753	0.877	-0.264	17.386	-0.100	52.656	0.461	0.021
**DCN**	-0.268	37.036	0.181	8.253	0.589	5.705	0.107	8.987	0.499	0.801
**DCTD**	-0.275	33.231	0.581	3.009	0.620	0.092	0.194	47.420	-0.148	36.831
**DCUN1D3**	0.135	44.812	-0.354	2.859	0.111	49.127	-0.134	47.420	0.447	0.746
**DCXR**	0.199	5.928	-0.300	0.514	0.084	41.103	0.252	5.543	0.487	0.021
**DDC**	-0.483	1.431	0.186	11.783	-0.284	22.899	-0.937	0.061	0.340	24.398
**DDHD2**	0.068	44.812	0.305	2.300	0.015	58.611	0.252	23.450	0.433	0.269
**DDIT4L**	0.051	55.170	0.457	5.829	0.351	8.121	-0.115	49.358	-0.403	0.977
**DDN**	0.100	52.218	0.401	1.212	0.172	29.328	-0.312	0.109	-0.448	0.021
**DDR1**	-0.111	33.231			0.021	58.074	0.063	52.124	0.654	0.166
**DDX1**	-0.404	0.052	0.358	0.514	-0.253	2.311	-0.527	47.420	0.416	0.307
**DDX17**	-0.106	44.812	0.271	3.009	0.060	54.331	-0.143	3.866	-0.944	0.021
**DDX31**							-0.471	0.573	0.075	35.531
**DDX46**	-0.243	0.780	0.840	0.595	-0.362	0.092	-0.200	1.210	-0.227	1.977
**DDX50**	0.187	33.231	0.677	0.877	0.066	36.892	0.066	32.497	-0.422	0.066
**DDX6**	-0.496	1.431	0.083	11.093	-0.405	0.281	0.080	52.124	-0.259	0.471
**DEAF1**	0.166	29.668	-0.592	0.773	0.298	8.121	0.052	50.493	0.249	4.218
**DEF6**	0.437	0.052	0.150	0.784	0.522	0.092	0.685	0.292	-0.370	24.398
**DENR**	0.110	49.540	0.777	1.289	-0.094	54.331	0.164	23.450	-0.456	0.021
**DEPTOR**	0.126	49.540	-0.241	2.773	-0.612	4.053	-0.791	0.061	0.572	0.977
**DFFA**	0.380	0.052	0.444	0.514	0.414	9.255	0.082	49.358	-0.667	0.021

**DFNB31**	-0.505	0.052	0.077	12.522			0.125	13.893	0.496	0.021
**DGKB**	-0.102	33.231	0.517	1.289	0.262	4.053	0.412	7.099	-0.524	0.993
**DGKH**	-0.721	0.052	0.329	8.253	-0.333	20.025	0.317	2.913	-0.844	0.119
**DHCR24**	0.603	0.780	0.215	8.881	-0.082	56.823	-0.473	1.332	-0.523	0.993
**DIDO1**	-0.115	29.668	-0.530	0.986	-0.207	17.386	-0.503	27.828	-0.254	1.193
**DIO2**	-0.098	51.013	0.482	0.107	0.223	33.124	0.073	50.493	-0.152	14.822
**DKKL1**	-0.073	44.812	-0.300	3.009	0.516	0.517			-0.214	6.001
**DLG1**	-1.024	0.052	0.368	0.354	0.343	3.475	0.183	27.828	-1.168	0.021
**DLG2**	0.065	49.540	0.261	8.253	-0.675	0.092	0.428	2.415	-0.411	0.021
**DLGAP2**	-0.069	49.540	-0.184	4.122	-0.360	2.659	-0.192	41.825	0.974	0.021
**DLL1**	0.118	37.036	0.573	0.267	0.273	17.386	0.217	1.210	0.184	39.468
**DMP1**	0.194	0.930	-0.378	9.629	0.322	3.068	0.347	0.061	0.500	31.624
**DMRT2**					0.038	55.771	-0.784	0.061	0.249	35.531
**DMXL2**	-0.526	0.052	0.501	2.300	-0.386	0.641	0.353	13.893	-0.380	24.398
**DNAH6**	-0.026	56.874	0.436	4.122	0.332	0.641	0.510	0.727		
**DNAJB4**	-0.219	29.668	0.309	0.595	-0.243	4.733	-0.239	8.987	-0.880	0.021
**DNAJB5**	0.156	33.231	0.447	0.420	0.432	0.517	-0.374	0.130	-0.490	0.021
**DNAJB9**	-0.110	52.218	0.635	0.107	-0.141	12.575	0.378	7.099	-0.231	0.307
**DNAJC1**	-0.327	0.052	0.583	2.300	-0.375	0.998	-0.457	0.061	0.148	35.531
**DNAJC13**	0.945	0.398	0.343	4.122	0.097	26.120	0.034	52.124	-0.318	1.193
**DNAJC18**	-0.073	37.036	0.458	1.029	-0.348	8.121	0.582	23.450	0.413	0.746
**DNAJC19**	-0.340	0.126	-0.188	4.122	-0.101	49.127	-0.377	0.177	-0.281	11.001
**DNAJC2**	-0.137	49.540	-0.625	0.223	-0.294	0.402			0.159	33.879
**DNAJC21**	0.178	5.928	0.394	0.893	0.105	49.127	0.285	8.987	-0.342	4.218
**DNAJC24**	0.285	0.262	0.606	1.663	0.028	58.406	-0.094	41.825	-0.360	0.021
**DNAJC28**	-0.472	0.052	0.572	0.180	-0.868	0.092	0.679	0.061	0.729	0.471
**DNAL1**	-0.072	57.293	0.575	1.029	-0.160	41.103	-0.190	0.292	-0.451	0.391
**DNALI1**	-0.041	53.690	0.405	3.009	0.176	12.575	0.059	52.124	0.947	0.021
**DNASE1L1**	-0.264	52.218			-0.137	41.103	-0.261	49.358	0.513	0.021
**DNM1L**	-0.293	21.797	0.627	1.457	-0.518	0.092	0.034	51.354	-0.438	0.021
**DNMT3A**	-0.114	5.928	0.368	1.086	0.416	0.641	-0.813	0.855	-0.281	24.398
**DOCK1**	-0.305	0.529	-0.555	0.893	0.214	2.659	0.043	47.420	0.567	0.045
**DOCK11**	-0.708	0.780	0.469	0.669	-0.484	0.856	-0.694	5.543	-0.561	0.891
**DOCK4**	-0.339	0.324	0.546	1.212	-0.236	4.733	-1.073	0.250	-0.674	0.021
**DOK1**	0.346	1.098	0.288	0.300	0.211	0.641	0.146	19.971	0.755	0.021
**DONSON**	0.082	44.812	0.876	0.877	-0.078	33.124	0.075	41.825	-0.480	0.119
**DPM1**	-0.246	0.052	0.280	1.772	-0.278	0.641	-0.818	0.061	-0.594	0.021
**DPPA2**	-0.083	49.540	-0.511	0.514	-0.089	29.328	0.047	47.420	-0.095	24.398
**DPYD**	-0.316	25.771	0.243	5.829	-0.234	36.892	-0.231	47.420	-0.956	0.471
**DPYSL4**	0.039	52.218	-0.132	2.614	0.161	36.892	0.127	7.099	0.502	0.021
**DR1**	0.041	54.154	0.544	2.773	0.025	58.074	-0.268	0.506	-0.416	0.039
**DRAM1**	0.381	0.529	0.485	0.354	0.288	0.092	0.977	0.121	-0.176	14.822
**DRAM2**	0.008	56.765	-0.153	0.784					-0.374	0.891
**DRD2**	-0.590	0.529	-0.212	4.122	-0.215	8.121	-0.071	50.493	0.419	0.746
**DRD4**	-0.312	0.780	0.299	4.122	0.379	20.025	0.048	53.272	0.625	0.045
**DRD5**	-0.152	5.928	0.595	2.614	-0.083	54.331	-0.255	41.825	-0.229	0.471
**DROSHA**	0.130	12.647	0.454	2.614	0.166	1.098	0.135	47.420	-0.595	0.021
**DRP2**	0.256	3.157	0.321	7.537	-0.166	54.331	0.144	41.825	-0.559	0.066
**DSG2**	0.062	49.540	-0.195	5.829	-0.041	54.331			0.438	0.391
**DSP**	0.322	44.812	-0.549	0.614	-0.294	54.331	0.105	41.825	0.397	24.398
**DSTN**	0.136	44.812	0.673	0.514	-0.219	26.120	-0.067	47.420	-0.415	0.269
**DTX4**	-0.208	12.647	0.457	0.784	0.415	0.092	-0.269	1.332	0.582	0.021
**DUSP1**	-0.210	9.997			-0.206	41.103	-0.594	0.727	0.456	0.021
**DUSP16**	-0.445	0.052	0.756	0.354	0.053	49.127	0.543	0.130	0.627	0.993
**DUSP2**	0.015	56.765			0.131	49.127	0.544	0.061	0.208	0.746
**DUSP26**	-0.325	0.052	-0.331	2.859	0.246	17.386	0.487	0.061	0.335	4.218
**DUSP4**	0.214	0.930	0.194	1.663	0.191	3.475	0.105	41.825	-0.417	0.269
**DUT**	0.019	56.874	0.401	0.514	-0.028	57.395	0.161	13.893	0.252	4.218
**DYNC1H1**	-0.394	0.052	0.530	0.944	-0.181	58.928	-0.948	0.399	-0.177	11.001
**DYNC2H1**	-0.794	0.052	0.615	0.944	-0.760	0.440	-0.436	0.947	0.608	0.021
**DYRK1A**	-0.232	5.928	0.727	0.595	-0.209	10.694	-0.838	0.061	-0.402	0.269
**DYRK1B**	-0.048	51.013	-0.448	0.784	0.053	54.331	0.237	11.241	0.522	0.021
**E2F1**	0.270	1.098	0.211	0.514	0.407	2.056	0.275	41.825	0.821	0.119
**E2F3**	0.125	37.036	0.370	0.514	-0.367	0.724	0.532	0.121	-0.553	0.021
**E2F5**	-0.048	55.870	0.309	2.614	0.081	26.120	-0.419	0.061	-0.203	0.891
**EARS2**	0.112	44.812	0.418	4.122	-0.045	58.611	-0.781	0.061	-0.234	14.822
**EBF3**	0.279	44.812	-0.705	2.773	-0.417	0.281	0.987	0.061	0.409	39.468
**EBI3**	0.309	0.052	0.115	5.829	0.313	0.517	-0.364	11.241	0.397	0.977

**ECHDC3**	-0.087	54.701	0.245	1.534	0.181	56.823	-0.077	51.354	1.129	0.021
**EDA**	0.439	0.052	0.234	8.881	0.282	0.092	0.194	7.099	-0.612	0.021
**EDNRB**	-0.899	0.052	-0.410	0.180	-0.199	49.127	-0.148	41.825	0.746	0.021
**EEA1**	0.191	3.157	-0.902	0.669	-0.375	2.311	0.231	0.399	-0.265	0.084
**EEF1D**	-0.243	9.997	-0.387	0.669	0.743	0.092	-0.710	1.332	-0.925	0.021
**EEPD1**	0.258	0.529	0.112	5.829	-0.050	59.793	0.071	49.358	-0.491	0.039
**EFCAB2**	-0.284	33.231	0.712	1.029	-0.145	55.771	0.323	5.543	-0.679	0.021
**EFEMP2**	-0.239	0.171	0.059	11.783	0.021	60.231	0.102	49.358	0.553	0.977
**EFHA1**	-0.491	0.052	0.736	0.107	0.218	29.328	0.654	0.341	0.077	35.531
**EFNA5**	-0.145	44.812	-0.589	0.784	0.238	41.103	-0.657	0.061	0.112	24.398
**EFNB2**	-0.156	52.218	0.648	0.354	0.279	12.575	0.223	0.573	-0.213	0.891
**EFR3A**	0.204	44.812	0.705	2.123	-0.145	36.892	-0.464	0.341	-0.654	0.066
**EFR3B**	-0.151	49.540	0.607	0.569	-0.152	49.127	0.059	41.825	-0.497	0.993
**EFS**	-0.151	5.928	-0.535	2.859	-0.126	41.103	-0.103	51.354	0.520	0.021
**EGF**	-0.135	29.668	0.345	0.514	0.430	14.770	-0.717	0.061	-0.704	0.993
**EGFR**	-0.421	0.780	0.306	1.457	-0.297	0.281	0.771	3.866	0.446	0.119
**EGR1**	0.541	0.324	0.507	0.602	0.230	12.575	-0.224	1.878	-0.059	37.824
**EGR2**	1.349	0.052	0.599	0.267	0.589	0.659	-0.089	41.825	0.320	35.531
**EGR4**	0.649	0.052	0.109	11.093	0.206	3.475	0.110	47.420	0.347	1.193
**EHD4**	-0.279	0.171	-0.548	2.614	-0.033	60.505			0.530	0.119
**EIF2AK1**	-0.359	0.171	-0.248	3.205	0.206	1.556	0.521	0.061	-0.391	0.213
**EIF2AK2**			0.468	0.514	0.087	41.103	0.019	55.172	0.562	0.977
**EIF2AK4**	0.192	3.157	-0.559	2.773	-0.284	14.770	-0.154	13.893	-0.354	0.977
**EIF2C1**	0.272	3.157	-0.987	0.107	0.127	17.386	0.066	52.124	0.356	0.471
**EIF2S3**	-1.061	0.052	0.345	8.253	0.128	12.575	-0.676	0.353	-1.376	0.021
**EIF3A**	-0.184	33.231	0.717	1.663	-0.431	0.402	0.098	16.936	-0.443	0.039
**EIF3D**	0.139	49.540	0.203	11.783	-0.075	49.127	-1.043	0.506	-0.101	31.624
**EIF3E**	0.106	29.668	-0.383	0.773	-0.068	49.127	0.024	53.272	0.360	24.398
**EIF3J**	0.238	9.997	-0.129	5.829	0.082	49.127	0.301	32.497	0.598	0.021
**EIF4A1**	0.245	0.324	0.626	2.859	0.055	55.771	-0.145	3.866	0.621	0.119
**Eif4e2**	0.211	37.036	0.509	0.784	-0.117	49.127			-0.247	14.822
**EIF4EBP1**	-0.358	0.052	-0.785	2.614	0.271	2.311	0.021	53.272	0.523	0.021
**EIF4G2**	-0.318	0.171	0.711	2.300	-0.285	6.932	0.360	27.828	-0.510	0.021
**EIF6**	0.453	0.052	0.135	11.093	0.179	12.575	0.078	47.420	0.055	42.670
**ELAVL2**	-0.501	0.052	-0.664	0.180	-0.400	0.659	-0.394	23.450	-0.521	0.746
**ELF1**	-0.118	25.771	-0.890	2.773	-0.322	1.556	0.608	0.177	0.553	0.993
**ELK3**	-0.096	44.812	-0.237	0.514	0.055	49.127	-0.108	51.354	0.917	0.021
**ELL2**	-0.518	0.052	-0.772	1.289	0.224	2.659	-0.281	50.493	-0.418	0.649
**ELMO1**			0.163	5.829	0.038	54.331	-0.274	0.947	0.456	0.129
**ELMO2**	0.394	0.052	0.107	7.537	0.168	41.103	-0.055	47.420	0.479	0.649
**ELMOD1**	0.249	0.262	0.439	4.122	0.206	33.124	-0.544	0.061	-0.464	0.471
**ELOVL1**			-0.261	3.205	0.116	8.121	0.087	49.358	0.543	0.021
**ELOVL7**	0.038	55.870	0.609	2.300	0.017	59.432	-0.408	0.727	0.158	24.398
**EMB**	0.142	49.540	0.395	1.663	0.433	3.068	0.554	0.130	0.121	31.624
**EMID1**	-0.121	51.013	-0.044	13.299			-0.133	47.420	0.510	0.918
**EML1**	-0.410	7.743	-0.530	8.881	0.062	49.127	0.074	41.825	-0.516	0.129
**EML5**	0.012	56.513	0.307	4.122	0.027	58.928	-0.593	2.913	-0.803	0.021
**EML6**	0.235	1.431	0.609	0.212	0.115	36.892	0.848	7.099	0.135	33.879
**EMP1**	-0.306	0.930	0.174	12.522	0.147	49.127	-0.587	0.061	0.397	0.993
**EMP3**	-0.250	9.997	0.166	8.881	0.178	9.255	0.424	32.497	0.637	0.119
**EMR1**	0.007	57.109	-0.809	0.174	0.054	54.331	-0.066	51.354	0.842	0.021
**EMX2**	0.425	0.324	0.418	1.772			-0.186	41.825	-0.392	0.021
**Enah**	-0.358	0.126	-0.610	4.122	-0.123	26.120	-0.599	0.947	-0.762	0.021
**ENC1**	-0.764	0.171	-0.706	0.300	-0.572	0.440	-0.633	13.893	-0.268	11.001
**ENO3**	-0.252	7.743	-0.423	0.784	0.027	58.406	0.188	47.420	0.396	0.649
**ENOX1**	0.061	54.154	0.336	0.569	-0.101	33.124	0.022	53.272	-0.484	0.021
**ENPP1**	-0.304	25.771	-0.168	5.829			-0.276	49.358	0.460	0.129
**ENTPD2**	-0.137	3.157					-0.097	52.124	0.556	0.021
**ENTPD3**	-0.421	0.262	0.285	5.829	-0.532	0.092	-0.452	0.061	-0.586	0.993
**ENTPD4**	0.137	25.771	0.429	0.663	0.021	59.793	0.019	55.172	0.118	33.879
**ENTPD7**	0.382	1.431	-0.861	0.614	-0.045	54.331	-0.037	49.358	0.270	31.624
**EPB41L2**	-0.099	33.231	-0.119	5.829	0.471	0.092	0.061	41.825	0.606	0.021
**EPC1**	-0.493	7.743	-0.631	0.859	0.078	54.331	0.451	2.913	0.230	31.624
**EPHA3**	-0.376	0.511	0.780	0.180	-0.272	0.517	0.481	0.353	-0.509	0.166
**EPHA5**	0.303	25.771	0.621	0.784	-0.384	8.121	-0.127	41.825	-0.520	0.021
**EPHB1**	-0.150	12.647					-0.560	3.866	0.477	0.021
**EPHB2**			0.132	9.629	0.067	54.331	0.120	7.099	-0.431	0.021
**ERAL1**	-0.263	0.324	-0.571	0.230	-0.221	3.068	-0.279	2.415	0.291	24.398

**ERBB2IP**	-0.535	0.052	0.429	2.300	-0.138	6.932	0.673	8.987	0.290	31.624
**ERBB3**	0.093	37.036	0.400	0.669	0.243	14.770	0.454	1.617	0.505	0.269
**ERC2**	-0.170	18.587	0.559	1.212	-0.167	33.124	0.345	11.241	-0.439	0.307
**ERCC6**	-0.063	51.013	0.816	1.289	0.213	1.309	-0.087	41.825	-0.395	0.166
**ERF**	-0.064	44.812	0.094	7.537	0.161	20.025	0.192	2.415	0.413	0.119
**ESCO1**	0.420	3.157	0.545	0.420	-0.143	26.120	-0.331	3.866	-0.138	31.624
**ETFDH**	0.077	49.540	0.543	1.212	-0.110	36.892	-0.795	0.121	-1.055	0.021
**ETV6**	0.093	49.540	-0.444	1.086	0.197	2.311	-0.219	5.543	0.416	0.119
**EVI5L**	0.207	1.098	0.519	0.569	0.252	0.517	0.329	32.497	0.689	0.471
**EVPL**	-0.157	0.529	-0.472	0.514	0.316	9.255	0.295	3.866	0.466	0.021
**EXD1**	-0.710	0.052	0.345	0.484	-0.086	60.505	-0.346	50.493	0.695	0.746
**EXOC2**	0.402	0.171	0.276	0.602	-0.114	14.770	0.240	32.497	0.318	1.977
**EXOC5**	0.192	21.797	0.650	1.086	0.054	54.331	0.438	1.617	-0.561	0.066
**EXOSC3**	0.080	37.036	0.531	0.267	-0.476	2.311	0.272	0.130	-0.216	31.624
**EZH2**	0.042	52.218	0.469	0.669			-0.185	41.825	0.167	42.212
**F11R**	-0.390	18.587			0.061	36.892	-0.257	47.420	0.613	0.021
**F2R**	0.028	55.559	-0.122	12.522	-0.274	26.120	-0.165	1.878	-0.337	0.993
**F3**	0.014	56.130	0.574	0.986	0.014	59.793			0.289	44.315
**FABP7**	-0.465	0.262	0.506	0.877	0.234	12.575	0.289	0.177	0.375	24.398
**FADD**	-0.089	44.812	-0.421	0.514	0.101	41.103	0.564	0.250	0.173	2.886
**FAIM**			0.706	0.300			0.205	27.828	-0.133	14.822
**FAM105A**	0.303	29.668	0.441	0.595			0.195	50.493	0.132	41.878
**FAM107B**	0.082	52.218	0.220	0.514	0.100	36.892	-0.612	0.061	-0.117	24.398
**FAM109A**	-0.113	18.587	-0.154	2.859	-0.048	57.395			0.413	0.166
**FAM114A1**	0.457	0.052			0.747	1.556			0.286	2.886
**FAM120B**	0.104	44.812	0.303	0.784	0.046	49.127	0.034	49.358	-0.370	0.649
**FAM123B**	-0.477	0.052			-0.372	0.092	0.187	27.828	-0.316	31.624
**FAM135A**	-0.216	2.450	0.717	0.595	-0.087	36.892	-0.129	2.913	-0.591	0.021
**FAM13B**	0.085	44.812	0.671	1.086	0.068	41.103	-0.051	49.358	-0.694	0.039
**FAM13C**	0.027	56.765	0.813	0.669	-0.144	10.694	0.108	8.987	-0.531	0.021
**FAM14A**	-0.113	49.540	-0.168	4.122	0.013	59.432	1.151	0.341	1.622	0.021
**FAM172A**	-0.113	44.812	-0.683	0.893	-0.232	9.255	0.777	5.543	0.337	8.002
**FAM176A**	-0.353	0.511	0.621	0.595	-0.337	14.770	-0.477	11.241	-0.648	0.021
**FAM184B**	-0.872	0.052	0.186	0.595	-0.209	41.103	-0.135	32.497	-0.380	0.045
**FAM185A**	-0.134	15.278	0.102	12.522	0.031	55.771	0.072	49.358	-0.498	0.021
**FAM188A**	0.042	52.218	0.108	11.093	-0.165	41.103	0.084	51.354	-0.443	8.002
**FAM198B**	0.433	3.157	-0.072	11.093	-0.154	49.127	-0.092	52.124	-0.506	0.269
**FAM19A1**	0.259	1.098	0.396	1.289	0.055	54.331	0.170	27.828	-0.444	0.021
**FAM19A2**	-0.300	33.231	-0.058	13.299	-0.281	0.092	-0.048	47.420	-0.836	0.649
**FAM203A/FAM203B**	0.063	49.540	0.330	1.772	-0.090	54.331	-0.069	41.825	-0.439	0.021
**FAM40B**	0.027	54.701	0.165	2.614	-0.532	3.068	-0.266	0.506	-0.471	0.213
**FAM46A**	-0.331	0.093	-0.395	6.703	-0.360	6.932	-0.241	47.420	0.101	14.822
**FAM49B**	0.087	37.036	0.124	5.829	-0.171	8.121	-0.126	27.828	-0.459	0.021
**FAM53B**	0.314	5.928	0.360	5.829	0.452	0.281	-0.444	8.987	0.053	43.008
**FAM59A**	0.213	9.997	0.637	1.457	-0.121	22.899	0.106	47.420	-0.405	0.891
**FAM5B**	-0.072	55.870	0.472	2.773	-0.610	0.092	0.363	2.415	-0.279	6.001
**FAM5C**	0.214	44.812	0.620	0.354	0.182	49.127	0.370	0.250	-0.114	24.398
**FAM65B**	0.080	49.540	0.902	0.602	0.266	4.733	0.151	8.987	-0.232	4.218
**FAM78B**			-0.329	4.122	0.128	17.386	-0.124	11.241	-0.772	0.021
**FAM81A**	-0.132	49.540	-0.249	4.122	-0.122	49.127	-0.257	7.099	-0.378	0.891
**FANCA**	-0.798	0.052	-0.302	6.703	0.264	49.127			0.463	24.398
**FANCB**	0.263	12.647	0.582	0.514	-0.160	6.932	-0.551	0.399	-0.498	0.021
**FANCD2**	-0.592	0.529	0.344	2.773	0.350	5.705	0.452	4.906	-0.863	0.021
**FAR1**	0.107	52.218	0.451	1.212	-0.109	41.103	0.247	5.543	-0.540	0.307
**FARP1**	0.499	0.324	0.331	4.122	-0.224	29.328	-0.184	19.971	1.023	0.021
**FAS**	0.029	52.827	0.401	5.829	-0.177	8.121	0.291	0.177	0.570	0.021
**FASLG**	0.771	0.052	-0.271	6.703	0.358	0.724	0.093	41.825	0.136	33.879
**FASTK**	0.127	33.231	0.358	0.484	0.105	17.386	-0.477	0.061	0.204	44.052
**FAT3**	0.426	0.529	-0.398	4.122	-0.241	2.056	0.080	41.825	0.037	44.052
**FAT4**	0.102	25.771	0.774	0.595	0.031	55.771	-0.201	27.828	-0.625	0.021
**FBLN2**	-0.088	55.170			0.524	0.659	-0.108	53.829	0.294	0.213
**FBLN5**	-0.511	0.666	0.227	6.703	0.123	29.328	-0.292	11.241	0.934	0.021
**FBN2**	-0.100	37.036	0.304	2.773	0.243	0.856	-0.921	1.210	-0.086	41.258
**FBRS**	0.103	44.812	-0.301	3.009	0.089	49.127	0.175	13.893	0.619	0.045
**FBXL18**	-0.103	49.540	-0.274	6.703	-0.128	49.127	-0.298	32.497	0.477	0.021
**FBXL7**	0.097	44.812	-0.768	1.457	-0.430	5.705	1.269	0.121	0.688	0.977
**FBXO21**	0.260	12.647	0.272	0.514	-0.175	49.127	1.439	0.061	-0.827	0.021
**FBXO22**	0.313	0.170	0.665	0.784	0.097	49.127			-0.638	0.649

**FBXO3**	0.430	0.126	0.562	0.514	0.208	54.331	-0.364	0.061	-0.777	0.066
**FBXO4**	-0.085	33.231	-0.663	0.986	0.092	49.127	-0.148	4.906	0.047	40.660
**FBXW8**	0.087	49.540	0.366	5.829	0.105	33.124	0.613	0.061	-0.288	0.084
**FCER1G**	-0.133	49.540	-0.544	1.663	0.052	56.823	0.301	0.061	1.062	0.021
**FCGR1A**	0.313	0.666			0.019	58.406	0.171	5.543	1.449	0.021
**FCGR2A**	0.507	0.052	0.373	5.829	0.262	0.659	-0.283	2.913	0.708	0.021
**FCGR2B**	-0.631	0.052	-0.482	1.289	-0.316	2.659	0.292	0.353	-0.415	6.001
**FCGR3A**	0.039	51.013	-0.331	3.009	0.066	49.127	0.013	55.172	0.559	0.021
**FCGRT**	-0.094	52.218	0.228	4.122	0.066	54.331	0.230	2.913	0.436	0.066
**FCHO2**	0.335	0.398	0.406	1.086	0.064	41.103	-0.145	3.866	-0.553	0.021
**FDX1**	-0.143	7.743	0.665	0.264	0.083	49.127	0.232	2.415	0.181	1.977
**FECH**	-0.106	37.036	0.675	1.289	-0.175	14.770	-0.254	2.913	-0.397	0.119
**FEM1C**	-0.184	51.013	-0.356	3.205	-0.416	0.856	-0.708	5.543	-1.126	0.021
**FES**	-0.133	52.827	0.399	0.264	0.185	1.556	0.201	27.828	0.656	0.021
**FGD2**	0.156	9.997					0.558	0.061	0.668	0.021
**FGD4**	0.490	0.052	-0.559	1.663	0.079	41.103	-0.722	0.061	-0.066	38.751
**FGD6**			-0.706	0.569	0.352	33.124	-0.643	11.241	0.401	0.471
**FGF10**	0.178	18.587	0.775	0.230	0.080	33.124	-0.062	41.825	0.249	40.660
**FGF11**	0.127	49.540	0.387	0.305	0.189	3.475	0.034	50.493	0.120	41.258
**FGF13**	-0.159	4.284	0.667	0.569	0.066	49.127	-1.155	0.061	-0.666	0.021
**FGF19**	0.158	12.647	-0.285	3.009	0.294	3.068	-0.596	4.906	0.402	0.307
**FGF5**	-0.842	0.052	0.482	0.445	-0.699	1.556	-0.736	1.878	-0.670	0.993
**FGF9**	0.228	37.036	0.573	0.107	-0.110	20.025	-0.054	47.420	-0.555	0.269
**FGFR2**	-0.149	1.431	0.553	0.669	0.309	0.440	0.476	1.617	-0.109	24.398
**FGFRL1**							-0.091	47.420	0.438	0.418
**FHDC1**	0.470	0.170			-0.209	6.932	-0.383	1.617	0.172	33.879
**FHOD1**	0.086	49.540	0.217	2.300			0.861	0.947	0.518	0.039
**FIGF**	0.208	4.284	0.208	7.537	0.133	49.127	0.649	0.121	0.300	11.001
**FKBP10**					0.092	22.899	0.249	7.099	0.455	0.901
**FKBP15**	-0.393	0.052	-0.339	0.944	-0.363	1.556	0.384	1.878	-1.116	0.021
**FKBP5**	-0.489	7.743	0.298	5.829	0.238	55.771	0.817	8.987	0.588	0.802
**FKTN**	0.065	49.540	0.453	0.784	0.159	2.056	0.093	49.358	-0.373	0.119
**FLNC**	0.142	7.743	0.240	3.205	-0.095	41.103	0.130	49.358	1.307	0.021
**FLRT3**	-0.439	0.666	0.571	1.457	-0.278	36.892	-0.356	2.913	-0.388	8.002
**FLT1**	0.029	52.218	0.475	0.602	0.045	54.331	0.237	0.855	0.399	0.993
**FLT3LG**	0.016	56.513	-0.436	0.784	-0.248	1.819	-0.306	16.936	-0.187	11.001
**FLVCR1**	0.396	0.780	0.450	0.264	0.154	4.733				
**Fmn1**	-0.532	0.052	-0.244	4.122	0.212	49.127	-0.044	47.420	0.378	24.398
**FMNL2**	-0.428	0.052	-0.295	3.205			0.124	19.971	-0.226	11.001
**FMNL3**					0.056	54.331	0.141	23.450	0.621	0.875
**FNTB**	0.275	5.928	0.377	4.122	0.091	33.124	-0.486	0.341	0.243	24.398
**FOS**	0.973	0.052	0.682	0.107	0.194	0.641	0.264	0.506	-0.102	24.621
**FOSB**	0.200	37.036	0.187	11.093	0.484	0.092	0.204	1.332	0.003	44.315
**FOSL2**	0.408	3.157	0.978	0.107	0.562	0.092	0.182	27.828	-0.350	0.166
**FOXC2**	-0.915	0.052			0.151	33.124	-0.192	50.493	0.344	11.001
**FOXF2**	-0.079	57.293	0.824	0.986	0.171	20.025	-0.640	0.855	-0.568	0.021
**FOXN3**	-0.213	0.780	0.654	0.784	-0.501	0.517	0.636	13.893	0.530	0.045
**FOXO1**	-0.089	33.231	0.856	0.484	0.078	36.892	0.588	0.061	0.435	0.891
**FOXP2**	-0.352	1.098	-0.122	9.629	-0.625	0.856	0.238	1.210	-0.217	8.002
**FPGS**	-0.473	0.666	-0.182	0.784			0.150	41.825	0.299	11.001
**FRAS1**	-0.525	0.171	-0.482	1.457	0.014	60.231	-0.777	0.061	-0.639	0.993
**FRMD5**	0.128	29.668	0.358	5.829	0.104	49.127	-0.265	0.341	-0.442	0.471
**FRRS1**	-0.079	44.812	0.477	2.614	0.114	8.121	0.108	53.272	0.760	0.039
**FRZB**	-0.151	51.013	0.641	1.029	-0.177	4.733	0.430	0.506	-0.536	0.119
**FSTL5**	-0.642	0.262	-0.230	2.859	-0.339	10.694	-0.184	27.828	-0.712	0.129
**FTO**	0.356	18.587	0.415	0.354	-0.118	49.127	-0.241	32.497	-0.181	2.886
**FUBP1**	-0.312	3.157	0.437	1.086	0.524	9.255	0.311	51.354	-0.478	0.066
**FUT8**			0.493	5.829	0.139	5.705			-0.278	6.001
**FXYD1**	0.089	53.690	-0.326	2.859	0.247	1.309	-0.135	5.543	0.793	0.021
**FXYD5**	0.077	33.231					0.135	27.828	0.524	0.021
**FXYD6**			0.255	2.123	0.395	1.556	-0.040	53.829	0.653	0.471
**FYB**	-0.045	52.218	-0.268	3.205	0.027	58.074	0.306	1.617	0.447	0.021
**FYCO1**	-0.428	0.052	0.772	1.534	-0.349	9.255	-0.253	5.543	-0.112	0.129
**FZD10**	0.215	0.666	0.709	0.569	-0.035	59.793	0.025	53.272	0.241	33.879
**FZD4**	-0.234	1.431	-0.262	2.859	0.105	55.771	0.672	0.250	-0.205	0.649
**FZD9**	-0.209	29.668	-0.434	4.122	0.057	54.331	0.276	3.866	0.521	0.021
**G2E3**	0.227	37.036	0.354	0.204	0.323	1.819	-0.554	0.130	0.221	31.624
**G3BP1**	0.096	25.771	0.321	0.267	0.143	17.386	0.278	19.971	-0.512	0.307

**G3BP2**	-0.078	49.540	-0.150	8.253	-0.147	10.694	0.075	49.358	-0.477	0.021
**GABBR1**	0.087	44.812	-0.400	2.773	0.279	49.127	0.808	0.061	0.259	4.218
**GABPA**	0.196	7.743	0.620	0.514	0.044	54.331	-0.390	0.353	-0.487	0.045
**GABRA1**	-0.380	0.052	-1.102	0.107	-0.240	17.386	-0.835	0.727	-0.333	0.307
**GABRA2**	-0.381	0.052	-0.409	2.123	-0.135	41.103	-0.195	27.828	-0.440	0.307
**GABRA3**	0.029	54.701	-0.970	2.773	0.458	0.641	-0.376	3.866	-0.136	8.002
**GABRA4**	-0.046	54.154	-0.562	1.212	-0.235	10.694	-0.214	41.825	-0.389	0.021
**GABRA5**	0.089	44.812	0.427	2.123	-0.238	8.121	-0.106	16.936	-0.446	0.021
**GABRG2**	-0.187	33.231	0.401	5.829	-0.734	17.386	-0.511	27.828	-0.704	0.021
**GABRG3**	-0.042	53.690	-0.230	3.009	-0.447	0.092	-0.634	0.061	0.054	42.030
**GABRR1**	-0.045	54.701	-0.369	0.422	-0.022	60.505	-0.088	47.420	-0.057	31.624
**GAD2**	-0.227	0.529	0.388	0.784	-0.426	0.659	-0.440	1.332	-0.268	4.218
**GADD45B**	0.526	0.052	0.207	1.289	0.178	3.475	-0.109	41.825	0.529	0.993
**GADD45G**	0.171	3.231	0.492	0.986	0.075	36.892	0.046	47.420	0.082	31.624
**GADD45GIP1**	0.455	0.930	0.460	0.829	-0.044	57.395	0.142	13.893	0.204	11.001
**GAL3ST1**	-0.101	56.765	0.224	1.663	0.477	0.240			-0.101	36.831
**GALC**	0.852	0.093	-0.227	2.859	0.223	3.068	0.100	27.828	0.283	31.624
**GALK2**	0.529	0.052			0.059	57.395	-0.187	13.893	-0.087	36.831
**GALNT1**	-0.379	0.511	0.114	8.253	-0.231	1.819	0.256	47.420	-0.835	0.021
**GALNT10**	-0.459	0.666	0.215	1.534	-0.139	41.103	0.277	41.825	0.526	0.993
**GALNT11**	0.135	29.668	-0.497	4.122	-0.024	59.432	-0.232	1.210	-0.478	0.119
**GALNT12**	-0.431	1.431	-0.256	4.122	0.358	1.819	-0.632	0.061	0.740	0.021
**GALNT13**	-0.204	1.098	0.656	0.514	-0.136	49.127	-0.206	0.573	-0.384	0.993
**GALNT14**	0.423	0.431	-0.196	3.009	-0.116	29.328	-0.289	41.825	0.338	24.398
**GALNT3**	0.205	33.231	-0.774	1.086	-0.714	0.641	0.094	51.354	-0.344	24.398
**GALNT6**	0.482	0.052	0.218	0.514	0.230	49.127	0.270	41.825	0.289	24.398
**GALNTL2**	-0.459	0.126			-0.221	26.120	0.941	0.130	1.338	0.021
**GALNTL4**	-0.487	0.052	0.710	1.457	-0.508	0.092	-0.307	2.415	-0.879	0.021
**GALT**	-0.163	25.771	-0.495	0.595	0.024	60.505	0.031	53.272	-0.116	14.822
**GARNL3**	-0.175	1.936	-0.705	2.859	0.369	0.240	-0.533	16.936	0.425	0.391
**GATA2**	-0.276	4.284	0.689	0.602	0.438	10.694	-1.213	0.121	0.595	0.021
**GATA6**	-0.519	1.936	0.646	0.514	-0.114	22.899	-0.163	23.450	-0.178	31.624
**GATM**	-0.258	0.171	0.277	9.629	-0.150	12.575	-0.225	0.573	0.535	0.045
**GBF1**	-0.634	0.093	-0.434	0.484	-0.344	1.819	0.065	52.656	-0.027	38.751
**GCC2**	0.132	51.013	0.462	1.212	0.041	55.771	0.421	0.353	-0.476	0.021
**GCN1L1**	0.307	0.529	0.430	1.457	-0.687	0.092	-0.490	1.332	0.249	0.391
**GCNT1**	0.629	0.052	-0.214	3.205	0.346	9.255	-0.351	49.358	1.105	0.021
**GCNT2**	-0.776	0.052			0.117	29.328	-0.691	7.099	-0.255	24.398
**GEN1**	0.028	55.870	0.645	0.180	-0.129	36.892	0.632	0.061	0.079	33.879
**GFAP**									1.808	0.021
**GFM1**	0.171	25.771	0.782	0.267	0.110	26.120	-0.300	1.617	-0.462	0.021
**GFRA1**	-0.467	0.780	-0.169	0.514	-0.360	0.092	-0.656	0.061	-0.432	0.993
**GGT5**	-0.475	2.450	0.180	0.595			0.975	0.061	0.833	0.021
**GGTA1P**	-0.164	29.668	-0.154	4.122	0.360	22.899	0.469	0.353	0.744	0.891
**GHR**	-0.748	0.780	-0.232	1.663	0.347	0.659	-1.725	0.061	0.607	0.084
**GIGYF1**	0.126	44.812	-0.497	2.614	0.063	54.331	-0.545	0.061	0.138	24.398
**GJA3**			-0.488	0.422	-0.058	54.331	0.064	49.358	0.172	31.624
**GJA5**	0.255	7.743	-0.250	3.205	0.311	0.724	0.529	0.399	0.278	4.218
**GJB2**	0.034	55.170	0.931	0.180	-0.322	3.068	0.605	16.936	0.601	0.993
**GJB6**	-0.099	44.812	0.290	0.514	0.054	54.331	0.150	8.987	0.637	0.021
**GLA**	0.035	53.690	0.413	0.829	-0.090	49.127	-0.112	47.420	-0.106	33.879
**GLI3**	-0.148	37.036	-0.051	10.404	0.287	29.328	-0.083	50.493	0.824	0.021
**GLIPR2**	0.248	0.324	-0.077	12.522	-0.088	59.432	-0.199	0.250	0.934	0.021
**GLMN**	0.309	0.666			0.016	60.231			-0.499	0.045
**GLRX2**	0.253	0.529	0.477	0.107	-0.041	58.074	-0.083	41.825	0.276	8.002
**GLS**	-0.250	15.278	0.617	0.912	-0.445	1.309	-0.344	13.893	0.616	31.624
**GMFB**	0.402	0.511	0.584	0.174	-0.177	8.121	-0.168	0.947	-0.524	0.066
**GMFG**	-0.043	56.130	0.469	0.180	0.097	49.127	-0.371	0.130	0.646	0.021
**GNAI1**	-0.193	3.157	-0.483	0.107	-0.243	2.659	-0.082	49.358	0.135	24.398
**GNAZ**	0.335	21.797	0.590	0.267	0.143	36.892	-0.157	47.420	-0.388	0.119
**GNB1**	-0.088	54.701	0.455	0.514	0.157	41.103	-0.295	0.727	-0.280	0.269
**GNG11**	-0.194	18.587	-0.507	0.614	0.150	17.386	0.242	1.332	0.364	14.822
**GNS**	-0.067	55.870	-0.929	0.935	0.232	49.127	-0.265	49.358	0.268	24.398
**GOLGA3**	0.464	0.052	0.522	0.514	-0.067	41.103	0.222	1.210	-0.112	31.624
**GOLGA5**	0.319	15.278	0.245	5.829	-0.706	0.092	0.444	0.399	-0.140	24.398
**GOLGA7**	-0.091	55.559	0.531	0.354	0.117	49.127	0.046	49.358	-0.217	1.977
**GOLGB1**	-0.461	0.052	0.428	6.703	-0.539	0.281	0.591	0.130	-0.407	0.084
**GOLPH3**	0.129	44.812	0.711	0.107	0.254	9.255	-0.447	41.825	0.462	0.977

**GOPC**	0.178	44.812	-0.414	3.205	0.306	0.092	-0.682	4.906	-0.225	11.001
**GORAB**	0.178	2.450	-0.077	13.299	-0.234	3.475	-0.121	32.497	-0.501	0.021
**GPAT2**	0.110	18.587	-0.219	1.772	-0.114	49.127			0.471	0.166
**GPC5**	-0.358	1.431	0.792	0.086	-0.558	1.309	0.342	47.420	0.123	44.052
**GPCPD1**	-0.332	0.324	0.227	8.253	-0.131	55.771	0.077	47.420	-0.427	0.021
**GPM6B**	0.078	49.540	-0.357	0.514	-0.356	0.641	-0.110	13.893	-0.350	0.045
**GPNMB**	0.058	44.812	-0.291	0.514			0.144	16.936	1.225	0.129
**GPR108**	-0.061	54.154	-0.430	3.205	0.017	58.406	0.095	23.450	0.395	0.119
**GPR124**	-0.038	55.870	-0.417	4.122	0.397	0.641	0.041	53.272	0.168	33.879
**GPR156**	-0.186	0.324	-0.522	0.514	-0.067	41.103			0.269	11.001
**GPR160**	-0.785	0.052			-0.163	49.127	0.383	8.987	0.849	0.021
**GPR17**	0.109	49.540	-0.161	5.829	0.071	54.331	-0.042	51.354	0.562	0.021
**GPR179**	-0.459	0.052	-0.558	2.859	0.130	36.892	0.191	4.906	0.171	6.001
**GPR26**	-0.509	0.930	0.180	7.537	-0.414	5.705	-0.622	41.825	-0.254	14.822
**GPR37**	-0.072	49.540	-0.306	5.829	0.023	58.406	-0.473	0.061	-0.291	0.746
**GPR37L1**	-0.132	7.743	-0.084	5.829	0.040	49.127	0.162	27.828	0.479	0.746
**GPR39**	-0.091	44.812	-0.665	0.514			0.069	47.420	0.238	24.398
**GPR4**	-0.033	56.130	-0.467	2.859	-0.180	8.121	-0.517	0.061	0.087	37.824
**GPR56**			0.291	0.944			0.185	41.825	0.572	0.129
**GPR6**	-0.130	21.797	-0.210	0.784	0.036	54.331	0.423	0.130	-0.589	0.021
**GPR64**	0.069	37.036	0.570	0.986	0.066	54.331	0.298	41.825	-0.299	36.831
**GPR84**	-0.182	5.928	0.488	0.514					1.566	0.021
**GPR85**			0.627	0.669	-0.089	33.124	0.175	1.332	-0.532	0.021
**GPR88**	-1.331	0.052	-0.425	4.122					0.093	42.369
**GPR98**	0.074	37.036	-0.362	3.205	0.132	5.705	0.427	0.573	-0.067	31.624
**GPRC5C**	-0.137	2.450	-0.353	3.009	0.276	0.659	-0.289	32.497	0.666	0.021
**GPSM3**	0.618	0.052	0.178	5.829	0.141	9.255	-0.425	0.506	0.567	0.471
**GPX3**			0.244	5.829	-0.563	0.092	-0.320	0.250	0.494	0.039
**GRAMD3**	-0.100	33.231	-0.308	8.253	0.091	26.120	0.523	0.353	0.411	0.891
**GRAP**	0.052	52.218	-0.393	0.354	0.077	41.103			0.589	0.021
**GRB7**	-0.146	52.218	-0.201	3.205	-0.134	12.575	0.375	0.109	0.455	0.886
**GREB1L**			-0.516	0.602			-0.160	2.913	0.312	0.269
**GREM1**	-0.241	49.540	0.102	6.703	0.503	0.440	-0.643	7.099	0.607	31.624
**GRIA2**	0.252	2.450	0.593	0.420	-0.322	36.892	-0.226	27.828	-0.268	1.193
**GRIA3**	-0.473	0.052	0.446	1.663	-0.187	6.932	-0.204	0.727	-0.656	0.021
**GRIA4**	-0.557	0.052	0.398	4.122	-0.785	0.402	-0.598	8.987	-0.449	0.129
**GRID1**	-0.369	0.511	0.329	1.534	0.088	57.395	-0.915	0.061	-0.614	0.021
**GRIK4**	-0.649	0.052	0.305	0.944	-0.123	58.074	0.452	1.617	-0.651	0.746
**GRIN1**	-0.031	54.154	-0.160	4.122	0.116	22.899	-0.569	0.061	-0.171	24.398
**GRIN2B**	-0.084	44.812	-0.537	0.514	-0.134	1.309	-0.376	3.866	-0.424	0.993
**GRIP1**	-0.127	29.668	0.352	3.009	-0.226	2.056	-0.155	19.971	-0.533	0.119
**GRK4**	0.020	57.109	-0.396	0.309	0.024	58.406	0.106	41.825	0.305	0.977
**GRK5**	0.025	52.827	0.370	0.514	0.248	5.705	0.594	16.936	-0.630	0.119
**GRK6**	0.133	29.668	-0.416	0.602	-0.116	54.331	0.312	32.497	0.059	42.554
**GRM5**	-0.643	0.262	0.343	2.300	-0.578	6.932	-0.228	11.241	-0.164	24.398
**GSDMA**	0.139	2.450	-0.480	0.944	-0.219	26.120	0.052	47.420	0.329	31.624
**GSDMD**	0.209	2.450	-0.064	11.093	0.250	9.255	-0.406	0.353	0.843	0.021
**GSG1L**	0.077	49.540	0.460	0.180	0.231	36.892			0.002	44.315
**GSK3A**	0.305	3.157	0.306	5.829	0.029	57.395			0.419	0.391
**GSN**	0.229	4.284	0.121	9.629			-0.390	0.399	0.904	0.021
**GSR**	-0.054	49.540	0.336	2.300	0.031	58.611	0.460	0.399	0.105	40.660
**GSTK1**	-0.236	0.666	-0.627	1.663			0.246	3.866	0.268	24.398
**Gstm3**	-0.166	37.036	-0.164	4.122	0.158	36.892	0.087	27.828	0.580	0.021
**GSTM5**	-0.175	29.668	-0.175	3.205	0.156	20.025	0.097	41.825	0.561	0.021
**GTDC1**	-0.416	0.052	-0.217	5.829	0.185	6.932	0.165	8.987	-0.378	0.269
**GTF2IRD1**	-0.487	0.052	0.413	5.829	-0.623	9.255	-0.358	1.332	-0.131	24.398
**GTF3C4**	0.392	0.052	-0.580	3.205	0.134	6.932	0.064	50.493	0.131	33.879
**GUCY1A3**	-0.890	0.052	-0.400	3.009	0.510	1.309	0.291	41.825	-0.731	0.021
**GUSB**	0.041	54.154	-0.189	5.829	-0.086	58.074	0.132	27.828	0.626	0.021
**GZMM**			0.383	7.537	0.074	41.103	-0.214	19.971	-0.645	0.039
**H1FX**	1.142	0.171	-0.119	5.829	0.250	12.575	0.105	41.825	0.449	0.746
**H6PD**	-0.244	0.398	-0.360	0.829	-0.170	2.311	0.097	19.971	-0.058	33.879
**HADH**	-0.888	0.052	-0.489	3.009	0.124	10.694	0.814	0.061	0.969	0.021
**HAO1**	-0.493	0.511	0.413	0.107	0.185	4.053	-1.031	1.332	-0.866	0.021
**HAPLN1**	-0.478	0.170	0.917	0.300	0.116	41.103	1.355	0.061	-0.868	0.021
**HAS1**	-0.389	0.171	-0.176	4.122	-0.567	0.092	0.966	1.617	-0.433	0.891
**HAS3**	0.488	0.052	0.547	0.877	0.211	0.856	0.132	47.420	-0.481	0.021
**HBEGF**	0.121	12.647	0.253	5.829	0.174	3.475	-0.422	0.177	0.363	8.002

**HCFC1**	-0.747	0.052	-0.366	5.829	0.322	5.705	0.260	4.906	-0.121	24.398
**HCFC2**	0.129	37.036	0.520	2.773	-0.120	20.025	-0.205	0.250	0.492	0.993
**HCK**	0.539	0.170	-0.366	6.703	0.127	4.053			0.978	0.021
**HCLS1**	0.019	56.130	-0.277	2.859	0.076	29.328	0.109	47.420	0.567	0.066
**HCN3**	-0.364	15.278	-0.464	2.773	-0.693	0.092	0.935	0.130	-0.146	35.531
**HCRTR2**	-0.849	0.171	-0.432	4.122	-0.071	57.395	-0.720	1.617	-0.790	0.021
**HCST**	-0.053	57.367	-0.150	0.784	0.297	2.056	-0.476	0.061	1.244	0.021
**HDAC7**	-0.511	0.052	-0.286	3.009	0.163	20.025	-0.235	27.828	0.288	31.624
**HDAC8**	-0.157	3.157	0.290	8.881	0.143	49.127	-0.288	11.241	-0.750	0.021
**HDAC9**	-0.536	0.052	-0.259	0.784	-0.641	0.092	-0.595	0.250	-1.016	0.021
**HDC**	-1.365	0.052	0.759	0.300	0.494	12.575	-0.063	50.493	1.183	0.021
**HDX**	-0.253	54.701	-0.428	4.122	-0.389	0.402				
**HEATR3**	0.226	0.171	0.529	3.205	0.147	22.899	0.459	0.506	-0.926	0.021
**HECTD2**	0.060	44.812	0.283	1.663	-0.390	0.092	-0.652	0.947	-0.638	0.045
**HECW1**	-0.371	0.170	-1.106	2.614	0.121	49.127	0.048	51.354	0.282	24.398
**HECW2**	0.180	18.587	0.632	0.784	0.138	49.127	0.682	0.947	-0.540	0.129
**HEG1**	0.368	0.171	-0.316	3.205	-0.132	54.331	-0.745	0.061	0.255	0.977
**HELB**	0.238	4.284	-0.125	5.829	-0.266	8.121	0.172	41.825	-0.641	0.021
**HELQ**	0.225	37.036	-0.287	4.122	-0.442	2.311	-1.137	0.061	0.034	43.609
**HERC1**	0.353	1.936	0.352	3.205	-0.556	0.092	-0.677	0.061	-0.122	14.822
**HEXB**	0.064	44.812	0.154	1.663	-0.133	9.255	0.161	19.971	0.579	0.021
**HFE**	0.632	0.171	0.149	5.829	0.569	2.056	0.376	8.987	0.620	0.021
**HGF**	0.065	44.812	-0.453	0.122	0.470	0.440	0.025	53.829	-0.043	44.052
**HHAT**	0.114	25.771	-0.293	4.122	-0.085	36.892	-0.561	0.061	-0.065	40.051
**HHEX**									1.435	0.021
**HIP1**	0.200	37.036	0.283	3.009	0.065	41.103	-0.077	49.358	-0.618	0.021
**HIVEP3**	-0.769	0.126	-0.370	4.122	-0.253	3.068	-0.864	0.061	-0.382	0.084
**HJURP**	-0.787	0.126	-0.923	3.205	0.304	33.124	-0.640	23.450	-0.745	0.166
**HK2**	0.179	4.284	-0.405	5.829	0.030	56.823	0.084	52.124	0.840	0.021
**HLA-B**	1.142	0.171	0.314	2.123	0.250	12.575	0.105	41.825	0.503	0.021
**HLA-C**	0.114	44.812	-0.119	5.829	-0.179	20.025	-0.360	0.061	0.981	0.021
**HLA-DMB**	0.424	3.157	-0.350	0.267	0.711	0.402	0.550	23.450	0.888	0.021
**HLA-DOA**	-0.322	5.928			0.458	4.053	0.338	32.497	0.666	0.166
**HLA-DQB1**	0.322	44.812	0.241	6.703	0.283	0.641	-0.191	47.420	0.706	0.021
**HLA-E**	0.198	21.797	0.234	2.773	0.128	22.899			0.399	0.119
**HLA-G**	0.197	9.997	-0.191	4.122	-0.525	0.092	-0.219	4.906	0.579	0.021
**HMCN1**	-0.259	0.930			-0.566	0.440	0.052	52.656	0.534	0.066
**HMGA2**	-0.085	57.293	-0.527	0.264	-0.238	20.025	-0.242	13.893	-0.096	39.468
**HMGCR**	0.309	44.812	0.663	0.669	-0.376	0.092	0.327	47.420	-0.919	0.084
**HMGXB4**	0.252	0.170	0.780	0.569	0.562	0.092	0.134	41.825	-0.145	24.398
**HMMR**	0.109	49.540	-0.712	0.514			-0.466	32.497	-0.256	14.822
**HN1L**	-0.319	49.540	0.233	4.122	-0.451	0.092	0.395	1.210	0.787	0.391
**HNRNPAB**	-0.123	44.812	0.455	1.212	-0.115	26.120	-0.736	0.121	-0.758	0.021
**HNRNPC**	-0.214	18.587	0.643	1.663	-0.187	10.694	-0.179	0.399	-0.524	0.021
**HNRNPR**	-0.199	44.812	0.568	1.663	-0.243	2.311	-0.328	5.543	-0.364	0.166
**HNRPDL**	0.132	44.812	0.664	1.772	-0.056	57.395	-0.134	41.825	-0.336	0.039
**HOMER1**	0.311	0.930	0.633	0.986	-0.166	26.120	-0.397	0.573	-0.287	0.391
**HOXA7**	-0.072	49.540	-0.350	0.986	0.043	54.331	0.045	50.493	-0.007	24.398
**HPS3**	-0.885	0.052	0.611	0.180	-0.579	1.819	0.072	49.358	-0.689	0.021
**HPS6**	0.140	7.743	0.335	1.772	0.039	55.771	0.565	0.061	0.122	31.624
**HS1BP3**	0.166	5.928	-0.144	8.253	0.486	0.240	-0.432	7.099	0.877	0.021
**HS2ST1**	0.170	7.743	0.753	0.602	0.157	3.475	0.169	7.099	-0.374	6.001
**HS3ST1**	0.456	0.529	-0.398	4.122	-0.218	22.899			0.072	40.660
**HS3ST3B1**	-0.219	9.997	-0.116	11.783	-0.292	0.856	0.243	49.358	0.512	0.021
**HSD3B7**	-0.230	4.284	0.111	2.859	-0.075	54.331	-0.376	1.617	0.446	0.084
**HSP90B1**	0.092	37.036	-0.511	2.123	-0.478	0.092	0.134	47.420	-0.018	41.878
**HSPA12A**	0.265	0.052	-1.387	0.204	0.233	4.053	0.263	1.617	-0.295	0.166
**HSPA2**	-0.115	12.647	0.705	0.180	-0.160	14.770	0.146	3.866	-0.413	4.218
**HSPA9**	0.142	44.812	0.738	0.986	-0.093	54.331			-0.447	0.021
**HSPB1**			0.280	1.772	0.305	0.402	0.241	8.987	0.756	0.021
**HSPB2**	0.140	44.812	0.148	9.629	0.404	0.092	0.594	0.061	0.277	4.218
**HSPB3**	0.305	0.780	-0.473	1.457	0.146	29.328	0.406	16.936	0.484	0.039
**HSPB6**	0.267	1.098			0.222	17.386	0.060	49.358	0.966	0.021
**HSPB8**	-0.226	49.540	0.312	2.859	0.918	0.402	0.404	0.130	0.868	0.891
**HTATIP2**	0.475	0.780	0.407	1.534	0.164	1.819	0.202	16.936	0.073	42.670
**HTR3A**	0.288	18.587	0.394	2.773	0.053	56.823	0.167	7.099	-0.678	0.045
**HTR5B**	0.139	44.812	0.490	0.614	0.334	1.098	0.027	52.656	0.653	0.993
**HTR6**	-0.193	25.771	-0.555	0.784	-0.296	5.705	0.026	54.831	0.407	0.084

**HUS1**	-0.385	0.431	-0.170	6.703	-0.314	29.328			0.045	42.836
**HUWE1**	-0.177	3.157	-0.402	0.784	-0.721	0.641	-0.300	41.825	0.168	31.624
**HYAL3**	-0.434	9.997	-0.109	6.703	0.134	29.328	0.325	1.617	0.694	0.021
**HYI**	-0.304	0.262	-0.265	4.122			0.271	3.866	0.587	0.269
**HYOU1**	-0.104	49.540	-0.439	3.205	0.075	49.127	0.079	53.272	0.448	0.039
**IARS2**	0.094	44.812	0.545	0.663	-0.364	2.659	0.073	49.358	0.138	39.468
**ICAM1**	0.035	51.013	-0.227	3.205	0.352	2.056	0.531	0.341	1.175	0.021
**ICAM2**	0.040	49.540	0.254	7.537	0.300	0.641	-0.971	0.061	-0.021	44.315
**ID1**					0.219	22.899	0.091	32.497	0.994	0.021
**ID2**	0.026	55.170	0.419	5.829	0.435	0.724	0.181	41.825	0.171	24.398
**ID3**	-0.283	49.540	-0.144	4.122	0.080	33.124	0.450	0.130	0.736	0.021
**IDH2**	0.385	0.450	0.271	5.829	0.170	1.309	0.206	47.420	0.176	4.218
**IDH3A**	0.079	44.812	0.435	4.122	-0.086	41.103	-0.102	41.825	-0.425	0.021
**IER2**	0.502	0.052	0.423	0.514	0.285	1.556	0.458	11.241	-0.391	0.649
**IER3**	0.477	0.171	0.345	5.829	0.528	1.098	0.091	41.825	0.429	0.021
**IFI27L2**	0.057	44.812	-0.168	4.122	0.013	59.432	1.151	0.341	1.482	0.021
**IFI30**	0.184	15.278	0.074	8.881	0.283	3.068	-0.229	19.971	0.468	0.213
**IFIH1**	-0.179	44.812	0.218	8.253			0.708	0.061	0.674	0.021
**IFIT3**			0.629	0.986	-0.101	49.127	-0.296	0.061	0.901	0.213
**IFITM2**	0.033	52.218	-0.580	0.514	0.218	2.311	-0.155	8.987	0.656	0.021
**IFITM3**	-0.398	0.529	0.106	11.093			0.319	0.573	1.191	0.021
**IFNGR1**			-0.250	6.703	0.084	36.892			0.636	0.021
**IFT140**	-0.432	0.052	0.235	5.829	-0.064	49.127	0.074	41.825	0.263	31.624
**IGF1**	0.800	0.093	0.166	8.253	0.125	54.331	0.509	0.061	0.289	24.398
**IGF1R**	0.392	0.093	0.287	1.457	-0.470	41.103	0.049	50.493	-0.129	33.879
**IGF2**	-0.640	0.052	0.413	0.514	0.137	14.770	0.058	53.272	0.337	0.471
**IGFBP2**	-0.130	49.540	-0.098	8.881	0.186	26.120	0.159	3.866	0.577	0.021
**IGFBP3**	0.245	0.262	0.438	0.944	-0.562	0.092	0.144	19.971	-0.392	0.119
**IGFBP5**	-0.369	2.450	-0.278	3.205	0.155	12.575	0.399	0.947	1.287	0.021
**IGFBP6**	0.228	9.997			0.369	2.056	0.189	2.415	0.733	0.129
**IGFBPL1**	-0.062	44.812	-0.229	6.703	-0.054	49.127	0.362	1.210	0.449	0.802
**IGHA1**	-0.133	37.036	-0.938	0.663	-0.395	29.328	-0.274	2.913	0.423	11.001
**IGHM**	-0.072	44.812	-0.512	0.784	-0.208	0.856	0.209	0.727	0.443	0.084
**IGSF3**	-0.575	0.052	-0.510	0.445	-0.269	9.255	0.117	16.936	0.151	33.879
**IGSF6**	-0.307	7.743	-0.133	5.829	-0.186	54.331	-0.146	23.450	0.840	0.021
**IL10RA**	0.344	0.171	0.408	0.107	0.443	1.309	-0.370	32.497	0.624	0.021
**IL10RB**	0.256	9.997	0.456	2.123	0.113	8.121	0.374	0.061	0.590	0.021
**IL11RA**	-0.127	3.157	-0.356	3.205	0.237	9.255	-0.215	49.358	0.459	0.471
**IL15RA**	-0.063	33.231	-0.603	0.614	-0.048	54.331	-0.057	41.825	0.189	31.624
**IL16**	-0.469	0.052	-0.162	4.122	-0.620	2.311	0.434	13.893	-0.583	0.269
**IL17F**	0.463	0.157	-0.087	8.253	0.031	56.823	-0.135	32.497	0.010	44.052
**IL18BP**	-0.181	18.587	-0.284	3.205	0.159	22.899	-0.347	1.878	0.563	0.021
**IL1RAP**	-0.423	0.052	0.410	0.514	-0.507	0.092	-0.525	1.332	-0.482	0.993
**IL20RA**	-0.454	44.812	-0.390	6.703	0.721	0.092	0.223	54.831	-0.893	0.891
**IL20RB**	-0.714	0.093	0.462	0.354	0.473	0.092	-0.886	0.061	0.396	31.624
**IL2RA**	-0.477	0.052	0.331	5.829	0.024	59.432	-0.354	4.906	0.882	0.021
**IL33**	-0.164	3.157	-0.140	11.093	0.047	54.331	0.143	5.543	0.444	0.213
**IL3RA**	0.067	44.812	-0.119	9.629	-0.098	54.331	-0.144	27.828	0.387	0.166
**IL4I1**	-0.055	51.013	-0.080	11.093	0.038	54.331	0.281	4.906	0.679	0.021
**IL4R**	0.200	15.278			0.537	0.733	0.063	52.656	0.276	11.001
**INADL**	-0.372	1.098	-0.663	2.614	0.156	54.331	-0.705	0.573	0.645	0.021
**INHA**	-0.067	44.812	0.347	2.123	0.088	54.331	0.136	41.825	-0.907	0.021
**INHBA**	-0.232	21.797	0.287	5.829	0.462	0.092	0.074	19.971	0.377	14.822
**INPP5D**					-0.073	56.823	0.340	7.099	1.004	0.021
**INPP5K**	-0.378	2.450	-0.641	0.935	-0.084	54.331	0.083	47.420	0.182	36.831
**INSIG1**	-0.489	0.126	0.339	3.009	-0.380	5.705	-0.474	27.828	-0.659	0.021
**INSM1**	0.165	29.668	0.196	7.537	0.206	8.121	-0.146	16.936	-0.506	0.166
**INTS3**	-0.073	44.812	-0.542	3.205	0.051	58.074	0.116	27.828	0.510	0.471
**IP6K1**	-0.087	52.218	-0.097	10.404	0.078	49.127	0.583	0.061	-0.226	1.193
**IPMK**	0.213	33.231	0.612	0.107	0.148	12.575	-0.161	4.906	0.328	14.822
**IPO5**	0.260	12.647	0.818	0.877	-0.099	33.124	0.186	23.450	-0.404	0.021
**IQGAP1**	-0.495	0.052	-0.226	4.122	0.067	29.328	-0.126	54.403	-0.179	33.879
**IQGAP3**	-0.393	12.647	-0.205	0.784	0.635	0.092	0.876	8.987	0.926	0.649
**IRAK1**	0.030	55.870	0.420	1.772	-0.176	47.420	-0.123	6.932	-0.589	0.166
**IRF1**	-0.147	1.098	0.215	7.537	0.141	3.068	0.151	47.420	0.475	0.021
**IRF5**	-0.125	52.218	-0.363	2.859	0.289	41.103	0.275	23.450	0.831	0.021
**IRF8**	-0.424	18.587	0.191	8.253	-0.054	58.074	0.287	1.617	0.688	0.021
**IRF9**			0.549	2.614			-0.207	1.332	0.592	0.021

**IRGM**	0.269	0.262	0.597	0.595	0.081	26.120	0.269	4.906	0.473	0.471
**Irgm2**	0.029	56.874	0.287	0.300	0.327	2.659			0.761	0.021
**ISG15**	-0.135	44.812	0.275	1.086	0.336	0.281	0.102	53.272	0.487	0.129
**ISL1**	-0.935	0.529	-0.338	2.859	0.081	49.127	0.139	19.971	-0.151	24.398
**ISLR**	-0.531	0.666	-0.140	5.829	0.091	36.892	-0.149	52.124	0.397	0.993
**ISOC1**	-0.315	7.743	0.331	3.205	-0.086	41.103	-0.244	7.099	-0.505	0.119
**ITGA3**	0.413	5.928	-0.673	2.614	-0.223	2.311	0.076	53.829	-0.435	0.307
**ITGA4**	0.162	29.668	-0.988	0.107	-0.238	12.575	-0.784	1.878	0.380	0.021
**ITGA7**	0.668	0.052	-0.327	0.944	-0.349	20.025	0.294	47.420	-0.191	31.624
**ITGA9**	-0.292	44.812	-0.174	2.614	-0.133	22.899	-0.636	5.543	0.446	0.269
**ITGAX**	-0.082	37.036	-0.343	0.784	0.010	60.231	-0.025	54.403	1.220	0.021
**ITGB1**	0.288	0.780	0.602	0.180	0.079	36.892	0.259	4.906	0.116	24.398
**ITGB2**	-0.114	7.743	-0.276	0.784	0.014	59.432	-0.115	27.828	0.576	0.021
**ITGB3**	-0.225	5.928	-0.393	0.784	0.439	12.575	-1.062	0.061	0.670	0.021
**ITGB4**	-0.437	18.587	0.098	11.093			0.509	0.061	0.313	24.398
**ITGB5**	-0.066	54.701	-0.378	2.614	-0.032	58.406	-0.087	49.358	0.677	0.021
**ITIH3**	-0.232	9.997	0.295	3.009	-0.419	0.281	0.119	11.241	0.605	0.021
**ITIH5**	-0.132	25.771	-0.083	10.404	0.197	9.255	-0.089	27.828	0.490	0.746
**ITPR3**	-0.196	18.587	-0.840	1.772			0.121	47.420	0.646	0.021
**JAK3**	-0.360	21.797	0.221	5.829	0.227	0.641	0.370	1.210	0.617	0.021
**JAKMIP2**			-0.535	0.514	-0.393	1.819	0.065	41.825	-0.446	0.307
**JHDM1D**	0.431	0.052	0.390	2.300	0.338	2.659	-0.467	0.130	0.608	0.021
**JMJD7-PLA2G4B**	0.291	0.126	0.657	0.107	0.318	0.402	-0.106	47.420	-0.711	0.021
**JMY**	0.016	56.130	-0.406	3.205	-0.058	58.074	-0.401	7.099	-0.557	0.021
**JTB**	0.031	56.130	0.472	0.107	0.018	58.611	0.113	41.825	0.085	31.624
**JUN**	-0.359	0.398	0.322	0.595	0.311	0.517	0.080	41.825	1.011	0.021
**JUND**	0.153	33.231			0.114	41.103	0.324	19.971	0.503	0.039
**KATNAL1**	-0.102	37.036			0.151	49.127	0.230	47.420	-0.602	0.269
**KATNAL2**	-0.234	0.093	0.598	1.289	0.056	54.331	0.184	23.450	-1.148	0.021
**KAZN**	-0.382	0.431	0.233	2.300	0.189	4.053	0.141	49.358	0.464	0.307
**KCNC1**	0.194	49.540	-0.363	4.122	0.134	20.025	-0.109	27.828	-0.478	0.039
**KCNF1**	-0.568	0.171	0.693	0.264	0.501	49.127	0.058		-0.327	31.624
**KCNG2**	-0.084	55.170					-0.190	16.936	-0.517	0.993
**KCNH1**	-0.398	12.647	0.601	0.514	-0.180	12.575	-0.418	27.828	-0.842	0.021
**KCNH3**	0.057	53.690			0.029	58.074	-0.245	19.971	-0.378	0.269
**KCNJ12**	-0.473	7.743			0.382	1.309	-0.201	52.124	0.637	0.193
**KCNJ16**	-0.030	57.367							-0.573	0.993
**KCNJ3**	0.278	0.398	0.541	2.123	-0.126	41.103	0.130	47.420	-0.493	0.129
**KCNK6**	-0.398	0.052	0.180	0.514	0.048	54.331			0.873	0.021
**KCNMA1**	0.059	49.540	0.343	6.703	-0.107	54.331	-0.095	41.825	-0.400	0.307
**KCNQ1OT1**	-0.493	0.324	-0.101	3.205	-0.486	0.092	-0.430	7.099	-0.789	0.021
**KCNT2**	-0.246	2.450	0.216	4.122	0.079	49.127	0.162	47.420	-0.572	0.066
**KCTD7**	-0.056	52.218	0.571	0.944	-0.239	1.309	-0.380	2.415	0.313	31.624
**KDM2A**	-0.204	0.511	-0.335	4.122	-0.530	0.641	-0.216	5.543	-0.462	0.993
**KDM2B**	0.077	49.540	0.535	0.264	0.203	4.733	0.430	0.341	-0.259	2.886
**KDM5B**	-0.200	1.936	0.415	5.829	-0.182	14.770	-0.376	0.573	-0.354	0.129
**KDM6B**	-0.121	37.036			0.098	41.103	-0.773	0.061	-0.116	24.398
**KDR**	-0.286	0.093	-0.330	4.122	0.138	3.475	-0.103	54.403	-0.832	0.021
**KHDRBS2**	-0.233	7.743	0.105	11.093	0.398	4.053	0.105	23.450	-0.127	44.315
**KIAA0090**	-0.120	44.812	0.506	0.986	0.138	9.255	0.229	7.099	0.139	24.398
**KIAA0101**	-0.670	0.093	-0.930	1.029	-0.493	1.309	-0.612	0.353	0.549	0.021
**KIAA0182**	0.067	49.540	-0.354	3.205	0.133	10.694			0.577	0.119
**KIAA0247**	0.128	12.647	0.201	8.881	0.401	1.098	-0.487	0.061	0.099	33.879
**KIAA0319**	0.058	54.154	0.522	1.534	-0.304	0.092	0.045	47.420	-0.430	0.391
**KIAA0368**	-0.735	0.171	0.566	0.784	-0.189	49.127	0.293	1.617	-1.225	0.021
**KIAA0913**	-0.223	0.780	0.337	2.614	-0.062	54.331	-0.075	41.825	-0.610	0.045
**KIAA0922**	-1.328	0.052	0.402	5.829	-0.639	0.724	-0.476	49.358	-0.421	0.119
**KIAA0947**	0.016	55.559	0.521	3.009	0.039	54.331	-0.229	0.573	-0.414	0.021
**KIAA1009**	-0.512	0.052	-0.505	0.944	0.204	14.770	-0.726	0.855	0.761	0.021
**KIAA1109**	-0.157	12.647	-0.307	6.703	-0.286	2.056	-0.472	0.250	-0.537	0.021
**KIAA1199**	-0.434	0.052	0.656	0.264	0.258	2.056	-0.282	19.971	-0.929	0.993
**KIAA1324**	0.055	49.540	0.762	0.445	0.151	8.121	0.041	49.358	0.157	14.822
**KIAA1377**	0.494	0.171	0.521	2.123	-0.060	58.406	0.099	47.420	-0.294	1.977
**KIAA1456**	0.083	44.812	0.481	0.595			0.117	47.420	-0.193	1.977
**KIAA1804**	-0.167	4.284	0.384	4.122	-0.130	8.121	0.502	0.061	-0.129	8.002
**KIAA1958**	0.191	1.098	0.396	0.514	0.162	26.120	-0.128	41.825	0.419	0.066
**KIF1B**	-0.440	0.052	0.558	1.289	-0.308	4.053	-0.368	47.420	0.361	0.129
**KIF1C**	-0.189	21.797	-0.693	0.534	-0.131	26.120	0.239	11.241	0.223	24.398

**KIF26B**	-0.742	0.052	0.202	0.514			-0.951	0.250	-0.012	44.315
**KIF2A**	-0.259	0.171	0.667	0.300	-0.134	4.053	0.128	47.420	-0.200	4.218
**KIF5C**	-0.300	0.666	0.282	1.289	0.167	49.127	-0.576	7.099	-0.281	8.002
**KIFAP3**	-0.247	0.780	-0.508	1.663	-0.283	0.856	-0.227	1.878	-0.451	0.021
**KIFC3**	0.094	29.668			0.118	8.121	-0.510	0.353	0.343	0.084
**KIT**	-0.829	0.052	0.685	0.204	0.115	41.103	0.154	19.971	-0.763	0.021
**KLC2**	-0.036	52.827	-0.391	0.859	0.033	54.331	0.081	47.420	0.004	44.052
**KLF13**	-0.580	0.666	-1.199	0.935	0.120	36.892	0.091	47.420	0.229	31.624
**KLF3**	-0.163	44.812	0.543	1.289	-0.400	0.240	-0.178	47.420	0.772	0.045
**KLF4**	-0.154		-0.561	4.122	0.402	0.724	-0.128	49.358	0.004	44.052
**KLHDC5**	0.746	0.052	0.569	0.107	0.357	0.092	-0.690	0.061	-0.677	0.066
**KLHL21**	0.370	2.450	0.417	0.784	-0.092	17.386	0.324	0.399	-0.065	31.624
**KLK6**	-0.065	54.701	-0.576	2.614	-0.208	10.694	0.413	1.210	1.188	0.021
**KLK7**	0.317	0.262	-0.405	1.086	0.166	9.255	0.250	41.825	0.656	0.166
**KLK9**	0.084	44.812	-0.245	4.122	0.117	9.255	0.243	32.497	0.453	0.886
**KPNA3**	-0.085	54.701	-0.455	0.514	-0.083	41.103	0.043	53.829	-0.315	0.119
**KPNA4**	0.146	51.013	-0.401	2.859	-0.674	0.092	-0.108	7.099	0.406	0.993
**KPNB1**	0.069	49.540	-0.224	3.009	0.061	54.331	0.098	41.825	0.667	0.066
**KRAS**	-0.161	5.928	0.559	5.829	-0.111	17.386	-0.232	0.573	-0.633	0.021
**KREMEN1**	-0.769	0.398	0.300	2.123	0.291	1.556	-1.616	0.061	0.316	42.670
**KRIT1**	-0.568	0.398	0.418	4.122	-0.116	58.406	-0.310	1.878	-0.775	0.021
**KRR1**	-0.269	0.511	0.885	0.877	0.101	20.025	-0.453	1.617	-0.666	0.021
**KRT2**	-0.319	25.771	0.391	0.445	0.876	0.402	0.453	41.825	-0.350	31.624
**KRT79**	-0.177	52.218	0.385	0.669	0.882	0.517	0.434	0.061	-0.453	24.398
**LAG3**					0.469	3.475	0.038	52.124	1.210	0.021
**LAIR1**	-0.291	0.171	-0.822	0.784	0.362	1.556	-0.728	0.061	1.192	0.039
**LAMA3**	0.446	0.052	-0.199	3.205	0.463	0.092	0.278	1.878	0.204	31.624
**LAMA5**	-0.413	0.052	0.329	5.829	0.053	56.823	-0.380	19.971	0.231	44.203
**LAMB2**	0.058	49.540	0.516	1.772	0.348	1.309			0.423	0.021
**LAMP2**	-0.094	52.218	0.278	1.457	-0.149	6.932	-0.204	23.450	0.460	0.398
**LANCL1**	0.105	29.668	0.677	2.300			-0.292	0.061	-0.493	0.021
**LANCL2**	0.110	52.218	0.414	0.174	-0.101	36.892	0.599	1.878	1.139	0.021
**LAPTM5**	-0.134	3.157			-0.292	33.124			1.024	0.021
**LARP4**	0.162	18.587	0.723	0.514	-0.155	36.892	-0.146	27.828	-0.411	0.391
**LARS**	-0.768	0.052	0.635	0.944	-0.760	0.092	0.110	41.825	0.171	31.624
**LASP1**	0.214	18.587	-0.192	4.122	0.328	0.402	-0.269	0.341	0.484	0.119
**LAT2**	0.153	53.690	-0.238	1.663	0.319	0.092	-0.400	41.825	0.914	0.021
**LCAT**	-0.142	52.827	0.165	10.404	0.418	0.733	-0.128	8.987	0.276	24.398
**LCn2**									1.554	0.021
**LCP1**	-0.682	1.098	0.104	9.629	0.048	58.928	0.277	4.906	0.769	0.021
**LDB2**	-0.270	2.450	-0.160	5.829	0.389	0.724	-0.224	0.250	-0.182	33.879
**LDLR**	-0.051	53.690	-0.175	0.784	-0.448	0.092	-0.081	47.420	0.064	41.258
**LDLRAP1**	0.415	0.052	0.059	12.522	0.268	33.124	-0.368	27.828	0.603	0.021
**LEFTY1**			0.323	5.829	0.042	58.074			1.649	0.021
**LEPREL4**	0.016	57.109	0.229	0.595	-0.173	54.331	0.431	0.947	-0.110	24.398
**LGALS1**	-0.128	7.743	-0.325	3.205	0.171	36.892			0.794	0.021
**LGALS3**	-0.022	57.293	-0.227	4.122	-0.084	41.103	-0.076	32.497	0.950	0.021
**LGALS3BP**	0.016	56.765	-0.351	4.122	0.166	3.475	0.653	0.061	1.135	0.021
**LGALS4**	-0.686	0.398	1.055	0.107	0.271	0.724	0.427	11.241	0.387	0.746
**LGI1**	-0.239	0.398	0.528	2.773	-0.263	29.328	0.292	5.543	-0.673	0.471
**LGI4**	-0.110	44.812	0.134	4.122	-0.063	54.331	0.501	0.399	0.324	14.822
**LGR5**	0.933	0.262	-0.408	0.784	-0.540	4.733			-0.457	6.001
**LHX9**	-0.741	0.052	0.204	2.123	-0.898	0.092	-0.208	4.906	-1.206	0.021
**LIG3**	0.282	0.930	-0.654	0.514	0.059	54.331	0.114	41.825	0.161	35.531
**Lilrb3**	0.049	49.540	-0.717	0.514			0.053	50.493	1.255	0.021
**LIMA1**	-0.466	0.052	0.413	0.602	0.012	60.505	-0.317	32.497	0.854	0.021
**LIMD1**	0.142	25.771	0.178	2.614	0.079	36.892	0.451	0.061	0.311	2.886
**LIME1**	0.029	52.827	-0.499	0.205	0.050	55.771	0.128	32.497	0.188	11.001
**LIMK1**	-0.410	0.171	-0.489	3.009					0.090	33.879
**LIMS2**	0.110	49.540	-0.180	8.253	0.049	54.331	0.262	0.292	0.396	0.045
**LIN28B**			-0.393	0.784	-0.323	17.386	-0.541	32.497	-0.094	40.051
**LIN7A**	0.490	0.171	0.824	0.595	0.283	1.556	0.101	49.358	-0.618	0.269
**LINGO2**	-0.507	0.052	-0.362	0.204	0.266	41.103	0.202	2.415	-0.321	8.002
**LIPE**	0.103	44.812	-0.889	2.859	-0.128	22.899	0.394	0.341	0.320	24.398
**LITAF**	-0.338	0.093	0.221	0.514	0.045	57.395	-0.325	0.177	0.560	0.977
**LMAN2**	-0.063	44.812	0.467	0.514	-0.070	58.406	-0.125	23.450	-0.514	0.166
**LMCD1**	0.097	37.036	0.195	5.829	-0.071	56.823	0.735	0.061	-0.109	37.824
**LMNB1**	-0.228	0.262	0.286	5.829	-0.127	3.475	-0.853	0.121	-0.650	0.977

**LMNB2**	0.201	12.647	-0.401	4.122	0.661	0.092	-0.607	1.617	0.927	0.021
**LMO3**	0.163	12.647	0.506	0.514	-0.082	29.328	-0.097	32.497	-0.194	31.624
**LMO4**	-0.083	37.036	-0.144	5.829	-0.044	58.928	-0.281	41.825	-0.420	0.084
**LMTK2**	0.211	0.930	0.536	0.107	0.448	0.092	0.232	19.971	0.588	0.391
**LOC100505793/SRSF10**	-0.096	18.587	0.670	2.300	-0.140	58.074	-0.900	0.177	-0.262	4.218
**LOC390760**	-0.345	0.324	0.286	0.514	0.079	56.823	-0.559	1.210	-0.400	8.002
**LPAR4**			0.162	9.629	-0.484	0.092	0.131	7.099	-0.358	31.624
**LPAR6**	-0.564	0.052	-0.710	0.484	-0.295	10.694	-0.473	0.353	0.104	42.212
**LPCAT2**	-0.080	49.540	-0.110	5.829	-0.099	49.127	-0.220	23.450	0.552	0.045
**LPGAT1**	-0.213	0.171	0.047	10.404	-0.146	14.770	-0.078	49.358	-0.353	0.066
**LPHN1**	-0.193	0.511	-0.563	2.123	0.087	41.103	-0.080	27.828	-0.484	0.649
**LPHN2**	-0.102	52.827	0.190	3.205	0.416	0.856	-0.243	23.450	0.238	42.030
**LPIN2**	0.262	1.431	0.450	0.107	0.052	41.103	-0.452	3.866	-0.208	0.213
**LPL**	0.221	7.743	0.212	2.614	-0.166	4.733	-0.310	5.543	-0.518	0.021
**LPP**	0.516	5.928	0.664	0.107	-0.512	0.092	-1.402	0.061	-0.343	24.398
**LPXN**	-0.228	51.013	0.746	0.944	0.025	58.074	-0.308	50.493	1.386	0.021
**LRCH1**	-0.263	0.666	-0.140	10.404	-0.158	22.899	0.327	0.061	-0.437	0.021
**LRIG2**	0.560	0.093	0.183	5.829	-0.183	4.053	0.171	52.124	0.523	0.021
**LRIG3**	0.083	25.771	-0.279	9.629					-0.437	0.471
**LRP2**	0.110	15.278	-0.184	2.614	-0.969	0.856	0.345	16.936	-0.346	31.624
**LRP8**	-0.111	57.293	0.607	0.180	0.068	58.406	-0.779	11.241	0.278	33.879
**LRRC15**	0.044	49.540	-0.415	0.205	0.102	49.127	0.126	2.913	0.103	31.624
**LRRC28**	0.194	3.157	0.368	0.514	-0.278	1.556	-0.715	0.061	-0.583	0.084
**LRRC46**	-0.086	37.036	0.089	8.253	0.201	6.932	0.502	0.921	0.070	38.751
**LRRC7**	0.133	12.647	0.670	1.457	0.139	4.733	0.064	47.420	-0.396	0.471
**LRRK2**	-0.231	0.529	0.121	11.783	0.173	5.705	-0.517	0.061	-0.408	0.119
**LRRN3**	0.213	33.231	0.481	0.514	-0.158	4.733	0.291	1.210	-0.297	2.886
**LRRN4**	-0.244	49.540	0.527	2.123	-0.093	49.127	-0.908	0.061	-0.194	36.831
**LRRTM3**	-0.751	0.398	0.550	0.784	0.141	55.771	-0.948	2.415	-0.502	0.166
**LSS**	0.110	44.812	0.727	1.212	0.099	49.127	-0.425	11.241	-0.391	0.746
**Lst1**	-0.093	37.036	-0.392	3.205	0.327	0.724	-0.057	41.825	0.916	0.021
**LTBP3**	-0.106	12.647	-0.543	2.859	0.124	49.127	0.123	2.415	0.452	0.066
**LTBR**	0.036	49.540	0.484	0.595	-0.063	55.771	0.591	0.292	0.937	0.021
**LTC4S**	0.131	44.812	-0.095	8.881	0.196	0.856			0.627	0.021
**LTK**	0.082	52.827			0.286	1.819	-0.356	0.727	-0.215	14.822
**LTN1**	0.173	7.743	-0.421	0.514	0.053	54.331	0.114	19.971	-0.172	24.398
**LUC7L**	0.127	18.587	0.614	0.595	-0.161	14.770	-0.301	1.617	-0.257	31.624
**LY6E**	0.056	51.013			-0.124	49.127	0.043	51.354	-0.546	0.119
**LY86**	-0.570	0.170	-0.191	8.881	-0.063	58.074	0.132	5.543	1.361	0.021
**LY96**	-0.712	4.284	0.124	2.300	0.291	2.056	0.962	0.061	0.670	0.021
**LYL1**	0.089	37.036	-0.119	10.404	-0.103	58.928	0.438	2.913	0.744	0.021
**LYN**	0.353	0.052	0.665	0.669	0.233	0.659	0.112	7.099	0.511	0.993
**LYVE1**	-0.140	29.668	-0.832	1.663	0.310	20.025	-0.218	0.506	-0.523	0.307
**Lyz1/Lyz2**	0.106	49.540	-0.338	3.009	-0.312	0.281	0.245	0.177	1.476	0.021
**LZTS1**	-0.169	15.278	-0.751	0.602	0.210	33.124	-0.908	0.061	0.420	11.001
**MAD2L1**	0.288	0.511	0.352	2.859	0.157	17.386	0.285	7.099	-0.393	0.891
**MADD**	-0.212	1.098	-0.116	6.703	0.068	41.103	0.075	41.825	0.458	0.084
**MAEL**	-0.183	29.668			-0.531	2.056	-1.249	0.061	0.266	8.002
**MAF**	0.668	1.098	-0.439	2.859	0.266	9.255	-0.315	0.061	0.444	0.993
**MAFB**	0.115	49.540	-0.124	8.253	-0.071	58.928	0.127	32.497	0.621	0.021
**MAFF**	-0.448	18.587	0.588	0.204	0.510	0.092	-0.418	1.878	0.956	0.021
**MAFG**	0.277	0.171	0.397	0.445	0.279	4.733	-0.540	3.866	-0.056	35.531
**MAGI2**	0.034	52.827	-0.227	3.205	0.050	49.127	0.091	51.354	0.499	0.021
**MAGT1**	-0.058	44.812	0.411	2.123	0.075	55.771	0.404	16.936	0.800	0.084
**MAL2**	-0.049	55.870			0.507	0.517	-0.283	11.241	0.160	38.751
**MALAT1**	-0.787	0.170	0.451	5.829	-0.870	0.092	-1.325	0.061	0.510	0.021
**MAMDC2**	-0.112	44.812			-0.110	14.770			0.825	0.021
**MAML1**	0.466	0.936	0.171	5.829	-0.106	54.331	-0.058	54.403	0.213	2.886
**MAN2A2**	-0.311	2.450	0.360	2.300	0.316	3.068	-0.240	47.420	0.506	0.021
**MAN2B1**	-0.054	52.827	-0.234	2.300	-0.079	58.406	0.127	27.828	0.641	0.021
**MAN2B2**	-0.100	52.218			0.079	49.127	0.133	47.420	0.529	0.021
**MAOB**	-0.319	0.780	-0.564	0.893	-0.224	0.641	0.082	32.497	0.437	41.258
**MAP1B**	-0.102	44.812	-0.440	1.086	-0.407	0.240	0.045	53.829	0.115	31.624
**MAP2**	0.162	44.812	-0.604	4.122	-0.333	20.025	-1.073	0.061	-0.507	0.021
**MAP2K1**	0.013	56.130	0.493	1.663	0.052	55.771			-0.450	0.021
**MAP2K6**	-0.458	0.052	-0.441	1.457	0.051	57.395	0.398	47.420	0.041	42.670
**MAP3K1**	-0.430	0.171	0.250	5.829	-0.199	36.892	-0.256	0.353	0.611	0.066
**MAP3K14**	0.610	0.262	0.462	0.514	-0.073	41.103	-0.517	0.061	-0.187	31.624

**MAP3K2**	0.210	15.278	-0.265	4.122	-0.627	0.092	-0.269	41.825	0.329	24.398
**MAP3K9**	-0.350	4.284	-0.135	5.829	-0.142	9.255	0.125	50.493	0.843	0.021
**MAP4K3**	-0.274	0.666	0.536	1.457	-0.294	17.386	-0.341	0.727	-0.498	0.084
**MAP4K5**	-0.329	15.278	0.734	0.514	0.103	41.103	0.065	49.358	-0.495	0.891
**MAP7**	0.221	1.098	0.262	0.986	0.027	56.823	-0.759	0.061	0.587	0.307
**MAPK1IP1L**	-0.238	44.812	-0.186	1.772	-0.321	0.281	-0.859	0.061	-0.647	0.021
**MAPK6**	-0.132	25.771	-0.091	8.881	0.339	6.932	-0.204	0.855	-0.363	0.891
**MAPK7**	0.111	15.278	-0.443	0.784	-0.040	58.074	0.027	54.831	0.441	0.891
**MAPK8**	-0.349	0.126	-0.449	2.123	-0.239	36.892	-0.371	47.420	-0.514	0.045
**MAPKAPK2**	-0.347	25.771	0.273	0.484	0.159	33.124	-0.364	19.971	1.044	0.021
**MARCH6**	-0.148	49.540	0.618	0.107	-0.389	0.517	0.413	27.828	1.275	0.021
**MARK1**	0.074	44.812	0.098	11.783	0.090	49.127	0.096	41.825	-0.608	0.084
**MAS1**	0.255	0.170	0.196	7.537	0.153	36.892	-0.186	0.727	-0.481	0.993
**MASP2**	-0.600	0.052	-0.478	0.514	-0.245	55.771	0.389	3.866	0.158	24.398
**MAST4**	0.101	37.036	0.399	0.354	0.164	4.053	-0.091	8.987	-0.117	24.398
**MAVS**	-0.049	52.218	-0.274	3.205	0.107	20.025	0.016	54.831	0.406	0.418
**MAZ**	0.169	44.812	-0.542	2.859	0.189	0.856	-0.122	41.825	0.264	0.993
**MB**			-0.556	0.484			-0.232	41.825	-0.356	0.021
**MB21D2**	0.298	21.797	-0.454	0.514	0.118	49.127	0.158	41.825	0.671	0.119
**MBD1**	0.288	0.171	0.400	0.180	0.520	8.121	-0.721	0.506	-0.172	36.831
**MBNL1**	0.157	33.231	0.531	2.614	-0.107	33.124	-0.088	23.450	-0.382	0.021
**MBOAT1**	0.009	56.765	0.583	0.663	-0.043	58.928	-0.250	0.947	0.165	11.001
**MBP**	-0.121	15.278	0.341	0.784	0.249	14.770	-0.132	54.403	0.603	0.021
**MCL1**	0.355	0.126	0.563	1.457	-0.027	58.406	-0.077	32.497	0.245	1.977
**MCM2**	0.170	3.157	-0.507	2.859	-0.067	55.771	-0.149	49.358	0.495	0.021
**MCM6**	0.072	37.036	-0.462	1.289			0.471	0.506	0.182	8.002
**MCM8**	-0.041	52.218	-0.599	2.859	-0.052	58.611	0.203	13.893	-0.397	0.993
**MCTP1**	-0.423	0.324	0.241	2.614	-0.456	14.770	0.070	49.358	-1.262	0.021
**MDGA2**	0.186	44.812	0.571	1.289	-0.064	41.103	-0.440	0.947	-1.158	0.021
**MDH1**	-0.261	0.324	0.500	2.300	-0.164	17.386	-1.185	0.061	-0.406	0.021
**MDM4**	0.144	54.154	-0.471	0.893	0.324	22.899	-0.287	19.971	0.202	31.624
**ME1**	0.172	21.797	0.712	0.877	0.108	54.331	0.146	5.543	-0.441	0.021
**MED13**	0.648	0.171	0.396	1.772	0.116	33.124	-0.772	0.061	-0.622	0.119
**MED13L**	-0.239	0.666	0.536	2.614	-0.205	41.103	-0.141	50.493	-0.127	31.624
**MED22**	0.290	0.171	0.483	4.122	0.131	49.127	0.555	1.878	0.479	0.891
**MED7**	0.274	0.324	0.137	5.829	-0.206	6.932	0.514	0.121	1.092	0.021
**MEF2B**	-0.398	0.357	-0.131	5.829	0.132	6.932	0.174	16.936	-0.032	39.468
**MEF2C**	-0.339	15.278	-0.527	0.944	0.091	55.771			-0.055	38.751
**MEGF6**	0.213		0.481	0.514	0.727	0.092	0.186	47.420	0.357	40.660
**MEGF9**	-0.306	0.780	-0.454	2.859	-0.485	1.819	0.117	13.893	-0.299	2.886
**MELK**	-0.496	25.771	0.131	9.629			1.126	0.061	0.407	0.129
**MET**	-0.202	37.036	0.355	0.267	0.296	41.103	-0.608	0.399	0.347	31.624
**METTL20**	0.090	44.812	0.375	1.663	0.041	56.823			-0.674	0.021
**METTL5**	0.102	49.540	0.548	0.300	-0.090	33.124	0.139	7.099	-0.241	0.391
**MFAP3**	0.148	5.928	0.479	2.773	0.283	12.575	-0.506	0.061	-0.696	0.039
**MFI2**	-0.226	0.780	-0.298	0.484	0.035	55.771	0.339	27.828	0.718	0.021
**MFN1**	-0.073	51.013	0.889	0.420	0.057	49.127	-0.499	2.415	-0.179	24.398
**MFSD8**	0.072	44.812	0.759	0.595	0.103	20.025	0.490	5.543	-0.351	0.993
**MGAT4A**	0.655	0.171	0.619	0.204	0.022	57.395	0.147	51.354	-0.583	0.021
**MGEA5**	0.247	21.797	0.601	0.264	0.234	1.098	0.476	0.061	-0.123	14.822
**MGP**	-0.224	49.540	0.499	2.773	0.161	36.892	-0.264	41.825	0.054	24.398
**MGST1**	0.116	18.587	0.642	0.595	-0.173	20.025	0.238	23.450	0.674	0.021
**MGST3**	0.420	0.052	-0.531	2.859	-0.241	1.098	-0.127	41.825	-0.901	0.066
**MIA**	0.153	25.771	0.354	0.986	0.171	1.098	0.265	47.420	1.050	0.021
**MIB1**	-0.779	0.052	0.820	0.300	-0.438	5.705	0.515	19.971	-1.077	0.021
**MICAL1**	-0.086	29.668	-0.553	2.123	0.260	12.575	0.048	52.656	0.597	0.021
**MICALL1**	-0.059	49.540	-0.611	0.614	0.209	1.556	-0.154	41.825	0.601	0.993
**MICALL2**	-0.604	0.052	0.323	5.829	0.684	0.092	0.037	54.831	1.060	0.021
**MID1**	-0.118	5.928	-0.621	0.893	-0.178	8.121	0.043	52.656	0.251	0.886
**MIER1**	-0.312	0.093	-0.156	11.093	-0.413	0.092	1.176	0.399	-0.099	24.398
**MITD1**	0.517	15.278	0.578	0.180	0.117	4.733	0.234	47.420	-1.278	0.021
**MITF**	-1.152	0.052	0.255	0.354	-0.530	10.694	-1.540	0.061	0.478	31.624
**MKL2**	0.518	1.098	-0.391	2.614	0.227	2.056	0.326	8.987	-0.699	0.021
**MLC1**	-0.191	0.511	-0.179	0.514	0.057	54.331	0.086	27.828	0.518	0.391
**MLLT4**	0.312	29.668	0.710	1.086	0.132	41.103	0.194	19.971	-0.579	0.021
**MLXIP**	0.290	4.284	0.251	7.537	0.037	58.406	-0.605	0.506	-0.386	0.066
**MLXIPL**	-0.413	29.668	-0.200	4.122	0.196	41.103	0.430	7.099	0.884	0.021
**MMD**	-0.096	54.701	0.491	0.180	0.045	56.823	0.218	0.353	-0.206	11.001

**MMP15**	0.414	0.324	0.274	2.300					-0.184	35.531
**MMP2**	0.223	0.529	0.271	2.773	0.053	54.331	-0.462	1.210	0.788	0.021
**MMP9**	-0.217	9.997	0.255	5.829	-0.112	56.823	1.018	0.061	-1.073	0.021
**MOBP**	-0.312	44.812	-0.541	0.669	-0.352	0.856	-0.698	41.825	0.442	31.624
**MOGAT1**	-0.096	37.036	-1.311	0.354	0.046	54.331			-0.424	0.269
**MORC3**	0.132	37.036	0.750	1.457	0.020	58.928	-0.103	47.420	-0.481	0.021
**MPDZ**			-0.574	2.773	-0.109	12.575	-0.877	2.913	1.183	0.021
**MPEG1**	-0.485	0.052	-0.230	0.514	-0.282	41.103	0.285	11.241	0.752	0.021
**MPHOSPH9**	-0.153	12.647	0.727	0.107	-0.049	56.823	-0.474	0.130	-0.754	0.021
**MPP1**	-0.559	0.093	0.177	1.534	-0.352	4.733	-0.154	52.656	-0.403	0.045
**MPP7**	-0.283	25.771	-0.789	5.829	-0.034	59.432	-0.221	49.358	-0.871	0.084
**MR1**	-0.584	4.284	-0.273	1.086	-0.343	17.386	-0.111	41.825	0.826	0.021
**MRC2**	-0.216	49.540	-0.115	5.829			0.320	16.936	0.705	0.021
**MRGPRF**	-0.124	29.668	-0.533	2.300	0.212	4.053	0.079	50.493	0.992	0.021
**MRI1**	0.183	1.431	0.099	0.784	0.025	60.505	0.409	0.061	0.235	0.649
**MRPL15**	0.200	0.529	0.581	1.086	-0.257	6.932	0.538	0.292	-0.837	0.021
**MRPS2**	-0.162	7.743	-0.474	2.773	0.017	58.611	-0.196	5.543	-0.471	0.084
**MSH3**	-0.471	0.511	0.624	0.595	-0.187	20.025	0.694	16.936	-0.760	0.021
**MSI2**	-0.293	1.936	0.366	1.457	-0.429	0.092	-0.513	19.971	0.338	0.649
**MSN**	-0.286	0.666	0.377	1.663	0.231	1.309	0.131	41.825	0.864	0.021
**MT1E**					0.117	49.127			0.772	0.269
**MT1F**	0.269	0.171	0.259	5.829	0.263	0.402	-0.039	54.403	0.657	0.084
**MTA1**	0.428	0.324	0.323	0.595	0.082	49.127			0.090	37.824
**MTA2**	0.146	29.668	-0.390	0.428	-0.039	58.611	-0.109	41.825	0.170	24.398
**MTDH**	-0.158	49.540	0.512	0.595	-0.227	2.659	0.470	0.947	-0.075	31.624
**MTHFSD**	0.041	49.540	-0.587	2.773	0.025	58.074			-0.366	0.166
**MTM1**	-0.425	0.780	-0.309	0.784	0.288	33.124	-1.283	0.121	-0.478	24.398
**MTMR10**	-0.374	0.398	0.305	1.457	0.156	36.892	-0.531	16.936	0.332	35.531
**MTMR11**	0.032	52.827	0.409	0.514	-0.163	36.892	0.250	27.828	0.630	0.021
**MTMR12**	0.042	49.540	0.210	5.829	-0.064	55.771	-0.276	0.573	-0.623	0.021
**MTPN**	-0.145	29.668	-0.603	0.514	0.211	4.053	0.137	47.420	-0.388	0.119
**MTRR**	-0.342	3.157	-0.459	2.300	0.042	58.074	-0.687	13.893	0.836	0.119
**MTSS1**	-0.432	2.450	0.556	0.174	-0.452	0.092	-0.206	41.825	-0.826	0.269
**MTSS1L**	-0.307	1.431			0.159	14.770	-0.497	4.906	0.442	0.021
**MTUS1**	0.514	1.431	0.477	0.445	0.050	49.127	0.295	3.866	0.589	0.119
**MUC1**	-0.041	51.013	-0.465	0.009	0.016	59.432	0.058	41.825	0.021	44.052
**MUTYH**	0.233	1.098	-0.721	0.784	0.268	4.053	0.244	27.828	0.429	0.021
**MX1**	-0.383	0.529	0.143	11.093	0.181	54.331	-0.077	47.420	1.012	0.021
**MXD3**	-0.236	2.450	-0.167	4.122	-0.369	0.517	-0.615	0.506	-0.492	0.891
**MXRA8**	-0.135	52.827	0.373	0.264			1.017	0.061	0.323	11.001
**MYB**	0.060	44.812	-0.420	0.534	-0.310	49.127	-0.518	0.332	0.159	24.398
**MYBPC3**	0.278	0.324	0.521	0.595	0.052	55.771	-0.431	0.061	-0.084	31.624
**MYCN**	-0.138	51.013	0.880	1.534	0.208	9.255			-0.475	0.021
**MYF6**	-0.084	37.036	-0.213	5.829	0.059	41.103	0.451	0.915	0.008	44.315
**MYH2**	-0.404	0.052	-0.163	4.122	0.343	49.127	0.037	49.358	0.067	37.824
**MYH3**	0.351	0.052	0.806	0.264	0.294	0.724	-0.083	54.403	-0.276	33.879
**MYH6**	0.407	0.052	0.394	2.773	0.602	0.092	0.122	32.497	0.083	44.203
**MYL12B**	0.042	52.218	0.395	0.514	-0.036	58.611	0.032	51.354	0.441	0.307
**MYL6B**	-0.422	0.126	-0.313	5.829	-0.076	54.331	-0.168	41.825	-0.243	11.001
**Myl9**			0.258	2.300	0.271	4.733	0.143	2.415	0.582	0.021
**MYO1B**	-0.318	0.930	0.876	0.986	-0.371	0.440	-0.456	0.399	-1.217	0.021
**MYO1F**			-0.155	0.784	0.583	0.440			1.046	0.021
**MYO3B**	0.422	0.170	-0.266	5.829	-0.092	54.331	-0.565	11.241	-0.051	37.824
**MYO5A**	0.129	29.668	0.473	1.663	0.245	0.724	0.191	11.241	-0.723	0.021
**MYO6**	0.230	4.284	0.211	0.944	-0.222	6.932	0.481	0.399	0.279	0.471
**MYO9A**	0.329	4.284	0.499	0.893	0.036	54.331	0.146	47.420	-0.290	6.001
**MYOD1**	-0.202	0.666	-0.498	0.784	-0.114	41.103	-0.089	51.354	0.007	44.315
**MYOM1**	-0.635	0.052	0.169	0.514	0.441	0.641	1.133	0.250	-0.302	6.001
**MYOM2**	0.287		0.396	0.669	-0.325	29.328	0.621	0.987	-0.057	58.421
**MYSM1**	-0.834	0.052	0.404	0.944	-0.206	14.770	0.832	0.292	-0.905	0.021
**NAA30**	0.528	0.052	0.744	0.569	0.235	0.240	0.032	55.172	-0.215	0.891
**NAB2**	0.329	0.398	-0.342	7.537	0.091	54.331	-0.062	52.124	0.520	0.119
**NANS**	-0.393	25.771	-0.666	3.205	0.128	14.770	0.483	0.061	0.153	38.751
**NAP1L3**	-0.086	33.231	0.436	5.829	-0.058	55.771	-0.225	0.947	-0.524	0.021
**NAPA**	-0.058	51.013	-0.417	0.204	0.073	49.127	0.425	7.099	0.607	0.021
**NAPG**	0.168	18.587	0.477	1.029	0.172	12.575			-0.734	0.045
**NAPRT1**	-0.110	44.812	-0.329	2.859	0.110	17.386	-0.091	49.358	0.577	0.213
**NAT**	0.409	1.431	-0.254	2.123	-0.392	29.328	-1.011	0.061	-1.094	0.021

**Nav2**	-0.061	51.013	0.563	1.289	0.161	41.103	-0.153	27.828	-0.434	0.993
**NAV3**	-0.268	0.398	-0.373	0.514	-0.531	0.092	-1.094	0.727	-0.466	0.084
**NBEAL1**	-0.103	51.013	-0.804	0.354	-0.347	6.932	0.134	8.987	0.275	31.624
**NCALD**	-0.273	15.278	-0.491	3.009	-0.358	10.694	-0.094	54.403	-0.622	0.993
**NCAM1**	-0.199	5.928	0.770	1.086	-0.232	12.575	-0.432	0.353	-0.390	0.045
**NCAPH2**	0.204	5.928	0.409	0.669	-0.044	54.331	0.198	0.506	-0.501	0.993
**NCF2**	-0.191	5.928	0.391	0.877	0.082	49.127	0.231	0.855	1.228	0.021
**NCF4**	0.194	25.771	-0.118	5.829	0.149	6.932	0.155	1.617	1.235	0.021
**NCK1**	-0.238	37.036	0.507	0.514	0.054	58.406	0.089	32.497	-1.172	0.021
**NCKAP1L**	-0.216	44.812	-0.236	2.773	0.078	54.331	-0.150	41.825	0.911	0.021
**Ncl**	-0.183	1.936	0.587	1.457	-0.057	57.395	0.237	5.543	0.435	0.021
**NCOA3**	0.119	37.036	-0.455	3.009	0.212	41.103	0.476	0.061	0.060	39.468
**NCOR1**	-0.149	4.284	0.729	1.534	-0.261	2.659	0.236	19.971	-0.653	0.021
**NDOR1**	0.288	0.529	0.451	0.514	0.050	57.395	-0.221	16.936	-0.669	0.021
**NDRG2**			0.558	0.180			0.063	47.420	0.141	38.751
**NDRG3**	0.176	5.928	0.338	2.300	-0.183	22.899	-0.050	49.358	-0.563	0.119
**NDST1**	0.113	44.812	0.675	0.986	-0.240	49.127	0.270	4.906	-0.206	24.398
**NDST3**	-0.381	0.052	-0.053	11.093	-0.235	1.309	0.137	51.354	-0.025	40.660
**NDUFAB1**	-0.029	57.109	0.480	2.300	-0.109	36.892	-0.482	1.332	-0.400	0.993
**NDUFAF4**	0.063	44.812	0.482	0.669	0.034	57.395	0.237	0.573	0.044	43.745
**NECAB2**	-0.433	0.052	-0.080	10.404	-0.356	29.328	0.302	0.506	-0.278	31.624
**NEDD4L**	0.373	0.052	0.627	0.669	-0.152	36.892	0.205	7.099	-1.209	0.021
**NEDD9**	0.390	0.170	0.690	0.569	0.209	3.475	0.486	5.543	0.710	0.166
**NEFL**	0.054	51.013	0.135	13.299	-0.161	26.120	-0.085	19.971	-0.430	0.021
**NEFM**	0.263	33.231	0.626	11.780	-0.049	58.406	-0.021	47.420	-0.676	0.021
**NEK1**	-0.583	0.052	0.665	0.514	-0.900	0.092	0.341	23.450	-0.355	0.066
**NEK4**	-0.173	1.098	-0.731	0.107	-0.277	1.556	0.121	32.497	-0.087	33.879
**NES**	-0.178	0.324	-0.671	2.773	0.448	0.402	-0.154	32.497	0.328	11.001
**NETO1**	-0.368	0.052	-0.249	2.773	-0.421	3.475	-0.354	1.617	-0.233	1.193
**NETO2**	0.245	0.171	0.301	6.703	0.063	41.103	0.441	0.061	0.057	38.751
**NEU2**	-0.225	21.797	-0.297	4.122	0.345	9.255	-0.409	0.061	-0.072	31.624
**NEUROD6**	0.241	0.398	0.151	11.783	-0.162	26.120	0.045	47.420	-0.381	0.391
**NFASC**	0.164	44.812	-0.237	2.614	0.017	59.793	-0.455	0.061	0.057	42.554
**NFATC2**	0.154	37.036	-0.279	3.205	-0.487	49.127	0.111	23.450	-0.578	0.021
**NFATC3**	-0.508	0.324	0.467	0.107	-0.473	0.092	-0.332	0.109	-0.723	0.021
**NFE2L2**	-0.270	9.997	0.592	1.457	0.130	26.120	0.257	32.497	0.574	0.129
**NFIA**	-0.181	12.647	0.079	8.881	-0.180	2.311	0.060	52.124	0.444	0.746
**NFIC**	0.052	53.690	-0.178	4.122	0.171	49.127	0.120	11.241	0.474	0.021
**NFIL3**	0.195	37.036	0.390	1.086	0.517	0.641	-0.177	27.828	0.486	0.021
**NFKB2**	0.268	15.278	0.585	0.514	0.104	49.127	-0.423	19.971	-0.542	0.993
**NFS1**	0.712	0.052	0.581	0.514	0.266	49.127	0.333	0.947	-0.261	14.822
**NFU1**	0.265	1.936	0.620	0.784	-0.061	55.771	-0.127	16.936	-0.275	1.977
**NHLH1**	0.095	52.218	0.309	0.514	0.462	0.724	0.185	47.420	0.901	0.021
**NIPA1**	0.102	44.812	-0.437	2.859	-0.147	9.255	-0.175	16.936	-0.754	0.021
**NKAIN2**	-0.397	3.157	0.222	4.122	0.459	0.092	-0.716	5.543	-0.260	2.886
**NKX6-2**	0.017	54.154	0.188	6.703	-0.230	0.281			0.539	0.993
**NLRP10**	0.057	44.812	0.298	5.829	0.326	2.659	-0.828	0.061	-0.281	14.822
**NME7**	0.035	51.013	0.600	1.457	-0.072	54.331	0.296	8.987	-0.436	0.021
**NMI**	0.199	33.231	0.306	5.829	0.360	14.770	-0.133	53.829	-0.683	0.021
**NNAT**	-0.281	4.284	0.161	8.881	0.071	55.771	0.043	51.354	0.400	0.021
**NOL6**	-0.230	9.997	0.409	5.829	-0.218	3.475	-0.488	41.825	-0.532	0.021
**NOP56**	-0.201	4.284	0.303	5.829	-0.162	14.770	0.148	32.497	0.356	0.021
**NOTCH3**	0.085	44.812	-0.237	4.122	0.111	22.899	0.261	3.866	0.540	0.021
**NOTCH4**	0.012		0.472	1.663	0.481	0.092	0.784	0.855	0.480	0.062
**NOV**	-0.265	1.098	0.999	0.569	0.549	0.092	0.176	2.415	0.263	41.258
**NOXO1**	0.193	29.668			-0.124	41.103	-0.593	0.866	0.331	11.001
**NPAS1**	0.084	49.540	0.498	0.230	0.359	0.693	-0.025	52.656	0.307	11.001
**NPAS4**	0.092	49.540	0.403	0.986	0.344	14.770	0.076	50.493	-0.486	0.269
**NPB**	0.246	9.997	-0.647	0.514	0.240	0.440	0.186	2.913	0.835	0.066
**NPC2**	0.147	1.431	0.360	0.514			0.160	47.420	0.454	0.045
**NPHP3**	-0.479	0.262	0.596	1.457	0.297	33.124	0.714	0.399	0.693	0.066
**NPPC**	0.128	15.278			-0.220	10.694	-0.173	1.617	-0.598	0.746
**NPR2**	0.332	1.431	-0.318	4.122	0.147	20.025	-0.443	0.341	0.693	0.021
**NPRL3**	0.136	18.587	0.192	0.514	0.048	54.331	0.298	0.109	-0.444	0.471
**NPY2R**			1.000	0.354			-0.431	3.866	-0.299	11.001
**NPY5R**	0.098	44.812	0.685	2.614	0.090	36.892	0.258	13.893	-0.547	0.993
**NR2C1**	-0.091	52.218	0.496	2.300	0.481	0.402	-0.824	0.121	0.200	8.002
**NR2E1**	0.023	55.170	0.628	0.602	-0.050	59.793	0.081	52.656	0.512	0.021

**NR2F2**	-0.360	0.052	-0.338	3.009	0.090	41.103	0.114	32.497	0.333	14.822
**NR4A1**	0.587	0.171	0.420	3.205	0.051	55.771	-0.249	7.099	0.124	44.052
**NR4A2**	-0.061	55.559	-0.386	5.829	-0.071	57.395	-0.100	23.450	-0.460	0.391
**NRARP**	0.039	49.540	-0.437	0.445	-0.226	2.659	0.763	0.292	-0.644	0.891
**NRAS**	0.116	37.036	0.268	0.514	0.121	8.121	-0.365	0.130	-0.302	0.213
**NRCAM**	-0.732	0.171	0.845	0.595	-0.099	29.328	0.044	47.420	1.065	0.084
**NRG1**	-0.459	0.052	-0.250	3.205	0.582	0.517	0.104	41.825	0.346	8.002
**NRIP1**	-0.182	37.036	-0.543	0.784	-0.355	0.402	-0.291	23.450	0.642	24.398
**NRP1**	-0.482	0.171	0.499	2.300	-0.480	0.092	0.501	0.250	-0.502	0.021
**NRXN1**	-0.311	0.052	-0.504	0.107	0.596	2.659	0.272	41.825	-0.421	0.746
**NRXN3**	0.706	0.052	0.638	0.877	-0.295	0.402	-0.563	27.828	-0.628	0.471
**NSBP1**	0.195	49.540	0.637	1.457	-0.080	41.103	-0.124	50.493	-0.473	0.021
**NSDHL**	0.262	0.324	0.360	4.122	-0.098	41.103	0.190	47.420	-0.791	0.021
**NSUN6**	0.117	7.743	0.550	0.514	-0.057	54.331	-0.179	49.358	-0.437	0.119
**NT5C2**	-0.190	0.398	-0.531	2.300	0.178	2.056	-0.147	13.893	-0.201	8.002
**NTF3**	-0.391	1.098	-0.202	2.859	-0.452	33.124	0.313	32.497	-0.476	24.398
**NTM**	0.205	0.262	0.453	0.180	0.256	2.056	0.110	41.825	-0.088	33.879
**NTNG1**	-0.585	0.052	-0.627	0.784	-0.235	6.932	0.277	0.855	-0.375	0.471
**NTRK2**	-0.092	44.812	0.425	4.122	0.182	8.121	0.078	47.420	0.609	0.021
**NTRK3**	0.077	44.812	0.088	5.829	0.080	36.892	0.031	50.493	-0.408	0.039
**NUAK1**	-0.550	0.052	-0.119	6.703	-0.652	2.659	-0.274	0.061	0.009	44.052
**NUAK2**	-0.550	0.052	-0.119	6.703	-0.652	2.659	-0.274	0.061	0.212	31.624
**NUCKS1**	-0.070	49.540	0.436	0.569	-0.070	55.771	-0.040	51.354	-0.454	0.045
**NUDCD1**	0.466	0.171	0.354	5.829	0.047	54.331	0.270	27.828	-0.249	14.822
**NUFIP2**	-0.141	49.540	-0.617	0.514	-0.278	36.892	-0.273	0.573	-0.216	0.649
**NUMB**	0.191	21.797	0.481	0.514	0.295	2.659	-0.108	41.825	-0.820	0.021
**NUP155**	-0.469	0.052	-0.763	0.107	-0.152	10.694	-0.338	5.543	-0.606	0.213
**NUP160**	0.235	25.771	0.566	1.772	-0.329	0.659	0.100	54.831	-0.459	0.021
**NUP205**	-0.899	0.052	-0.522	4.122	0.449	0.856	0.200	1.617	0.132	33.879
**NUPR1**	0.184	49.540	0.636	0.300	0.406	3.068	-0.270	41.825	0.777	0.021
**NVL**	-0.529	0.052	0.369	5.829	-0.615	0.240	-0.339	32.497	1.125	0.021
**NXN**	0.131	37.036	0.178	1.663	0.326	0.440	-0.538	0.130	0.458	0.471
**NXNL2**	0.221	21.797	0.668	0.420	0.320	14.770			0.385	33.879
**OAS1**	0.475	0.052	0.086	12.522	0.609	0.092	0.680	2.415	0.716	0.021
**Oas2**			-0.086	11.093					1.140	0.021
**OBFC2A**	-0.138	4.284	0.223	2.300	-0.162	10.694	0.141	19.971	-0.422	0.119
**OCRL**	-0.136	55.170	0.475	0.107	-0.189	29.328	0.319	32.497	-0.765	0.119
**ODF2L**	0.156	9.997	0.754	0.784	-0.044	60.231	0.176	47.420	-0.221	24.398
**ODZ3**	0.430	15.278	-0.640	0.514	-0.375	2.659	-0.180	49.358	0.479	0.021
**OGG1**	0.207	25.771	-0.401	6.703	-0.091	54.331	0.399	8.987	0.554	0.021
**OGN**	-0.411	29.668	0.320	9.629	0.765	0.092	0.649	2.415	-0.406	24.398
**OLFML2B**	0.072	44.812	0.196	12.522	-0.162	56.823	-0.084	49.358	-0.523	0.391
**OLFML3**	0.073	37.036	-0.114	4.122	0.131	36.892	0.123	41.825	0.598	0.021
**OPLAH**	-0.558	0.052	0.230	8.881	0.200	9.255	0.605	1.617	0.426	31.624
**OPN4**			0.086	5.829					-0.474	0.891
**OPRM1**	0.023	52.827	-0.567	0.514	-0.109	54.331	-0.101	49.358	-0.226	8.002
**ORC4**	0.289	3.157	0.512	0.484	0.036	54.331	0.066	41.825	-0.359	0.391
**OSBPL11**	0.214	0.780	0.869	0.986	0.109	20.025	-0.054	47.420	-0.083	31.624
**OSBPL3**	0.350	4.284	0.311	5.829	0.272	4.053	-0.199	0.727	-0.954	0.021
**OSMR**	0.193	15.278			0.311	0.724	0.408	5.543	1.362	0.021
**OTOF**	-0.327	33.231	0.261	1.663	0.508	0.453	0.072	47.420	0.042	43.175
**OTOR**	-0.330	0.171	0.082	10.404	0.079	26.120	-0.115	41.825	-0.442	0.977
**OTOS**	-0.063	49.540	-0.244	2.614	-0.380	0.821	-0.100	13.893	0.106	43.008
**OTUB1**	-0.286	51.013	0.223	8.881	0.066	49.127	-0.429	0.061	-0.450	0.119
**OXR1**	0.187	33.231	0.471	0.107	-0.145	14.770	-0.255	11.241	-0.401	0.119
**P2RX7**	0.247	5.928			0.155	33.124	0.155	41.825	0.455	0.193
**P2RY1**	0.128	25.771	0.277	5.829	0.443	2.056	0.559	5.543	0.461	0.391
**P2RY13**	-0.139	25.771	0.212	9.629	0.064	33.124	0.442	2.415	0.662	0.021
**P2RY2**	-0.613	0.052	-0.710	0.484	-0.295	10.694	-0.473	0.353	0.104	42.212
**P2RY6**	0.307	0.052	0.248	0.784	-0.530	0.440	-0.168	53.829	0.406	0.993
**PACSIN3**	0.662	0.170	-0.769	1.772	0.163	10.694	0.036	54.831	0.448	0.391
**PADI2**	-0.245	4.284			0.172	3.068	0.196	0.855	0.839	0.021
**PADI4**	-0.406	3.157	-0.190	4.122	-0.171	56.823	-0.437	27.828	0.583	0.418
**PAG1**	0.027	56.874	-1.437	0.107	-0.289	1.098	-0.609	19.971	-0.519	24.398
**PAK2**	0.054	49.540	0.233	5.829	-0.069	41.103	-0.472	0.061	-0.429	0.021
**PAK3**	0.100	37.036	-0.710	0.204	-0.320	0.281	-0.134	47.420	-0.222	0.649
**PAK4**	0.052	44.812			0.095	36.892	-0.124	49.358	0.536	0.021
**PAM**	-0.305	0.170	-0.545	0.669	-0.239	12.575	-0.079	52.124	-0.476	0.066

**PANK1**	-0.068	49.540	0.402	2.300	0.230	2.311	-0.261	2.415	-0.622	0.391
**PANK2**	0.066	44.812	0.539	0.944	0.026	56.823	0.070	47.420	-0.387	0.021
**PANK3**	-0.139	51.013	0.700	3.205	-0.134	33.124	-0.158	41.825	-0.727	0.021
**PAOX**	0.049	44.812	-0.492	0.784	-0.106	36.892	0.244	8.987	0.485	0.269
**PARD3**	-0.096	44.812	0.570	0.602	0.229	0.856	-0.635	4.906	-0.166	24.398
**PARD6G**	0.275	15.278	0.558	1.457	0.236	1.819	0.032	52.656	-0.439	0.084
**PARP14**	0.236	18.587	0.228	8.253			0.436	5.543	1.411	0.021
**PARP3**	0.049	49.540	0.207	4.122	0.577	0.092	0.084	51.354	0.284	33.879
**PARP9**	-0.381	37.036			-0.142	5.705	0.334	32.497	0.492	0.021
**PARVG**	0.239	0.171	-0.162	4.122	0.357	10.694	-0.159	47.420	0.557	0.891
**PAX6**	-0.431	25.771	0.331	1.457	0.665	3.475	0.470	1.210	0.993	0.045
**PBX1**	-0.477	0.171	0.818	0.595	-0.411	2.659	-0.734	0.061	-0.463	0.649
**PBX3**	-0.130	49.540	0.383	1.086	0.312	4.053	0.171	3.866	-0.663	0.993
**PBXIP1**	-0.157	44.812	-0.345	0.784	0.159	29.328	-0.117	7.099	0.527	0.084
**PCBP1**	0.040	52.218	-0.581	2.773	0.057	56.823	-0.082	32.497	0.529	0.471
**PCBP2**	0.196	1.098	0.403	0.514	0.050	54.331	0.217	19.971	0.433	0.021
**PCDH1**	-0.061	51.013	-0.405	0.986			-0.053	50.493	0.007	44.315
**PCDH17**	-0.768	0.666	0.301	1.663	-0.750	0.092	-0.605	1.210	0.198	40.660
**PCDH9**	-0.608	0.052	0.545	5.829	-1.195	0.724	0.128	47.420	0.497	24.398
**Pcdhb12**	0.029	57.109	-0.866	2.859	-0.089	54.331	0.542	0.061	0.165	11.001
**PCGF5**	0.077	49.540	0.669	0.514	0.132	49.127	0.086	16.936	0.496	0.119
**PCM1**	-0.547	0.170	0.495	3.205	-0.466	0.402	-0.218	0.399	0.497	0.021
**PCP4**	-1.036	0.052	-0.212	0.514	-0.663	5.705	0.467	0.250	-0.318	33.879
**PCSK1**	0.730	52.218	0.907	2.614	0.178	3.475	0.696	32.497	0.771	0.977
**PCYT1B**	0.411	29.668	-0.460	4.122	-0.135	36.892	0.185	27.828	-0.509	0.045
**PDCD1**									1.200	0.021
**PDCD11**	0.467	0.052	0.460	2.773	0.569	0.092	0.629	32.497	0.529	1.193
**PDE10A**	-0.282	15.278	-0.560	1.086	0.229	41.103	-0.366	0.727	0.488	24.398
**PDE12**	0.396	0.170	0.589	0.514			-0.134	47.420	0.073	43.867
**PDE1A**	-0.124	37.036	0.462	4.122	-0.145	33.124	-0.110	13.893	-0.434	0.021
**PDE2A**	-0.089	49.540	-0.428	0.595	-0.231	0.517	-0.078	49.358	-0.067	36.831
**PDE4A**	0.090	49.540	0.354	0.514	-0.217	0.659	0.136	51.354	-0.502	0.119
**PDE4DIP**	-0.865	0.511	0.767	0.877	-0.203	20.025	-0.491	47.420	0.246	0.391
**PDE6G**	-0.080	21.797	-0.581	2.773			-0.061	41.825	0.472	0.891
**PDE6H**	0.081	55.559	-0.357	4.122	-0.687	0.092	-0.389	2.415	-0.061	31.624
**PDEFD**			-0.476	0.784					0.691	0.021
**PDGFC**	-0.702	0.052	0.698	1.029	0.314	4.053	0.710	0.250	-0.873	0.993
**PDGFRA**	0.087	44.812	0.635	0.595	0.092	36.892	-0.075	52.656	0.263	33.879
**PDHA1**	0.028	55.170	0.444	0.514	-0.258	0.641	-0.062	47.420	-0.518	0.021
**PDIA5**	0.149	2.450	0.397	0.180	0.049	54.331	-0.089	47.420	-0.234	31.624
**PDlim1**	-0.248	0.171	0.203	1.772	0.247	20.025	0.117	47.420	0.420	0.021
**PDLIM2**	0.156	0.930	0.291	0.986	-0.063	54.331	-0.079	52.656	0.621	0.891
**PDLIM4**	-0.108	25.771	-0.383	3.205	0.193	41.103	0.404	0.130	1.217	0.021
**PDP1**	0.137	37.036	0.360	2.300	0.279	0.281	0.289	5.543	-0.540	0.649
**PDSS1**	0.251	7.743	0.356	7.537			0.342	1.210	-0.856	0.021
**PDXK**	-0.426	3.157	-0.951	0.107	-0.677	0.092	0.293	41.825	0.450	0.066
**PDYN**	-0.181	44.812	-0.223	7.537	0.367	5.705	0.107	41.825	0.503	0.021
**PDZD2**	-0.953	0.052	0.344	1.772	0.898	0.092	-0.639	5.543	-0.217	6.001
**PDZD3**	0.175	1.431	0.232	2.123	0.209	41.103	-0.311	19.971	-0.922	0.021
**PEBP1**	0.995	0.052	0.181	8.253	-0.055	54.331	-0.429	41.825	0.232	37.824
**PELI1**	0.367	0.529	-0.366	2.773	-0.468	0.402	-1.048	0.061	-0.442	0.119
**PELI2**	0.434	0.052	0.853	0.569	-0.311	9.255	0.314	19.971	0.699	0.021
**PELP1**	0.094	49.540	0.490	0.107	0.798	0.092	-0.873	0.727	0.201	1.193
**PEMT**	0.271	44.812			0.214	14.770	-0.927	0.061	0.547	0.021
**PENK**	-0.402	0.262	0.608	0.180	0.549	0.517	-0.197	51.354	0.674	0.649
**PER1**	0.326	0.324	0.188	7.537	0.173	1.309	-0.418	0.061	-0.266	11.001
**PER2**	0.185	3.157	0.812	0.569	0.010	60.505	-0.098	11.241	-0.223	31.624
**PERP**	-0.142	5.928	0.468	1.086	-0.056	56.823	0.534	0.130	0.249	11.001
**PES1**	-0.173	25.771			-0.104	58.074	0.469	32.497	1.060	0.021
**PEX1**	-0.795	0.171	0.830	0.877	0.149	49.127	0.653	0.061	-0.606	14.822
**PEX13**	0.307	12.647	0.670	0.595	0.141	33.124	-0.105	52.656	-0.563	0.084
**PEX14**	0.214	21.797	-0.512	2.859	0.088	49.127	0.061	47.420	0.627	0.269
**PEX5L**	0.050	49.540	-0.412	0.944	0.073	49.127	0.077	32.497	0.138	38.751
**PFDN2**	-0.075	53.690	0.544	0.264	0.051	54.331	0.075	47.420	0.081	36.831
**PFKFB3**	0.016	56.874	-0.475	0.784	0.313	0.092	0.032	50.493	0.260	0.269
**PFKFB4**	-0.094	44.812	-0.144	2.614	0.026	57.395	-0.095	50.493	0.496	0.021
**PGCP**	-0.484	9.997	0.562	0.514	-0.140	41.103	0.139	41.825	0.489	0.891
**PGD**	0.106	44.812	0.320	0.595	-0.067	49.127	0.460	0.061	-0.206	24.398

**PGDS**									0.785	0.045
**PGM2L1**	-0.198	1.431	0.435	5.829	0.443	0.092	-0.688	19.971	-1.277	0.021
**PGM3**	0.068	37.036	-0.182	0.514	0.046	49.127	-0.426	0.061	0.451	0.213
**PGM5**	0.032	51.013	-0.266	3.205	0.325	1.819	0.250	19.971	0.541	0.066
**PGM5**	-0.324	1.936	-0.266	3.205	0.325	1.819	0.250	19.971	0.541	0.066
**PGRMC2**	0.271	0.171	0.502	0.107	0.048	54.331	0.183	7.099	-0.196	1.193
**PHACTR1**	0.180	37.036	0.401	0.204	-0.212	49.127	0.754	0.061	-0.509	0.993
**PHC3**	-0.132	33.231	0.359	5.829	0.045	49.127	-0.077	52.124	-0.464	0.021
**PHF14**	0.333	15.278	0.578	0.784	0.069	41.103	0.058	53.272	-0.417	0.084
**PHF20L1**	0.280	25.771	0.541	0.514	-0.145	54.331	-0.123	32.497	-0.632	0.021
**PHF21A**	-0.050	56.765	0.533	5.829	-0.188	54.331	-0.109	52.656	-0.409	0.045
**PHKA1**	0.178	33.231	0.081	10.404			0.919	0.573	0.509	0.993
**PHKA2**	-0.207	0.666	-0.549	0.300	-0.129	49.127	-0.394	41.825	0.619	0.471
**PHTF2**	0.013	56.765	-0.212	4.122	0.062	54.331	0.117	32.497	0.642	0.471
**PHYHD1**			-0.243	2.300	0.032	58.406	0.125	47.420	0.835	0.021
**PIAS1**	0.159	18.587	0.542	1.289	0.115	54.331	-0.317	0.855	-0.437	0.307
**PIGG**	-0.309	0.262	-0.648	0.784	0.194	14.770	-0.276	47.420	0.459	0.269
**PIGh**	-0.178	1.936	0.259	8.881	-0.272	20.025	-0.256	1.210	-0.799	0.021
**PIK3C2G**	-0.147	9.997	-0.673	2.773	0.378	14.770	-0.438	23.450	-0.399	0.213
**PIK3CD**	0.053	44.812	-0.618	3.009	0.125	9.255	0.155	41.825	0.451	0.021
**PIK3IP1**	0.137	49.540	0.416	0.514	0.224	9.255	0.163	49.358	0.437	0.021
**PIK3R1**	-0.158	9.997	0.546	1.086	0.482	22.899	-0.686	0.061	0.528	0.649
**PIKFYVE**			0.468	0.944	0.027	58.611	0.276	0.573	-0.313	11.001
**PIN1**	0.115	44.812	0.160	9.629	0.152	4.053	-0.153	16.936	-0.449	0.471
**PIP5K3**	0.033	49.540	-0.444	0.445	-0.233	0.399	0.039	55.771	0.100	33.879
**PITPNC1**	-0.335	0.052	0.366	2.859	-0.244	29.328	-0.492	5.543	-0.519	0.021
**PKD2L2**	0.087	44.812	-0.253	6.703	-0.052	54.331	0.068	47.420	0.477	0.649
**PKIB**	-0.296	0.930	0.522	0.784	-0.464	1.098	0.326	2.913	-0.779	0.119
**PKM2**	-0.178	55.870	0.137	4.122	-0.607	0.092	-1.365	0.061	0.185	14.822
**PKN2**	-0.163	3.157	0.600	1.772	-0.188	10.694	-0.491	7.099	0.682	0.021
**PKP3**	0.043	52.827	-0.073	10.404	0.165	54.331	0.056	49.358	0.540	0.021
**PLA2G16**	-0.071	56.874	-0.144	8.253	0.486	0.240	0.174	13.893	0.877	0.021
**PLA2G2F**	0.008	56.513	-0.480	0.829					-0.067	33.879
**PLA2G4C**	0.091		-0.382	0.669	0.045	49.127	0.073	41.825	0.231	14.822
**PLA2G5**	-0.160	54.701	0.237	5.829	0.499	0.240	-0.189	47.420	0.506	0.039
**PLAA**	0.453	0.324	0.360	0.784	0.073	55.771	-0.201	0.947	-0.098	24.398
**PLAGL1**	-0.356	0.052	-0.519	4.122	0.202	8.121	0.193	16.936	0.309	33.879
**PLAUR**			-0.096	9.629	0.326	1.309	0.392	0.109	0.647	0.021
**PLCB1**	0.352	0.529	0.414	2.773	-0.531	0.092	-0.241	41.825	-0.632	0.021
**PLCb2**	0.139	1.936	0.171	9.629	-0.313	20.025	-0.090	41.825	0.989	0.021
**PLCE1**	0.544	0.780	-0.133	5.829	0.133	12.575	0.761	0.061	1.016	0.021
**PLCG1**	0.084	53.690	-0.418	0.859	0.611	2.659	0.139	13.893	0.093	33.879
**PLCG2**	0.172	4.284	-0.276	4.122			0.088	49.358	0.661	0.471
**PLCZ1**	-0.524	0.052	0.305	5.829	0.354	4.733	0.661	0.061	0.324	24.398
**PLD4**	0.208	1.936	-0.118	5.829	0.059	54.331	0.171	19.971	0.527	0.129
**PLEC**	-0.851	0.126	0.538	0.107	0.187	4.053	-1.479	0.061	-0.907	0.066
**PLEKHA3**	0.006	57.109	-0.061	11.093	0.156	29.328	-0.188	0.353	-0.367	0.084
**PLEKHA5**	-0.452	0.052	0.493	0.514	0.024	58.406	-0.039	47.420	-0.063	35.531
**PLEKHA6**	-0.398	0.930	-0.366	0.784	0.473	1.309	-0.166	52.124	-0.142	4.218
**PLEKHG3**	-0.358	0.262	0.407	0.595	-0.827	0.092	0.433	0.061	0.539	0.166
**PLIN1**	0.094	44.812	-0.122	6.703	-0.368	33.124	0.112	27.828	-0.481	0.977
**PLIN2**	-0.162	37.036	0.192	4.122			0.326	1.878	0.684	0.045
**PLK3**	0.444	0.093	0.098	4.122	0.095	10.694	-0.077	41.825	0.073	40.660
**PLOD1**	0.057	52.827	-0.254	2.859	-0.173	54.331	0.131	47.420	-0.513	0.166
**PLP1**	-0.170	1.936	-0.168	4.122	0.123	12.575	-0.190	41.825	0.605	0.021
**PLSCR1**	-0.893	0.170	0.120	11.783	0.051	59.432	0.238	8.987	0.600	0.021
**PLSCR2**	-0.941	0.052	0.278	6.703	0.271	0.240			1.096	0.021
**PLXDC1**	-0.068	55.870	-0.167	2.859	0.125	49.127	0.246	1.617	0.570	0.166
**PLXDC2**	-0.071	54.154	-0.590	3.205	-0.156	3.475	0.024	55.172	0.657	0.021
**PLXNA2**	0.106	51.013	-0.087	8.253	0.031	56.823	-0.135	32.497	-0.432	0.061
**PLXNB1**	0.086	49.540	0.399	0.935	0.206	1.309	-0.233	0.855	0.172	31.624
**Pmaip1**	-0.382	3.157	-0.410	4.122	0.152	33.124	-0.580	27.828	0.578	0.119
**PMCH**			-0.378	7.537	-0.227	8.121	0.177	47.420	0.677	0.021
**PMS2**	-0.259	44.812	0.263	2.123	-0.199	54.331	0.549	0.727	-0.227	11.001
**PNMT**	0.045	44.812	-0.125	6.703	0.032	55.771	0.454	0.573	-0.089	31.624
**PNN**			0.389	0.174	-0.125	26.120			-0.367	0.021
**PNOC**	0.313	1.431	-0.184	8.881	0.043	55.771	-0.544	0.061	0.097	43.008
**PNPLA2**	0.092	49.540	-0.360	4.122	-0.094	36.892	0.214	4.906	0.665	0.021

**POFUT1**	0.171	3.157	-0.264	4.122	-0.234	0.659	-0.846	0.061	0.146	42.836
**POLE**	-0.390	3.157	0.406	0.428	-0.107	49.127	0.635	23.450	0.417	0.993
**POLG2**	0.312	29.668	0.322	0.602	-0.023	60.505	0.462	0.061	0.342	0.746
**POLH**	0.042	54.701	-0.647	0.514	0.404	3.475	0.205	13.893	-0.184	2.886
**POLI**	0.224	3.157	-0.154	4.122	-0.176	17.386	0.513	0.061	0.279	1.977
**POLK**	0.015	56.874	0.461	4.122	-0.145	17.386	-0.443	0.061	-0.395	0.649
**POLL**	0.156	15.278	-0.446	2.773	0.177	4.733	-0.219	27.828	-0.423	0.746
**POLM**	-0.142	3.157	-0.588	0.784	-0.265	0.402	0.133	27.828	0.131	14.822
**POLR1E**	0.469	0.052	0.475	0.514	0.016	58.406			-0.187	8.002
**POLR2A**	-0.085	25.771	-0.406	0.893	-0.402	5.705	-0.042	52.656	0.064	40.660
**POLR2I**	-0.060	54.701	-0.413	0.784	0.175	6.932	0.130	19.971	0.441	0.391
**POLR3A**	-1.005	0.398	-0.825	0.300	-0.255	2.311	-0.246	0.130	-0.350	14.822
**POLR3E**	-0.398	0.171	0.395	1.534	0.068	49.127	0.354	41.825	-0.617	0.021
**POLR3F**	-0.437	0.511	0.558	0.514	0.261	12.575	-0.472	0.177	-0.273	11.001
**POLR3H**	0.087	33.231	0.261	3.205	0.161	49.127	0.161	47.420	-0.744	0.039
**POLR3K**	-0.159	44.812	0.573	1.086	0.254	8.121	0.427	41.825	-1.136	0.021
**POLRMT**			-0.086	11.783	0.052	49.127	0.332	3.866	0.473	0.129
**POM121/POM121C**	0.150	1.431	0.344	2.123	-0.216	49.127	-0.947	0.109	-0.793	0.021
**PON3**	0.092	44.812	-0.318	0.514	0.513	0.092	0.540	0.341	0.843	0.021
**POPDC2**	0.246	1.936	0.171	6.703	-0.251	3.475	-0.250	0.121	-0.543	0.021
**PPAPDC1A**	-0.307	3.157	-0.392	0.300	-0.295	1.556	-0.087	53.272	-0.109	31.624
**PPARG**	-0.136	1.431	-0.398	0.805	0.108	29.328	0.099	32.497	0.329	11.001
**PPARGC1B**	-0.269	37.036	0.489	1.086	-0.502	0.092	0.026	52.656	0.431	0.021
**PPFIBP2**	0.141	25.771	0.764	0.669	0.592	0.092	-0.457	32.497	0.350	0.166
**PPHLN1**	-0.284	44.812	0.601	1.212	-0.096	49.127	-0.061	47.420	-0.650	0.021
**PPIC**	0.011	56.513	0.872	2.300	-0.125	33.124	-0.122	47.420	-0.360	0.021
**PPM1D**	0.285	0.529	0.210	3.009	-0.133	41.103	-0.302	0.506	-0.390	0.213
**PPM1H**	0.065	44.812	-0.368	0.267	0.335	4.733	-0.260	4.906	-0.908	0.021
**PPM1L**	-0.437	0.126			-0.169	17.386	-0.218	41.825	-0.342	0.891
**PPP1R12B**	0.124	44.812	0.563	0.595	-0.103	55.771	0.124	41.825	-0.175	33.879
**PPP1R14A**	0.049	52.218	0.282	5.829	0.077	41.103	-0.048	47.420	0.433	0.601
**PPP1R15**	0.283	29.668	0.376	0.877	0.369	0.092	0.127	54.403	-0.450	0.084
**PPP1R3C**	0.107	7.743	0.440	0.514	0.105	54.331	0.024	53.272	0.517	0.391
**PPP2R1B**	0.100	49.540	-0.665	0.180	-0.199	49.127	-0.117	13.893	0.485	0.993
**PPP2R2B**	-0.244	1.431	0.200	5.829	-0.144	20.025	-0.321	19.971	-0.555	0.021
**PPP2R3A**	0.067	49.540	-0.495	0.445	-0.698	0.092	-0.419	0.061	-0.062	39.468
**PPP3R1**	-0.076	44.812	0.756	0.784	-0.073	55.771	-0.284	0.177	0.690	0.119
**PPP5C**	0.292	9.997	0.548	4.122	0.205	20.025	0.140	47.420	-0.550	0.129
**PPP6R1**	-0.446	0.126	-0.151	5.829	0.022	55.771	-0.318	47.420	-0.409	0.084
**PPTC7**	-0.068	51.013	0.216	0.264	-0.124	9.255	0.139	41.825	0.551	0.084
**PRCP**	0.335	0.780	-0.600	0.829	-0.374	4.053	0.171	19.971	0.596	11.001
**PRDM2**	0.592	0.170	0.509	0.877	-0.315	10.694	-0.607	41.825	-0.654	0.066
**PRDX6**	-0.471	1.936	0.531	1.086	-0.135	56.823	-0.117	32.497	0.561	0.129
**Prdx6b**	0.125	37.036	0.250	4.122	0.031	54.331	0.101	16.936	0.570	0.021
**PRELP**	-0.283	0.511	-0.375	4.122	0.206	0.641	0.183	11.241	0.555	0.021
**PRICKLE1**	-0.612	0.052	0.278	1.086	-0.261	14.770	-0.029	52.656	0.537	24.398
**PRIM1**	0.438	0.052	-0.205	8.253	-0.054	59.432	0.310	1.617	-0.039	39.468
**PRIM2**	-0.374	44.812	-0.462	0.823	-0.709	9.255	-0.092	13.893	0.256	4.218
**PRKAA2**	-0.152	1.431			0.146	36.892	0.047	53.829	-0.766	0.021
**PRKACB**	-0.438	29.668	0.379	3.205	0.168	17.386	-1.250	0.341	-0.373	0.746
**PRKAR2A**	0.187	5.928	0.168	8.253	0.073	54.331	-0.099	41.825	-0.840	0.021
**PRKAR2B**	-0.257	2.450	-0.427	0.784	0.314	4.733	0.044	53.272	0.035	43.476
**PRKCA**	0.131	9.997	0.663	1.772	-0.137	49.127	-0.121	47.420	-0.426	0.039
**PRKCD**	0.110	49.540	-0.155	5.829	-0.305	49.127	0.608	0.061	0.744	0.213
**PRKCDBP**	-0.184	44.812	-0.528	0.877	0.026	59.793	0.205	0.573	0.575	0.021
**PRKCE**	-0.139	52.827	0.635	0.944	-0.195	36.892	-0.356	0.506	-0.387	0.021
**PRKCQ**	-0.445	12.647	-0.219	3.009	-0.172	2.311	-0.774	0.543	0.332	24.398
**PRKD3**	-0.219	3.157	-0.426	5.829	0.113	9.255	-0.621	0.061	0.585	0.649
**PRKG1**	-0.597	0.052	0.558	0.514	0.484	0.092	0.213	1.332	-0.370	1.977
**Prl3b1**	-0.112	7.743	-0.481	0.669			-0.045	49.358	0.006	44.315
**PRMT3**	0.137	33.231	0.460	0.514	0.175	3.475	-0.404	0.061	-0.454	0.391
**PROCR**					0.294	49.127			1.287	0.021
**PROP1**	-0.047	49.540	-0.398	0.669	0.034	55.771			0.218	44.315
**PROS1**	-0.385	0.052	-0.381	1.086	-0.151	6.932	0.423	16.936	0.573	0.039
**PROSC**	0.487	3.157	-0.594	2.614	-0.873	17.386	0.565	0.061	0.803	0.391
**PRPF40A**	0.213	21.797	0.614	0.484	-0.164	5.705	0.228	7.099	-0.432	0.307
**PRPF4B**	0.133	49.540	0.659	1.029	0.158	26.120	0.585	0.573	0.576	0.021
**PRR13**	-0.116	49.540	0.589	0.107	0.119	33.124	-0.031	52.656	0.104	44.052

**PRR15L**	0.102	33.231	-0.409	0.784			-0.062	50.493	0.091	44.315
**PRR16**	-0.657	0.529			0.046	58.928	-0.527	0.061	-0.977	0.129
**PRR5L**	-0.745	0.093	0.210	1.086	-0.072	36.892	-0.205	47.420	0.253	36.831
**PRRC2C**	0.275	0.398	0.341	0.300	0.136	17.386	0.152	52.124	0.719	0.119
**PRRX1**	-0.451	0.780	-0.666	2.859	-0.675	0.092	-0.549	1.878	0.617	0.649
**PRRX2**	-0.273	33.231	0.234	0.300	0.062	58.074	0.818	0.061	1.262	0.021
**PRTG**	-0.194	4.284	0.318	0.534	0.476	0.092	0.261	47.420	0.233	1.977
**PSCD4**	-0.100	57.293	-0.401	3.009	0.059	49.127	0.632	0.292	0.935	0.021
**PSD3**	-0.278	7.743	-0.498	0.784	-0.203	17.386	-0.544	1.210	-0.510	0.993
**PSMA6**	0.097	51.013	0.644	0.180	-0.080	29.328	0.056	50.493	-0.169	6.001
**PSMA8**	-0.084	37.036			0.763	0.092	0.349	13.893	-0.125	36.831
**PSMB11**	0.105	15.278			-0.260	10.694	0.227	47.420	-0.488	0.045
**PSMB8**	0.052	52.218	0.613	1.663	0.110	54.331			0.619	0.021
**PSMB9**	0.299	0.511	0.285	2.773			-0.150	13.893	0.447	0.119
**PSMC3IP**	0.199	7.743	0.769	0.457	-0.085	36.892	-0.331	0.855	0.229	36.831
**PSMC5**	0.123	5.928	0.683	1.029	0.115	22.899	0.314	0.109	0.472	0.213
**PSMD7**	0.103	37.036	0.654	0.595	-0.089	26.120	0.313	0.399	0.475	0.021
**PSME4**	0.188	1.098	0.380	0.514	0.093	49.127	-0.079	27.828	-0.358	0.993
**PSMF1**	0.265	0.171	0.659	0.514	0.126	41.103	-0.351	2.415	1.318	0.021
**PSPC1**	-0.030	56.513	0.572	1.534	0.081	26.120	-0.068	49.358	-0.525	0.021
**PTBP1**	-0.064	51.013	0.120	9.629			0.070	47.420	0.486	0.119
**PTCHD1**	-0.546	0.052	0.084	5.829	0.320	49.127	0.150	50.493	0.142	40.051
**PTDSS2**	-0.128	25.771	-0.423	0.784	0.100	49.127	-0.071	51.354	-0.359	0.977
**PTGDS**	-0.541	0.930	-0.092	7.537	0.326		-0.071	55.172	-0.241	44.203
**PTGES**	0.333	0.930	0.178	0.669	0.140	49.127	0.221	27.828	0.501	0.021
**PTGR1**	0.109	33.231	0.492	0.445	0.113	22.899	0.084	52.656	0.348	0.021
**PTGS2**	1.082	0.052	0.483	2.300	0.232	3.068	0.859	0.061	-0.939	0.021
**PTH1R**	-0.620	0.262	0.199	1.772	0.110	12.575			0.501	0.977
**PTHLH**	-0.048	57.293	-0.529	1.534	0.147	33.124	-0.257	0.727	-0.424	0.891
**PTPLAD2**	-0.459	1.431	-0.145	4.122	0.484	3.068	-0.355	16.936	0.895	0.021
**PTPN21**	-0.701	1.431	0.462	0.204	0.019	60.231			0.198	40.051
**PTPN5**	0.029	54.154			-0.152	49.127	-0.074	27.828	-0.582	0.129
**PTPN6**	-0.043	52.218	0.482	3.205	0.278	12.575	0.390	41.825	1.393	0.021
**PTPRC**	-0.107	52.218	0.186	6.703	0.113	54.331	-0.431	23.450	0.999	0.021
**Ptprd**	0.244	5.928	0.104	11.093					1.128	0.021
**PTPRR**	0.080	52.218	0.449	1.534	-0.284	0.092	-0.200	0.727	-0.297	0.166
**PUS10**	0.118	33.231	0.360	1.086	0.192	0.659	0.064	49.358	-0.376	0.166
**PUS3**	0.154	7.743	0.455	0.784	-0.136	10.694	0.413	0.506	0.541	0.045
**PVRL3**	-0.097	56.513	0.610	0.595	-0.108	26.120	-0.830	0.855	-0.727	0.307
**PVT1**	-0.245	0.171	-0.894	0.230					0.278	24.398
**PWP1**	-0.060	54.154	-0.400	0.484	0.018	58.406	-0.042	51.354	-0.090	24.398
**PXDN**	-0.405	0.052	0.303	6.703	0.072	54.331	-0.196	1.878	-0.239	2.886
**PYCARD**	-0.101	52.218	-0.241	4.122	0.112	49.127	-0.076	53.272	1.009	0.021
**QRFPR**			-0.475	3.009	0.197	49.127			-0.333	2.886
**QRICH1**	-0.033	55.559	0.449	1.534	-0.120	49.127	-0.399	19.971	-1.115	0.021
**R3HDM1**	-0.451	0.262	0.525	5.829	-0.234	2.056	-0.242	41.825	-0.427	0.119
**RAB11FIP4**	0.392	0.262	0.476	2.300	0.112	12.575	-0.194	1.617	-0.351	0.993
**RAB1A**	0.193	29.668	0.550	1.772	-0.283	20.025	-0.329	0.292	-0.505	0.021
**RAB22A**	-1.026	0.052	0.239	0.669	0.336	41.103	-0.297	0.947	0.424	0.021
**RAB23**	0.130	37.036	-0.469	2.614	-0.123	33.124	-0.502	0.121	0.459	0.649
**RAB27A**	-0.433	0.052	0.447	2.614	0.327	2.659	1.023	0.061	-0.866	0.021
**RAB28**	0.146	25.771	0.540	0.986	-0.097	33.124	-0.645	0.061	-0.391	0.471
**RAB32**	-0.255	44.812	0.110	7.537	0.285	20.025	-0.264	1.210	0.938	0.129
**RAB3C**	0.342	0.126	0.660	1.534	0.075	49.127	-0.123	49.358	-0.545	0.045
**RAB3D**	-0.407	0.666	-0.623	2.614	0.068	36.892	-0.080	27.828	0.305	24.398
**RAB40B**	0.216	4.284	0.749	1.772	0.044	49.127	0.152	47.420	-0.251	0.993
**RAB7L1**	0.041	55.170	-0.279	4.122	-0.140	14.770	0.336	0.109	0.664	0.021
**RABIF**	0.169	9.997	0.662	1.534	0.053	54.331			-0.351	0.391
**RAC1**	0.219	0.171	0.454	0.877	0.016	60.505	-0.133	13.893	0.352	0.021
**RAC2**	-0.077	51.013	-0.460	1.663	0.015	60.505	0.450	0.855	0.901	0.021
**RALGAPA1**	-0.134	1.098	0.367	1.289	0.040	54.331	0.502	0.292	-0.270	0.746
**RALGPS1**	0.115	9.997	0.185	0.514	-0.164	1.819	-0.423	11.241	-0.484	0.213
**RALY**	-0.246	0.666	-0.700	0.663	0.065	41.103	0.028	55.172	0.230	31.624
**RANBP17**	-0.090	51.013	-0.167	0.514	0.503	0.856	-0.202	41.825	0.572	0.391
**RANBP3**	0.114	37.036	0.099	11.783	-0.194	12.575	-0.662	0.061	-0.578	0.993
**RAP1GAP2**	0.121	18.587	-0.147	6.703	0.134	49.127	-0.401	7.099	0.661	0.129
**RAP2B**	0.125	44.812	0.475	0.204	-0.137	3.068	0.026	51.354	-0.547	0.039
**RAPGEF3**	0.037	49.540	0.181	2.859			0.094	49.358	0.512	0.886

**RAPGEF6**	-0.170	44.812	0.491	0.174	-0.465	0.092	-0.159	47.420	-1.090	0.021
**RARB**	-0.497	0.398	0.501	0.300	-0.099	17.386	-0.142	47.420	-0.201	0.307
**RARRES1**			-0.218	9.629	-0.296	36.892	-0.763	0.061	0.367	31.624
**RARRES2**	-0.106	49.540	-0.243	3.205	0.075	54.331	0.111	27.828	0.812	0.021
**RASA2**	0.274	2.450	-0.893	1.663	-0.109	29.328	-0.139	47.420	-0.628	0.977
**RASA4/RASA4B**	0.032	51.013	-0.371	3.205	-0.037	56.823	0.439	0.061	0.423	0.084
**RASD2**	0.550	7.743	0.082	10.404	0.066	36.892	-0.228	41.825	0.495	0.977
**RASGEF1B**	-0.380	1.098	0.656	0.595	0.523	0.281	0.170	1.617	0.402	0.307
**RASGRF2**	-0.129	52.218	0.384	0.877	0.314	0.440	-0.219	3.866	-0.763	0.021
**RASGRP3**	0.011	56.765	0.396	0.986	-0.068	57.395	-0.178	41.825	0.597	0.039
**RASIP1**	0.199	15.278	0.109	8.253	0.060	49.127	-0.560	0.573	0.176	11.001
**RASL12**	-0.267	25.771	-0.084	6.703	-0.147	36.892	-0.525	0.906	0.082	42.554
**RASSF2**	0.206	3.157	-0.481	2.859	-0.308	0.402	-0.301	7.099	0.507	0.977
**RB1CC1**	-0.347	0.780	0.187	8.253	-0.284	0.517	-0.257	13.893	-0.484	0.391
**RBBP4**	0.161	44.812	0.554	1.534	-0.201	1.556	-0.230	19.971	0.506	0.746
**RBFOX2**	-0.198	0.511			0.013	58.928	0.281	32.497	0.496	0.021
**RBM8A**	-0.148	2.450	0.496	0.986	0.090	54.331	-0.715	0.061	0.440	11.001
**RBMS3**	0.054	52.827	0.460	0.420	-0.575	0.092	0.321	0.250	0.256	24.398
**RBMX**	0.150	18.587	0.605	1.772	-0.131	20.025	-0.345	0.109	0.632	0.129
**RBP1**	-0.322	21.797	0.481	0.514	0.057	56.823	-0.105	16.936	1.153	0.021
**RBP4**	-0.293	7.743	-0.272	5.829	0.658	0.092	-0.198	32.497	0.390	0.993
**RCAN1**	0.033	53.690	-0.482	1.212	-0.382	1.309	-0.530	0.177	-0.138	31.624
**RCOR1**	0.086	44.812	-0.563	0.595	0.258	0.659	0.536	0.061	-0.267	24.398
**RCSD1**	-0.293	44.812	0.123	10.404			-0.635	1.210	0.706	0.021
**RDH10**	0.376	0.126	0.684	1.663	0.272	0.402	-0.282	11.241	-0.405	8.002
**RDH5**	-0.153	49.540	-0.214	6.703	0.160	10.694	-0.160	47.420	0.630	0.391
**RECQL**	0.098	29.668	0.731	0.784			0.639	0.727	-0.230	1.193
**RELN**	-0.159	3.157	0.566	0.829	0.348	2.311	0.148	13.893	0.173	40.660
**REN**	0.409	0.171	-0.219	3.205	0.455	26.120	-0.563	4.906	-0.191	31.624
**RENBP**	0.173	1.936			0.234	0.641	0.160	27.828	0.843	0.021
**REST**	-0.148	29.668	0.335	0.784	0.472	0.659	0.340	0.727	0.175	33.879
**RETSAT**	0.197	25.771	0.297	0.784	-0.055	58.611	-0.425	0.061	0.709	0.021
**REV3L**	-0.431	0.171	-0.276	0.514	-0.078	26.120	-0.570	7.099	-0.456	0.166
**RFFL**	0.142	37.036	0.635	0.204	1.194	0.092	0.435	27.828	0.417	0.269
**RFX3**	-0.737	0.052	0.435	5.829	-0.425	0.092	-0.544	23.450	-1.070	0.021
**RGMA**	-0.134	49.540	-0.355	2.773	-0.333	0.856	0.505	0.855	0.777	0.021
**RGMB**	0.324	0.052	0.665	0.107	-0.130	17.386	-0.285	0.947	0.595	0.039
**RGS14**	-0.106	49.540	0.235	5.829	-0.304	22.899			-0.581	0.471
**RGS3**	-0.128	25.771	-0.478	2.859	0.076	55.771	-0.595	0.061	-0.214	24.398
**RGS6**	-0.544	0.324	-0.222	0.784			0.055	47.420	0.758	0.021
**RGS9**	0.245	1.098	0.743	0.602	0.373	6.932	-0.249	41.825	-0.383	0.021
**RHOBTB1**	0.409	0.052	-0.409	5.829	-0.065	55.771	-0.138	32.497	-0.163	33.879
**RHOC**	-0.280	15.278	0.162	2.773	0.140	14.770	0.046	52.124	0.713	0.021
**RHOD**	0.156	21.797	0.208	1.663	0.809	0.092	-0.056	54.403	1.108	0.021
**RHOG**	-0.221	1.936	-0.220	1.534	0.070	49.127	0.032	54.403	0.549	0.021
**RHOH**	0.543	0.052	-0.380	1.663	-0.352	0.517	-0.227	13.893	1.216	0.039
**RHOJ**	-0.378	0.052	0.229	1.086	-0.270	26.120	1.555	0.061	1.110	0.021
**RHOT1**	0.158	1.431	0.387	1.086	0.130	9.255	0.356	0.573	-0.324	0.021
**RHPN2**	0.371	0.780	0.546	0.986	0.361	20.025	-0.900	5.543	-0.016	41.878
**RIF1**	-0.414	0.093	0.609	1.534	-0.418	2.056	-1.006	0.061	0.416	31.624
**RIMS2**	0.154	15.278	0.565	5.829	0.057	49.127	-0.227	0.292	-0.661	0.021
**RIN2**	0.131	18.587	0.466	0.944	-0.087	29.328	0.142	41.825	0.285	24.398
**RINT1**	0.132	33.231	0.387	4.122	-0.151	4.733	-0.353	49.358	-0.188	31.624
**RLBP1**	-0.253	7.743	0.275	1.289	0.139	26.120	0.027	53.829	0.783	0.021
**RLBP1**	-0.253	7.743	0.275	1.289	0.139	26.120	0.027	53.829	0.783	0.021
**RLN1/RLN2**	-0.331	37.036			0.034	59.793	1.125	0.727	-0.225	11.001
**RMND1**	0.021	55.170	0.822	0.180	0.199	3.068	0.249	32.497	-0.175	0.891
**RNASEN**	-0.127	29.668	-0.712	0.569	0.268	5.705	-0.643	0.061	-0.207	31.624
**RND1**	0.036	54.701	-0.249	4.122	0.069	54.331	-0.146	23.450	-0.455	0.119
**RND3**	-0.316	0.171	0.529	5.829	0.048	54.331	0.146	47.420	-0.122	24.398
**RNF111**	-0.590	0.052	0.259	2.300	-0.340	4.053	-0.405	41.825	-0.173	2.886
**RNF121**	-0.839	0.052	0.394	0.877	0.066	49.127	0.151	41.825	-0.678	0.021
**RNF122**	-0.046	53.690	-0.114	9.629	-0.045	55.771	-0.077	47.420	0.542	0.021
**RNF128**	-0.224	4.284	0.829	0.877			-0.209	2.913	-0.709	0.021
**RNF130**	-0.166	7.743	-0.341	3.009	0.089	14.770	-0.030	54.831	0.454	0.129
**RNF135**	-0.110	49.540	0.062	10.404	0.170	9.255	0.472	5.543	0.391	0.891
**RNF141**	0.218	0.529	0.677	0.944	0.203	4.053	-0.930	0.061	-0.445	0.269
**RNF144B**	0.472	1.098	0.216	0.484	0.322	49.127	0.804	0.727	-0.324	31.624

**RNF185**	0.160	7.743	0.451	1.663	0.014	60.505	0.827	0.061	0.219	42.369
**RNF2**	0.071	33.231	0.386	2.123	0.120	49.127	-0.079	47.420	0.427	0.391
**RNF213**	0.080	44.812	-0.420	3.009	0.050	56.823	0.280	0.250	1.020	0.021
**RNF38**	0.058	52.827	0.260	4.122	0.389	0.440			-0.117	36.831
**RNF4**	-0.135	29.668	0.453	3.205	0.396	0.641	-0.799	8.987	-0.225	0.471
**RNF41**	0.054	49.540	-0.206	5.829	0.056	58.611	-0.851	0.341	0.051	41.878
**RNLS**	-0.173	5.928	0.198	3.205	0.014	58.928	-1.031	0.947	-0.254	14.822
**ROBO1**	-0.299	0.666	0.651	0.420	-0.238	20.025	0.345	41.825	-0.483	0.021
**ROBO3**	0.221	29.668	-0.455	2.123	0.236	10.694	0.182	16.936	0.708	0.021
**RP2**	-0.196	25.771	-0.749	0.893	-0.232	14.770	-0.210	32.497	0.361	6.001
**RPH3A**	0.029	56.513	-0.141	4.122	0.247	22.899	-0.367	0.061	-0.233	4.218
**RPL37**	0.154	9.997	0.327	5.829	0.592	0.092	-0.631	3.866	-0.418	0.993
**RPL39L**	-0.622	12.647	-0.746	0.514			-0.374	49.358	1.401	0.021
**RPL7A**	0.147	37.036	-0.372	1.772	0.066	49.127	0.164	3.866	0.540	0.129
**RPRM**	-0.970	0.511	0.713	0.107	0.532	0.240	-0.245	41.825	0.876	0.021
**RPS21**	-0.073	55.870	-0.436	0.514	0.078	54.331	0.164	41.825	0.839	0.021
**Rps24**	0.482	0.093	0.607	0.514	-0.224	33.124	-1.142	0.109	-0.664	0.021
**RPS6KB1**	-0.553	0.052	0.545	1.772	-0.425	36.892	0.189	1.210	-0.354	0.119
**RPS6KC1**	-0.200	2.450	0.285	1.663	0.394	0.402	0.172	23.450	0.618	0.021
**RRAD**	-0.332	1.936	0.270	0.514	0.405	9.255	0.367	0.727	0.493	0.021
**RRAS2**	-0.131	15.278	0.530	1.289	-0.158	29.328	0.128	41.825	-0.406	0.993
**RRBP1**	-0.541	4.284	-0.212	13.299	-0.313	0.517	-0.557	11.241	-0.955	0.021
**RRN3**	-0.457	0.052	-0.118	7.537	-0.141	8.121	-0.319	47.420	-0.598	0.021
**RRP1B**	-0.196	15.278	-0.981	0.445	-0.163	33.124	-0.630	2.913	-0.337	14.822
**RRP8**	-0.161	0.930	0.246	0.420	0.035	56.823	-0.528	0.341	-0.155	24.398
**RSPO1**	-0.670	0.093	-0.456	4.122	0.578	22.899	0.658	0.061	1.507	0.021
**RSPO2**	-0.927	7.743	0.766	0.420			-0.185	23.450	-0.831	0.021
**RSRC2**	0.196	37.036	0.394	1.029	-0.089	41.103	0.118	41.825	-0.357	0.166
**RTN4**	-0.506	0.052	0.760	1.772	-0.196	20.025	0.263	16.936	-0.399	0.021
**RTN4R**	0.176	29.668	-0.403	3.205	0.120	41.103	0.177	11.241	0.561	0.119
**RTP4**	-0.313	3.157	0.189	7.537	0.723	0.092	0.306	23.450	0.549	0.119
**RUNX2**	0.126	44.812	-0.240	8.253	0.072	55.771	0.536	3.866	-0.901	0.021
**RXRA**	0.076	44.812	-0.128	8.881	-0.199	9.255	0.287	0.292	0.463	0.039
**RXRG**	-0.714	4.284	-0.259	1.457	-0.058	49.127	0.912	0.292	1.023	0.021
**S100A1**	-0.119	51.013			0.025	59.793	0.106	23.450	0.679	0.021
**S100A10**	0.171	2.450	0.155	0.514	0.379	1.309	0.131	23.450	1.014	0.021
**S100A11**	-0.277	49.540	0.144	6.703	0.277	29.328	-0.057	50.493	1.102	0.021
**S100A13**	-0.090	18.587	0.346	0.107	0.150	41.103	0.115	4.906	0.597	0.021
**S100A16**	0.049	49.540	0.399	2.300	0.089	33.124	-0.119	5.543	0.404	0.039
**S100A3**	0.054	44.812	-0.223	3.009	0.298	0.641			0.418	0.993
**S100A4**	-0.127	44.812	-0.090	14.770	0.210	9.629	0.351	0.353	1.427	0.021
**S100A6**	-0.202	44.812	0.053	11.093	0.465	12.575	0.025	54.403	1.348	0.021
**S100B**	-0.125	51.013	-0.206	5.829	-0.049	57.395	0.143	47.420	0.545	0.101
**S1PR3**	-0.108	49.540	-0.179	3.205	-0.085	54.331	0.072	41.825	0.619	0.119
**SAG**	-0.076	49.540	0.526	0.986	-0.178	58.074	0.498	0.061	-1.044	0.084
**SAMD4A**	-0.535	0.052	-0.128	5.829	0.299	14.770	-0.313	0.341	-0.319	14.822
**SAMSN1**	-0.156	49.540	-0.198	5.829	-0.374	17.386	-0.291	1.617	1.495	0.021
**SAP130**	-0.202	25.771	-0.102	5.829	-0.199	4.733	-0.601	0.727	-0.966	0.066
**SAP18**	-0.196	0.930	0.650	1.212	-0.081	22.899	-0.642	0.061	-0.412	0.084
**SATB1**	0.337	21.797	0.315	4.122	-0.434	0.092	-0.569	23.450	-0.569	0.021
**SATB2**	0.150	33.231	0.753	0.986	0.507	1.309	0.079	52.124	-0.395	8.002
**SAV1**	0.182	4.284	0.134	7.537	-0.047	54.331	0.122	47.420	-0.534	0.746
**SBNO2**	-0.027	57.109	-0.463	0.514	0.098	8.121	-0.142	2.913	-0.274	0.166
**SCAMP1**	-0.786	0.126	0.402	0.986			0.523	23.450	-0.461	0.119
**SCAMP5**	0.057	54.701			0.398	0.092	-0.117	41.825	-0.265	0.269
**SCAPER**	-0.213	21.797	0.419	4.122	-0.434	0.092	0.312	32.497	-0.247	14.822
**SCARA3**	0.217	33.231	0.270	0.514	0.139	36.892	0.051	53.829	0.471	0.021
**SCGB3A1**	-0.445	0.324	-0.355	0.514	0.369	17.386	-0.119	41.825	1.126	0.021
**SCIN**	-0.583	0.511	0.495	0.180	0.042	54.331	0.735	0.506	-0.236	14.822
**SCN1A**	0.270	37.036	0.231	11.783	0.021	60.231	-0.038	49.358	-0.408	0.021
**SCPEP1**	0.110	44.812	-0.454	4.122	0.034	56.823	0.381	16.936	0.398	0.977
**SCRG1**	-0.267	29.668	-0.325	2.614			0.101	32.497	0.616	0.021
**SDC4**	-0.303	7.743	0.740	0.514	-0.183	4.733	0.222	32.497	0.767	0.021
**SDF4**	-0.072	37.036	0.307	2.614	0.058	54.331	0.107	47.420	-0.432	0.471
**SDPR**	0.073	37.036	-0.399	0.595			-0.479	0.130	0.234	4.218
**SDSL**	-0.035	56.765	0.266	5.829	-0.074	36.892	0.090	51.354	0.479	0.269
**SEC23IP**	-0.243	0.930	0.628	0.784	0.174	3.475	-0.082	41.825	-0.359	0.129
**SEC63**	0.194	25.771	0.954	0.484	-0.091	41.103	0.137	4.906	-0.504	0.021

**SECISBP2**	0.054	44.812	0.699	0.180	0.025	58.928	0.554	0.061	-0.562	8.002
**SEL1L**	-0.329	0.052	-0.409	0.420	-0.278	10.694	0.372	19.971	-0.836	0.119
**SELENBP1**	0.092	37.036	-0.345	3.009	-0.093	55.771	0.131	41.825	0.457	0.891
**SELPLG**	-0.072	55.870	-0.426	2.773	0.122	5.705	0.098	47.420	0.557	0.021
**SELT**	0.390	15.278	0.379	1.289	0.102	54.331	0.446	1.878	-0.935	0.021
**SEMA3C**	-0.109	49.540	0.304	0.514	0.412	2.056	0.044	50.493	0.820	0.193
**SEMA3G**	-0.033	55.170	0.144	6.703	0.473	0.092	-0.156	52.656	-0.459	0.649
**SEMA5A**	0.038	52.827			-0.056	55.771	-0.143	41.825	-0.332	0.993
**SEPP1**	-0.174	12.647	-0.300	2.773	-0.069	54.331	0.136	27.828	0.524	0.021
**SEPT11**	-0.059	52.827	0.458	0.514	0.184	33.124	0.150	47.420	-0.621	0.021
**SEPT6**	0.227	0.511	0.637	0.569	0.256	4.053	0.350	0.061	0.443	0.084
**SEPT7**	0.087	49.540	0.241	2.773	-0.133	17.386	-0.105	32.497	-0.462	0.021
**SEPT8**	-0.196	44.812	-0.361	0.514	0.131	22.899	0.120	19.971	0.644	0.166
**SERBP1**	-0.042	57.109	0.660	0.267	0.107	10.694	-0.160	0.947	-0.185	4.218
**SERPINA1**	-0.089	37.036	-0.231	3.205	0.041	57.395	0.278	41.825	0.428	0.021
**SERPINA9**	0.147	15.278	-0.200	5.829	0.018	58.074	0.026	52.124	-0.463	0.066
**SERPINF1**	-0.172	44.812	-0.139	5.829	0.129	49.127	0.458	41.825	0.839	0.021
**SERPINF2**	0.300	0.052	-0.082	5.829			-0.224	41.825	0.980	0.021
**SERPING1**	-0.192	12.647	0.451	4.122	-0.044	58.611	0.359	1.332	0.594	0.307
**SERPINH1**	-0.309	21.797	0.083	7.537	0.127	9.255			0.708	0.021
**SERTAD1**	0.469	0.666	0.393	1.086	0.404	0.092	-0.023	53.272	0.436	0.649
**SERTAD4**	0.132	18.587	0.593	0.669	0.080	41.103	0.445	0.061	-0.036	41.258
**SESN3**	-0.153	49.540	0.655	0.354	0.060	49.127	-0.232	0.506	0.575	0.021
**SESTD1**	0.073	49.540	-0.075	12.522	-0.041	56.823	-0.159	13.893	-0.531	0.021
**SET**	-0.119	21.797	-0.390	4.122	-0.033	60.231	-0.096	41.825	-0.476	0.307
**SETBP1**	0.149	4.284	-0.333	2.859	0.144	49.127	-0.041	47.420	0.556	0.166
**SETD3**	-0.046	54.154	0.729	0.669	0.163	17.386	0.503	27.828	-0.788	0.021
**SETD5**	-0.380	3.157	0.399	7.537	-0.456	9.255	0.586	13.893	-0.975	0.021
**SETD8**	0.141	44.812	0.189	6.703	0.145	6.932	-0.394	0.506	-0.494	0.119
**SF3B3**	0.107	52.218	0.439	0.514	-0.201	12.575	0.143	41.825	-0.440	0.021
**SFMBT1**	-0.141	7.743	0.449	0.514	0.104	17.386	0.194	0.947	0.347	24.398
**SFRP4**	0.193	3.157			-0.420	0.724	0.229	49.358	-0.137	38.751
**SFSWAP**	-0.159	1.431	-0.547	3.009	0.069	49.127	0.245	8.987	0.486	0.213
**SFT2D2**	0.045	51.013	0.593	1.029	0.589	0.092	0.039	51.354	0.463	0.021
**SFTPC**	-1.247	0.052	0.250	0.602	-0.244	12.575	-0.737	3.866	-1.201	0.021
**SFXN1**	-0.104	52.827	-0.266	4.122	-0.192	2.659	-0.296	7.099	-0.493	0.021
**SGCZ**	-0.614	0.666	-0.424	3.009					0.032	31.624
**SGPL1**	-0.117	53.690	-0.151	5.829	0.275	1.309			0.443	0.129
**SGSH**	0.457	0.780	0.542	1.086	0.449	1.309	0.550	0.121	0.297	24.398
**SGTB**			0.531	1.772	0.187	2.659	-0.095	50.493	-0.453	0.391
**SH3BGRL**	0.040	49.540	0.370	5.829	-0.215	26.120	-0.297	11.241	-0.434	0.119
**SH3BP1**	0.134	33.231	-0.535	4.122	0.073	41.103	0.039	52.656	0.453	0.039
**SH3D19**	-0.421	0.171	0.486	2.859	-0.423	22.899	-0.395	4.906	-0.518	0.746
**SH3GL2**	-0.161	25.771	-0.275	3.205	-0.153	41.103	-0.169	0.727	0.771	0.021
**SH3GL3**	-0.414	0.511	-0.247	0.514	0.210	17.386	0.092	41.825	0.093	38.751
**SH3KBP1**	-0.512	0.170	0.749	0.669	-0.265	0.092	0.172	2.415	-0.012	41.258
**SH3PXD2B**	-0.088	44.812	0.263	8.253	0.343	4.733	0.193	41.825	0.490	0.193
**SH3RF1**	0.196	44.812	0.463	0.784	-0.362	36.892	-0.740	0.061	0.390	0.993
**SH3TC1**	-0.025	56.765	-0.185	8.881	0.260	14.770	-0.411	0.506	1.073	0.021
**SHANK2**	0.079	29.668	0.191	0.514	-0.165	8.121	0.340	0.353	0.588	0.021
**SHCBP1**	-0.284	0.936	-0.521	0.805	0.164	12.575	0.062	52.124	0.231	31.624
**SHMT1**	0.077	52.218	-0.334	3.009	-0.225	49.127	-0.690	0.061	-0.218	0.269
**SHROOM2**	-0.075	37.036	-0.149	10.404	0.104	29.328	-0.527	0.292	0.170	4.218
**SIK1**	0.190	37.036	0.539	0.107	0.408	0.724	-0.954	0.855	-0.536	0.021
**SIPA1L1**	0.203	37.036	-0.400	0.669	0.014	59.793	-0.143	27.828	-0.187	4.218
**SIPA1L3**	0.320	15.278	0.642	0.595	-0.086	49.127	0.017	55.172	0.073	40.660
**SIRT1**	0.136	29.668	0.891	1.029	-0.519	0.402	-0.245	27.828	0.455	0.021
**SIVA1**	0.016	55.170	-0.270	5.829			0.086	47.420	0.534	0.021
**SKAP1**	-0.169	52.218	0.179	7.537	0.397	0.641	0.468	1.210	-0.101	24.398
**SKI**	-0.492	0.930	0.157	0.944	-0.342	0.440	-0.336	23.450	-0.270	4.218
**SLA**	0.716	0.126	-0.336	3.205	-0.303	0.440	-0.289	27.828	0.561	0.021
**SLAMF9**	0.144	1.431	-0.285	3.205	-0.196	1.556	0.270	0.727	0.761	0.021
**SLC11A1**	0.046	52.827			0.018	57.395	-0.102	54.831	1.248	0.021
**SLC11A2**	0.160	9.997	0.517	1.086	-0.068	49.127	0.126	49.358	0.464	0.307
**SLC12A4**	-0.048	49.540	0.485	0.595	0.206	0.517	0.071	52.124	0.624	0.471
**SLC12A7**	0.080	49.540	0.301	0.944	-0.300	1.309	-0.136	47.420	0.416	0.391
**SLC14A1**	0.248	9.997	0.449	0.514	-0.202	3.475	0.233	0.341	0.771	0.021
**SLC15A2**	0.346	0.262	0.707	0.445	0.643	1.819	0.167	32.497	-0.263	4.218

**SLC16A1**	0.145	3.157	-0.169	5.829	-0.426	4.733	0.468	0.109	0.979	0.021
**SLC16A9**	0.010	57.293	0.156	5.829			0.515	0.061	0.095	36.831
**SLC17A2**	-0.148	0.529	-0.629	2.859	0.016	58.406	0.024	55.172	0.124	24.398
**SLC17A5**	0.307	0.171	0.428	5.829	-0.065	55.771	-0.479	0.353	-0.250	0.391
**SLC18A2**	-1.089	0.093	-0.197	0.784	-0.720	0.092	-1.013	0.250	-0.068	31.624
**SLC1A2**	0.265	0.398	-0.848	0.107	0.246	1.098	0.282	1.617	-0.822	0.021
**SLC1A4**	-0.104	44.812	-0.372	0.784	-0.126	22.899	-0.126	2.913	-0.133	31.624
**SLC22A1**	-0.364	7.743	-0.389	0.784	-0.153	10.694	-0.149	41.825	-0.277	11.001
**SLC22A15**	0.149	18.587	-0.417	4.122	0.048	55.771	0.209	41.825	0.533	0.066
**SLC22A2**	0.045	51.013	-0.636	0.300	-0.246	55.771			0.285	40.051
**SLC22A4**	0.035	53.690	0.338	0.514			-0.239	0.855	1.307	0.021
**SLC25A17**	0.166	18.587	0.352	0.204	-0.216	10.694	-0.458	0.061	-0.467	0.119
**SLC26A3**			-0.735	2.859	0.033	55.771	-0.038	49.358	-0.417	0.993
**SLC27A2**	-0.285	0.093	0.353	1.534	-0.523	1.819	-0.159	50.493	-1.739	0.021
**SLC27A3**	-0.043	56.765	0.476	2.773	0.258	0.517	-0.433	4.906	-0.241	6.001
**SLC27A5**	0.106	44.812	0.344	0.180	0.331	0.092	-0.729	0.617	-0.402	14.822
**SLC2A4**	-0.171	51.013	0.172	0.514	-0.340	4.733	-0.579	1.878	1.434	0.021
**SLC2A5**	-0.073	29.668	0.317	6.703	-0.090	41.103	0.478	0.061	0.324	11.001
**SLC31A2**	0.252	3.157	0.542	0.445	-0.231	41.103	-0.148	0.855	0.075	38.751
**SLC35D1**	-0.216	44.812	0.223	7.537	0.021	60.231			0.539	0.600
**SLC35D3**	0.161	37.036	-0.545	0.264	0.113	20.025	0.122	27.828	-0.103	39.468
**SLC35F3**	-0.132	3.157	-0.310	6.703	-0.107	8.121	-0.192	13.893	0.414	0.119
**SLC38A1**	-0.256	29.668	0.947	0.877	-0.207	12.575	-0.335	0.061	-0.704	0.021
**SLC39A12**	-0.635	7.743	0.309	8.881	-0.076	49.127	-0.160	32.497	0.410	0.021
**SLC39A14**	0.999	0.052	0.336	4.122	0.049	54.331	-0.066	32.497	0.166	8.002
**SLC46A1**	-0.183	44.812	-0.455	0.230	-0.112	26.120	0.144	32.497	0.279	1.977
**SLC47A1**	-0.364	0.171	-0.526	0.107	-0.325	10.694	0.590	16.936	0.313	37.824
**SLC4A10**	-0.336	0.052	-0.532	2.859	-0.281	0.641	0.240	41.825	0.707	0.021
**SLC6A13**	0.914	0.052	0.128	9.629	0.711	0.659	0.843	0.947	0.645	0.213
**SLC6A15**	-0.791	0.052	0.680	2.300	-1.074	0.092	0.506	41.825	-1.432	0.021
**SLC6A9**	-0.121	12.647	-0.257	6.703	0.073	41.103	0.301	2.415	0.456	0.993
**SLC7A1**	-0.134	1.431	-0.639	2.773	-0.051	49.127	-0.195	0.292	-0.466	0.084
**SLC7A3**	-0.166	7.743	0.290	4.122	0.028	57.395	0.532	0.506	0.546	0.119
**SLC7A7**	-0.241	44.812	-0.320	0.784	0.174	29.328	0.442	11.241	0.671	0.039
**SLC8A3**	-0.188	1.936	-0.422	0.663	0.054	55.771	0.068	50.493	-0.130	11.001
**SLCO1A2**	-0.164	0.324	-0.694	2.614	-0.035	57.395	0.066	49.358	0.216	24.398
**Slco1a5**	-0.108	25.771	-0.694	0.614	-0.035	57.395	0.066	49.358	0.216	24.398
**SLCO2A1**	-0.356	25.771	-0.404	0.784			0.465	3.866	-0.268	0.649
**SLCO2B1**	0.041	52.827	0.462	1.772	0.095	41.103	0.176	7.099	0.840	0.021
**SLCO3A1**	-0.062	51.013	0.066	8.253	-0.285	4.053	0.110	41.825	-0.503	0.021
**SLCO4A1**	-0.354	0.171	0.464	0.595	-0.556	0.092	0.549	16.936	0.769	0.021
**SLFN2**	-0.640	0.171	0.179	9.629	0.226	10.694	0.327	32.497	0.990	0.021
**SLIT2**	-0.464	0.052	-0.250	4.122	-0.101	54.331	-0.579	27.828	0.091	44.052
**SLMAP**	-0.360	33.231	-0.455	1.772	-0.409	0.659	-0.110	8.987	-0.445	0.039
**SLPI**	-0.204	2.450	-0.368	2.859	0.523	0.092			0.388	24.398
**SMAD3**	0.184	4.284	0.472	0.457	0.317	22.899	0.186	52.656	0.316	31.624
**SMARCA5**	0.069	51.013	0.803	0.595	0.062	54.331	0.143	50.493	-0.468	0.084
**SMC6**	-0.206	44.812	0.613	0.230	0.174	12.575	0.440	0.947	-0.232	0.129
**SMEK1**	-0.200	9.997	0.692	0.784	-0.399	4.733	-0.540	27.828	-0.895	0.021
**SMG6**	-0.557	0.052	0.600	0.514	-0.350	0.240	-0.568	8.987	0.604	0.119
**SMG7**	0.276	0.171	0.283	1.663	0.195	22.899	-0.932	0.061	0.401	0.021
**SMNDC1**	0.023	54.701	0.585	0.893	0.048	54.331	0.098	41.825	-0.283	6.001
**SMOC2**	-0.658	0.052	-0.373	0.180	-0.358	4.733	0.233	3.866	-0.222	24.398
**SMPD2**	-0.145	9.997	0.329	3.205	0.087	49.127	-1.044	0.061	0.182	33.879
**SMPD3**	-0.880	0.052	-0.490	0.267	-0.202	33.124	0.068	47.420	0.150	38.751
**SMS**	-0.265	33.231	0.517	0.107	-0.238	17.386	0.053	47.420	-0.946	0.021
**SMTNL2**	0.371	0.171	0.211	8.881			-0.638	0.061	-0.563	0.021
**SMUG1**	0.055	44.812	0.583	0.669	0.290	49.127	0.121	23.450	-0.444	0.993
**SMURF1**	0.258	0.324	0.298	0.300	0.252	0.659	0.088	23.450	0.509	0.119
**SNAP25**	0.011	56.513	0.233	10.404	0.205	54.331	-0.207	0.130	-0.481	0.119
**SNCA**	-0.127	51.013	0.262	5.829	-0.098	58.074			-0.429	0.746
**SNF1LK**	0.999	0.052	0.539	0.107	0.408	0.724	-0.954	0.855	1.405	0.021
**SNTG2**	-0.793	0.171	0.513	1.289	-0.171	54.331	-0.467	0.947	-0.953	0.746
**SNX16**	0.202	44.812	0.508	0.420	-0.149	20.025	-0.131	16.936	-0.491	0.066
**SNX33**	-0.093	49.540	0.205	0.514	0.198	9.255			0.410	0.021
**snx4**	0.061	49.540	0.475	1.289	0.121	55.771	-0.191	23.450	0.534	0.021
**SNX7**	-0.145	1.431	0.252	7.537	-0.059	59.432	-0.147	13.893	-0.446	0.021
**SNX8**	-0.068	52.218	-0.148	5.829	0.095	49.127	-0.427	0.061	0.069	39.468

**SOAT1**	-0.691	0.052	-0.447	4.122	0.291	22.899	-1.277	0.061	-0.497	0.993
**SOCS2**	-0.609	0.052	0.413	1.663	-0.400	0.092	-0.473	27.828	-1.770	0.021
**SOCS3**			-0.333	2.859	0.279	17.386	-0.306	0.130	0.813	0.021
**SOCS4**	0.196	44.812	0.707	0.669	0.118	29.328			-0.465	0.119
**SOCS6**	-0.057	55.170	0.542	0.107	0.018	58.406	0.503	1.332	-0.357	31.624
**SORCS2**	-0.049	54.154	-0.122	7.537	-0.229	49.127			-0.401	8.002
**SOS1**	0.576	0.930	1.399	0.107	0.176	14.770	0.348	0.399	-0.526	0.021
**SOSTDC1**			0.170	4.122	-1.042	8.121	0.535	1.210	-0.898	0.983
**SOX10**	-0.156	3.157	-0.559	0.784			0.111	41.825	0.484	0.166
**SOX11**	-0.546	0.262	0.626	0.944	0.114	49.127	1.134	0.061	1.246	0.021
**SOX14**	-0.156	0.930	-0.625	0.829	-0.042	54.331	0.037	53.272	0.164	11.001
**SOX15**	-0.460	0.930	-0.761	2.773			0.034	53.829	0.272	24.398
**Sox17**	0.037	51.013	0.693	0.514	0.247	1.556	-0.294	1.878	-0.456	0.021
**SOX21**	-0.411	0.171	-0.410	3.009	0.147	22.899	-0.621	0.061	-0.307	0.746
**SOX9**	0.183	15.278	0.592	0.354	-0.274	0.724	-0.336	0.250	0.838	0.021
**SP110**	0.412	3.157	0.294	8.881	-0.093	54.331	0.244	41.825	0.996	0.021
**SP4**	-0.410	0.093	-0.620	0.107	-0.419	1.556	0.283	0.353	0.354	0.471
**SPARC**	-0.276	0.171	-0.494	2.773	-0.179	5.705	0.077	47.420	0.842	0.021
**SPATA5**	0.110	44.812	-0.565	2.123	-0.077	49.127	0.380	13.893	-0.095	24.398
**SPINT2**			0.133	6.703	0.136	29.328	0.432	1.878	0.812	0.471
**SPOCK1**	0.211	0.324	0.169	6.703	-0.526	3.475	-0.433	11.241	-0.755	0.993
**SPOCK3**	0.056	51.013	-0.102	10.404	-0.418	1.556			-0.708	0.084
**SPON1**	0.045	52.827	0.378	4.122	0.333	3.068	0.492	0.947	0.266	24.398
**SPP1**	-0.396	0.324	0.892	0.107	-0.205	12.575	-0.667	0.855	1.517	0.021
**SPPL2A**	-0.257	0.529	0.530	0.212	-0.367	12.575	0.097	52.124	0.208	36.831
**SPRED1**	-0.300	0.262			0.187	41.103	0.106	41.825	0.548	0.471
**Sprr1a**	-0.043	49.540	-0.157	5.829			0.022	55.172	0.433	0.119
**SPRY4**	0.448	18.587	-0.398	0.784	0.169	29.328	-0.226	11.241	0.818	0.021
**SPSB2**	0.147	25.771			0.014	60.505	0.080	49.358	-0.516	0.993
**SPTBN1**	0.296	0.529	0.718	0.514	-0.628	0.724	0.289	41.825	0.530	0.993
**SPTLC2**	0.215	7.743	-0.560	1.086	-0.184	1.098	-0.513	0.177	-0.060	40.051
**SQRDL**	-0.179	0.666	0.384	0.420	0.271	41.103	-0.077	41.825	0.552	0.021
**SRCAP**	-0.494	0.052	0.175	0.986	-0.230	3.475	-0.265	0.130	0.221	4.218
**SRD5A1**	0.050	55.170	0.498	0.180	-0.128	29.328	-0.334	11.241	-0.436	0.129
**SREBF1**	-0.148	44.812			-0.049	55.771	-0.116	41.825	0.576	0.039
**SREK1**	-0.084	44.812	0.544	1.457	-0.256	1.309	-0.051	41.825	-0.418	0.021
**SRGAP1**	0.710	0.052	0.405	1.289	-0.384	6.932	-0.987	0.061	-0.549	0.021
**SRGN**	-0.431	0.171	-0.532	3.009	0.036	58.074	0.136	41.825	0.798	0.021
**SRL**					-0.255	29.328	0.088	50.493	-0.503	0.891
**SRPK3**	0.307	44.812	0.558	1.663	0.136	22.899	0.723	0.061	-0.437	0.066
**SRPR**	-0.360	0.666	0.577	1.457	0.167	5.705	0.283	19.971	-0.366	0.993
**SSBP1**	-0.147	5.928	0.795	0.986	0.224	12.575	-0.576	2.913	-0.385	0.166
**SSTR2**	-0.259	12.647	-0.168	0.514	-0.391	3.475	-0.209	16.936	-0.627	0.391
**SSX2IP**	0.264	44.812	0.234	8.881	0.153	41.103	-0.557	0.855	-0.250	4.218
**ST14**	-0.113	44.812	-0.560	2.859	-0.057	54.331	0.077	27.828	0.423	0.746
**ST18**	-0.304	44.812	0.271	8.253	0.043	58.928	-0.893	0.250	-0.312	24.398
**St3gal3**	-0.839	0.052	-0.303	6.703	0.341	33.124	0.831	0.130	0.570	0.993
**ST5**	-0.119	49.540			0.402	3.475	-0.135	41.825	-0.529	0.307
**ST6GALNAC2**	-0.103	52.218	-0.392	0.784	0.421	0.402	0.512	0.121	0.050	44.315
**ST8SIA6**	-0.481	0.093	0.544	2.300	-0.648	6.932	-0.625	2.415	-0.490	0.993
**STAM**	0.278	9.997	0.649	1.212	0.380	4.733	-0.056	47.420	-0.492	0.021
**STAP2**	0.138	49.540	-0.249	2.123	-0.188	20.025	-0.273	16.936	-0.844	0.039
**STAR**	0.251	5.928	0.769	0.595	0.318	3.068	-0.350	50.493	-0.594	0.021
**STARD8**	-0.187	2.450	0.182	7.537	0.570	0.517	-0.394	1.617	0.889	0.166
**STAT1**	-0.313	0.324	0.528	0.264	-0.210	0.641	-0.603	0.250	-0.628	0.021
**STAT3**	-0.252	1.431	0.406	0.669	0.099	54.331	-0.291	13.893	0.276	31.624
**STAT5B**	-0.635	0.666	-0.149	7.537	0.348	1.309	-0.202	11.241	0.589	0.045
**STBD1**	0.820	0.052	0.571	0.569	-0.458	10.694	1.105	11.241	-0.235	31.624
**STC1**	-0.345	0.052	0.308	1.772	-0.354	4.733	-0.304	41.825	-1.496	0.021
**STEAP3**	-0.529	0.262	-0.249	4.122	-0.615	0.240	-0.392	47.420	-1.051	0.021
**STK35**	-0.409	15.278	-0.453	0.784	0.290	49.127	-0.292	19.971	-0.518	0.021
**STRADA**	0.020	56.874	-0.183	2.614	-0.197	36.892	0.085	47.420	-0.744	0.021
**STRBP**	-0.043	57.109	0.881	1.212	0.091	14.770	0.086	27.828	-0.270	0.119
**STRN**	0.413	0.930	0.822	0.354	-0.057	54.331	0.399	0.109	-0.157	4.218
**STT3B**	-0.266	21.797	0.419	0.514	0.072	49.127	-0.401	19.971	-0.720	0.269
**STX16**	-0.460	9.997	0.246	0.944	0.275	0.517	-0.317	1.617	-0.553	0.269
**STX3**	0.196	52.218	0.888	0.107	0.604	4.733	-1.209	0.250	0.573	0.891
**STX5**	-0.067	49.540	0.286	4.122	0.074	41.103	0.315	0.061	-0.487	0.021

**SULF1**	-0.680	0.052			0.090	55.771	0.137	47.420	1.352	0.021
**SULT1A1**	0.298	25.771	0.294	5.829	-0.353	3.475	0.184	50.493	0.566	0.119
**SULT6B1**	0.176	49.540	-0.692	0.784	-0.385	3.068	-0.309	47.420	-0.227	24.398
**SUPT3H**	0.030	52.827	0.577	0.514			0.150	50.493	-0.376	0.166
**SUSD3**	-0.225	1.098	-0.208	5.829			-0.103	49.358	0.402	0.045
**SUV39H2**	0.327	18.587	0.613	0.300	-0.350	17.386	0.666	4.906	-1.116	0.021
**SUV420H1**	0.434	15.278	0.565	0.174	-0.725	0.092	-0.331	27.828	0.049	41.258
**SV2B**	-0.082	29.668	0.548	2.773	-0.112	49.127	-0.047	49.358	-0.558	0.269
**SYCE2**	-0.069	55.559	-0.328	0.877	-0.529	5.705	0.275	0.109	-0.148	38.751
**SYK**	0.055	44.812			0.067	58.406	-0.771	0.130	0.388	0.391
**SYNCRIP**	-0.226	49.540	0.747	2.300	-0.787	0.281	0.264	7.099	-0.562	0.021
**SYNE1**	-0.234	5.928	0.631	2.123	-0.085	56.823	-0.223	2.415	-0.746	0.307
**SYNE2**	-0.339	0.052	-0.395	4.122	0.103	41.103	-0.915	0.061	0.582	11.001
**SYNGR1**	0.328	1.936	-0.609	1.663	0.135	26.120	-0.215	1.878	0.902	0.021
**SYNGR2**	-0.294	3.157	-0.093	8.253	0.174	0.856	0.296	7.099	0.471	0.471
**SYNJ2**	0.499	0.171	0.213	5.829	0.271	0.440	0.167	7.099	0.947	0.021
**SYNPO**	0.195	21.797	-0.263	4.122	0.392	6.932	-0.250	47.420	0.710	0.471
**SYP**	0.416	0.666	0.148	2.300			-0.098	5.543	-0.204	0.649
**SYPL1**	-0.256	0.171	0.539	4.122	0.171	1.309	0.526	0.061	-0.437	0.977
**SYT10**	0.489	0.052	-0.092	8.253	-0.073	54.331			-0.087	31.624
**SYT17**	-0.105	55.559	0.252	6.703	0.454	0.092	0.079	51.354	-0.523	4.218
**SYT4**	0.088	29.668	0.779	1.457	-0.169	12.575	-0.303	19.971	-0.513	0.269
**TAC1**	-0.437	12.647	0.516	0.445	0.168	2.311	-0.162	13.893	-0.906	0.269
**TAC10**	0.286	0.052	0.535	0.514	0.326	0.092	0.125	16.936	-0.412	0.021
**TAF9B**	0.111	49.540	0.776	0.669	0.046	54.331			-0.617	0.993
**TAGLN**	0.522	0.529	0.468	0.514	0.568	0.092	-0.631	0.109	1.103	0.021
**TANC1**	-0.032	57.367	0.669	0.107	0.126	49.127	0.227	16.936	0.228	0.391
**TARDBP**	-0.273	0.529	0.715	1.086	-0.135	4.053	0.351	23.450	-0.398	0.045
**TAS1R1**	0.070	44.812	-0.216	2.300	0.135	26.120	0.394	0.399	0.224	33.879
**TASP1**	0.221	21.797	0.601	0.300	0.302	8.121	-0.366	3.866	-0.285	1.193
**TBC1D2**	-0.178	0.511	-0.714	2.773	0.063	55.771	0.211	32.497	0.669	0.021
**TBC1D4**	-0.194	5.928	-0.178	4.122	-0.146	49.127	0.716	0.250	0.779	0.021
**TBPL1**	-0.265	1.431	0.847	0.569	0.256	6.932	-0.181	47.420	-0.364	0.993
**TBX1**			0.354	8.881	0.984	0.641	0.520	2.913	0.315	8.002
**TBXAS1**	-0.226	33.231	0.465	0.986	0.240	49.127	0.112	47.420	1.216	0.021
**TCF12**	0.358	1.936	0.427	0.877	-0.047	55.771	-1.118	0.399	-0.439	24.398
**TCF7L2**	-0.287	5.928	0.395	0.514	-0.335	1.819	-0.565	0.109	0.767	0.021
**TCP1**	0.154	44.812	0.893	0.420	-0.087	41.103	-0.220	4.906	-0.168	6.001
**TDG**	0.291	1.936	0.774	0.944	0.230	0.281	0.286	0.855	0.503	0.066
**Tdg**	-0.458	0.052	-0.633	2.614	-0.477	0.092	-0.170	1.617	-0.207	24.398
**TDP2**	-0.085	56.130	0.560	0.212	-0.223	0.819	-0.282	0.573	0.081	41.258
**TDRD5**	0.045	53.690	0.183	4.122	-0.251	26.120	-0.301	27.828	0.579	0.601
**TEK**	-0.540	0.052	-0.313	2.773	0.339	0.659	-0.195	23.450	-0.563	0.129
**TF**	-0.342	21.797	-0.484	0.784	0.794	3.068	0.296	27.828	1.212	0.021
**TFAP4**	-0.283	1.098	-0.680	2.859	0.151	22.899	0.300	23.450	0.515	0.213
**TFDP2**	-0.321	21.797	0.581	0.204	-0.179	26.120	0.499	0.727	0.594	1.193
**TFRC**	0.316	0.930	0.872	0.986	-0.094	36.892	0.260	2.913	-0.432	0.213
**TGFB1**	0.267	7.743	0.190	5.829	0.472	1.556	0.486	1.210	0.882	0.021
**TGFBR1**	0.106	25.771	0.105	8.881	-0.504	0.240	-0.089	32.497	0.374	0.166
**TGFBR2**	-0.061	54.154	0.460	3.205	-0.141	36.892	-0.463	0.573	0.601	0.021
**TGFBRAP1**	0.269	9.997	0.614	0.514	-0.124	54.331	-1.288	0.061	-0.443	0.021
**TGIF2**	-0.188	37.036	-0.186	9.629	0.228	17.386	0.292	8.987	0.688	0.021
**TGM1**	0.487	0.171			0.559	0.092	-0.455	7.099	1.353	0.021
**TGM2**	-0.573	0.052	-0.554	0.784	0.238	1.309	0.376	2.913	0.482	0.307
**Tgtp1**	-0.205	55.870	0.783	0.107	-0.714	0.281	0.191	41.825	0.541	0.021
**TH**	-0.107	33.231	-0.379	0.354	-0.503	9.255	0.156	23.450	0.615	0.746
**THAP2**	-0.209	29.668	0.780	0.602	-0.142	10.694	0.219	3.866	0.525	0.021
**THBD**	-0.082	51.013	-0.538	0.309	0.328	12.575	-0.063	47.420	0.220	31.624
**THBS2**	-0.271	1.431	-1.038	0.773					0.289	24.398
**THBS4**	-0.152	29.668	-0.196	2.859	0.424	0.819	0.164	41.825	0.226	31.624
**THOC2**	-0.189	1.098	0.222	4.122	-0.309	0.856	-0.396	0.855	0.454	0.993
**THPO**	-0.196	37.036	-0.652	3.205	0.278	4.733	-0.092	47.420	0.463	0.021
**THRAP3**	-0.148	49.540	0.591	0.445	-0.498	0.092	-0.866	1.332	-0.222	14.822
**THRB**	-0.138	44.812	-0.322	4.122	-0.154	17.386	-0.586	0.121	-0.898	0.119
**THSD7B**	0.064	52.218	0.906	0.944	-0.167	4.053	0.106	47.420	-0.396	0.891
**TIA1**	-0.107	51.013	0.712	0.595	-0.218	1.819	-0.293	0.109	-0.402	0.269
**TIAM1**	-0.160	33.231	0.060	8.881	0.116	49.127	-0.126	19.971	-0.521	0.269
**TIAM2**	-0.684	0.052	0.274	0.784	0.090	54.331	0.132	41.825	-0.900	0.993

**TICAM1**	0.024	56.513	0.143	2.614	0.287	0.402	0.225	16.936	0.515	0.021
**TIFA**	0.143	44.812	-0.376	5.829	-0.041	54.331	-0.061	41.825	0.508	0.649
**TIMM17B**	-0.133	5.928	0.296	5.829	0.334	36.892	0.174	27.828	-0.584	0.021
**TIMP1**	-0.318	12.647	-0.296	3.009	0.350	0.724	-0.363	8.987	1.332	0.021
**TIMP2**	-0.655	0.093	0.093	10.404	0.240	20.025	0.258	41.825	0.191	24.398
**TIMP4**	-0.366	0.398	-0.375	0.569	-0.177	8.121	0.204	2.415	0.042	43.175
**TIRAP**	0.174	33.231	-0.573	0.595	0.409	0.440	-0.949	0.061	-0.089	36.831
**TLE3**	0.172	5.928	-0.574	0.859	0.189	2.056	0.144	32.497	0.329	0.471
**TLE6**	0.036	49.540			0.381	26.120	-0.123	49.358	-0.431	0.891
**TLN1**	0.138	7.743	-0.158	0.784	0.234	2.311	0.107	41.825	0.590	0.021
**TLR1**	0.290	0.170	0.620	0.107	0.169	55.771	-0.274	49.358	0.955	0.021
**TLR2**	-0.521	1.431	0.191	6.703	0.427	2.311	0.676	11.241	1.571	0.021
**TLR7**	-0.149	56.874	0.209	8.881	0.370	3.068	0.148	41.825	0.724	0.021
**TM4SF1**	-0.168	5.928	0.118	11.783	0.186	12.575	0.109	23.450	0.646	0.021
**TM4SF5**	0.350	0.171	0.163	5.829	-0.039	57.395	0.946	0.061	-0.342	14.822
**TM6SF1**	0.127	37.036	0.747	0.877	0.015	60.231	-0.366	1.332	-0.587	0.021
**TMBIM4**	0.348	2.450	0.298	0.514	0.072	29.328	-0.232	41.825	0.572	0.021
**TMC6**	0.069	51.013	-0.289	0.784	0.581	0.092	-0.062	41.825	0.691	0.021
**TMCC3**	-0.325	0.052	0.062	11.783	0.332	0.641	-0.270	1.332	0.718	0.021
**TMEM132D**	-0.284	37.036	-0.560	0.457					0.182	35.531
**TMEM158**	0.099	44.812	0.102	8.881	-0.060	55.771	0.160	16.936	0.568	0.119
**TMEM163**	-0.213	18.587	0.510	1.457	0.193	29.328	-0.069	49.358	0.889	0.021
**TMEM173**	-0.230	0.529	-0.427	2.859	0.052	49.127	0.173	13.893	0.636	0.021
**TMEM176B**	-0.163	49.540	0.388	2.614	0.156	8.121	0.130	13.893	0.780	0.021
**TMEM189**					0.338	2.311	-0.374	23.450	0.695	0.021
**TMEM47**	-0.235	0.666	-0.828	0.773	-0.056	54.331	0.035	53.829	0.274	24.398
**TMEM51**	-0.061	53.690	0.263	0.602	0.527	1.819	-0.218	54.403	0.703	0.021
**TMEM57**	0.035	52.827	0.219	0.514	0.045	56.823	0.455	0.109	0.216	14.822
**TMEM63A**	0.165	5.928	-0.561	3.205	0.084	26.120	0.250	0.177	-0.351	0.993
**TMEM86A**	-0.354	0.126	-0.072	11.783			0.252	0.399	0.486	0.021
**tmf1**	0.183	44.812	0.649	1.029	0.070	49.127	-0.151	4.906	-0.421	0.084
**TMLHE**	0.211	18.587	-0.392	5.829	0.179	26.120	0.125	41.825	-0.386	0.084
**tmod1**	0.399	0.171	0.440	0.445	0.158	33.124	-0.292	1.617	-0.584	0.021
**TMOD2**	-0.571	0.511	0.494	0.514	-0.496	0.724	0.152	16.936	-0.341	0.119
**TMPRSS6**	0.279	21.797	-0.165	3.205	0.360	0.659			0.796	0.021
**TMSB10/TMSB4X**	0.178	33.231	0.310	1.663	0.250	29.328	-0.232	0.947	0.778	0.269
**TMTC1**	-0.207	0.511	0.763	1.457	0.188	10.694	-0.289	4.906	-0.497	0.021
**TMTC2**	0.215	0.666	0.199	0.944	0.036	56.823	0.098	49.358	-0.532	0.649
**TMX1**	-0.139	1.098	0.770	1.029	0.017	58.406			-0.472	0.021
**TMX4**	-0.106	44.812	0.680	1.772	0.156	49.127	0.074	52.124	-0.750	0.021
**TNFAIP2**	-0.063	44.812	-0.621	1.772	-0.038	58.406	-0.025	54.831	0.408	0.021
**TNFAIP8L2**	-0.161	29.668	0.193	2.773	0.541	0.402	-0.370	0.506	0.361	0.021
**TNFRSF21**	0.234	0.398	0.531	0.877	0.035	56.823	0.076	47.420	-0.608	0.649
**TNFRSF9**									0.692	0.021
**TNIK**	-0.207	25.771	0.759	1.289	-0.249	36.892	-0.413	32.497	-0.381	0.307
**TNKS**	-0.260	37.036	0.605	1.663	-0.345	0.641	0.144	23.450	-0.362	0.307
**TNNI2**	-0.156	33.231	-0.290	2.614	0.369	1.309	0.077	50.493	0.610	0.039
**TNS1**	-0.365	0.052	-0.354	3.205	0.424	0.724	-0.969	0.353	0.654	0.066
**TNS3**	0.233	15.278	0.680	0.204	0.127	49.127	0.221	16.936	0.340	6.001
**TNXB**	0.224	37.036	0.459	0.986	0.949	0.092	-0.230	47.420	0.638	0.649
**TOM1L2**	-0.346	0.262	0.430	0.445	0.633	0.092	-0.285	0.292	-0.494	0.213
**TOMM34**	0.268	1.936	0.251	7.537	0.099	54.331	0.120	50.493	-0.481	0.021
**TOMM70A**	-0.199	7.743	0.342	0.595	-0.102	49.127	0.049	47.420	-0.381	0.166
**TOR3A**	-0.126	52.827	0.558	0.267	-0.119	26.120	0.270	41.825	0.607	0.021
**TP73**	0.617	0.171	0.645	0.595	-0.460	26.120	0.642	0.353	0.269	1.977
**TPD52L2**	0.264	0.666	0.722	0.877	0.126	22.899	0.142	19.971	-0.367	1.977
**TPM1**			0.244	1.212	0.545	0.092	-0.272	16.936	-0.129	24.398
**TPMT**	-0.129	25.771	0.467	0.107	-0.347	3.068	-0.513	0.947	0.415	0.993
**TPP2**	0.354	0.052	0.443	0.180	-0.037	56.823	0.041	47.420	-0.117	11.001
**TPPP**	-0.065	44.812	0.240	5.829	-0.135	8.121	-0.164	8.987	-0.471	0.021
**TPPP3**			0.282	5.829	0.043	57.395	0.062	53.272	0.594	0.119
**TRA2A**	-0.234	18.587	0.583	1.457	0.261	3.068	-0.164	13.893	-0.567	0.021
**TRAF3IP3**	0.092	37.036	0.647	0.595	0.164	36.892	0.105	41.825	-0.557	0.021
**TRAF4**	-0.319	29.668	0.357	0.514	-0.127	54.331	-0.771	0.341	1.228	0.021
**TRAF5**	0.268	33.231	0.675	1.029	0.690	0.092	-0.453	1.332	0.460	0.471
**TRAPPC8**	0.299	0.262	-0.142	3.205	0.124	41.103	0.162	41.825	-0.367	0.039
**TRDMT1**	-0.118	37.036	0.199	12.522	-0.131	14.770	0.463	0.250		
**TREM2**	0.194	3.157	-0.681	0.569	0.042	56.823	0.115	47.420	1.283	0.021

**TREML1**	0.061	44.812			0.223	0.659	-0.862	0.061	-0.331	11.001
**TRERF1**	-0.328	0.052			0.165	49.127	0.515	0.061	0.619	0.066
**TRHDE**	0.214	18.587	0.547	2.773	-0.239	0.856	-0.177	4.906	-0.467	0.021
**TRIB1**	1.015	0.052	0.904	0.354	0.606	0.092	-0.049	55.172	-0.347	44.203
**TRIM25**	0.068	49.540	0.645	0.784	-0.182	9.255	-0.056	47.420	-0.474	0.119
**Trim30a/Trim30d**			-0.254	2.614	-0.663	0.092	0.457	19.971	0.674	0.649
**TRIM37**	-0.939	0.052	0.431	2.123	-0.650	0.092	-0.110	23.450	-0.298	0.119
**TRIM68**	-0.059	51.013	-0.148	8.253	-0.158	4.053	0.560	0.061	-0.792	0.021
**TRIM7**	0.128	15.278	0.131	5.829	0.272	8.121	0.058	52.124	0.463	0.471
**TRIP13**	0.198	5.928	0.448	1.534	-0.158	55.771	0.153	47.420	-0.667	0.021
**TRIP4**	0.095	51.013	-0.353	0.569	0.202	49.127	0.767	0.855	-0.781	0.021
**TRIP6**	0.031	55.170	0.266	1.457	0.227	0.641	0.783	0.061	0.438	0.021
**TRMT12**	-0.722	0.171	-0.774	2.773	-0.492	0.440	-0.354	41.825	-0.413	0.129
**TRMT1L**	0.125	18.587	0.659	0.514	0.079	54.331			-0.403	0.213
**TRMT6**	-0.533	0.052	0.270	11.093	-0.513	0.092	0.145	23.450	0.892	0.993
**TROVE2**	0.145	1.936	-0.591	1.457	-0.218	10.694	-0.376	0.573	-0.230	1.193
**TRPC2**	-0.322	3.157	0.756	0.514	0.668	1.309	1.166	0.061	-0.384	0.649
**TRPC4**	-0.266	0.780	-0.118	8.881	0.312	10.694	1.250	0.506	-0.241	0.649
**TRPM3**	0.017	57.293	0.217	1.534	-0.293	1.098	-0.646	0.061	-0.853	0.119
**TRPS1**	-0.742	0.052	0.410	0.264	-0.284	4.053	0.211	1.878	-0.774	0.021
**TSC1**	-0.485	0.052	0.577	1.534	0.430	10.694	-0.278	13.893	-0.627	0.021
**TSC22D4**	-0.445	0.052	0.489	0.669	0.270	26.120	0.716	0.061	0.635	0.993
**TSHZ3**	0.119	44.812	-0.633	3.009	-0.286	3.475	-0.804	0.061	-0.221	0.649
**TSN**	-0.357	0.171	0.456	0.420	-0.611	0.440	-0.397	0.061	-0.555	24.398
**TSPAN15**			0.573	0.569	0.276	1.819	-0.193	19.971	0.612	0.213
**TSPAN18**	0.050	49.540	0.094	8.253	-0.157	49.127	0.789	0.061	0.989	0.021
**TSPAN2**	-0.031	55.870	-0.352	4.122			0.366	0.061	0.644	0.307
**TSPAN4**					-0.079	49.127			0.522	0.391
**TSPAN9**	0.222	0.666	-0.136	6.703	0.334	0.402	0.046	53.272	0.485	0.129
**TSPEAR**	0.099	29.668	-0.806	0.309	0.280	0.856			0.306	0.307
**TSPO**	-0.449	18.587	-0.354	5.829	0.231	0.517	0.538	0.727	0.944	0.021
**TTBK2**	0.171	15.278	0.187	5.829	-0.185	1.556	-0.674	0.061	-0.505	0.021
**TTC30B**	0.216	5.928	0.510	1.534	0.080	36.892	0.112	49.358	-0.381	0.021
**TTC38**	-0.191	51.013	0.158	8.881	0.134	41.103	0.944	0.061	-0.375	31.624
**TTC7A**	-0.126	44.812	-0.357	3.009			0.187	0.573	0.539	0.021
**TTC9C**	0.298	0.511	0.686	1.772	-0.170	10.694	0.150	11.241	-0.478	0.269
**TTF1**	0.438	9.997	0.311	2.773	-0.374	0.659	-0.079	47.420	0.217	31.624
**TTF2**	-0.237	37.036	-0.310	0.784	-0.214	33.124	-1.229	0.061	0.333	6.001
**TTLL7**	0.032	56.130	0.368	2.300	0.294	2.311	0.167	55.172	-1.005	0.021
**TUBA1C**	-0.626	0.170	0.299	0.484	0.096	26.120	0.448	0.061	1.100	0.021
**TUBB2B**	-0.105	33.231	-0.285	5.829	0.056	49.127	-0.067	41.825	0.428	0.021
**TUBB6**	0.354	0.093	-0.291	4.122	0.269	33.124	0.568	0.399	0.739	0.129
**TUBGCP4**	0.129	7.743	0.786	1.029	0.099	36.892	-0.464	5.543	-0.696	0.021
**TULP1**	0.139	15.278	-0.669	0.944			0.045	52.656	0.128	31.624
**TUSC3**	0.082	44.812	0.633	1.289	-0.081	49.127			-0.433	0.166
**TWF1**	-0.556	2.450	0.520	2.614	-0.561	0.092	0.409	0.353	-0.623	0.307
**TWIST2**	-0.228	3.157	-0.144	5.829	-0.813	3.068	0.575	0.061	-0.612	0.947
**TXNDC9**	-0.464	0.780	-0.369	4.122	0.334	1.556	0.120	27.828	0.053	43.745
**TXNRD2**	-0.084	49.540	0.102	6.703	0.057	49.127	-0.482	0.109	0.048	40.660
**TYMS**	-0.218	0.511	0.453	0.514	-0.480	4.733	0.222	41.825	0.143	42.369
**TYRO3**	0.287	18.587	0.298	5.829	0.416	0.092	-0.176	13.893	0.064	43.175
**TYROBP**	-0.214	44.812			0.093	54.331	0.113	23.450	0.934	0.021
**U2AF1**	0.203	33.231	-0.622	0.663	0.126	3.068	0.176	27.828	-0.153	11.001
**U2af2**	0.071	54.701	-0.161	10.404	0.264	2.311	-1.260	0.061	-0.373	0.993
**UBA5**	0.341	33.231	0.496	0.514	-0.554	0.281	-0.751	0.109	-0.183	2.886
**UBAP2L**	-0.637	0.052	0.608	0.107	-0.746	0.092	-0.476	2.913	-1.192	0.021
**UBE2D1**	0.417	0.511	0.474	2.123	0.222	0.724	-0.254	5.543	-0.320	0.746
**UBE2D2**	0.059	49.540	0.703	0.595	-0.085	36.892	0.144	47.420	-0.479	0.307
**UBE2E1**	0.117	44.812	0.650	0.595	-0.179	8.121	-0.051	49.358	-0.460	0.021
**UBE2E2**	-0.040	57.367	-0.389	3.205	0.151	36.892	0.128	4.906	0.505	0.039
**UBE2I**	-0.372	0.780	0.319	4.122	0.105	41.103	-0.253	8.987	-0.490	0.993
**UBE2J2**	0.159	21.797	0.605	0.514	0.034	56.823	0.098	41.825	-0.182	2.886
**UBE2N**	0.157	21.797	0.550	0.180	-0.092	41.103	-0.464	0.130	-0.899	0.021
**UBE2T**	0.267	1.098	-0.294	3.009	-0.149	17.386	0.553	0.061	-0.057	35.531
**UBE2V2**	0.184	37.036	-0.408	0.595	-0.109	29.328	-0.145	2.913	-0.380	0.021
**UBE2W**	0.220	49.540	0.583	0.602	-0.111	22.899	0.258	47.420	-0.528	0.021
**UBE3A**	-0.195	25.771	0.929	1.029	-0.203	26.120	0.142	1.617	-0.448	0.021
**UBR2**	0.164	18.587	0.249	0.514	-0.203	0.724	0.445	0.061	0.234	38.751

**UBR3**	-0.481	0.052	-0.212	0.784	-0.716	0.402	0.110	41.825	0.206	24.398
**UCHL3**	0.132	44.812	0.613	0.264	-0.085	41.103			-0.063	38.751
**UCP2**	0.095	49.540	-0.273	0.602	0.021	58.406	-0.348	5.543	0.841	0.021
**UGT2B10**	-0.425	0.052	0.163	8.253	-0.600	2.659	0.977	0.109	1.541	0.021
**UMPS**	0.179	0.529	0.526	0.595	0.077	41.103	0.181	23.450	-0.172	24.398
**UNC45B**	0.036	51.013	-0.401	4.122	0.494	0.517	-0.226	13.893	-0.363	24.398
**UNC93B1**	-0.071	56.765	-0.306	3.205	0.081	41.103	-0.167	49.358	0.791	0.021
**UPF2**	0.537	29.668	-1.104	2.614	-0.397	0.092	-0.034	53.272	-0.329	14.822
**UPF3B**	-0.128	18.587	0.786	0.595	-0.207	1.556	0.467	0.506	-0.313	0.891
**UQCRQ**	-0.372	0.171	0.241	4.122	-0.171	5.705			0.217	39.468
**USH1C**	0.032	51.013	-0.403	0.205	0.011	60.231	0.088	41.825	0.198	8.002
**USO1**	-0.081	51.013	0.481	1.086	-0.053	57.395	0.259	32.497	-0.366	0.166
**USP13**	0.247	4.284	0.393	3.205	0.234	6.932	-0.044	47.420	-0.370	0.021
**USP15**	-0.903	0.052	0.553	1.663	-0.573	0.092	-1.159	0.061	0.803	0.993
**USP25**	0.183	33.231	0.647	0.595	0.119	6.932	-0.269	3.866	-0.175	0.746
**USP32**	-0.125	1.936	-0.358	4.122	0.058	49.127	-0.054	47.420	-0.459	0.021
**USP33**	0.105	44.812	0.532	1.457	-0.091	33.124	0.077	51.354	-0.435	0.021
**USP36**	-0.473	0.052	0.134	7.537	-0.407	0.092	0.640	19.971	-0.377	0.993
**USP39**	-0.108	52.218	0.811	0.595	-0.110	29.328	0.258	32.497	-0.375	0.021
**USP45**	-0.112	44.812			-0.125	49.127	-0.170	47.420	-0.494	0.649
**USP46**	-0.323	29.668	0.466	0.784	0.218	8.121	0.167	41.825	-0.541	0.021
**USP47**	-1.080	0.052	0.259	5.829	-0.533	1.309	0.695	0.399	-0.406	0.166
**USP53**	-0.984	1.098	0.561	0.180	0.076	41.103	0.113	41.825	0.416	0.307
**UST**	-0.045	52.218	-0.430	3.205	-0.213	8.121	-0.393	0.292	0.089	37.824
**UTP18**	0.208	29.668	-0.363	4.122	0.293	14.770	-0.574	0.250	0.129	33.879
**UTP23**	-0.205	7.743	0.817	0.107	-0.216	26.120	0.381	41.825	-0.869	0.021
**VAMP7**	0.019	54.701			-0.294	3.475	-0.287	0.573	-0.386	0.471
**VAMP8**	0.233	0.324	0.324	1.772	-0.079	55.771	-0.116	8.987	0.520	0.045
**VANGL1**	-0.576	0.262	-0.130	3.205	0.312	5.705	-0.168	11.241	0.402	0.993
**VANGL2**	-0.723	0.780	-1.035	1.029	0.366	0.281	-0.559	19.971	0.490	0.746
**VAPB**	-0.494	0.052	0.411	0.174	0.065	49.127	0.337	8.987	0.241	8.002
**VARS2**	0.101	33.231	0.249	0.986	-0.036	60.231	0.475	0.061	0.074	40.660
**VAT1L**	-0.241	33.231	0.249	5.829	0.322	0.092	-0.053	52.124	0.533	0.021
**VAV1**	0.486	2.450	-0.529	2.300	0.370	22.899	0.525	41.825	1.269	0.021
**VCAM1**	-0.184	1.431	-0.329	4.122	0.261	1.309	0.413	0.947	0.434	0.213
**VCL**	0.155	25.771	0.537	0.830	0.210	0.724	-0.174	0.573	0.348	0.993
**VEGFA**	0.229	0.262	-0.190	7.537	0.666	0.092	-0.767	5.543	-0.677	0.084
**VEGFB**	-0.058	53.690	-0.439	0.164	0.119	6.932	0.186	3.866	0.288	1.977
**VIM**	-0.392	0.052	0.638	0.445	0.198	0.856	0.298	1.332	1.573	0.021
**VPS13A**	0.164	0.930	0.521	0.595	-0.081	54.331	0.245	2.913	-0.525	0.066
**VPS24**	0.128	25.771	0.596	0.514	0.131	49.127	0.177	47.420	-0.112	37.824
**VPS35**	-0.424	0.171	0.232	2.614	-0.432	4.733	0.078	49.358	-0.353	0.021
**VPS36**	-0.443	44.812	0.611	0.180	-0.065	56.823	-0.468	0.250	-0.029	39.468
**VSNL1**	0.164	44.812	0.539	0.174	-0.076	41.103	-0.070	23.450	-0.547	0.045
**VSX2**	-0.301	33.231	0.196	8.253	0.069	58.406	-0.168	41.825	0.483	0.977
**VTA1**	0.027	55.170	-0.361	3.205	0.075	55.771	0.062	41.825	0.753	0.129
**VTI1A**	0.461	0.093	0.621	0.669	0.301	1.556	-0.360	49.358	-0.553	0.746
**VWA3B**	-0.098	49.540	-0.142	9.629	0.226	8.121	0.518	11.241	0.563	0.021
**VWF**	-0.827	0.052	0.829	0.300	0.181	10.694	-0.259	23.450	0.558	0.021
**WAPAL**	0.215	0.666	0.636	2.300	-0.076	41.103	0.128	41.825	-0.664	0.021
**WARS2**	-0.559	0.262	0.615	0.669	0.369	1.556	0.759	1.878	0.441	24.398
**WASF2**	0.075	51.013	0.304	0.440	0.298	8.881	0.180	19.971	0.638	0.021
**WASF3**	-0.127	55.559	-0.424	0.669	-0.043	58.611	-0.048	49.358	-0.191	4.218
**WBP4**	0.024	55.870	0.977	0.264	0.043	54.331	0.075	41.825	-0.578	0.021
**WBSCR17**	-0.133	49.540	0.477	0.986	0.491	2.056	-0.847	0.947	0.476	0.993
**WDFY3**	-0.503	15.278	-0.112	6.703	0.856	0.092	-0.168	1.617	0.550	0.021
**WDR11**	-0.856	0.052	0.411	5.829	-0.446	0.092	-0.304	47.420	-0.540	0.021
**WDR12**	-0.204	25.771	0.648	0.514	-0.810	0.092	-0.536	0.947	-0.377	4.218
**WDR20**	0.284	4.284	0.953	0.267	-0.223	2.659	-0.137	11.241	-0.425	0.021
**WDR33**	0.198	44.812	0.526	0.514	-0.106	41.103	0.228	27.828	-0.305	11.001
**WDR37**	0.134	44.812	1.092	0.986	0.169	41.103	-0.747	7.099	-0.617	0.021
**WDR59**	0.449	0.052	0.416	0.669	0.050	54.331	0.269	0.130	-0.713	0.084
**WDR70**	-0.111	18.587	-0.135	10.404	-0.182	4.733	-0.232	27.828	-0.475	0.891
**WDR77**	0.390	0.171	0.633	1.663	0.230	4.053	-0.518	0.061	-0.575	0.891
**WFDC5**	-0.139	51.013			-0.482	8.121	0.522	0.573	0.449	0.891
**WHSC1**	0.283	0.171	0.584	0.669	0.151	2.056	0.247	2.913	0.559	1.193
**WHSC1L1**	0.383	0.511	0.273	1.086	0.301	0.240	-0.579	0.061	-0.663	0.021
**WIF1**	-0.779	0.052	-0.170	6.703	-0.288	9.255	0.478	0.855	-0.081	38.751

**WISP1**	-0.299	4.284	0.129	13.299	0.292	29.328	-0.749	0.061	-0.682	24.398
**WISP2**	-0.253	5.928	-0.110	3.205	0.286	0.281	0.233	2.415	1.003	0.021
**WNK1**	0.126	44.812	-0.492	0.986	-0.315	1.098	##########	50.002	-0.964	0.021
**WNT3A**	0.498	18.587	0.837	0.107	0.244	26.120	0.333	0.506	-0.056	37.824
**WNT4**	0.056	55.170	0.347	5.829	0.284	2.056	0.456	0.947	-0.264	24.621
**WRN**	0.524	0.511	0.376	0.569	-0.246	4.733	0.741	27.828	0.460	0.021
**WSCD2**	0.102	33.231	0.053	8.881	0.017	58.611	0.134	41.825	0.618	0.601
**WWOX**	-0.445	0.052	0.376	0.602	0.210	3.068	0.204	13.893	-0.554	0.307
**WWP1**	0.266	15.278	0.561	1.663	0.162	4.733	-0.680	1.878	0.731	0.021
**XDH**	-0.569	0.262	-0.566	1.029	-0.222	26.120	-0.180	47.420	0.393	36.831
**XRCC2**	0.229	0.398	-0.570	0.944			-0.837	0.947	0.117	31.624
**XRN1**	0.148	25.771	0.213	0.514	-0.034	60.231	0.264	41.825	-0.375	0.993
**YARS2**	0.130	33.231	0.727	0.595	-0.058	57.395	-0.581	0.061	-0.500	0.021
**YEATS2**	-0.049	44.812	-0.052	13.299	0.075	41.103	0.231	7.099	0.423	0.391
**YLPM1**	-0.202	18.587	0.541	0.420	-0.961	0.092	-0.722	1.878	-0.557	0.166
**YTHDC2**	0.035	54.701	0.793	0.877	0.017	59.793	0.267	0.573	-0.297	6.001
**YWHAG**	0.070	49.540	0.898	0.267			-0.049	50.493	-0.434	0.021
**Ywhaq**	-0.384	0.052	0.636	0.514	-0.361	0.856	0.072	47.420	-0.215	2.886
**YY1**	0.328	37.036	0.795	1.534	-0.123	6.932	-0.503	8.987	0.434	0.649
**ZAP70**	-0.096	33.231	0.456	0.174	0.131	36.892	-0.216	1.878	0.436	0.891
**ZBTB10**	0.504	0.511	0.622	0.514	0.367	0.724	0.941	0.061	0.300	1.977
**ZBTB12**	-0.292	0.126	-0.370	0.514	-0.375	0.659	-0.294	0.727	0.931	0.021
**ZBTB20**	-0.125	44.812	0.451	1.772	-0.541	0.092	-0.226	4.906	-0.387	0.391
**ZBTB43**	0.112	15.278	0.438	0.107	0.080	54.331	0.165	11.241	0.098	38.751
**ZBTB5**	-0.087	44.812	-0.254	2.123	-0.302	0.641	-0.665	0.573	-0.620	0.021
**ZBTB7A**	0.238	12.647	0.125	10.404	0.231	20.025	0.250	3.866	0.617	0.021
**ZC3H12C**	0.525	0.052	-0.106	6.703	0.423	5.705	-0.602	1.878	1.026	0.021
**ZCCHC11**	-0.510	0.052	-0.360	4.122	-0.214	9.255	-0.242	23.450	-0.995	0.021
**ZCCHC7**	-0.421	0.093	0.698	2.300	-0.348	0.092	0.152	41.825	0.298	14.822
**ZCCHC8**	0.024	54.154	-0.349	0.784	0.056	58.406	-1.133	0.061	0.215	0.471
**ZDHHC21**	0.093	44.812	0.679	1.457	-0.210	1.098	-0.429	8.987	-0.703	0.119
**ZEB2**	-0.303	0.262	0.452	0.289	-0.374	29.328	-0.597	0.061	0.182	36.831
**ZFC3H1**	0.182	49.540	0.356	2.300	-0.088	41.103	0.668	0.177	0.172	8.002
**ZFHX3**	-0.915	0.171	0.769	0.174	-0.231	29.328			1.029	0.021
**Zfp386**	0.436	0.780	0.973	0.107	-0.429	0.440	-0.538	1.332	-1.625	0.021
**ZFP91**	-0.747	0.093	0.714	1.212	-0.332	4.733	-0.684	0.130	1.053	0.021
**ZFPM1**	0.183	2.450			0.251	8.121	0.067	49.358	0.597	0.269
**ZFR**	0.319	1.098	0.504	0.300	-0.346	0.724	-0.579	0.353	-0.265	24.398
**ZFYVE26**	-0.378	0.052	-0.606	0.784	-0.429	0.281	1.172	0.061	0.287	0.391
**ZIC1**	-1.544	0.052	0.386	2.773	-0.573	0.092	-0.335	32.497	0.403	31.624
**ZKSCAN1**	-0.432	0.052	0.732	0.602	0.041	56.823	0.274	2.913	0.071	40.660
**ZKSCAN2**	-0.078	33.231	-0.910	0.428	-0.063	49.127			0.424	0.977
**ZKSCAN5**	0.083	49.540	0.452	2.300	0.097	41.103	-0.371	0.947	-0.325	0.269
**ZMAT1**	-0.463	0.052	0.322	8.253	-0.403	0.092	0.220	1.617	-0.480	0.021
**ZMYM2**	0.192	7.743	0.568	0.484	-0.160	3.475	-0.205	13.893	0.349	0.891
**ZNF169**	-0.684	0.052			-0.242	6.932			-0.738	0.213
**ZNF175**	0.505	0.052	0.487	2.123	0.105	55.771	-0.737	1.878	-0.739	0.021
**ZNF202**	0.254	0.171	-0.284	4.122	0.168	2.056	-0.376	0.947	-0.258	11.001
**ZNF205**	0.135	44.812	0.663	0.893	0.089	29.328	-0.161	23.450	-0.215	8.002
**ZNF276**	0.245	0.511	-0.321	4.122	-0.119	54.331	-0.556	0.341	-0.298	6.001
**ZNF317**	-0.064	55.559	0.674	0.514	-0.148	6.932	0.638	0.061	-0.363	0.746
**ZNF385B**	0.420	12.647	-0.132	13.299	0.429	4.733	-0.123	32.497	1.182	0.213
**ZNF440/ZNF808**	0.529	5.928	0.168	5.829	0.641	0.092	0.459	19.971	-0.146	24.398
**ZNF451**	-0.212	0.529	0.705	0.514	0.494	3.068	0.400	0.341	-0.386	0.039
**ZNF469**	-0.281	0.052	-0.696	0.944	-0.114	29.328	0.350	8.987	0.135	31.624
**ZNF536**	0.389	0.052	-0.579	1.534	-0.164	9.255	0.116	47.420	0.441	0.307
**ZNF593**	-0.157	15.278	-0.310	4.122	0.096	49.127	-0.618	0.061	0.225	1.193
**ZNF642**	-0.148	33.231	0.586	0.784			0.223	2.913	0.818	0.021
**ZNF652**	0.109	21.797	0.399	0.164	0.224	9.255	-0.705	16.936	-0.038	40.660
**ZNF667**	0.025	53.690	0.586	1.212	-0.042	58.928	-0.144	19.971	-0.486	0.066
**ZNF668**	-0.783	0.052	-0.422	0.877	-0.873	0.641	-1.206	0.061	0.637	0.021
**ZNFX1**	0.356	0.780	-0.384	2.859	-0.150	26.120	0.397	27.828	-0.480	0.391
**ZNHIT6**	-0.096	51.013	0.533	0.784	0.088	41.103	0.336	11.241	-0.389	0.039
**ZNRF3**	0.313	5.928	0.423	0.180	0.279	9.255			-0.115	33.879
**ZWILCH**	0.122	44.812	-0.489	1.663	-0.317	2.659	-0.496	0.341	-0.122	31.624
**ZWINT**	0.193	4.284	0.502	0.174			0.327	23.450	0.275	0.213
