# Supplementary material for: Early Mechanisms of Pathobiology Are Revealed by Transcriptional Temporal Dynamics in Hippocampal CA1 Neurons of Prion Infected Mice
Source: PLoS Pathog. 2012 Nov 8;8(11):e1003002. doi: 10.1371/journal.ppat.1003002 (PMC3493483; doi:10.1371/journal.ppat.1003002)
Supplement: Table S2 — List of miRNAs that were differentially expressed during at least one time-point in prion infected versus mock infected mice (p-value≤0.1). The bolded miRNAs were found to be significantly deregulated during pre-clinical disease. (PDF) [file ppat.1003002.s010.pdf]

	70	70	90	90	110	110	130	130	EP	EP
let-7b-5p	1.15	-1.29	0.97	-3.97	0.00	0.00	-9.48	-6.30	-5.42	-5.70
let-7c-5p	1.73	1.32	-1.75	-3.65	-2.37	-2.66	-1.70	-2.23	-1.57	1.01
let-7d-5p	-1.53	-1.88	1.21	-1.23	-6.54	-4.40	-1.39	-4.69	-1.12	1.08
**let-7e-5p**	**-1.61**	**-2.07**	**4.68**	**1.02**	**-64.66**	**-26.97**	**3.24**	**-1.29**	**-1.83**	**-1.69**
let-7g-5p	0.00	0.00	0.00	0.00	13.08	11.11	4.44	0.00	-7.20	-8.97
miR-100-5p	2.01	2.92	-2.76	-7.57	-5.21	-2.73	2.26	-1.50	-2.25	-1.11
miR-101a-3p	2.03	-4.52	-2.06	-3.75	-3.24	-7.77	2.41	1.58	-3.15	-3.52
miR-101b-3p	0.00	0.00	-1.38	-3.76	-2.20	1.12	-15.69	-37.65	-5.42	-1.39
miR-125a-5p	2.37	2.52	-1.67	-1.56	-1.03	1.70	2.72	2.06	1.15	-1.02
miR-125b-5p	4.47	2.13	-2.37	-5.32	-1.31	-4.33	1.66	-1.02	1.15	2.27
**miR-126-3p**	**1.21**	**1.34**	**-1.22**	**-1.92**	**-2.62**	**-1.10**	**1.60**	**-1.40**	**-1.17**	**1.77**
miR-127-3p	1.58	-1.15	-1.07	-2.64	-2.48	-4.25	1.78	1.10	1.08	1.73
**miR-128a-3p**	**-1.50**	**-1.31**	**-3.55**	**-10.57**	**-4.55**	**-6.43**	**-1.44**	**-2.85**	**-1.30**	**1.09**
miR-129-3p	1.79	-1.13	-1.16	-3.66	-5.78	-3.24	-1.23	-1.92	-1.66	-1.01
**miR-132-3p**	**4.13**	**6.12**	**-1.54**	**-3.69**	**-1.20**	**1.33**	**4.57**	**1.40**	**-1.07**	**3.16**
miR-133a-3p	0.00	0.00	2.27	-1.70	-4.98	-1.85	-5.18	-5.20	-1.12	-1.45
miR-135a-1-3p	4.13	1.87	1.59	-2.74	-15.82	-1.08	-1.50	1.51	-6.37	1.03
miR-136-5p	2.15	1.47	1.21	-1.34	-2.62	1.82	-1.44	-2.52	-2.17	-1.81
miR-137-3p	2.57	2.02	-4.71	-5.32	-2.64	-4.92	-1.47	-1.24	-2.12	-1.64
miR-138-1-3p	-2.33	-4.03	-1.37	-4.79	-4.78	-4.25	-7.18	-6.49	-5.19	-1.93
miR-138-5p	2.38	1.67	-1.23	-1.86	-3.51	-3.18	-1.26	-2.15	-1.67	1.10
miR-139-5p	1.48	1.35	-2.43	-3.24	-2.50	-2.27	2.29	1.05	-1.07	1.41
**miR-140-5p**	**3.25**	**2.21**	**0.00**	**0.00**	**-10.85**	**-2.77**	**-1.19**	**-2.47**	**-1.06**	**-1.34**
miR-145-5p	5.71	1.53	-1.70	-3.12	-3.74	-1.46	1.02	1.68	1.18	2.52
miR-146a-5p	5.24	1.63	2.54	-1.85	12.87	13.07	-1.49	-7.58	4.12	3.18
miR-146b-5p	2.36	1.93	1.39	-1.89	-2.58	-1.13	1.57	-1.37	1.36	4.07
miR-150-5p	3.07	2.31	-2.49	-3.72	-1.72	1.28	-1.25	-1.52	2.08	2.43
**miR-16-5p**	**4.87**	**2.79**	**1.20**	**-1.89**	**0.00**	**0.00**	**1.68**	**-1.63**	**-2.38**	**-2.14**
miR-181a-5p	2.37	-1.44	-2.44	-2.34	0.00	0.00	1.70	-2.17	-1.71	-1.62
miR-186-5p	2.02	2.25	-1.18	-2.61	-3.12	1.32	2.41	0.00	0.00	0.00
miR-191-5p	2.16	2.18	-1.72	-3.07	-1.68	-1.58	2.43	1.11	-1.25	1.21
miR-193b-3p	3.41	4.28	-1.61	-3.71	-4.73	-2.04	1.71	1.18	1.02	1.31
**miR-195-5p**	**2.37**	**-2.08**	**1.75**	**-2.64**	**-1.92**	**-2.10**	**1.38**	**-1.45**	**1.60**	**-1.43**
miR-19b-3p	2.43	1.04	-2.40	-5.51	-2.91	-2.97	-1.45	-1.48	1.02	1.64
miR-204-5p	4.46	-2.02	1.24	-2.55	-26.07	-1.98	-1.66	-2.60	-1.02	-1.25
**miR-218-5p**	**2.54**	**1.61**	**-1.65**	**-2.62**	**-2.27**	**-2.35**	**1.52**	**-1.36**	**1.27**	**1.95**
miR-222-3p	2.02	1.82	-1.06	-1.30	-1.28	-1.48	1.21	-2.22	1.06	2.48
miR-22-3p	-2.04	-3.54	1.65	-4.04	-28.46	0.00	-1.48	1.07	-7.36	1.47
miR-24-3p	2.41	1.12	1.16	-1.66	-1.63	-1.09	-1.56	-2.32	-1.05	1.29
**miR-26a-5p**	**2.46**	**1.40**	**-1.05**	**-2.58**	**-2.42**	**-3.76**	**1.25**	**-1.33**	**-1.06**	**1.31**
**miR-29a-3p**	**2.56**	**1.47**	**1.28**	**-1.37**	**-2.53**	**-2.18**	**1.46**	**-2.07**	**-1.06**	**1.42**
miR-29b-1-5p	-1.41	1.12	1.13	-2.55	-5.68	-1.23	-6.07	-1.72	1.04	-2.56
miR-29c-3p	2.53	-1.10	-1.24	-2.68	-3.03	-2.91	-1.67	1.04	-1.12	1.24
miR-301a-3p	7.61	1.74	-1.62	-5.36	-1.13	-1.80	7.49	1.47	-2.30	-3.27
miR-30a-3p	2.50	2.93	-1.44	-2.86	-1.52	-1.79	2.43	3.90	1.11	1.16
miR-30a-5p	2.03	-1.46	-4.59	-5.60	-3.16	-2.98	1.94	-1.38	1.03	1.38
miR-30b-5p	2.24	1.63	-1.57	-3.66	-3.47	-3.03	1.53	1.12	-1.13	1.30
miR-30c-5p	2.58	1.78	1.16	-1.85	-3.00	-2.59	2.51	-1.06	-1.10	1.69
miR-30d-5p	1.50	-1.20	-2.10	-5.47	-3.51	-1.69	1.53	-1.63	1.11	2.89
miR-30e-3p	1.23	2.68	-1.33	-2.13	-1.89	-3.94	2.26	3.33	-1.33	-1.33
miR-30e-5p	1.03	-3.08	-1.22	-1.48	-3.42	-2.36	-1.85	-2.54	-1.40	-1.08
miR-31-5p	1.37	1.79	1.01	-2.60	-2.13	-1.53	-1.87	-2.84	1.24	-1.15
**miR-324-5p**	**0.00**	**0.00**	**-12.68**	**-1.01**	**3.25**	**2.00**	**9.05**	**-2.16**	**2.58**	**1.10**
**miR-328-3p**	**2.35**	**1.55**	**-1.78**	**-2.64**	**-2.42**	**-4.11**	**-1.27**	**-2.23**	**1.22**	**1.29**
miR-331-3p	3.11	1.51	1.01	-1.31	-2.12	-2.71	-1.04	-2.82	-1.13	1.36
miR-335-5p	1.45	1.10	0.00	0.00	0.00	0.00	-2.35	-1.10	-6.60	-7.40
miR-337-5p	0.00	0.00	0.00	-1.72	12.93	13.11	-4.13	-3.10	0.00	-3.57
miR-342-3p	11.85	9.32	2.00	1.07	1.23	2.65	3.91	1.61	2.49	3.88
**miR-365-3p**	**4.88**	**4.43**	**-3.03**	**-7.38**	**-3.34**	**-4.34**	**2.02**	**-2.92**	**0.00**	**0.00**
miR-376b-3p	-2.41	-9.54	2.50	-3.69	0.00	0.00	-4.69	-2.96	-2.45	-3.82
miR-376c-3p	1.89	1.80	1.03	-2.65	-2.24	-5.44	2.11	1.29	-4.77	-1.28
miR-379-5p	1.99	2.72	-1.27	-7.69	-3.41	-3.87	11.06	1.32	-1.20	-4.54
miR-384-5p	1.28	1.35	-3.57	-5.27	-2.42	-2.22	2.80	1.21	1.40	1.82
miR-411-5p	2.26	1.93	-1.15	-3.75	-1.75	1.05	1.80	-1.21	-1.40	2.83
**miR-434-3p**	**5.89**	**3.08**	**-1.21**	**-2.63**	**-1.56**	**-1.37**	**1.30**	**-1.83**	**-1.25**	**2.64**
miR-470-3p	0.00	0.00	0.00	0.00	0.00	0.00	-1.30	-1.86	-10.20	-11.43
miR-484	2.49	1.73	-2.09	-2.63	-1.96	1.21	-1.00	-1.90	1.68	1.38
miR-485-3p	1.63	1.59	1.01	-3.54	-1.94	-1.42	-2.13	-1.53	-2.85	-1.16
**miR-491-5p**	**-6.37**	**-7.68**	**0.00**	**0.00**	**0.00**	**0.00**	**0.00**	**0.00**	**-2.96**	**-1.42**
miR-495-3p	1.07	-1.02	-1.07	-2.58	-14.64	-12.97	3.98	1.05	-1.26	-2.87
**miR-544-5p**	**0.00**	**0.00**	**3.60**	**6.58**	**1.03**	**-1.67**	**0.00**	**0.00**	**1.35**	**-1.18**
miR-667-5p	1.32	1.25	-1.76	-1.81	-3.15	-2.21	-1.29	-3.00	-1.46	1.25
miR-674-3p	4.43	5.08	1.58	-1.51	-3.80	-2.97	-1.26	-1.13	2.57	1.35
miR-678	1.04	-1.05	-1.03	-3.46	-5.79	-1.22	-3.62	-1.17	-1.33	-2.19
miR-690	1.33	-2.17	1.06	-4.50	-17.02	-8.09	-1.85	-1.13	-7.12	-1.13
miR-709	1.19	-1.44	1.15	-3.46	-4.88	-4.20	-1.82	1.08	-3.15	-1.56
miR-720	-1.03	1.36	-2.60	-2.99	-1.57	-3.14	-2.96	-1.70	-1.15	1.18
miR-744-5p	1.60	-1.23	-1.71	2.42	-2.50	-2.02	-2.45	-17.42	1.40	-2.87
miR-760-5p	-1.65	-2.07	2.06	-1.67	0.00	0.00	-2.11	-2.14	0.00	-4.92
miR-7a-1-3p	2.32	1.76	-2.47	0.00	-1.94	-1.61	1.67	-1.18	-4.53	-1.41
**miR-7b-5p**	**1.51**	**3.66**	**-2.32**	**-4.93**	**11.27**	**1.95**	**0.00**	**0.00**	**0.00**	**0.00**
miR-801	4.01	0.00	0.00	0.00	-3.46	-6.03	1.29	2.89	-23.52	-6.41
miR-805	-1.23	-2.27	-1.03	-5.90	-3.27	-3.08	-2.77	-1.62	-2.34	-3.80
miR-872-3p	-1.59	-1.90	-1.76	-6.15	-1.64	-1.24	4.04	-2.08	1.69	-5.27
miR-9-3p	2.35	1.86	-1.23	-4.32	-5.24	-4.95	1.82	-1.17	-1.58	-1.19
miR-92a-3p	2.06	-6.10	1.21	-7.45	-3.34	-3.00	1.11	1.44	1.50	1.20
miR-9-5p	2.53	2.06	-2.27	-4.88	-1.55	-1.32	1.19	1.75	-1.38	1.01
miR-99b-5p	3.32	1.82	-2.59	-3.77	-4.78	-7.13	1.31	-1.29	-2.05	-1.52
