# Supplementary material for: Early Mechanisms of Pathobiology Are Revealed by Transcriptional Temporal Dynamics in Hippocampal CA1 Neurons of Prion Infected Mice
Source: PLoS Pathog. 2012 Nov 8;8(11):e1003002. doi: 10.1371/journal.ppat.1003002 (PMC3493483; doi:10.1371/journal.ppat.1003002)
Supplement: Table S7 — List of mature miRNA sequences for the 7 miRNAs that we successfully validated. MiRNA sequences in black represent mature mouse sequences while the ones in red represent rat sequences. (DOC) [file ppat.1003002.s015.doc]

| miRNA ID | Mature miRNA sequence |
| --- | --- |
| [mmu-miR-26a-5p](http://microrna.sanger.ac.uk/cgi-bin/sequences/query.pl?terms=mmu-miR-26a-5p) | 5’-UUCAAGUAAUCCAGGAUAGGCU  5’-UUCAAGUAAUCCAGGAUAGGCU |
| [mmu-miR-29a-3p](http://microrna.sanger.ac.uk/cgi-bin/sequences/query.pl?terms=mmu-miR-29a-3p) | 5’-UAGCACCAUCUGAAAUCGGUUA  5’-UAGCACCAUCUGAAAUCGGUUA |
| [mmu-miR-124-3p](http://microrna.sanger.ac.uk/cgi-bin/sequences/query.pl?terms=mmu-miR-124-3p) | 5’-UAAGGCACGCGGUGAAUGCC  5’-UAAGGCACGCGGUGAAUGCC |
| [mmu-miR-132-3p](http://microrna.sanger.ac.uk/cgi-bin/sequences/query.pl?terms=mmu-miR-132-3p) | 5’-UAACAGUCUACAGCCAUGGUCG  5’-UAACAGUCUACAGCCAUGGUCG |
| [mmu-miR-146a-5p](http://microrna.sanger.ac.uk/cgi-bin/sequences/query.pl?terms=mmu-miR-146a-5p) | 5’-[UGAGAACUGAAUUCCAUGGGUU](https://products.appliedbiosystems.com/ab/en/US/adirect/ab?cmd=ABAssayDetailDisplay&assayID=000468&Fs=y&SearchRequest.Common.PageNumber=1&assayType=taqman&chkBatchQueryText=false&srchType=keyword&searchValue=000468&searchBy=all&msgType=ABmiRNAKeywordResults)  5’-UGAGAACUGAAUUCCAUGGGUU |
| [mmu-miR-140-5p](http://microrna.sanger.ac.uk/cgi-bin/sequences/query.pl?terms=mmu-miR-140-5p) | 5’-CAGUGGUUUUACCCUAUGGUAG  5’-CAGUGGUUUUACCCUAUGGUAG |
| [mmu-miR-16-5p](http://microrna.sanger.ac.uk/cgi-bin/sequences/query.pl?terms=mmu-miR-16-5p) | 5’-UAGCAGCACGUAAAUAUUGGCG  5’-UAGCAGCACGUAAAUAUUGGCG |
